# Supplementary figures and images for: High-resolution prediction of American red squirrel in Interior Alaska: a role model for conservation using open access data, machine learning, GIS and LIDAR
Source: PeerJ. 2021 Sep 14;9:e11830. doi: 10.7717/peerj.11830 (PMC8447940; doi:10.7717/peerj.11830)

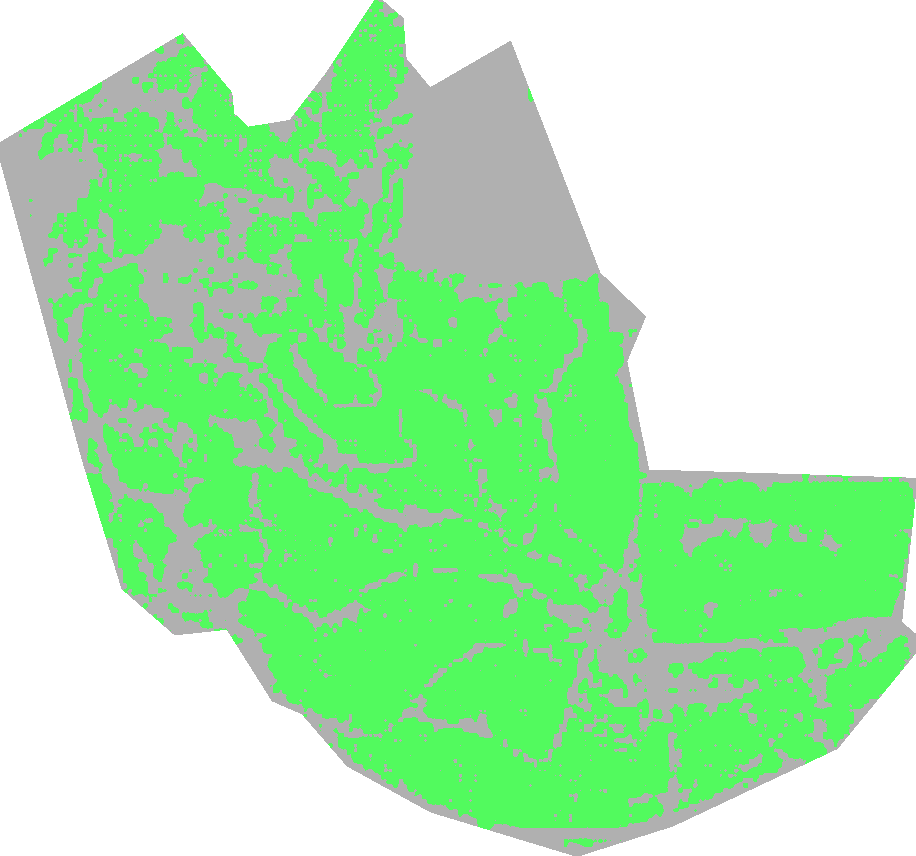

Supplement: Supplemental Information 7 — The data background to the binary map figure. The presence part contains 95% of all presence points. [file peerj-09-11830-s007.tif]

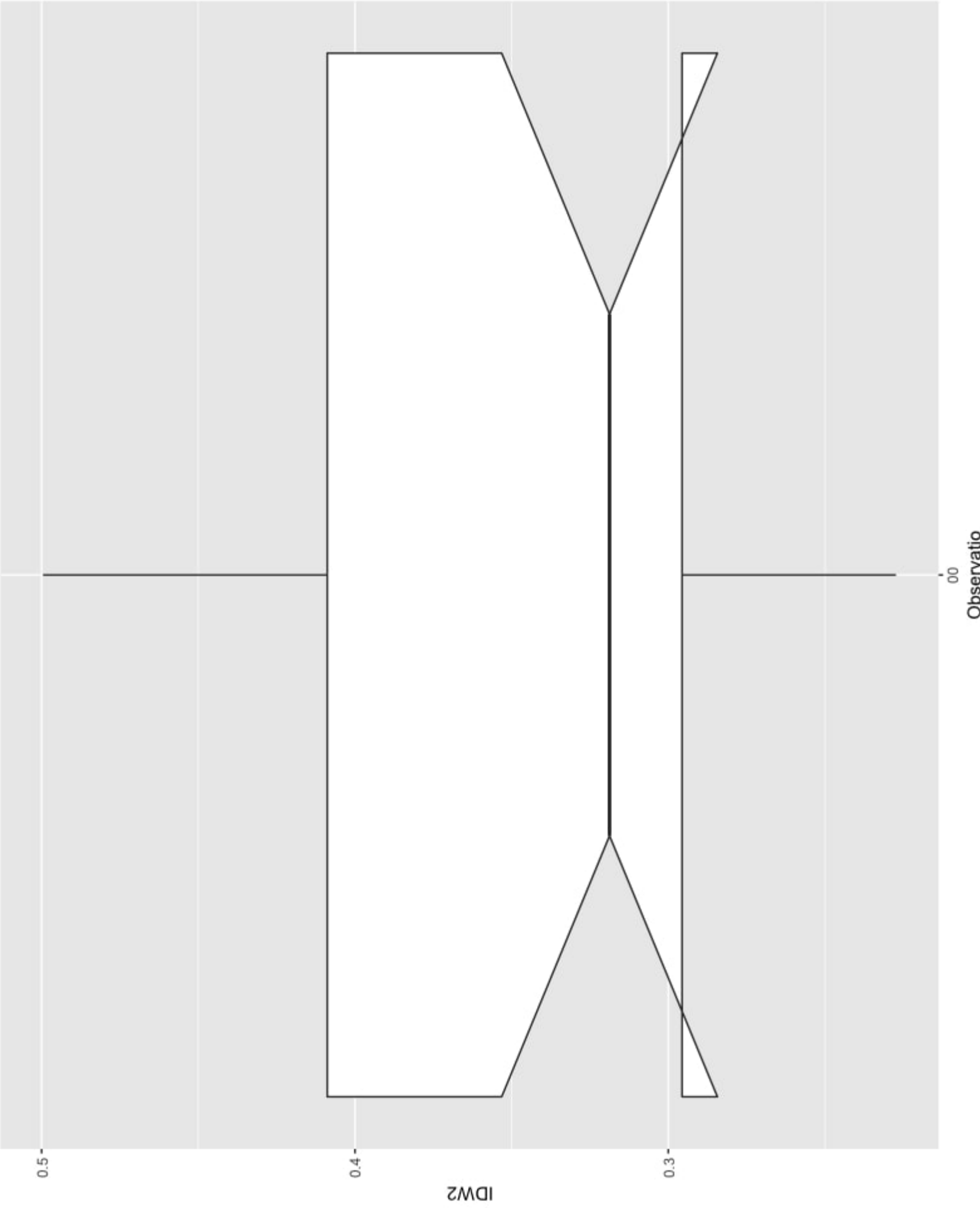

Supplement: Supplemental Information 16 — The RIO values of the squirrel midden presence points of the cruising survey in 2016. [file peerj-09-11830-s016.pdf]

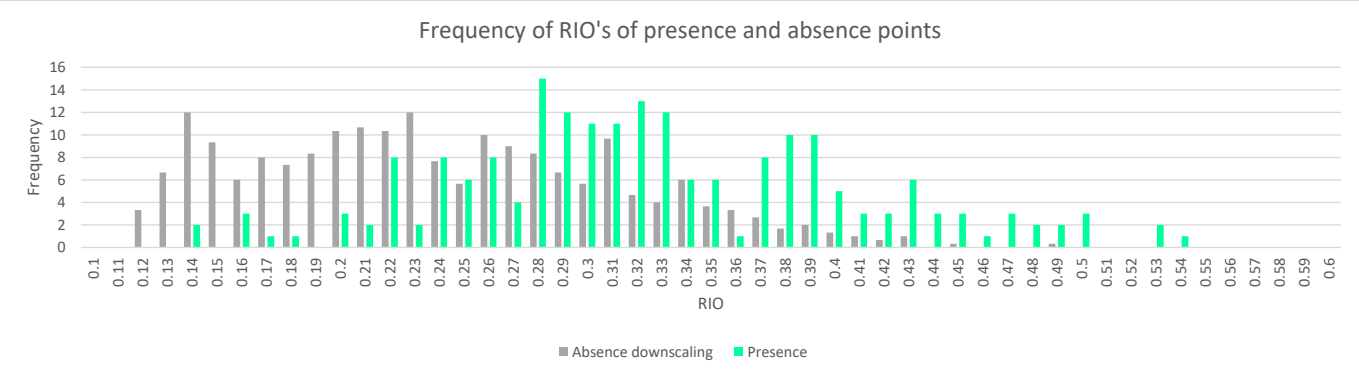

Supplement: Supplemental Information 19 — The histogram shows a histogram of presence values (green) compared to absence values (grey; divided by 3, because three times more absence values were used). It shows no clear separation but a strong indication of higher values for the presence points. [file peerj-09-11830-s019.pdf]

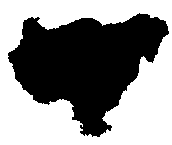

Supplement: Supplemental Information 23 — The lake next to the research area. This layer was used to extract the values of the local distance to the lake for the prediction. [file peerj-09-11830-s023.tif]
