# Supplementary material for: High-resolution prediction of American red squirrel in Interior Alaska: a role model for conservation using open access data, machine learning, GIS and LIDAR
Source: PeerJ. 2021 Sep 14;9:e11830. doi: 10.7717/peerj.11830 (PMC8447940; doi:10.7717/peerj.11830)
Supplement: Supplemental Information 25 [file peerj-09-11830-s025.rtf]

List of shape files and their content:Absence_points: This shapefile contains the location of the randomly generated absence points within the research area.Buildings_final: This shapefile shows the buildings within the research area. This layer was used to extract the values of the local distance to buildings for the prediction.Dogtrail: This shapefile contains frequently used dog trails within the research area. This layer was used to extract the values of the local distance to the dogtrails for the prediction.Grid_clipped: This lattice grid was used to extract local values of all predictor layers to calculate the prediction.Highway_final: This shapefile contains the highway next to the research area. This layer was used to extract the values of the local distance to the highway for the prediction.Lattice3_score: This shapefile contains the calculated prediction for each point of the lattice grid base. This layer was used to interpolate and with that generate a complete prediction for the relative occurrence of squirrel middens.midden_valid_2016_NAD83_N6: The midden locations collected for a university intern project in 2016 were used to validate the prediction. This shapefile contains the locations of all middens found in 2016.middens_clip: This shapefile contains all the locations were middens could be found within the research area during the survey for this project in 2017.Research_area: The area of interest were the research took placeRoute_walked: This shapefile contains the route that was used to survey the area and collect all the midden locations. Trails_final: This shapefile contains the trails within the research area that are frequently used by humans for recreational purposes. This layer was used to extract the values of the local distance to the frequently used trails for the prediction.
