# Supplementary material for: High-resolution prediction of American red squirrel in Interior Alaska: a role model for conservation using open access data, machine learning, GIS and LIDAR
Source: PeerJ. 2021 Sep 14;9:e11830. doi: 10.7717/peerj.11830 (PMC8447940; doi:10.7717/peerj.11830)

Z:\LIDAR_PROCESSING\6100401_Fairbanks_North_Star_Borough\QAQC\Control\PENZ_PPK_Adjusted_ASPZ3_usft_G06.xyz

Analyzed by: kkyzer on 9/13/2010 at 11:49:12 AM

| Number | Easting | Northing | Known Z | Laser Z | Dz | Dz^2 |
|---|---|---|---|---|---|---|
| 5527 | 1388588 | 4005429 | 737.802 | 737.62 | -0.182 | 0.033124 |
| 5528 | 1388625 | 4005465 | 738.378 | 738.29 | -0.088 | 0.007744 |
| 5536 | 1388930 | 4005772 | 742.083 | 742 | -0.083 | 0.006889 |
| 5541 | 1389119 | 4005964 | 744.429 | 744.35 | -0.079 | 0.006241 |
| 5531 | 1388740 | 4005581 | 739.446 | 739.37 | -0.076 | 0.005776 |
| 5538 | 1389005 | 4005848 | 743.057 | 742.99 | -0.067 | 0.004489 |
| 5532 | 1388779 | 4005620 | 739.993 | 739.93 | -0.063 | 0.003969 |
| 5548 | 1389381 | 4006229 | 748.271 | 748.21 | -0.061 | 0.003721 |
| 5540 | 1389081 | 4005926 | 743.984 | 743.93 | -0.054 | 0.002916 |
| 5534 | 1388855 | 4005696 | 741.136 | 741.1 | -0.036 | 0.001296 |
| 5544 | 1389229 | 4006075 | 746.043 | 746.01 | -0.033 | 0.001089 |
| 5550 | 1389458 | 4006308 | 749.138 | 749.11 | -0.028 | 0.000784 |
| 5533 | 1388818 | 4005659 | 740.687 | 740.68 | -0.007 | 0.000049 |
| 5542 | 1389155 | 4006000 | 744.941 | 744.94 | -0.001 | 0.000001 |
| 5543 | 1389191 | 4006037 | 745.551 | 745.55 | -0.001 | 0.000001 |
| 5529 | 1388664 | 4005504 | 738.759 | 738.77 | 0.011 | 0.000121 |
| 5546 | 1389306 | 4006153 | 747.245 | 747.26 | 0.015 | 0.000225 |
| 5524 | 1388467 | 4005315 | 735.988 | 736.01 | 0.022 | 0.000484 |
| 5551 | 1389498 | 4006349 | 749.535 | 749.56 | 0.025 | 0.000625 |
| 5545 | 1389268 | 4006115 | 746.769 | 746.8 | 0.031 | 0.000961 |
| 5539 | 1389043 | 4005887 | 743.524 | 743.56 | 0.036 | 0.001296 |
| 5549 | 1389421 | 4006270 | 748.671 | 748.71 | 0.039 | 0.001521 |
| 5526 | 1388548 | 4005390 | 737.248 | 737.29 | 0.042 | 0.001764 |
| 5547 | 1389342 | 4006191 | 747.67 | 747.72 | 0.05 | 0.0025 |
| 5537 | 1388968 | 4005811 | 742.589 | 742.64 | 0.051 | 0.002601 |
| 5530 | 1388703 | 4005543 | 739.068 | 739.12 | 0.052 | 0.002704 |
| 5525 | 1388508 | 4005352 | 736.478 | 736.56 | 0.082 | 0.006724 |
| 5535 | 1388893 | 4005734 | 741.603 | 741.75 | 0.147 | 0.021609 |

Statistical Summary

| | | |
|---|---|---|
| Mean: | -0.009 | feet |
| Median: | -0.001 | feet |
| Mode: | -0.001 | feet |
| Kurtosis: | 0.839 | |
| Skew: | -0.219 | |
| Minimum: | -0.182 | feet |
| Maximum: | 0.147 | feet |
| Range: | 0.329 | feet |
| Count: | 28 | |
| | | |
| RMSE: | 0.066 | |
| **RMSE*1.96 (95% CI - NSSDA)** | **0.129** | |

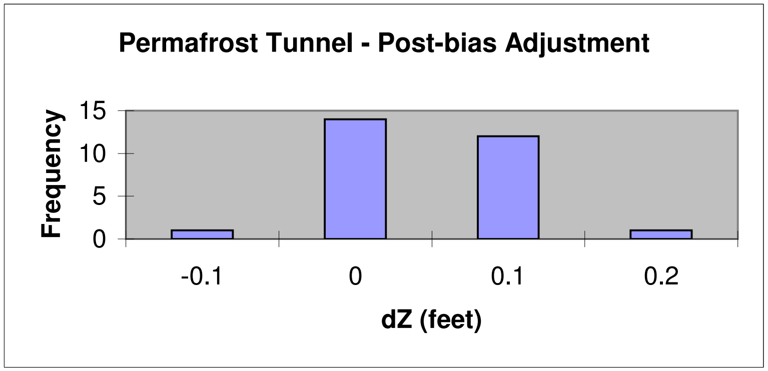

Supplement: Supplemental Information 26 — All metadata contents provided by the Alaska Division for Geographic and Geophysical Surveys for the used LIDAR dataset. [file peerj-09-11830-s026.zip › metadata/Permafrost_Tunnel_QC_Results.pdf]
