# Supplementary material for: High-resolution prediction of American red squirrel in Interior Alaska: a role model for conservation using open access data, machine learning, GIS and LIDAR
Source: PeerJ. 2021 Sep 14;9:e11830. doi: 10.7717/peerj.11830 (PMC8447940; doi:10.7717/peerj.11830)
Supplement: Supplemental Information 26 — All metadata contents provided by the Alaska Division for Geographic and Geophysical Surveys for the used LIDAR dataset. [file peerj-09-11830-s026.zip › metadata/Permafrost_Tunnel_Lidar_Report.pdf]

PROCEDURAL REPORT

FOR

CRREL PERMAFROST TUNNEL LIDAR

AERO-METRIC, INC.

NOVEMBER 2010

Table of Contents

Overview 1
Lidar Acquisition Planning 1
Lidar Acquisition 1
Lidar Processing 2
Lidar Accuracy 3
Deliverables 4
Conclusions 4

Overview

DOWL/HKM awarded Aero-Metric, Inc. a contract to acquire and produce lidar derived mapping products, for the Fairbanks North Star Borough Area, including the CRREL Permafrost Tunnel. This report will outline the planning, processing and quality control procedures used for the Permafrost Tunnel portion of the project. The lidar data was merged with ground data collected by DOWL/HKM in the southeast corner of the project area.

Lidar Acquisition Planning

The lidar data for this project was collected with Aero-Metric's Optech Gemini-167 Airborne lidar system (Serial Number 03SEN145). All flight planning and acquisition was completed using Optech's ALTM-Nav, version 1.0.67b (flight planning and lidar control software). The nominal point spacing for this project was 2.7 feet. The following are the planned lidar settings for acquiring the data for this project.

- Flying Height (Above Ground): 4600 feet
- Laser Pulse Rate: 70 kHz
- Mirror Scan Frequency: 48 Hz
- Scan Angle (+/-): 12°
- Side Lap: 50 %
- Ground Speed: 150 kts

Lidar Acquisition

The following are the data for this area was collect on May 6, 2010, under mission number 43Q12610A.

DOWL/HKM provided two static GPS base stations located in the project area for airborne GPS control. These points, in conjunction with the two stations operated by Aero-Metric at the Fairbanks airport constituted the basis for the airborne GPS/IMU control.

Ground Survey Data

DOWL/HKM collected ground survey data in the southeast corner of the project area in order to fill a void in the lidar acquisition. The data was collected on November 11, 2010. Below is a map showing the extent of the surveyed data as part of the entire dataset. The area surveyed was approximately 1.3 acres of the project area.

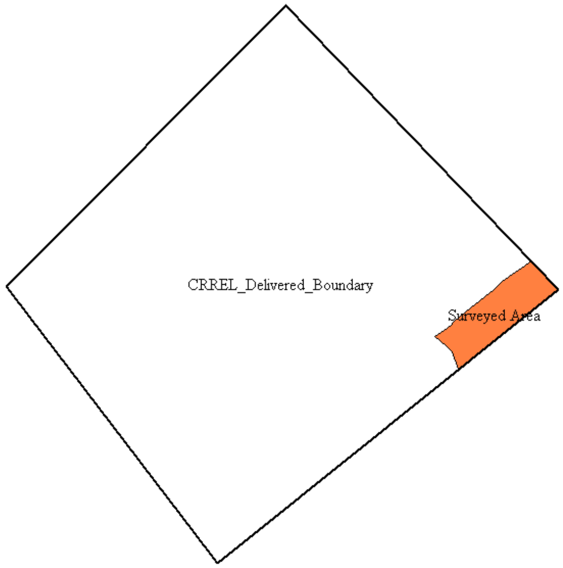

Lidar Processing

The raw lidar data was merged with the processed ABGPS/IMU data using Optech's ASDA software, which computes an adjusted lidar point cloud for each flight line. Each flight line was then verified for data coverage and consistency with the adjacent flight lines.

Automated classification algorithms in TerraSolid's TerraScan, version 9.011 were used to produce the majority of the bare-earth dataset. The remainder of the data was classified using manual classification techniques.

The manually surveyed data points and breaklines were merged into the lidar data and classified as ground points (LAS class 2), with bit 5 turned on, indicating these points are synthetic (not created by lidar) ground points.

The digital elevation models and contours were created from the ground classified lidar points using LP360 (QCoherent Software, LLC.). These were converted to Arc GeoDatabase format.

Lidar Accuracy

The final lidar dataset was verified using 28 GPS surveyed ground truth points. These points were collected by DOWL/HKM for use in this project. The ground truth points were compared against the lidar using TerraScan, which computes the difference between each ground truth point and the lidar generated surface. These differences are recorded in an output file in ASCII format. This file is imported into Microsoft Excel, where a statistical analysis is performed. The full lidar control check analysis is included in the deliverables for this project, named: Permafrost_Tunnel_QC_Results.pdf

For this dataset the vertical accuracy, assessed at the 95% confidence interval (1.96 x RMSE) was 0.129 feet.

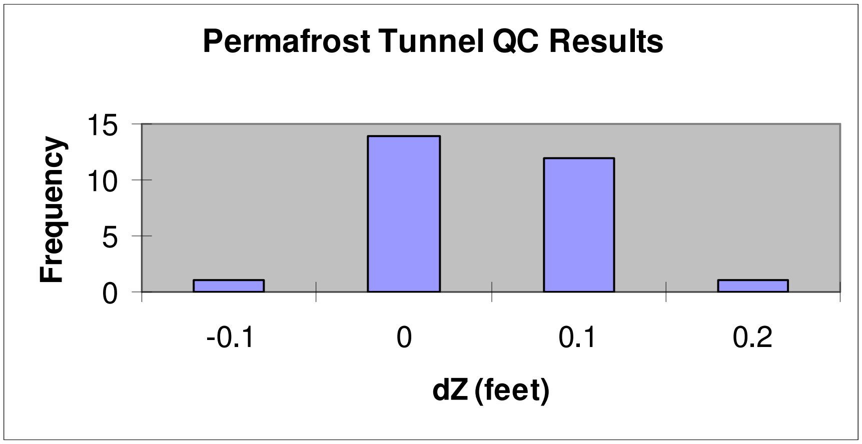

Figure 1: Histogram of QC data check

Deliverables

Bare-Earth Digital Elevation Model: Derived from the bare-earth processed data, it is in Arc GeoDatabase format. The following are the deliverable parameters:

- Grid Resolution: 4 feet
- 32 bit floating point rasters

LAS Point Cloud: Classified lidar point cloud data, in LAS v1.2 format.

- Included Classes:
    - Class 1: Unclassified
    - Class 2: Ground
    - Class 12: Overlap (Unclassified)
- Delivered as on file: CRREL_Permafrost_Tunnel.las

Contours: 2 foot contours in Arc GeoDatabase format .

FDGC Compliant Metadata: Deliverable level metadata for all delivered lidar datasets, in .xml format.

Reports: Three .PDF files:

1. ***100109_LIDAR_Calibration_Report.pdf*** is the sensor calibration and testing report.
2. ***Permafrost_Tunnel_QC_Results.pdf*** is the lidar control check analysis.
3. ***Permafrost_Tunnel_Lidar_Report.pdf*** is the procedural report (this document).

Boundary: Shapefile depicting the extents of the deliverables.

Conclusions

The lidar data included in this delivery was acquired for a planned point spacing of approximately 2.7 feet. The vertical accuracy, assessed at the 95% confidence interval was 0.129 feet, on hard, open surfaces. The accuracy of the lidar data was not verified on all surface types, and accuracies may be degraded in areas of steep terrain or heavy vegetation.
