# Supplementary material for: High-resolution prediction of American red squirrel in Interior Alaska: a role model for conservation using open access data, machine learning, GIS and LIDAR
Source: PeerJ. 2021 Sep 14;9:e11830. doi: 10.7717/peerj.11830 (PMC8447940; doi:10.7717/peerj.11830)
Supplement: Supplemental Information 26 — All metadata contents provided by the Alaska Division for Geographic and Geophysical Surveys for the used LIDAR dataset. [file peerj-09-11830-s026.zip › metadata/FNSB_LiDAR-MAPPING_Survey-Report_2010-11-02.pdf]

Alaska District

CEPOA-CT
Contract: W911KB-07-D-0001
D.O. No. 0038

Topographic Survey

Fairbanks North Star Borough LiDAR Mapping Ft. Wainwright, Alaska

October 2010

SURVEY REPORT

DOWL ENGINEERS

4041 B Street, Anchorage, AK 99503
Phone (907) 562-2000 Fax (907) 563-3953
www.dowlhkm.com

DOWL WO# 1127.60548.01
Field Books: 2330, 2331, 2332, 2333

FAIRBANKS NORTHSTAR BOROUGH LIDAR MAPPING

FAIRBANKS, ALASKA

AUGUST, 2010

HORIZONTAL CONTROL

Coordinates are NAD83(2007.00) Alaska State Plane Coordinates, Zone 3, in US Feet. The Basis of Coordinates is the NAD83(2007.00) ASPC Zone 3 position of NGS Primary Airport Control Station "FAI A", PID DF3640 as retrieved from the NGS database on July 1, 2008, a 3-1/4" brass cap pipe monument, having coordinates of N=3,956,198.10967, E=1,351,803.19115 US Feet. Other horizontal positions shown hereon were determined by differential static GPS observations using Leica dual frequency receivers during the period of July 2008 and May 2010.

The Project Combined Scale Factor is 0.99994797534. To convert ground distances to ASP grid distances, multiply by a combined scale factor of 0.99994797534. To convert ASP grid distances to ground distances, multiply by a combined scale factor of 100000000000/99994797534.

VERTICAL CONTROL

Elevations are NAVD88 in US Feet. The Basis of Elevations are USC&GS Bench Mark "G 121", PID TT2770, a 3-3/4" brass cap on stainless steel rod monument, having a value of 499.66 US Feet and USEO Bench Mark "DIKE 23 USE", PID TT2766, a brass cap having a value of 490.90 US Feet; as retreived from the NGS database on July 1, 2008. Other elevations are derived from differential leveling using a Leica DNA10 digital level with fiberglass barcode rod, or differential static GPS observations using Leica dual frequency GPS receivers. Elevations derived from differential static GPS observations or RTK GPS positioning were computed using GEOID06 orthometric heights, and adding a bias of 0.080 feet to the GEOID06 othrometric height to derive NAVD88 elevations.

LIDAR MAPPING

A bare-earth Digital Elevation Model (DEM) for this project was generated by Aero-Metric, Inc. of Anchorage, Alaska from Light Detection and Ranging (LiDAR) data acquired by them on **May 4th and May 6th, 2010**. Said DEM is of a quality that meets or exceeds ASPRS Class II standards for the production **of 2-foot coutours** in areas of moderate terrain and minimal vegetation. Areas with steep terrain or areas obscured by dense vegetation may be less accurate.

CHECK SHOT SURVEYS

DOWL HKM performed check shot surveys based on existing site control monuments, at various locations throughout the mapping limits in April and May of 2010. Check shot surveys were performed using Real Time Kinematic (RTK) GPS methods and appropriate quality assurance procedures. Check shot surveys result in the following accuracy statements applicable to the terrain and ground cover in the areas that were sampled.

86 check shots tested 0.26 feet fundamental vertical accuracy at 95 percent confidence level on a paved surface using RMSE x 1.9600.

308 check shots tested 0.35 feet fundamental vertical accuracy at 95 percent confidence level in open terrain using RMSE x 1.9600.

63 check shots tested 0.59 feet supplemental vertical accuracy at 95 percent confidence level in grass covered terrain using RMSE x 1.9600.

43 check shots tested 0.55 feet supplemental vertical accuracy at 95 percent confidence level in brush covered terrain using RMSE x 1.9600.

85 check shots tested 0.93 feet supplemental vertical accuracy at 95 percent confidence level in forest covered terrain using RMSE x 1.9600.

SURVEY CONTROL POINT LISTING
FAIRBANKS NORTH STAR BOROUGH
LiDAR MAPPING PROJECT
FAIRBANKS, ALASKA

Horizontal Control - NAD83(2007.00) Alaska State Plane Coordinates, Zone 3, USFeet

Coordinates are NAD83(2007.00) Alaska State Plane Coordinates, Zone 3, in US Feet. The Basis of Coordinates is the NAD83(2007.00) ASPC Zone 3 position of NGS Primary Airport Control Station "FAI A", PID DF3640 as retrieved from the NGS database on July 1, 2008, a 3-1/4" brass cap pipe monument, having coordinates of N=3956198.10967, E=1351803.19115 US Feet. Other horizontal positions shown hereon were determined by differential static GPS observations using Leica dual frequency receivers during the period of July 2008 and May 2010.

Scale Factor

The Project Combined Scale Factor is 0.99994797534. To convert ground distances to Alaska State Plane Zone 3 (ASPCZ3) grid distances, multiply by a combined scale factor of 0.99994797534. To convert ASPZ3 grid distances to ground distances, multiply by a combined scale factor of 100000000000/99994797534.

Vertical Control - NAVD88, US Feet

Elevations are NAVD88 in US Feet. The Basis of Elevations are USC&GS Bench Mark "G 121", PID TT2770, a 3-3/4" brass cap on stainless steel rod monument, having a value of 499.66 US Feet and USEO Bench Mark "DIKE 23 USE", PID TT2766, a brass cap having a value of 490.90 US Feet; as retreived from the NGS database on July 1, 2008. Other elevations are derived from differential leveling using a Leica DNA10 digital level with fiberglass barcode rod (Lev.), or differential static GPS observations using Leica dual frequency GPS receivers (GPS), or real time kinematic GPS positioning using the same receivers (RTK), as noted, during the period of July 27 through August 15, 2008, and during the period of April 19 through May 22, 2010. Elevations derived from differential static GPS observations or RTK GPS positioning were computed using GEOID06 orthometric heights, and adding a bias of 0.080 feet to the GEOID06 orthometric height to derive NAVD88 elevations.

Primary Control Monuments Held

ID	PID	Northing	Easting	Elevation	Lev./GPS	Description	Survey Point #
**FAI A**	**DF3640**	**3,956,198.11**	**1,351,803.19**	**431.54**	**Lev.**	**Brass Cap**	**551**

LIDAR Control Monuments

ID	PID	Northing	Easting	Elevation	Lev./GPS	Description	Survey Point #
BUNKER		3,933,338.50	1,449,701.88	519.03	Lev.	Brass Cap	1
POT STA 400+97.57		3,945,308.17	1,456,120.73	531.35	Lev.	Aluminum Mon	729

SURVEY CONTROL POINT LISTING
FAIRBANKS NORTH STAR BOROUGH
LiDAR MAPPING PROJECT
FAIRBANKS, ALASKA

Control Monuments

ID	PID	Northing	Easting	Elevation	Lev./GPS	Description	Survey Point #
COLLAGE	TT2844	3,971,376.33	1,357,412.48	522.8	GPS	Brass Cap	705
CRREL		4,005,859.39	1,388,940.07	739.3	GPS	Alcap	403
FAIR-6		3,931,066.98	1,429,017.38	494.4	GPS	Brass Cap	706
GATE		3,908,409.98	1,464,435.64	533.5	GPS	Aluminum Mon	734
GPS CHS 6.4		3,983,144.21	1,420,167.39	611.3	GPS	Aluminum Mon	709
GPS TLC-1		3,949,230.54	1,380,130.37	459.18	Lev.	Alcap on rod	702
INITIAL	TT6711	3,973,388.07	1,384,880.56	1,100.6	GPS	Brass Cap	552
PC STA 112+13.17		3,920,927.56	1,441,394.27	525.87	Lev.	Aluminum Mon	708
PI 3 (Tanana)		3,924,815.07	1,428,047.05	497.1	GPS	Brass Cap	707
Q 121	TT2808	4,004,587.87	1,385,264.27	721.4	GPS	Brass Cap	741
RIDGE	DH4891	3,969,263.39	1,350,260.85	458.2	GPS	Brass Cap/Drive Rod	703
U 53	TT2896	3,954,070.11	1,394,742.74	456.8	GPS	Brass Cap	701
XS-TLC-3R		3,947,075.41	1,333,084.66	547.9	GPS	Alcap	704
401		3,959,232.85	1,355,915.14	434.1	GPS	Magnail	401
402		3,920,248.91	1,439,563.53	514.9	GPS	Magnail	402

SURVEY CONTROL POINT LISTING
FAIRBANKS NORTH STAR BOROUGH
LiDAR MAPPING PROJECT
FAIRBANKS, ALASKA

GPS, Traverse, and Sideshot Summaries

File Name	Basis of Bearings	Includes Points	Raw Angular Error Per/Set	Raw Error of Closure	Error of Closure After Adj.
STS01	Static GPS	BUNKER, COLLAGE, CRREL, FAI A, FAIR-6, GATE, CHS 6.4, RIDGE, GPS TLC-1, INITIAL, PC STA 112+13.17, PI 3, POT STA 400+97.57, Q 121, U 53, XS-TLC-3R, 401, 402	N/A	N/A	N/A

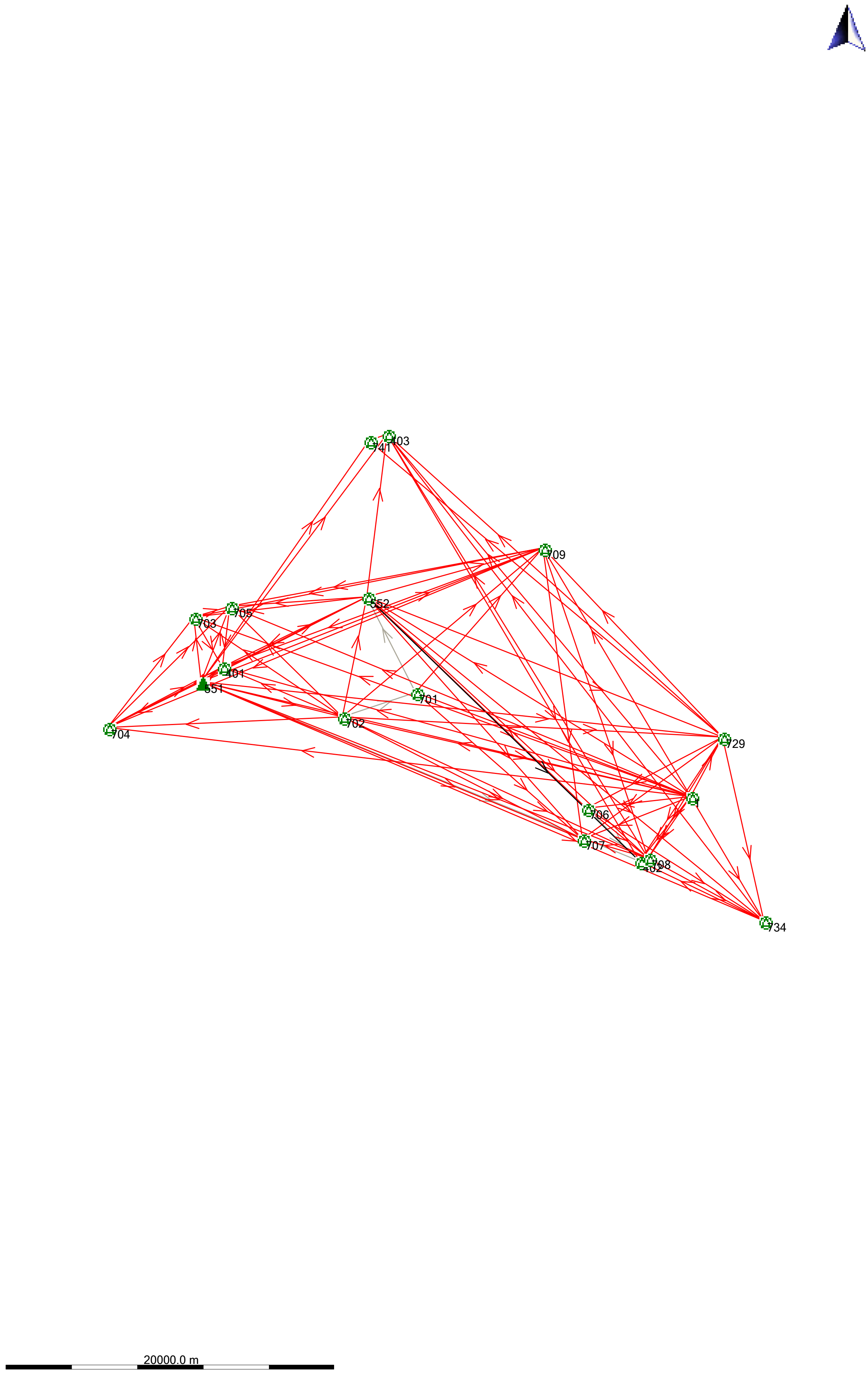

Estimated
Navigated
SPP
Measured
Average
Reference
Adjusted
Control - 1D
Control - 2D
Control - 3D

Point Id	Point Class	Latitude	Longitude	Ellip. Hgt.	Ortho. Hgt.	Code
☑ 1	Adjusted	64° 45' 28.83430" N	147° 13' 16.86361" W	550.27792	519.03455	CNTL/HVPT.BCMON
☑ 401	Adjusted	64° 49' 21.41286" N	147° 49' 35.18762" W	465.23547	434.13974	CNTL/HCPT.MAG
☑ 402	Adjusted	64° 43' 18.04977" N	147° 17' 04.41267" W	546.24248	514.89412	CNTL/HCPT.MAG
☑ 403	Adjusted	64° 57' 09.00166" N	147° 37' 19.95371" W	770.18689	739.28144	CNTL/HCPT.ALCAP
☑ 551	Control	64° 48' 50.38170" N	147° 51' 08.10888" W	462.53544	431.37737	CNTL/HVPT.BCMON
☑ 552	Control	64° 51' 48.48997" N	147° 38' 34.60171" W	1131.47024	1100.57791	CNTL/HVPT.BCMON
☑ 701	Adjusted	64° 48' 40.88746" N	147° 34' 35.30747" W	487.74739	456.84522	CNTL/HVPT.BCMON
☑ 702	Control	64° 47' 49.58474" N	147° 40' 09.75174" W	490.06633	459.07230	CNTL/HVPT.ALMON
☑ 703	Adjusted	64° 50' 58.46942" N	147° 51' 52.60864" W	489.31767	458.18912	CNTL/HVPT.BCMON
☑ 704	Adjusted	64° 47' 15.07652" N	147° 58' 13.72314" W	579.34802	547.86515	CNTL/HCPT.ALCAP
☑ 705	Adjusted	64° 51' 21.30427" N	147° 49' 08.66623" W	553.84594	522.80597	CNTL/HVPT.BCMON
☑ 706	Control	64° 45' 02.34370" N	147° 21' 12.46525" W	525.45798	494.33271	CNTL/HVPT.BCMON
☑ 707	Control	64° 44' 00.61851" N	147° 21' 31.73227" W	528.27448	497.06063	CNTL/HCPT.BCMON
☑ 708	Control	64° 43' 25.09175" N	147° 16' 22.58812" W	557.22116	525.86952	CNTL/HVPT.ALMON
☑ 709	Adjusted	64° 53' 32.88236" N	147° 25' 03.24280" W	642.15174	611.32175	CNTL/HCPT.ALMON
☑ 729	Adjusted	64° 47' 27.82469" N	147° 10' 54.06599" W	562.57286	531.40166	CNTL/HVPT.ALMON
☑ 734	Adjusted	64° 41' 26.19579" N	147° 07' 27.06885" W	565.12427	533.44782	CNTL/HVPT.ALMON
☑ 741	Adjusted	64° 56' 55.55680" N	147° 38' 44.50502" W	752.27131	721.37570	CNTL/HVPT.BCMON

Point Id	Point Class	Northing	Easting	Ellip. Hgt.	Ortho. Hgt.	Code
☑ 1	Adjusted	3933338.50085	1449701.87531	550.27792	519.03455	CNTL/HVPT.BCMON
☑ 401	Adjusted	3959232.85454	1355915.14279	465.23547	434.13974	CNTL/HCPT.MAG
☑ 402	Adjusted	3920248.91167	1439563.50985	546.24248	514.89412	CNTL/HCPT.MAG
☑ 403	Adjusted	4005859.39038	1388940.07359	770.18689	739.28144	CNTL/HCPT.ALCAP
☑ 551	Control	3956198.10993	1351803.19116	462.53544	431.37737	CNTL/HVPT.BCMON
☑ 552	Control	3973388.06945	1384880.56272	1131.47024	1100.57791	CNTL/HVPT.BCMON
☑ 701	Adjusted	3954070.11303	1394742.73703	487.74739	456.84522	CNTL/HVPT.BCMON
☑ 702	Control	3949230.53903	1380130.36728	490.06633	459.07230	CNTL/HVPT.ALMON
☑ 703	Adjusted	3969263.38809	1350260.85091	489.31767	458.18912	CNTL/HVPT.BCMON
☑ 704	Adjusted	3947075.41180	1333084.65809	579.34802	547.86515	CNTL/HCPT.ALCAP
☑ 705	Adjusted	3971376.33223	1357412.47709	553.84594	522.80597	CNTL/HVPT.BCMON
☑ 706	Control	3931066.97509	1429017.38329	525.45798	494.33271	CNTL/HVPT.BCMON
☑ 707	Control	3924815.06847	1428047.05091	528.27448	497.06063	CNTL/HCPT.BCMON
☑ 708	Control	3920927.55885	1441394.26674	557.22116	525.86952	CNTL/HVPT.ALMON
☑ 709	Adjusted	3983144.20855	1420167.38735	642.15174	611.32175	CNTL/HCPT.ALMON
☑ 729	Adjusted	3945308.16549	1456120.72917	562.57286	531.40166	CNTL/HVPT.ALMON
☑ 734	Adjusted	3908409.97610	1464435.63516	565.12427	533.44782	CNTL/HVPT.ALMON
☑ 741	Adjusted	4004587.86836	1385264.26926	752.27131	721.37570	CNTL/HVPT.BCMON

Processing Summary
1127.60548.01 FNSB LiDAR Mapping STS01

Project Information

Project name:	1127.60548.01 FNSB LiDAR Mapping STS01
Date created:	04/22/2010 00:21:31
Time zone:	-8h 00'
Coordinate system name:	AK Zone 3 NAD83 Geoid09
Application software:	LEICA Geo Office 5.0
Start date and time:	04/21/2010 07:29:20
End date and time:	04/24/2010 17:30:25
Manually occupied points:	139
Processing kernel:	PSI-Pro 2.0
Processed:	04/25/2010 22:18:07

Processing Parameters

**Parameters**	**Selected**
Cut-off angle:	13°
Ephemeris type:	Broadcast
Solution type:	Automatic
GNSS type:	Automatic
Frequency:	Automatic
Fix ambiguities up to:	80 km
Min. duration for float solution (static):	5' 00"
Sampling rate:	Use all
Tropospheric model:	Hopfield
Ionospheric model:	Automatic
Use stochastic modelling:	Yes
Min. distance:	8 km
Ionospheric activity:	Automatic

Baseline Overview

**551 - 1**	**Reference: 551**	**Rover: 1**
Coordinates:		
Latitude:	64° 48' 50.38170" N	64° 45' 28.83428" N
Longitude:	147° 51' 08.10888" W	147° 13' 16.86415" W
Ellip. Hgt:	462.53516 fts	550.31586 fts
Solution type:	Phase: all fix	
GNSS type:	GPS	
Frequency:	IonoFree (L3)	
Ambiguity:	Yes	

**551 - 701**	**Reference: 551**	**Rover: 701**

Coordinates:		
Latitude:	64° 48' 50.38170" N	64° 48' 40.89406" N
Longitude:	147° 51' 08.10888" W	147° 34' 35.27404" W
Ellip. Hgt:	462.53516 fts	486.65172 fts
Solution type:	Float	
GNSS type:	GPS	
Frequency:	L1 and L2	
Ambiguity:	No	
**551 - 702**	**Reference: 551**	**Rover: 702**
Coordinates:		
Latitude:	64° 48' 50.38170" N	64° 47' 49.58460" N
Longitude:	147° 51' 08.10888" W	147° 40' 09.75184" W
Ellip. Hgt:	462.53516 fts	490.10450 fts
Solution type:	Phase: all fix	
GNSS type:	GPS	
Frequency:	L1 and L2	
Ambiguity:	Yes	
**702 - 701**	**Reference: 702**	**Rover: 701**
Coordinates:		
Latitude:	64° 47' 49.58460" N	64° 48' 40.88790" N
Longitude:	147° 40' 09.75184" W	147° 34' 35.30800" W
Ellip. Hgt:	490.10450 fts	487.75821 fts
Solution type:	Phase: all fix	
GNSS type:	GPS	
Frequency:	L1 and L2	
Ambiguity:	Yes	
**551 - 552**	**Reference: 551**	**Rover: 552**
Coordinates:		
Latitude:	64° 48' 50.38170" N	64° 51' 48.48987" N
Longitude:	147° 51' 08.10888" W	147° 38' 34.60198" W
Ellip. Hgt:	462.53516 fts	1131.51378 fts
Solution type:	Phase: all fix	
GNSS type:	GPS	
Frequency:	L1 and L2	
Ambiguity:	Yes	
**1 - 729**	**Reference: 1**	**Rover: 729**
Coordinates:		
Latitude:	64° 45' 28.83428" N	64° 47' 27.82474" N
Longitude:	147° 13' 16.86415" W	147° 10' 54.06641" W
Ellip. Hgt:	550.31586 fts	562.63096 fts
Solution type:	Phase: all fix	
GNSS type:	GPS	
Frequency:	L1 and L2	
Ambiguity:	Yes	
**701 - 552**	**Reference: 701**	**Rover: 552**

Coordinates:		
Latitude:	64° 48' 40.88790" N	64° 51' 48.49073" N
Longitude:	147° 34' 35.30800" W	147° 38' 34.60217" W
Ellip. Hgt:	487.75821 fts	1131.43931 fts
Solution type:	Phase: all fix	
GNSS type:	GPS	
Frequency:	L1 and L2	
Ambiguity:	Yes	
**1 - 702**	**Reference: 1**	**Rover: 702**
Coordinates:		
Latitude:	64° 45' 28.83428" N	64° 47' 49.58439" N
Longitude:	147° 13' 16.86415" W	147° 40' 09.75230" W
Ellip. Hgt:	550.31586 fts	490.04636 fts
Solution type:	Phase: all fix	
GNSS type:	GPS	
Frequency:	IonoFree (L3)	
Ambiguity:	Yes	
**702 - 552**	**Reference: 702**	**Rover: 552**
Coordinates:		
Latitude:	64° 47' 49.58460" N	64° 51' 48.48987" N
Longitude:	147° 40' 09.75184" W	147° 38' 34.60195" W
Ellip. Hgt:	490.10450 fts	1131.51447 fts
Solution type:	Phase: all fix	
GNSS type:	GPS	
Frequency:	L1 and L2	
Ambiguity:	Yes	
**1 - 729**	**Reference: 1**	**Rover: 729**
Coordinates:		
Latitude:	64° 45' 28.83428" N	64° 47' 27.82467" N
Longitude:	147° 13' 16.86415" W	147° 10' 54.06654" W
Ellip. Hgt:	550.31586 fts	562.58975 fts
Solution type:	Phase: all fix	
GNSS type:	GPS	
Frequency:	L1 and L2	
Ambiguity:	Yes	
**1 - 701**	**Reference: 1**	**Rover: 701**
Coordinates:		
Latitude:	64° 45' 28.83428" N	64° 48' 40.88730" N
Longitude:	147° 13' 16.86415" W	147° 34' 35.30836" W
Ellip. Hgt:	550.31586 fts	487.73659 fts
Solution type:	Phase: all fix	
GNSS type:	GPS	
Frequency:	IonoFree (L3)	
Ambiguity:	Yes	
**551 - 709**	**Reference: 551**	**Rover: 709**

Coordinates:		
Latitude:	64° 48' 50.38170" N	64° 53' 32.88214" N
Longitude:	147° 51' 08.10888" W	147° 25' 03.24325" W
Ellip. Hgt:	462.53516 fts	641.05334 fts
Solution type:	Phase: all fix	
GNSS type:	GPS	
Frequency:	IonoFree (L3)	
Ambiguity:	Yes	

**702 - 709**	**Reference: 702**	**Rover: 709**
Coordinates:		
Latitude:	64° 47' 49.58460" N	64° 53' 32.88232" N
Longitude:	147° 40' 09.75184" W	147° 25' 03.24286" W
Ellip. Hgt:	490.10450 fts	641.11972 fts
Solution type:	Phase: all fix	
GNSS type:	GPS	
Frequency:	IonoFree (L3)	
Ambiguity:	Yes	

**701 - 709**	**Reference: 701**	**Rover: 709**
Coordinates:		
Latitude:	64° 48' 40.88790" N	64° 53' 32.88292" N
Longitude:	147° 34' 35.30800" W	147° 25' 03.24315" W
Ellip. Hgt:	487.75821 fts	641.01112 fts
Solution type:	Phase: all fix	
GNSS type:	GPS	
Frequency:	L1 and L2	
Ambiguity:	Yes	

**552 - 709**	**Reference: 552**	**Rover: 709**
Coordinates:		
Latitude:	64° 51' 48.48988" N	64° 53' 32.88260" N
Longitude:	147° 38' 34.60197" W	147° 25' 03.24289" W
Ellip. Hgt:	1131.51230 fts	641.04688 fts
Solution type:	Phase: all fix	
GNSS type:	GPS	
Frequency:	L1 and L2	
Ambiguity:	Yes	

**729 - 706**	**Reference: 729**	**Rover: 706**
Coordinates:		
Latitude:	64° 47' 27.82470" N	64° 45' 02.34373" N
Longitude:	147° 10' 54.06648" W	147° 21' 12.46555" W
Ellip. Hgt:	562.60888 fts	525.62815 fts
Solution type:	Phase: all fix	
GNSS type:	GPS	
Frequency:	L1 and L2	
Ambiguity:	Yes	

**1 - 552**	**Reference: 1**	**Rover: 552**

Coordinates:		
Latitude:	64° 45' 28.83428" N	64° 51' 48.48973" N
Longitude:	147° 13' 16.86415" W	147° 38' 34.60217" W
Ellip. Hgt:	550.31586 fts	1131.51713 fts
Solution type:	Phase: all fix	
GNSS type:	GPS	
Frequency:	IonoFree (L3)	
Ambiguity:	Yes	
**1 - 709**	**Reference: 1**	**Rover: 709**
Coordinates:		
Latitude:	64° 45' 28.83428" N	64° 53' 32.88210" N
Longitude:	147° 13' 16.86415" W	147° 25' 03.24335" W
Ellip. Hgt:	550.31586 fts	641.06043 fts
Solution type:	Phase: all fix	
GNSS type:	GPS	
Frequency:	IonoFree (L3)	
Ambiguity:	Yes	
**1 - 706**	**Reference: 1**	**Rover: 706**
Coordinates:		
Latitude:	64° 45' 28.83428" N	64° 45' 02.34369" N
Longitude:	147° 13' 16.86415" W	147° 21' 12.46570" W
Ellip. Hgt:	550.31586 fts	525.62674 fts
Solution type:	Phase: all fix	
GNSS type:	GPS	
Frequency:	L1 and L2	
Ambiguity:	Yes	
**1 - 729**	**Reference: 1**	**Rover: 729**
Coordinates:		
Latitude:	64° 45' 28.83428" N	64° 47' 27.82465" N
Longitude:	147° 13' 16.86415" W	147° 10' 54.06656" W
Ellip. Hgt:	550.31586 fts	562.61036 fts
Solution type:	Phase: all fix	
GNSS type:	GPS	
Frequency:	L1 and L2	
Ambiguity:	Yes	
**1 - 552**	**Reference: 1**	**Rover: 552**
Coordinates:		
Latitude:	64° 45' 28.83428" N	64° 51' 48.48970" N
Longitude:	147° 13' 16.86415" W	147° 38' 34.60209" W
Ellip. Hgt:	550.31586 fts	1131.47192 fts
Solution type:	Phase: all fix	
GNSS type:	GPS	
Frequency:	IonoFree (L3)	
Ambiguity:	Yes	
**1 - 708**	**Reference: 1**	**Rover: 708**

Coordinates:		
Latitude:	64° 45' 28.83428" N	64° 43' 25.09188" N
Longitude:	147° 13' 16.86415" W	147° 16' 22.58853" W
Ellip. Hgt:	550.31586 fts	557.25814 fts
Solution type:	Phase: all fix	
GNSS type:	GPS	
Frequency:	L1 and L2	
Ambiguity:	Yes	

**1 - 729**	**Reference: 1**	**Rover: 729**
Coordinates:		
Latitude:	64° 45' 28.83428" N	64° 47' 27.82469" N
Longitude:	147° 13' 16.86415" W	147° 10' 54.06657" W
Ellip. Hgt:	550.31586 fts	562.60120 fts
Solution type:	Phase: all fix	
GNSS type:	GPS	
Frequency:	L1 and L2	
Ambiguity:	Yes	

**729 - 708**	**Reference: 729**	**Rover: 708**
Coordinates:		
Latitude:	64° 47' 27.82470" N	64° 43' 25.09190" N
Longitude:	147° 10' 54.06648" W	147° 16' 22.58845" W
Ellip. Hgt:	562.60888 fts	557.26890 fts
Solution type:	Phase: all fix	
GNSS type:	GPS	
Frequency:	L1 and L2	
Ambiguity:	Yes	

**551 - 704**	**Reference: 551**	**Rover: 704**
Coordinates:		
Latitude:	64° 48' 50.38170" N	64° 47' 15.07646" N
Longitude:	147° 51' 08.10888" W	147° 58' 13.72322" W
Ellip. Hgt:	462.53516 fts	579.37017 fts
Solution type:	Phase: all fix	
GNSS type:	GPS	
Frequency:	L1 and L2	
Ambiguity:	Yes	

**551 - 703**	**Reference: 551**	**Rover: 703**
Coordinates:		
Latitude:	64° 48' 50.38170" N	64° 50' 58.46939" N
Longitude:	147° 51' 08.10888" W	147° 51' 52.60869" W
Ellip. Hgt:	462.53516 fts	489.34023 fts
Solution type:	Phase: all fix	
GNSS type:	GPS	
Frequency:	L1 and L2	
Ambiguity:	Yes	

**704 - 703**	**Reference: 704**	**Rover: 703**

Coordinates:		
Latitude:	64° 47' 15.07646" N	64° 50' 58.46939" N
Longitude:	147° 58' 13.72322" W	147° 51' 52.60871" W
Ellip. Hgt:	579.37017 fts	489.33754 fts
Solution type:	Phase: all fix	
GNSS type:	GPS	
Frequency:	L1 and L2	
Ambiguity:	Yes	
**1 - 729**	**Reference: 1**	**Rover: 729**
Coordinates:		
Latitude:	64° 45' 28.83428" N	64° 47' 27.82464" N
Longitude:	147° 13' 16.86415" W	147° 10' 54.06655" W
Ellip. Hgt:	550.31586 fts	562.58710 fts
Solution type:	Phase: all fix	
GNSS type:	GPS	
Frequency:	L1 and L2	
Ambiguity:	Yes	
**552 - 729**	**Reference: 552**	**Rover: 729**
Coordinates:		
Latitude:	64° 51' 48.48988" N	64° 47' 27.82474" N
Longitude:	147° 38' 34.60197" W	147° 10' 54.06613" W
Ellip. Hgt:	1131.51230 fts	562.57063 fts
Solution type:	Phase: all fix	
GNSS type:	GPS	
Frequency:	IonoFree (L3)	
Ambiguity:	Yes	
**1 - 708**	**Reference: 1**	**Rover: 708**
Coordinates:		
Latitude:	64° 45' 28.83428" N	64° 43' 25.09182" N
Longitude:	147° 13' 16.86415" W	147° 16' 22.58877" W
Ellip. Hgt:	550.31586 fts	557.28773 fts
Solution type:	Phase: all fix	
GNSS type:	GPS	
Frequency:	L1 and L2	
Ambiguity:	Yes	
**729 - 708**	**Reference: 729**	**Rover: 708**
Coordinates:		
Latitude:	64° 47' 27.82470" N	64° 43' 25.09188" N
Longitude:	147° 10' 54.06648" W	147° 16' 22.58868" W
Ellip. Hgt:	562.60888 fts	557.28698 fts
Solution type:	Phase: all fix	
GNSS type:	GPS	
Frequency:	L1 and L2	
Ambiguity:	Yes	
**706 - 708**	**Reference: 706**	**Rover: 708**

Coordinates:		
Latitude:	64° 45' 02.34371" N	64° 43' 25.09188" N
Longitude:	147° 21' 12.46563" W	147° 16' 22.58866" W
Ellip. Hgt:	525.62739 fts	557.28980 fts
Solution type:	Phase: all fix	
GNSS type:	GPS	
Frequency:	L1 and L2	
Ambiguity:	Yes	

**551 - 705**	**Reference: 551**	**Rover: 705**
Coordinates:		
Latitude:	64° 48' 50.38170" N	64° 51' 21.30424" N
Longitude:	147° 51' 08.10888" W	147° 49' 08.66629" W
Ellip. Hgt:	462.53516 fts	553.86684 fts
Solution type:	Phase: all fix	
GNSS type:	GPS	
Frequency:	L1 and L2	
Ambiguity:	Yes	

**704 - 705**	**Reference: 704**	**Rover: 705**
Coordinates:		
Latitude:	64° 47' 15.07646" N	64° 51' 21.30426" N
Longitude:	147° 58' 13.72322" W	147° 49' 08.66633" W
Ellip. Hgt:	579.37017 fts	553.86533 fts
Solution type:	Phase: all fix	
GNSS type:	GPS	
Frequency:	L1 and L2	
Ambiguity:	Yes	

**703 - 705**	**Reference: 703**	**Rover: 705**
Coordinates:		
Latitude:	64° 50' 58.46939" N	64° 51' 21.30424" N
Longitude:	147° 51' 52.60870" W	147° 49' 08.66627" W
Ellip. Hgt:	489.33931 fts	553.86736 fts
Solution type:	Phase: all fix	
GNSS type:	GPS	
Frequency:	L1 and L2	
Ambiguity:	Yes	

**1 - 734**	**Reference: 1**	**Rover: 734**
Coordinates:		
Latitude:	64° 45' 28.83428" N	64° 41' 26.19579" N
Longitude:	147° 13' 16.86415" W	147° 07' 27.06942" W
Ellip. Hgt:	550.31586 fts	565.16905 fts
Solution type:	Phase: all fix	
GNSS type:	GPS	
Frequency:	L1 and L2	
Ambiguity:	Yes	

**552 - 734**	**Reference: 552**	**Rover: 734**

Coordinates:		
Latitude:	64° 51' 48.48988" N	64° 41' 26.19577" N
Longitude:	147° 38' 34.60197" W	147° 07' 27.06889" W
Ellip. Hgt:	1131.51230 fts	565.13686 fts
Solution type:	Phase: all fix	
GNSS type:	GPS	
Frequency:	IonoFree (L3)	
Ambiguity:	Yes	
**551 - 403**	**Reference: 551**	**Rover: 403**
Coordinates:		
Latitude:	64° 48' 50.38170" N	64° 57' 09.00156" N
Longitude:	147° 51' 08.10888" W	147° 37' 19.95393" W
Ellip. Hgt:	462.53516 fts	770.15256 fts
Solution type:	Phase: all fix	
GNSS type:	GPS	
Frequency:	IonoFree (L3)	
Ambiguity:	Yes	
**1 - 403**	**Reference: 1**	**Rover: 403**
Coordinates:		
Latitude:	64° 45' 28.83428" N	64° 57' 09.00153" N
Longitude:	147° 13' 16.86415" W	147° 37' 19.95384" W
Ellip. Hgt:	550.31586 fts	770.16299 fts
Solution type:	Phase: all fix	
GNSS type:	GPS	
Frequency:	IonoFree (L3)	
Ambiguity:	Yes	
**552 - 704**	**Reference: 552**	**Rover: 704**
Coordinates:		
Latitude:	64° 51' 48.48988" N	64° 47' 15.07656" N
Longitude:	147° 38' 34.60197" W	147° 58' 13.72333" W
Ellip. Hgt:	1131.51230 fts	579.36157 fts
Solution type:	Phase: all fix	
GNSS type:	GPS	
Frequency:	IonoFree (L3)	
Ambiguity:	Yes	
**552 - 703**	**Reference: 552**	**Rover: 703**
Coordinates:		
Latitude:	64° 51' 48.48988" N	64° 50' 58.46939" N
Longitude:	147° 38' 34.60197" W	147° 51' 52.60881" W
Ellip. Hgt:	1131.51230 fts	489.33213 fts
Solution type:	Phase: all fix	
GNSS type:	GPS	
Frequency:	L1 and L2	
Ambiguity:	Yes	
**552 - 705**	**Reference: 552**	**Rover: 705**

Coordinates:		
Latitude:	64° 51' 48.48988" N	64° 51' 21.30427" N
Longitude:	147° 38' 34.60197" W	147° 49' 08.66640" W
Ellip. Hgt:	1131.51230 fts	553.86100 fts
Solution type:	Phase: all fix	
GNSS type:	GPS	
Frequency:	L1 and L2	
Ambiguity:	Yes	

**551 - 741**	**Reference: 551**	**Rover: 741**
Coordinates:		
Latitude:	64° 48' 50.38170" N	64° 56' 55.55673" N
Longitude:	147° 51' 08.10888" W	147° 38' 44.50500" W
Ellip. Hgt:	462.53516 fts	752.28429 fts
Solution type:	Phase: all fix	
GNSS type:	GPS	
Frequency:	IonoFree (L3)	
Ambiguity:	Yes	

**1 - 741**	**Reference: 1**	**Rover: 741**
Coordinates:		
Latitude:	64° 45' 28.83428" N	64° 56' 55.55615" N
Longitude:	147° 13' 16.86415" W	147° 38' 44.49849" W
Ellip. Hgt:	550.31586 fts	752.53993 fts
Solution type:	Phase: all fix	
GNSS type:	GPS	
Frequency:	IonoFree (L3)	
Ambiguity:	Yes	

**403 - 741**	**Reference: 403**	**Rover: 741**
Coordinates:		
Latitude:	64° 57' 09.00154" N	64° 56' 55.55669" N
Longitude:	147° 37' 19.95389" W	147° 38' 44.50522" W
Ellip. Hgt:	770.15778 fts	752.23393 fts
Solution type:	Phase: all fix	
GNSS type:	GPS	
Frequency:	L1 and L2	
Ambiguity:	Yes	

**1 - 708**	**Reference: 1**	**Rover: 708**
Coordinates:		
Latitude:	64° 45' 28.83428" N	64° 43' 25.09178" N
Longitude:	147° 13' 16.86415" W	147° 16' 22.58840" W
Ellip. Hgt:	550.31586 fts	557.26854 fts
Solution type:	Phase: all fix	
GNSS type:	GPS	
Frequency:	L1 and L2	
Ambiguity:	Yes	

**552 - 708**	**Reference: 552**	**Rover: 708**

Coordinates:		
Latitude:	64° 51' 48.48988" N	64° 43' 25.09175" N
Longitude:	147° 38' 34.60197" W	147° 16' 22.58827" W
Ellip. Hgt:	1131.51230 fts	557.29373 fts
Solution type:	Phase: all fix	
GNSS type:	GPS	
Frequency:	IonoFree (L3)	
Ambiguity:	Yes	
**729 - 708**	**Reference: 729**	**Rover: 708**
Coordinates:		
Latitude:	64° 47' 27.82470" N	64° 43' 25.09178" N
Longitude:	147° 10' 54.06648" W	147° 16' 22.58839" W
Ellip. Hgt:	562.60888 fts	557.24780 fts
Solution type:	Phase: all fix	
GNSS type:	GPS	
Frequency:	L1 and L2	
Ambiguity:	Yes	
**729 - 734**	**Reference: 729**	**Rover: 734**
Coordinates:		
Latitude:	64° 47' 27.82470" N	64° 41' 26.19582" N
Longitude:	147° 10' 54.06648" W	147° 07' 27.06941" W
Ellip. Hgt:	562.60888 fts	565.16337 fts
Solution type:	Phase: all fix	
GNSS type:	GPS	
Frequency:	L1 and L2	
Ambiguity:	Yes	
**701 - 704**	**Reference: 701**	**Rover: 704**
Coordinates:		
Latitude:	64° 48' 40.88790" N	64° 47' 15.08911" N
Longitude:	147° 34' 35.30800" W	147° 58' 13.71809" W
Ellip. Hgt:	487.75821 fts	577.68684 fts
Solution type:	Float	
GNSS type:	GPS	
Frequency:	IonoFree (L3)	
Ambiguity:	No	
**551 - 552**	**Reference: 551**	**Rover: 552**
Coordinates:		
Latitude:	64° 48' 50.38170" N	64° 51' 48.48976" N
Longitude:	147° 51' 08.10888" W	147° 38' 34.60168" W
Ellip. Hgt:	462.53516 fts	1131.48819 fts
Solution type:	Phase: all fix	
GNSS type:	GPS	
Frequency:	L1 and L2	
Ambiguity:	Yes	
**701 - 703**	**Reference: 701**	**Rover: 703**

Coordinates:		
Latitude:	64° 48' 40.88790" N	64° 50' 58.46950" N
Longitude:	147° 34' 35.30800" W	147° 51' 52.60536" W
Ellip. Hgt:	487.75821 fts	489.41995 fts
Solution type:	Float	
GNSS type:	GPS	
Frequency:	L1 and L2	
Ambiguity:	No	
**701 - 705**	**Reference: 701**	**Rover: 705**
Coordinates:		
Latitude:	64° 48' 40.88790" N	64° 51' 21.31759" N
Longitude:	147° 34' 35.30800" W	147° 49' 08.65928" W
Ellip. Hgt:	487.75821 fts	552.65191 fts
Solution type:	Phase: all fix	
GNSS type:	GPS	
Frequency:	L1 and L2	
Ambiguity:	Yes	
**702 - 704**	**Reference: 702**	**Rover: 704**
Coordinates:		
Latitude:	64° 47' 49.58460" N	64° 47' 15.07641" N
Longitude:	147° 40' 09.75184" W	147° 58' 13.72339" W
Ellip. Hgt:	490.10450 fts	579.38227 fts
Solution type:	Phase: all fix	
GNSS type:	GPS	
Frequency:	L1 and L2	
Ambiguity:	Yes	
**702 - 703**	**Reference: 702**	**Rover: 703**
Coordinates:		
Latitude:	64° 47' 49.58460" N	64° 50' 58.46936" N
Longitude:	147° 40' 09.75184" W	147° 51' 52.60881" W
Ellip. Hgt:	490.10450 fts	489.34712 fts
Solution type:	Phase: all fix	
GNSS type:	GPS	
Frequency:	L1 and L2	
Ambiguity:	Yes	
**552 - 403**	**Reference: 552**	**Rover: 403**
Coordinates:		
Latitude:	64° 51' 48.48988" N	64° 57' 09.00169" N
Longitude:	147° 38' 34.60197" W	147° 37' 19.95399" W
Ellip. Hgt:	1131.51230 fts	770.22910 fts
Solution type:	Phase: all fix	
GNSS type:	GPS	
Frequency:	L1 and L2	
Ambiguity:	Yes	
**708 - 734**	**Reference: 708**	**Rover: 734**

Coordinates:		
Latitude:	64° 43' 25.09186" N	64° 41' 26.19584" N
Longitude:	147° 16' 22.58856" W	147° 07' 27.06959" W
Ellip. Hgt:	557.27316 fts	565.18824 fts
Solution type:	Phase: all fix	
GNSS type:	GPS	
Frequency:	L1 and L2	
Ambiguity:	Yes	
**702 - 705**	**Reference: 702**	**Rover: 705**
Coordinates:		
Latitude:	64° 47' 49.58460" N	64° 51' 21.30420" N
Longitude:	147° 40' 09.75184" W	147° 49' 08.66641" W
Ellip. Hgt:	490.10450 fts	553.86992 fts
Solution type:	Phase: all fix	
GNSS type:	GPS	
Frequency:	L1 and L2	
Ambiguity:	Yes	
**551 - 708**	**Reference: 551**	**Rover: 708**
Coordinates:		
Latitude:	64° 48' 50.38170" N	64° 43' 25.09160" N
Longitude:	147° 51' 08.10888" W	147° 16' 22.58821" W
Ellip. Hgt:	462.53516 fts	557.25174 fts
Solution type:	Phase: all fix	
GNSS type:	GPS	
Frequency:	IonoFree (L3)	
Ambiguity:	Yes	
**1 - 708**	**Reference: 1**	**Rover: 708**
Coordinates:		
Latitude:	64° 45' 28.83428" N	64° 43' 25.09177" N
Longitude:	147° 13' 16.86415" W	147° 16' 22.58884" W
Ellip. Hgt:	550.31586 fts	557.27441 fts
Solution type:	Phase: all fix	
GNSS type:	GPS	
Frequency:	L1 and L2	
Ambiguity:	Yes	
**702 - 708**	**Reference: 702**	**Rover: 708**
Coordinates:		
Latitude:	64° 47' 49.58460" N	64° 43' 25.09183" N
Longitude:	147° 40' 09.75184" W	147° 16' 22.58768" W
Ellip. Hgt:	490.10450 fts	557.32047 fts
Solution type:	Phase: all fix	
GNSS type:	GPS	
Frequency:	IonoFree (L3)	
Ambiguity:	Yes	
**701 - 708**	**Reference: 701**	**Rover: 708**

Coordinates:		
Latitude:	64° 48' 40.88790" N	64° 43' 25.08555" N
Longitude:	147° 34' 35.30800" W	147° 16' 22.57031" W
Ellip. Hgt:	487.75821 fts	557.32261 fts
Solution type:	Float	
GNSS type:	GPS	
Frequency:	IonoFree (L3)	
Ambiguity:	No	
**552 - 708**	**Reference: 552**	**Rover: 708**
Coordinates:		
Latitude:	64° 51' 48.48988" N	64° 43' 25.09179" N
Longitude:	147° 38' 34.60197" W	147° 16' 22.58796" W
Ellip. Hgt:	1131.51230 fts	557.26595 fts
Solution type:	Phase: all fix	
GNSS type:	GPS	
Frequency:	IonoFree (L3)	
Ambiguity:	Yes	
**709 - 708**	**Reference: 709**	**Rover: 708**
Coordinates:		
Latitude:	64° 53' 32.88242" N	64° 43' 25.09196" N
Longitude:	147° 25' 03.24303" W	147° 16' 22.58796" W
Ellip. Hgt:	641.05994 fts	557.27047 fts
Solution type:	Phase: all fix	
GNSS type:	GPS	
Frequency:	IonoFree (L3)	
Ambiguity:	Yes	
**552 - 741**	**Reference: 552**	**Rover: 741**
Coordinates:		
Latitude:	64° 51' 48.48988" N	64° 56' 55.55636" N
Longitude:	147° 38' 34.60197" W	147° 38' 44.50497" W
Ellip. Hgt:	1131.51230 fts	752.40594 fts
Solution type:	Float	
GNSS type:	GPS	
Frequency:	L1 and L2	
Ambiguity:	No	
**709 - 704**	**Reference: 709**	**Rover: 704**
Coordinates:		
Latitude:	64° 53' 32.88242" N	64° 47' 15.07670" N
Longitude:	147° 25' 03.24303" W	147° 58' 13.72335" W
Ellip. Hgt:	641.05994 fts	579.35104 fts
Solution type:	Phase: all fix	
GNSS type:	GPS	
Frequency:	IonoFree (L3)	
Ambiguity:	Yes	
**709 - 703**	**Reference: 709**	**Rover: 703**

Coordinates:		
Latitude:	64° 53' 32.88242" N	64° 50' 58.46945" N
Longitude:	147° 25' 03.24303" W	147° 51' 52.60879" W
Ellip. Hgt:	641.05994 fts	489.32711 fts
Solution type:	Phase: all fix	
GNSS type:	GPS	
Frequency:	IonoFree (L3)	
Ambiguity:	Yes	
**551 - 707**	**Reference: 551**	**Rover: 707**
Coordinates:		
Latitude:	64° 48' 50.38170" N	64° 44' 00.61830" N
Longitude:	147° 51' 08.10888" W	147° 21' 31.73340" W
Ellip. Hgt:	462.53516 fts	528.53152 fts
Solution type:	Phase: all fix	
GNSS type:	GPS	
Frequency:	IonoFree (L3)	
Ambiguity:	Yes	
**1 - 707**	**Reference: 1**	**Rover: 707**
Coordinates:		
Latitude:	64° 45' 28.83428" N	64° 44' 00.61821" N
Longitude:	147° 13' 16.86415" W	147° 21' 31.73349" W
Ellip. Hgt:	550.31586 fts	528.56233 fts
Solution type:	Phase: all fix	
GNSS type:	GPS	
Frequency:	L1 and L2	
Ambiguity:	Yes	
**702 - 707**	**Reference: 702**	**Rover: 707**
Coordinates:		
Latitude:	64° 47' 49.58460" N	64° 44' 00.61851" N
Longitude:	147° 40' 09.75184" W	147° 21' 31.73285" W
Ellip. Hgt:	490.10450 fts	528.60140 fts
Solution type:	Phase: all fix	
GNSS type:	GPS	
Frequency:	IonoFree (L3)	
Ambiguity:	Yes	
**701 - 707**	**Reference: 701**	**Rover: 707**
Coordinates:		
Latitude:	64° 48' 40.88790" N	64° 44' 00.61870" N
Longitude:	147° 34' 35.30800" W	147° 21' 31.73326" W
Ellip. Hgt:	487.75821 fts	528.54005 fts
Solution type:	Phase: all fix	
GNSS type:	GPS	
Frequency:	L1 and L2	
Ambiguity:	Yes	
**552 - 707**	**Reference: 552**	**Rover: 707**

Coordinates:		
Latitude:	64° 51' 48.48988" N	64° 44' 00.61848" N
Longitude:	147° 38' 34.60197" W	147° 21' 31.73314" W
Ellip. Hgt:	1131.51230 fts	528.56171 fts
Solution type:	Phase: all fix	
GNSS type:	GPS	
Frequency:	IonoFree (L3)	
Ambiguity:	Yes	

**709 - 707**	**Reference: 709**	**Rover: 707**
Coordinates:		
Latitude:	64° 53' 32.88242" N	64° 44' 00.61856" N
Longitude:	147° 25' 03.24303" W	147° 21' 31.73315" W
Ellip. Hgt:	641.05994 fts	528.55153 fts
Solution type:	Phase: all fix	
GNSS type:	GPS	
Frequency:	IonoFree (L3)	
Ambiguity:	Yes	

**709 - 705**	**Reference: 709**	**Rover: 705**
Coordinates:		
Latitude:	64° 53' 32.88242" N	64° 51' 21.30419" N
Longitude:	147° 25' 03.24303" W	147° 49' 08.66643" W
Ellip. Hgt:	641.05994 fts	553.84443 fts
Solution type:	Phase: all fix	
GNSS type:	GPS	
Frequency:	IonoFree (L3)	
Ambiguity:	Yes	

**1 - 704**	**Reference: 1**	**Rover: 704**
Coordinates:		
Latitude:	64° 45' 28.83428" N	64° 47' 15.07646" N
Longitude:	147° 13' 16.86415" W	147° 58' 13.72332" W
Ellip. Hgt:	550.31586 fts	579.35091 fts
Solution type:	Phase: all fix	
GNSS type:	GPS	
Frequency:	IonoFree (L3)	
Ambiguity:	Yes	

**708 - 707**	**Reference: 708**	**Rover: 707**
Coordinates:		
Latitude:	64° 43' 25.09186" N	64° 44' 00.61829" N
Longitude:	147° 16' 22.58856" W	147° 21' 31.73317" W
Ellip. Hgt:	557.27316 fts	528.56522 fts
Solution type:	Phase: all fix	
GNSS type:	GPS	
Frequency:	L1 and L2	
Ambiguity:	Yes	

**551 - 729**	**Reference: 551**	**Rover: 729**

Coordinates:		
Latitude:	64° 48' 50.38170" N	64° 47' 27.82464" N
Longitude:	147° 51' 08.10888" W	147° 10' 54.06624" W
Ellip. Hgt:	462.53516 fts	562.63401 fts
Solution type:	Phase: all fix	
GNSS type:	GPS	
Frequency:	IonoFree (L3)	
Ambiguity:	Yes	
**1 - 729**	**Reference: 1**	**Rover: 729**
Coordinates:		
Latitude:	64° 45' 28.83428" N	64° 47' 27.82472" N
Longitude:	147° 13' 16.86415" W	147° 10' 54.06657" W
Ellip. Hgt:	550.31586 fts	562.62834 fts
Solution type:	Phase: all fix	
GNSS type:	GPS	
Frequency:	L1 and L2	
Ambiguity:	Yes	
**702 - 729**	**Reference: 702**	**Rover: 729**
Coordinates:		
Latitude:	64° 47' 49.58460" N	64° 47' 27.82490" N
Longitude:	147° 40' 09.75184" W	147° 10' 54.06569" W
Ellip. Hgt:	490.10450 fts	562.69720 fts
Solution type:	Phase: all fix	
GNSS type:	GPS	
Frequency:	IonoFree (L3)	
Ambiguity:	Yes	
**701 - 729**	**Reference: 701**	**Rover: 729**
Coordinates:		
Latitude:	64° 48' 40.88790" N	64° 47' 27.82336" N
Longitude:	147° 34' 35.30800" W	147° 10' 54.04786" W
Ellip. Hgt:	487.75821 fts	563.01272 fts
Solution type:	Float	
GNSS type:	GPS	
Frequency:	IonoFree (L3)	
Ambiguity:	No	
**552 - 729**	**Reference: 552**	**Rover: 729**
Coordinates:		
Latitude:	64° 51' 48.48988" N	64° 47' 27.82485" N
Longitude:	147° 38' 34.60197" W	147° 10' 54.06590" W
Ellip. Hgt:	1131.51230 fts	562.65865 fts
Solution type:	Phase: all fix	
GNSS type:	GPS	
Frequency:	IonoFree (L3)	
Ambiguity:	Yes	
**729 - 709**	**Reference: 729**	**Rover: 709**

Coordinates:		
Latitude:	64° 47' 27.82470" N	64° 53' 32.88206" N
Longitude:	147° 10' 54.06648" W	147° 25' 03.24345" W
Ellip. Hgt:	562.60888 fts	641.00915 fts
Solution type:	Phase: all fix	
GNSS type:	GPS	
Frequency:	IonoFree (L3)	
Ambiguity:	Yes	
**729 - 708**	**Reference: 729**	**Rover: 708**
Coordinates:		
Latitude:	64° 47' 27.82470" N	64° 43' 25.09174" N
Longitude:	147° 10' 54.06648" W	147° 16' 22.58882" W
Ellip. Hgt:	562.60888 fts	557.25263 fts
Solution type:	Phase: all fix	
GNSS type:	GPS	
Frequency:	L1 and L2	
Ambiguity:	Yes	
**1 - 703**	**Reference: 1**	**Rover: 703**
Coordinates:		
Latitude:	64° 45' 28.83428" N	64° 50' 58.46920" N
Longitude:	147° 13' 16.86415" W	147° 51' 52.60881" W
Ellip. Hgt:	550.31586 fts	489.33108 fts
Solution type:	Phase: all fix	
GNSS type:	GPS	
Frequency:	IonoFree (L3)	
Ambiguity:	Yes	
**551 - 734**	**Reference: 551**	**Rover: 734**
Coordinates:		
Latitude:	64° 48' 50.38170" N	64° 41' 26.19560" N
Longitude:	147° 51' 08.10888" W	147° 07' 27.06890" W
Ellip. Hgt:	462.53516 fts	565.08165 fts
Solution type:	Phase: all fix	
GNSS type:	GPS	
Frequency:	IonoFree (L3)	
Ambiguity:	Yes	
**1 - 705**	**Reference: 1**	**Rover: 705**
Coordinates:		
Latitude:	64° 45' 28.83428" N	64° 51' 21.30388" N
Longitude:	147° 13' 16.86415" W	147° 49' 08.66642" W
Ellip. Hgt:	550.31586 fts	553.84706 fts
Solution type:	Phase: all fix	
GNSS type:	GPS	
Frequency:	IonoFree (L3)	
Ambiguity:	Yes	
**729 - 707**	**Reference: 729**	**Rover: 707**

Coordinates:		
Latitude:	64° 47' 27.82470" N	64° 44' 00.61821" N
Longitude:	147° 10' 54.06648" W	147° 21' 31.73346" W
Ellip. Hgt:	562.60888 fts	528.54123 fts
Solution type:	Phase: all fix	
GNSS type:	GPS	
Frequency:	L1 and L2	
Ambiguity:	Yes	

**734 - 403**	**Reference: 734**	**Rover: 403**
Coordinates:		
Latitude:	64° 41' 26.19580" N	64° 57' 09.00165" N
Longitude:	147° 07' 27.06939" W	147° 37' 19.95436" W
Ellip. Hgt:	565.16518 fts	770.22126 fts
Solution type:	Phase: all fix	
GNSS type:	GPS	
Frequency:	IonoFree (L3)	
Ambiguity:	Yes	

**551 - 729**	**Reference: 551**	**Rover: 729**
Coordinates:		
Latitude:	64° 48' 50.38170" N	64° 47' 27.82453" N
Longitude:	147° 51' 08.10888" W	147° 10' 54.06614" W
Ellip. Hgt:	462.53516 fts	562.53693 fts
Solution type:	Phase: all fix	
GNSS type:	GPS	
Frequency:	IonoFree (L3)	
Ambiguity:	Yes	

**734 - 741**	**Reference: 734**	**Rover: 741**
Coordinates:		
Latitude:	64° 41' 26.19580" N	64° 56' 55.55343" N
Longitude:	147° 07' 27.06939" W	147° 38' 44.50489" W
Ellip. Hgt:	565.16518 fts	752.65338 fts
Solution type:	Phase: all fix	
GNSS type:	GPS	
Frequency:	IonoFree (L3)	
Ambiguity:	Yes	

**551 - 402**	**Reference: 551**	**Rover: 402**
Coordinates:		
Latitude:	64° 48' 50.38170" N	64° 43' 18.04960" N
Longitude:	147° 51' 08.10888" W	147° 17' 04.41259" W
Ellip. Hgt:	462.53516 fts	546.13874 fts
Solution type:	Phase: all fix	
GNSS type:	GPS	
Frequency:	IonoFree (L3)	
Ambiguity:	Yes	

**729 - 403**	**Reference: 729**	**Rover: 403**

Coordinates:		
Latitude:	64° 47' 27.82470" N	64° 57' 09.00166" N
Longitude:	147° 10' 54.06648" W	147° 37' 19.95414" W
Ellip. Hgt:	562.60888 fts	770.23983 fts
Solution type:	Phase: all fix	
GNSS type:	GPS	
Frequency:	IonoFree (L3)	
Ambiguity:	Yes	
**551 - 708**	**Reference: 551**	**Rover: 708**
Coordinates:		
Latitude:	64° 48' 50.38170" N	64° 43' 25.09156" N
Longitude:	147° 51' 08.10888" W	147° 16' 22.58826" W
Ellip. Hgt:	462.53516 fts	557.23373 fts
Solution type:	Phase: all fix	
GNSS type:	GPS	
Frequency:	IonoFree (L3)	
Ambiguity:	Yes	
**729 - 741**	**Reference: 729**	**Rover: 741**
Coordinates:		
Latitude:	64° 47' 27.82470" N	64° 56' 55.55678" N
Longitude:	147° 10' 54.06648" W	147° 38' 44.50487" W
Ellip. Hgt:	562.60888 fts	752.38590 fts
Solution type:	Phase: all fix	
GNSS type:	GPS	
Frequency:	IonoFree (L3)	
Ambiguity:	Yes	
**403 - 402**	**Reference: 403**	**Rover: 402**
Coordinates:		
Latitude:	64° 57' 09.00154" N	64° 43' 18.04972" N
Longitude:	147° 37' 19.95389" W	147° 17' 04.41242" W
Ellip. Hgt:	770.15778 fts	546.16807 fts
Solution type:	Phase: all fix	
GNSS type:	GPS	
Frequency:	IonoFree (L3)	
Ambiguity:	Yes	
**741 - 402**	**Reference: 741**	**Rover: 402**
Coordinates:		
Latitude:	64° 56' 55.55670" N	64° 43' 18.04891" N
Longitude:	147° 38' 44.50519" W	147° 17' 04.40565" W
Ellip. Hgt:	752.24443 fts	546.31489 fts
Solution type:	Phase: all fix	
GNSS type:	GPS	
Frequency:	IonoFree (L3)	
Ambiguity:	Yes	
**708 - 403**	**Reference: 708**	**Rover: 403**

Coordinates:		
Latitude:	64° 43' 25.09186" N	64° 57' 09.00173" N
Longitude:	147° 16' 22.58856" W	147° 37' 19.95429" W
Ellip. Hgt:	557.27316 fts	770.19958 fts
Solution type:	Phase: all fix	
GNSS type:	GPS	
Frequency:	IonoFree (L3)	
Ambiguity:	Yes	
**708 - 741**	**Reference: 708**	**Rover: 741**
Coordinates:		
Latitude:	64° 43' 25.09186" N	64° 56' 55.55376" N
Longitude:	147° 16' 22.58856" W	147° 38' 44.50409" W
Ellip. Hgt:	557.27316 fts	752.59787 fts
Solution type:	Phase: all fix	
GNSS type:	GPS	
Frequency:	IonoFree (L3)	
Ambiguity:	Yes	
**1 - 706**	**Reference: 1**	**Rover: 706**
Coordinates:		
Latitude:	64° 45' 28.83428" N	64° 45' 02.34386" N
Longitude:	147° 13' 16.86415" W	147° 21' 12.46591" W
Ellip. Hgt:	550.31586 fts	525.63054 fts
Solution type:	Phase: all fix	
GNSS type:	GPS	
Frequency:	L1 and L2	
Ambiguity:	Yes	
**552 - 706**	**Reference: 552**	**Rover: 706**
Coordinates:		
Latitude:	64° 51' 48.48988" N	64° 45' 02.34351" N
Longitude:	147° 38' 34.60197" W	147° 21' 12.46562" W
Ellip. Hgt:	1131.51230 fts	525.62907 fts
Solution type:	Phase: all fix	
GNSS type:	GPS	
Frequency:	IonoFree (L3)	
Ambiguity:	Yes	
**729 - 706**	**Reference: 729**	**Rover: 706**
Coordinates:		
Latitude:	64° 47' 27.82470" N	64° 45' 02.34395" N
Longitude:	147° 10' 54.06648" W	147° 21' 12.46570" W
Ellip. Hgt:	562.60888 fts	525.63727 fts
Solution type:	Phase: all fix	
GNSS type:	GPS	
Frequency:	L1 and L2	
Ambiguity:	Yes	
**706 - 734**	**Reference: 706**	**Rover: 734**

Coordinates:		
Latitude:	64° 45' 02.34371" N	64° 41' 26.19576" N
Longitude:	147° 21' 12.46563" W	147° 07' 27.06867" W
Ellip. Hgt:	525.62739 fts	565.17203 fts
Solution type:	Phase: all fix	
GNSS type:	GPS	
Frequency:	L1 and L2	
Ambiguity:	Yes	
**706 - 708**	**Reference: 706**	**Rover: 708**
Coordinates:		
Latitude:	64° 45' 02.34371" N	64° 43' 25.09170" N
Longitude:	147° 21' 12.46563" W	147° 16' 22.58808" W
Ellip. Hgt:	525.62739 fts	557.25095 fts
Solution type:	Phase: all fix	
GNSS type:	GPS	
Frequency:	L1 and L2	
Ambiguity:	Yes	
**706 - 402**	**Reference: 706**	**Rover: 402**
Coordinates:		
Latitude:	64° 45' 02.34371" N	64° 43' 18.04966" N
Longitude:	147° 21' 12.46563" W	147° 17' 04.41410" W
Ellip. Hgt:	525.62739 fts	546.26777 fts
Solution type:	Phase: all fix	
GNSS type:	GPS	
Frequency:	L1 and L2	
Ambiguity:	Yes	
**552 - 729**	**Reference: 552**	**Rover: 729**
Coordinates:		
Latitude:	64° 51' 48.48988" N	64° 47' 27.82614" N
Longitude:	147° 38' 34.60197" W	147° 10' 54.06843" W
Ellip. Hgt:	1131.51230 fts	562.40235 fts
Solution type:	Float	
GNSS type:	GPS	
Frequency:	IonoFree (L3)	
Ambiguity:	No	
**1 - 706**	**Reference: 1**	**Rover: 706**
Coordinates:		
Latitude:	64° 45' 28.83428" N	64° 45' 02.34390" N
Longitude:	147° 13' 16.86415" W	147° 21' 12.46571" W
Ellip. Hgt:	550.31586 fts	525.67559 fts
Solution type:	Phase: all fix	
GNSS type:	GPS	
Frequency:	L1 and L2	
Ambiguity:	Yes	
**729 - 708**	**Reference: 729**	**Rover: 708**

Coordinates:		
Latitude:	64° 47' 27.82470" N	64° 43' 25.09182" N
Longitude:	147° 10' 54.06648" W	147° 16' 22.58808" W
Ellip. Hgt:	562.60888 fts	557.23261 fts
Solution type:	Phase: all fix	
GNSS type:	GPS	
Frequency:	L1 and L2	
Ambiguity:	Yes	
**1 - 552**	**Reference: 1**	**Rover: 552**
Coordinates:		
Latitude:	64° 45' 28.83428" N	64° 51' 48.48737" N
Longitude:	147° 13' 16.86415" W	147° 38' 34.58958" W
Ellip. Hgt:	550.31586 fts	1131.65682 fts
Solution type:	Float	
GNSS type:	GPS	
Frequency:	IonoFree (L3)	
Ambiguity:	No	
**1 - 729**	**Reference: 1**	**Rover: 729**
Coordinates:		
Latitude:	64° 45' 28.83428" N	64° 47' 27.82455" N
Longitude:	147° 13' 16.86415" W	147° 10' 54.06729" W
Ellip. Hgt:	550.31586 fts	562.50931 fts
Solution type:	Phase: all fix	
GNSS type:	GPS	
Frequency:	L1 and L2	
Ambiguity:	Yes	
**1 - 706**	**Reference: 1**	**Rover: 706**
Coordinates:		
Latitude:	64° 45' 28.83428" N	64° 45' 02.34381" N
Longitude:	147° 13' 16.86415" W	147° 21' 12.46560" W
Ellip. Hgt:	550.31586 fts	525.60800 fts
Solution type:	Phase: all fix	
GNSS type:	GPS	
Frequency:	L1 and L2	
Ambiguity:	Yes	
**729 - 706**	**Reference: 729**	**Rover: 706**
Coordinates:		
Latitude:	64° 47' 27.82470" N	64° 45' 02.34497" N
Longitude:	147° 10' 54.06648" W	147° 21' 12.46741" W
Ellip. Hgt:	562.60888 fts	525.90863 fts
Solution type:	Phase: all fix	
GNSS type:	GPS	
Frequency:	L1 and L2	
Ambiguity:	Yes	

Kinematic Overview

**551 - 401**	**Reference: 551**	**Rover: 401**
Receiver type / S/N:	SR530 / 34670	GX1230GG / 458014
Antenna type / S/N:	AT502 Tripod / -	AX1202 GG Pole / -
Antenna height:	4.46521 fts	

Reference coordinates:
- Latitude: 64° 48' 50.38170" N
- Longitude: 147° 51' 08.10888" W
- Ellip. Hgt: 462.53516 fts

Manually occupied points: 2

Time span: 04/21/2010 07:42:00 - 04/21/2010 15:44:40
Duration: 8h 02' 40"

DOPs (min-max): GDOP: 1.9 - 13.1
PDOP: 1.7 - 10.9 HDOP: 0.8 - 3.2 VDOP: 1.4 - 10.4

Manually occupied points
401
Coordinates:
- Latitude: 64° 49' 21.41283" N
- Longitude: 147° 49' 35.18769" W
- Ellip. Hgt: 465.26439 fts

Antenna height: 5.20668 fts

Solution type: Phase: all fix
Frequency: L1 and L2
Ambiguity: Yes
Time span: 04/21/2010 07:42:00 - 04/21/2010 12:12:15
Duration: 4h 30' 15"

401
Coordinates:
- Latitude: 64° 49' 21.41282" N
- Longitude: 147° 49' 35.18766" W
- Ellip. Hgt: 465.25534 fts

Antenna height: 5.20668 fts

Solution type: Phase: all fix
Frequency: L1 and L2
Ambiguity: Yes
Time span: 04/21/2010 13:57:15 - 04/21/2010 15:44:40
Duration: 1h 47' 25"

**704 - 401**	**Reference: 704**	**Rover: 401**
Receiver type / S/N:	SR530 / 39986	GX1230GG / 458014
Antenna type / S/N:	AT502 Tripod / -	AX1202 GG Pole / -
Antenna height:	3.70078 fts	

Reference coordinates:
- Latitude: 64° 47' 15.07646" N
- Longitude: 147° 58' 13.72322" W
- Ellip. Hgt: 579.37017 fts

Manually occupied points:	2		
Time span:	04/21/2010 07:29:20 - 04/21/2010 15:31:00		
Duration:	8h 01' 40"		
DOPs (min-max):	GDOP: 1.9 - 37.1		
	PDOP: 1.7 - 29.5	HDOP: 0.8 - 14.9	VDOP: 1.5 - 25.4

Manually occupied points

401

Coordinates:	
Latitude:	64° 49' 21.41278" N
Longitude:	147° 49' 35.18780" W
Ellip. Hgt:	465.27000 fts
Antenna height:	5.20668 fts
Solution type:	Phase: all fix
Frequency:	L1 and L2
Ambiguity:	Yes
Time span:	04/21/2010 07:29:20 - 04/21/2010 12:12:15
Duration:	4h 42' 55"

401

Coordinates:	
Latitude:	64° 49' 21.41277" N
Longitude:	147° 49' 35.18771" W
Ellip. Hgt:	465.25475 fts
Antenna height:	5.20668 fts
Solution type:	Phase: all fix
Frequency:	L1 and L2
Ambiguity:	Yes
Time span:	04/21/2010 13:57:15 - 04/21/2010 15:31:00
Duration:	1h 33' 45"

**703 - 401**	**Reference: 703**	**Rover: 401**
Receiver type / S/N:	SR530 / 132169	GX1230GG / 458014
Antenna type / S/N:	AT502 Tripod / -	AX1202 GG Pole / -
Antenna height:	6.20734 fts	

Reference coordinates:			
Latitude:	64° 50' 58.46939" N		
Longitude:	147° 51' 52.60870" W		
Ellip. Hgt:	489.33931 fts		
Manually occupied points:	2		
Time span:	04/21/2010 07:59:10 - 04/21/2010 15:19:35		
Duration:	7h 20' 25"		
DOPs (min-max):	GDOP: 1.9 - 7.1		
	PDOP: 1.7 - 5.9	HDOP: 0.8 - 2.5	VDOP: 1.4 - 5.3

Manually occupied points
401

Coordinates:	
Latitude:	64° 49' 21.41283" N
Longitude:	147° 49' 35.18769" W
Ellip. Hgt:	465.25826 fts
Antenna height:	5.20668 fts
Solution type:	Phase: all fix
Frequency:	L1 and L2
Ambiguity:	Yes
Time span:	04/21/2010 07:59:10 - 04/21/2010 12:12:15
Duration:	4h 13' 05"

401

Coordinates:	
Latitude:	64° 49' 21.41282" N
Longitude:	147° 49' 35.18767" W
Ellip. Hgt:	465.25629 fts
Antenna height:	5.20668 fts
Solution type:	Phase: all fix
Frequency:	L1 and L2
Ambiguity:	Yes
Time span:	04/21/2010 13:57:15 - 04/21/2010 15:19:35
Duration:	1h 22' 20"

**705 - 401**	**Reference: 705**	**Rover: 401**	
Receiver type / S/N:	SR530 / 32671	GX1230GG / 458014	
Antenna type / S/N:	AT502 Tripod / -	AX1202 GG Pole / -	
Antenna height:	2.75262 fts		
Reference coordinates:			
Latitude:	64° 51' 21.30425" N		
Longitude:	147° 49' 08.66629" W		
Ellip. Hgt:	553.86667 fts		
Manually occupied points:	2		
Time span:	04/21/2010 08:16:45 - 04/21/2010 15:10:05		
Duration:	6h 53' 20"		
DOPs (min-max):	GDOP: 1.9 - 6.5		
	PDOP: 1.7 - 5.3	HDOP: 0.7 - 2.4	VDOP: 1.4 - 4.8

Manually occupied points
401

Coordinates:	
Latitude:	64° 49' 21.41286" N
Longitude:	147° 49' 35.18772" W
Ellip. Hgt:	465.25064 fts
Antenna height:	5.20668 fts

Solution type:	Phase: all fix
Frequency:	L1 and L2
Ambiguity:	Yes
Time span:	04/21/2010 08:16:45 - 04/21/2010 12:12:15
Duration:	3h 55' 30"

401

Coordinates:	
Latitude:	64° 49' 21.41281" N
Longitude:	147° 49' 35.18765" W
Ellip. Hgt:	465.24694 fts
Antenna height:	5.20668 fts
Solution type:	Phase: all fix
Frequency:	L1 and L2
Ambiguity:	Yes
Time span:	04/21/2010 13:57:15 - 04/21/2010 15:10:05
Duration:	1h 12' 50"

**552 - 401**	**Reference: 552**	**Rover: 401**	
Receiver type / S/N:	SR530 / 132135	GX1230GG / 458014	
Antenna type / S/N:	AT502 Tripod / -	AX1202 GG Pole / -	
Antenna height:	4.06823 fts		
Reference coordinates:			
Latitude:	64° 51' 48.48988" N		
Longitude:	147° 38' 34.60197" W		
Ellip. Hgt:	1131.51230 fts		
Manually occupied points:	2		
Time span:	04/21/2010 09:10:15 - 04/21/2010 15:44:40		
Duration:	6h 34' 25"		
DOPs (min-max):	GDOP: 1.9 - 277.9		
	PDOP: 1.7 - 265.1	HDOP: 0.8 - 229.8	VDOP: 1.4 - 132.2

Manually occupied points
401

Coordinates:	
Latitude:	64° 49' 21.41280" N
Longitude:	147° 49' 35.18786" W
Ellip. Hgt:	465.23903 fts
Antenna height:	5.20668 fts
Solution type:	Phase: all fix
Frequency:	L1 and L2
Ambiguity:	Yes
Time span:	04/21/2010 09:10:15 - 04/21/2010 12:12:15
Duration:	3h 02' 00"

401

Coordinates:	
Latitude:	64° 49' 21.41284" N

Longitude:	147° 49' 35.18780" W	
Ellip. Hgt:	465.24576 fts	
Antenna height:	5.20668 fts	
Solution type:	Phase: all fix	
Frequency:	L1 and L2	
Ambiguity:	Yes	
Time span:	04/21/2010 13:57:15 - 04/21/2010 15:44:40	
Duration:	1h 47' 25"	
**701 - 401**	**Reference: 701**	**Rover: 401**
Receiver type / S/N:	GX1230GG / 458092	GX1230GG / 458014
Antenna type / S/N:	AX1202 GG Tripod / -	AX1202 GG Pole / -
Antenna height:	3.97309 fts	

Reference coordinates:

Latitude:	64° 48' 40.88790" N
Longitude:	147° 34' 35.30800" W
Ellip. Hgt:	487.75821 fts
Manually occupied points:	2
Time span:	04/21/2010 09:42:50 - 04/21/2010 15:44:40
Duration:	6h 01' 50"

DOPs (min-max):	GDOP: 1.8 - 83.1		
	PDOP: 1.7 - 67.7	HDOP: 0.8 - 25.9	VDOP: 0.0 - 62.6

Manually occupied points

401

Coordinates:

Latitude:	64° 49' 21.41338" N
Longitude:	147° 49' 35.18739" W
Ellip. Hgt:	465.37522 fts
Antenna height:	5.20668 fts
Solution type:	Float
Frequency:	L1 and L2
Ambiguity:	No
Time span:	04/21/2010 09:42:50 - 04/21/2010 12:12:15
Duration:	2h 29' 25"

401

Coordinates:

Latitude:	64° 49' 21.41297" N
Longitude:	147° 49' 35.18653" W
Ellip. Hgt:	465.18519 fts
Antenna height:	5.20668 fts
Solution type:	Float
Frequency:	L1 and L2
Ambiguity:	No
Time span:	04/21/2010 13:57:15 - 04/21/2010 15:44:40

Duration:	1h 47' 25"	
**1 - 402**	**Reference: 1**	**Rover: 402**
Receiver type / S/N:	SR530 / 39982	GX1230GG / 458014
Antenna type / S/N:	AT502 Tripod / -	AX1202 GG Pole / -
Antenna height:	1.56168 fts	
Reference coordinates:		
Latitude:	64° 45' 28.83428" N	
Longitude:	147° 13' 16.86415" W	
Ellip. Hgt:	550.31586 fts	
Manually occupied points:	2	
Time span:	04/22/2010 12:58:50 - 04/22/2010 18:46:05	
Duration:	5h 47' 15"	
DOPs (min-max):	GDOP: 1.7 - 6.0	
	PDOP: 1.5 - 5.1 HDOP: 0.7 - 1.7	VDOP: 1.3 - 4.9

Manually occupied points
402

Coordinates:	
Latitude:	64° 43' 18.04975" N
Longitude:	147° 17' 04.41269" W
Ellip. Hgt:	546.29189 fts
Antenna height:	6.56167 fts
Solution type:	Phase: all fix
Frequency:	L1 and L2
Ambiguity:	Yes
Time span:	04/22/2010 12:58:50 - 04/22/2010 14:23:00
Duration:	1h 24' 10"

402

Coordinates:	
Latitude:	64° 43' 18.04977" N
Longitude:	147° 17' 04.41375" W
Ellip. Hgt:	546.29815 fts
Antenna height:	6.56167 fts
Solution type:	Phase: all fix
Frequency:	L1 and L2
Ambiguity:	Yes
Time span:	04/22/2010 16:57:35 - 04/22/2010 18:46:05
Duration:	1h 48' 30"

**552 - 402**	**Reference: 552**	**Rover: 402**
Receiver type / S/N:	SR530 / 132135	GX1230GG / 458014
Antenna type / S/N:	AT502 Tripod / -	AX1202 GG Pole / -
Antenna height:	4.06823 fts	
Reference coordinates:		
Latitude:	64° 51' 48.48988" N	

Longitude:	147° 38' 34.60197" W
Ellip. Hgt:	1131.51230 fts

Manually occupied points: 2

Time span: 04/22/2010 12:58:50 - 04/22/2010 18:46:05
Duration: 5h 47' 15"

DOPs (min-max): GDOP: 2.2 - 10.7
PDOP: 1.9 - 8.2 HDOP: 0.8 - 6.3 VDOP: 1.6 - 6.8

Manually occupied points
402
Coordinates:
Latitude: 64° 43' 18.04965" N
Longitude: 147° 17' 04.41271" W
Ellip. Hgt: 546.17210 fts

Antenna height: 6.56167 fts

Solution type: Phase: all fix
Frequency: IonoFree (L3)
Ambiguity: Yes
Time span: 04/22/2010 12:58:50 - 04/22/2010 14:23:00
Duration: 1h 24' 10"

402
Coordinates:
Latitude: 64° 43' 18.04985" N
Longitude: 147° 17' 04.41300" W
Ellip. Hgt: 546.22883 fts

Antenna height: 6.56167 fts

Solution type: Phase: all fix
Frequency: IonoFree (L3)
Ambiguity: Yes
Time span: 04/22/2010 16:57:35 - 04/22/2010 18:46:05
Duration: 1h 48' 30"

**729 - 402**	**Reference: 729**	**Rover: 402**
Receiver type / S/N:	GX1230GG / 458087	GX1230GG / 458014
Antenna type / S/N:	AX1202 GG Tripod / -	AX1202 GG Pole / -
Antenna height:	4.61613 fts	

Reference coordinates:
Latitude: 64° 47' 27.82470" N
Longitude: 147° 10' 54.06648" W
Ellip. Hgt: 562.60888 fts

Manually occupied points: 2

Time span: 04/22/2010 12:58:50 - 04/22/2010 18:46:05
Duration: 5h 47' 15"

DOPs (min-max): GDOP: 1.7 - 6.0

	PDOP: 1.5 - 5.1	HDOP: 0.7 - 2.3	VDOP: 1.3 - 4.9

Manually occupied points
402
Coordinates:
Latitude: 64° 43' 18.04981" N
Longitude: 147° 17' 04.41269" W
Ellip. Hgt: 546.28198 fts

Antenna height: 6.56167 fts

Solution type: Phase: all fix
Frequency: L1 and L2
Ambiguity: Yes
Time span: 04/22/2010 12:58:50 - 04/22/2010 14:23:00
Duration: 1h 24' 10"

402
Coordinates:
Latitude: 64° 43' 18.04985" N
Longitude: 147° 17' 04.41364" W
Ellip. Hgt: 546.27036 fts

Antenna height: 6.56167 fts

Solution type: Phase: all fix
Frequency: L1 and L2
Ambiguity: Yes
Time span: 04/22/2010 16:57:35 - 04/22/2010 18:46:05
Duration: 1h 48' 30"

**702 - 401**	**Reference: 702**	**Rover: 401**
Receiver type / S/N:	GX1230GG / 458087	GX1230GG / 458014
Antenna type / S/N:	AX1202 GG Tripod / -	AX1202 GG Pole / -
Antenna height:	4.51443 fts	

Reference coordinates:
Latitude: 64° 47' 49.58460" N
Longitude: 147° 40' 09.75184" W
Ellip. Hgt: 490.10450 fts

Manually occupied points: 2

Time span: 04/21/2010 10:07:00 - 04/21/2010 15:44:40
Duration: 5h 37' 40"

DOPs (min-max):	GDOP: 1.7 - 6.5		
	PDOP: 1.5 - 5.3	HDOP: 0.8 - 2.4	VDOP: 1.3 - 4.8

Manually occupied points
401
Coordinates:
Latitude: 64° 49' 21.41285" N
Longitude: 147° 49' 35.18773" W
Ellip. Hgt: 465.26698 fts

Antenna height:	5.20668 fts	
Solution type:	Phase: all fix	
Frequency:	L1 and L2	
Ambiguity:	Yes	
Time span:	04/21/2010 10:07:00 - 04/21/2010 12:12:15	
Duration:	2h 05' 15"	
**401**		
Coordinates:		
Latitude:	64° 49' 21.41287" N	
Longitude:	147° 49' 35.18773" W	
Ellip. Hgt:	465.26203 fts	
Antenna height:	5.20668 fts	
Solution type:	Phase: all fix	
Frequency:	L1 and L2	
Ambiguity:	Yes	
Time span:	04/21/2010 13:57:15 - 04/21/2010 15:44:40	
Duration:	1h 47' 25"	

**708 - 402**	**Reference: 708**	**Rover: 402**	
Receiver type / S/N:	SR530 / 32671	GX1230GG / 458014	
Antenna type / S/N:	AT502 Tripod / -	AX1202 GG Pole / -	
Antenna height:	4.22899 fts		
Reference coordinates:			
Latitude:	64° 43' 25.09186" N		
Longitude:	147° 16' 22.58856" W		
Ellip. Hgt:	557.27316 fts		
Manually occupied points:	2		
Time span:	04/22/2010 13:09:25 - 04/22/2010 18:46:05		
Duration:	5h 36' 40"		
DOPs (min-max):	GDOP: 1.7 - 6.0		
	PDOP: 1.5 - 5.1	HDOP: 0.7 - 1.7	VDOP: 1.3 - 4.9

Manually occupied points
402

Coordinates:	
Latitude:	64° 43' 18.04988" N
Longitude:	147° 17' 04.41284" W
Ellip. Hgt:	546.33592 fts
Antenna height:	6.56167 fts
Solution type:	Phase: all fix
Frequency:	L1 and L2
Ambiguity:	Yes
Time span:	04/22/2010 13:09:25 - 04/22/2010 14:23:00
Duration:	1h 13' 35"

402

Coordinates:

Latitude:	64° 43' 18.04985" N	
Longitude:	147° 17' 04.41389" W	
Ellip. Hgt:	546.32732 fts	
Antenna height:	6.56167 fts	
Solution type:	Phase: all fix	
Frequency:	L1 and L2	
Ambiguity:	Yes	
Time span:	04/22/2010 16:57:35 - 04/22/2010 18:46:05	
Duration:	1h 48' 30"	

**734 - 402**	**Reference: 734**	**Rover: 402**	
Receiver type / S/N:	SR530 / 39986	GX1230GG / 458014	
Antenna type / S/N:	AT502 Tripod / -	AX1202 GG Pole / -	
Antenna height:	4.54067 fts		
Reference coordinates:			
Latitude:	64° 41' 26.19580" N		
Longitude:	147° 07' 27.06939" W		
Ellip. Hgt:	565.16518 fts		
Manually occupied points:	2		
Time span:	04/22/2010 12:58:50 - 04/22/2010 18:17:40		
Duration:	5h 18' 50"		
DOPs (min-max):	GDOP: 2.1 - 6.4		
	PDOP: 1.9 - 5.4	HDOP: 1.0 - 1.7	VDOP: 1.6 - 5.2

Manually occupied points

402

Coordinates:

Latitude:	64° 43' 18.04972" N
Longitude:	147° 17' 04.41274" W
Ellip. Hgt:	546.28368 fts
Antenna height:	6.56167 fts
Solution type:	Phase: all fix
Frequency:	L1 and L2
Ambiguity:	Yes
Time span:	04/22/2010 12:58:50 - 04/22/2010 14:23:00
Duration:	1h 24' 10"

402

Coordinates:

Latitude:	64° 43' 18.04976" N
Longitude:	147° 17' 04.41370" W
Ellip. Hgt:	546.29743 fts
Antenna height:	6.56167 fts
Solution type:	Phase: all fix
Frequency:	L1 and L2

Ambiguity: Yes
Time span: 04/22/2010 16:57:35 - 04/22/2010 18:17:40
Duration: 1h 20' 05"

**709 - 401**	**Reference: 709**	**Rover: 401**
Receiver type / S/N:	SR530 / 34753	GX1230GG / 458014
Antenna type / S/N:	AT502 Tripod / -	AX1202 GG Pole / -
Antenna height:	4.88844 fts	

Reference coordinates:
Latitude: 64° 53' 32.88242" N
Longitude: 147° 25' 03.24303" W
Ellip. Hgt: 641.05994 fts

Manually occupied points: 2

Time span: 04/21/2010 10:45:50 - 04/21/2010 15:44:40
Duration: 4h 58' 50"

DOPs (min-max): GDOP: 1.9 - 7.1
PDOP: 1.7 - 5.8 HDOP: 0.8 - 2.5 VDOP: 1.4 - 5.3

Manually occupied points
401
Coordinates:
Latitude: 64° 49' 21.41286" N
Longitude: 147° 49' 35.18788" W
Ellip. Hgt: 465.18778 fts

Antenna height: 5.20668 fts

Solution type: Phase: all fix
Frequency: IonoFree (L3)
Ambiguity: Yes
Time span: 04/21/2010 10:45:50 - 04/21/2010 12:12:15
Duration: 1h 26' 25"

401
Coordinates:
Latitude: 64° 49' 21.41309" N
Longitude: 147° 49' 35.18766" W
Ellip. Hgt: 465.22578 fts

Antenna height: 5.20668 fts

Solution type: Phase: all fix
Frequency: IonoFree (L3)
Ambiguity: Yes
Time span: 04/21/2010 13:57:15 - 04/21/2010 15:44:40
Duration: 1h 47' 25"

**1 - 401**	**Reference: 1**	**Rover: 401**
Receiver type / S/N:	SR530 / 39982	GX1230GG / 458014
Antenna type / S/N:	AT502 Tripod / -	AX1202 GG Pole / -
Antenna height:	1.56168 fts	

Reference coordinates:			
Latitude:	64° 45' 28.83428" N		
Longitude:	147° 13' 16.86415" W		
Ellip. Hgt:	550.31586 fts		
Manually occupied points:	2		
Time span:	04/21/2010 11:22:25 - 04/21/2010 15:44:40		
Duration:	4h 22' 15"		
DOPs (min-max):	GDOP: 1.7 - 4.4		
	PDOP: 1.5 - 3.8	HDOP: 0.8 - 2.3	VDOP: 1.3 - 3.5

Manually occupied points
401

Coordinates:	
Latitude:	64° 49' 21.41264" N
Longitude:	147° 49' 35.18722" W
Ellip. Hgt:	465.23024 fts
Antenna height:	5.20668 fts
Solution type:	Phase: all fix
Frequency:	IonoFree (L3)
Ambiguity:	Yes
Time span:	04/21/2010 11:22:25 - 04/21/2010 12:12:15
Duration:	49' 50"

401

Coordinates:	
Latitude:	64° 49' 21.41278" N
Longitude:	147° 49' 35.18750" W
Ellip. Hgt:	465.22394 fts
Antenna height:	5.20668 fts
Solution type:	Phase: all fix
Frequency:	IonoFree (L3)
Ambiguity:	Yes
Time span:	04/21/2010 13:57:15 - 04/21/2010 15:44:40
Duration:	1h 47' 25"

Network Adjustment

www.MOVE3.com

(c) 1993-2006 Grontmij
Licensed to Leica Geosystems AG

Created: 04/25/2010 22:50:26

Project Information

Project name:	1127.60548.01 FNSB LiDAR Mapping STS01
Date created:	04/22/2010 00:21:31
Time zone:	-8h 00'
Coordinate system name:	AK Zone 3 NAD83 Geoid09
Application software:	LEICA Geo Office 5.0
Processing kernel:	MOVE3 3.4

General Information

**Adjustment**		
Type:	Constrained	
Dimension:	3D	
Coordinate system:	WGS 1984	
Height mode:	Ellipsoidal	
Number of iterations:	1	
Maximum coord correction in last iteration:	0.00000 fts	✓ (tolerance is met)
**Stations**		
Number of (partly) known stations:	6	
Number of unknown stations:	12	
Total:	18	
**Observations**		
GPS coordinate differences:	342 (114 baselines)	
Known coordinates:	16	
Total:	358	
**Unknowns**		
Coordinates:	54	
Total:	54	
Degrees of freedom:	304	
**Testing**		
Alfa (multi dimensional):	0.6497	
Alfa 0 (one dimensional):	1.0 %	
Beta:	80.0 %	
Sigma a-priori (GPS):	10.0	

Critical value W-test:	2.58		
Critical value T-test (2-dimensional):	3.81		
Critical value T-test (3-dimensional):	2.83		
Critical value F-test:	0.97		
F-test:	0.08	✔	(accepted)

Results based on a-posteriori variance factor

Adjustment Results

Coordinates

Station		Coordinate	Corr	Sd	
1	Latitude	64° 45' 28.83429" N	0.00085 fts	0.00292 fts	
	Longitude	147° 13' 16.86361" W	0.02347 fts	0.00285 fts	
	Height	550.27764 fts	-0.03822 fts	0.00344 fts	
401	Latitude	64° 49' 21.41285" N	0.00240 fts	0.00351 fts	
	Longitude	147° 49' 35.18763" W	0.00320 fts	0.00338 fts	
	Height	465.23519 fts	-0.02146 fts	0.00387 fts	
402	Latitude	64° 43' 18.04977" N	-0.00352 fts	0.00551 fts	
	Longitude	147° 17' 04.41267" W	0.02255 fts	0.00538 fts	
	Height	546.24221 fts	-0.05676 fts	0.00597 fts	
403	Latitude	64° 57' 09.00166" N	0.01131 fts	0.00856 fts	
	Longitude	147° 37' 19.95371" W	0.00760 fts	0.00846 fts	
	Height	770.18662 fts	0.02884 fts	0.00886 fts	
551	Latitude	64° 48' 50.38170" N	0.00000 fts	-	fixed
	Longitude	147° 51' 08.10888" W	0.00000 fts	-	fixed
	Height	462.53516 fts	0.00000 fts	-	fixed
552	Latitude	64° 51' 48.48997" N	0.00000 fts	-	fixed
	Longitude	147° 38' 34.60171" W	0.00000 fts	-	fixed
	Height	1131.46995 fts	0.00000 fts	-	fixed
701	Latitude	64° 48' 40.88746" N	-0.04432 fts	0.01086 fts	
	Longitude	147° 34' 35.30746" W	0.02366 fts	0.01076 fts	
	Height	487.74712 fts	-0.01109 fts	0.01155 fts	
702	Latitude	64° 47' 49.58474" N	0.00000 fts	-	fixed
	Longitude	147° 40' 09.75174" W	0.00000 fts	-	fixed
	Height	490.06605 fts	0.00000 fts	-	fixed
703	Latitude	64° 50' 58.46942" N	0.00273 fts	0.00449 fts	
	Longitude	147° 51' 52.60865" W	0.00229 fts	0.00433 fts	
	Height	489.31739 fts	-0.02195 fts	0.00489 fts	
704	Latitude	64° 47' 15.07651" N	0.00554 fts	0.00561 fts	
	Longitude	147° 58' 13.72314" W	0.00346 fts	0.00551 fts	
	Height	579.34773 fts	-0.02241 fts	0.00594 fts	
705	Latitude	64° 51' 21.30427" N	0.00220 fts	0.00449 fts	
	Longitude	147° 49' 08.66623" W	0.00264 fts	0.00436 fts	
	Height	553.84564 fts	-0.02103 fts	0.00489 fts	
706	Latitude	64° 45' 02.34370" N	0.00000 fts	-	fixed
	Longitude	147° 21' 12.46525" W	0.00000 fts	-	fixed
	Height	525.58356 fts	0.12585 fts	0.00492 fts	
707	Latitude	64° 44' 00.61851" N	0.00000 fts	-	fixed
	Longitude	147° 21' 31.73227" W	0.00000 fts	-	fixed
	Height	528.52381 fts	0.24961 fts	0.00620 fts	

708	Latitude	64° 43' 25.09175" N	0.00000 fts	-	fixed
	Longitude	147° 16' 22.58812" W	0.00000 fts	-	fixed
	Height	557.22087 fts	0.00000 fts	-	fixed
709	Latitude	64° 53' 32.88237" N	-0.00548 fts	0.00623 fts	
	Longitude	147° 25' 03.24278" W	0.01090 fts	0.00617 fts	
	Height	641.03271 fts	-0.02723 fts	0.00646 fts	
729	Latitude	64° 47' 27.82469" N	-0.00136 fts	0.00328 fts	
	Longitude	147° 10' 54.06599" W	0.02103 fts	0.00322 fts	
	Height	562.57256 fts	-0.03632 fts	0.00371 fts	
734	Latitude	64° 41' 26.19579" N	-0.00148 fts	0.00610 fts	
	Longitude	147° 07' 27.06885" W	0.02346 fts	0.00597 fts	
	Height	565.12400 fts	-0.04117 fts	0.00653 fts	
741	Latitude	64° 56' 55.55680" N	0.01073 fts	0.01010 fts	
	Longitude	147° 38' 44.50502" W	0.00741 fts	0.00988 fts	
	Height	752.27103 fts	0.02657 fts	0.01073 fts	

Observations and Residuals

	Station	Target	Adj obs	Resid	Resid (ENH)	Sd
DX	729	707	-30962.94582 fts	-0.03002 fts	-0.02753 fts	0.00420 fts
DY			12979.05403 fts	0.01342 fts	-0.02815 fts	0.00387 fts
DZ			-9007.55508 fts	-0.02799 fts	-0.01765 fts	0.00630 fts
DX	709	708	-34854.53390 fts	0.01837 fts	0.01821 fts	0.00640 fts
DY			-49115.66348 fts	-0.00988 fts	0.01726 fts	0.00636 fts
DZ			-26362.61978 fts	0.01893 fts	0.01283 fts	0.00669 fts
DX	709	707	-39346.99864 fts	-0.00791 fts	-0.02339 fts	0.00669 fts
DY			-36046.07473 fts	0.02270 fts	0.00400 fts	0.00653 fts
DZ			-24847.54328 fts	-0.00240 fts	-0.00453 fts	0.00810 fts
DX	1	707	-18419.94239 fts	-0.03458 fts	-0.02615 fts	0.00390 fts
DY			13696.65164 fts	0.00883 fts	-0.02615 fts	0.00358 fts
DZ			-3843.76180 fts	-0.00978 fts	0.00151 fts	0.00610 fts
DX	1	702	-26698.02029 fts	-0.00341 fts	-0.00036 fts	0.00335 fts
DY			65910.27595 fts	-0.00177 fts	-0.03189 fts	0.00328 fts
DZ			6039.78989 fts	-0.06663 fts	-0.05863 fts	0.00387 fts
DX	701	707	-3462.45872 fts	-0.02736 fts	-0.01693 fts	0.01093 fts
DY			-42507.37112 fts	0.00269 fts	-0.02231 fts	0.01083 fts
DZ			-12100.58246 fts	-0.00633 fts	0.00348 fts	0.01178 fts
DX	552	707	-12255.55531 fts	-0.00741 fts	-0.02415 fts	0.00308 fts
DY			-60343.54621 fts	0.02388 fts	0.00709 fts	0.00259 fts
DZ			-20788.26036 fts	0.00285 fts	-0.00020 fts	0.00591 fts
DX	552	706	-7027.21541 fts	-0.01355 fts	-0.00499 fts	0.00269 fts
DY			-57987.48442 fts	-0.00269 fts	-0.01191 fts	0.00233 fts
DZ			-18114.81666 fts	-0.00056 fts	0.00499 fts	0.00482 fts
DX	702	708	12770.54263 fts	0.00676 fts	0.02267 fts	0.00200 fts
DY			-65283.21307 fts	-0.02254 fts	0.02054 fts	0.00197 fts
DZ			-11398.62819 fts	0.06171 fts	0.05853 fts	0.00210 fts
DX	702	707	8278.07790 fts	-0.01250 fts	-0.01791 fts	0.00308 fts
DY			-52213.62435 fts	0.01329 fts	0.01555 fts	0.00262 fts
DZ			-9883.55169 fts	0.04386 fts	0.04117 fts	0.00591 fts
DX	702	552	20533.63324 fts	-0.00095 fts	-0.00551 fts	0.00187 fts
DY			8129.92186 fts	0.00591 fts	0.00443 fts	0.00184 fts
DZ			10904.70870 fts	0.00538 fts	0.00387 fts	0.00194 fts
DX	708	707	-4492.46474 fts	-0.02913 fts	-0.01617 fts	0.00305 fts
DY			13069.58876 fts	0.00049 fts	-0.02982 fts	0.00259 fts

DZ			1515.07650 fts	-0.01850 fts	-0.00636 fts	0.00577 fts
DX	551	707	18749.50555 fts	-0.01732 fts	-0.03927 fts	0.00518 fts
DY			-79317.19950 fts	0.03550 fts	-0.00689 fts	0.00492 fts
DZ			-12488.29582 fts	-0.02520 fts	-0.02461 fts	0.00725 fts
DX	551	702	10471.42765 fts	-0.00135 fts	0.00240 fts	0.00453 fts
DY			-27103.57519 fts	-0.00367 fts	-0.00154 fts	0.00449 fts
DZ			-2604.74417 fts	0.00299 fts	0.00404 fts	0.00495 fts
DX	729	741	5069.22342 fts	-0.02313 fts	0.02490 fts	0.01033 fts
DY			88891.72671 fts	-0.04455 fts	-0.00617 fts	0.01020 fts
DZ			24669.90283 fts	0.07812 fts	0.08924 fts	0.01132 fts
DX	729	734	-23044.97235 fts	-0.00164 fts	-0.00364 fts	0.00620 fts
DY			-25574.12533 fts	0.00325 fts	0.00194 fts	0.00617 fts
DZ			-15676.87928 fts	0.00374 fts	0.00325 fts	0.00669 fts
DX	729	709	8384.05282 fts	-0.01079 fts	-0.00879 fts	0.00682 fts
DY			49025.12875 fts	0.00351 fts	-0.03310 fts	0.00679 fts
DZ			15839.98820 fts	-0.06250 fts	-0.05348 fts	0.00719 fts
DX	729	403	8057.82686 fts	-0.01056 fts	0.00079 fts	0.00892 fts
DY			86474.04795 fts	-0.00774 fts	-0.00279 fts	0.00889 fts
DZ			25264.55987 fts	0.02123 fts	0.02477 fts	0.00938 fts
DX	709	705	-43629.42478 fts	-0.00607 fts	0.00026 fts	0.00735 fts
DY			46265.19261 fts	-0.00420 fts	-0.01722 fts	0.00728 fts
DZ			-5755.74597 fts	-0.02480 fts	-0.01932 fts	0.00781 fts
DX	709	703	-49156.58194 fts	-0.00138 fts	0.00203 fts	0.00738 fts
DY			51161.90128 fts	-0.00331 fts	-0.00676 fts	0.00732 fts
DZ			-6800.12482 fts	-0.00968 fts	-0.00751 fts	0.00784 fts
DX	734	403	31102.79921 fts	-0.00627 fts	-0.00597 fts	0.01017 fts
DY			112048.17328 fts	0.00305 fts	-0.00341 fts	0.01010 fts
DZ			40941.43915 fts	-0.00036 fts	0.00121 fts	0.01073 fts
DX	1	734	-10501.96892 fts	-0.00259 fts	-0.00151 fts	0.00604 fts
DY			-24856.52771 fts	0.00013 fts	0.00098 fts	0.00600 fts
DZ			-10513.08599 fts	0.00676 fts	0.00699 fts	0.00653 fts
DX	1	709	20927.05624 fts	-0.01998 fts	-0.00190 fts	0.00669 fts
DY			49742.72637 fts	-0.01060 fts	-0.02684 fts	0.00666 fts
DZ			21003.78148 fts	-0.01516 fts	-0.00410 fts	0.00705 fts
DX	1	705	-22702.36854 fts	-0.01942 fts	0.01207 fts	0.00568 fts
DY			96007.91894 fts	-0.02687 fts	-0.04416 fts	0.00561 fts
DZ			15248.03551 fts	-0.03809 fts	-0.02126 fts	0.00630 fts
DX	1	703	-28229.52566 fts	-0.01066 fts	0.01293 fts	0.00571 fts
DY			100904.62765 fts	-0.02224 fts	-0.02710 fts	0.00564 fts
DZ			14203.65666 fts	-0.01896 fts	-0.00820 fts	0.00636 fts
DX	1	403	20600.83029 fts	0.01732 fts	0.01611 fts	0.00886 fts
DY			87191.64553 fts	-0.00801 fts	-0.01411 fts	0.00883 fts
DZ			30428.35315 fts	-0.05476 fts	-0.05387 fts	0.00935 fts
DX	701	709	35884.53991 fts	0.02119 fts	0.00584 fts	0.01109 fts
DY			-6461.29639 fts	0.00653 fts	0.00974 fts	0.01102 fts
DZ			12746.96082 fts	-0.02254 fts	-0.02949 fts	0.01171 fts
DX	706	734	2689.63356 fts	0.00725 fts	0.02343 fts	0.00630 fts
DY			-40909.24115 fts	-0.02316 fts	-0.00453 fts	0.00617 fts
DZ			-9342.76790 fts	0.00292 fts	0.00538 fts	0.00732 fts
DX	704	705	31726.06109 fts	0.00476 fts	-0.00059 fts	0.00627 fts
DY			-7943.49838 fts	0.00371 fts	0.00472 fts	0.00617 fts
DZ			10620.81488 fts	-0.00164 fts	-0.00404 fts	0.00689 fts
DX	704	703	26198.90393 fts	0.00344 fts	0.00066 fts	0.00617 fts
DY			-3046.78971 fts	0.00138 fts	0.00276 fts	0.00610 fts

			9576.43606 fts	-0.00128 fts	-0.00272 fts	0.00679 fts
DX	552	734	-4337.58184 fts	0.01722 fts	0.00823 fts	0.00620 fts
DY			-98896.72557 fts	0.00115 fts	0.00433 fts	0.00614 fts
DZ			-27457.58455 fts	-0.02211 fts	-0.02644 fts	0.00676 fts
DX	552	709	27091.44332 fts	0.03169 fts	0.00492 fts	0.00636 fts
DY			-24297.47148 fts	0.01427 fts	0.02913 fts	0.00633 fts
DZ			4059.28292 fts	-0.00476 fts	-0.01893 fts	0.00669 fts
DX	552	705	-16537.98146 fts	0.00390 fts	-0.00010 fts	0.00505 fts
DY			21967.72112 fts	0.00259 fts	0.00118 fts	0.00499 fts
DZ			-1696.46308 fts	-0.00728 fts	-0.00860 fts	0.00558 fts
DX	552	703	-22065.13861 fts	0.00157 fts	-0.00020 fts	0.00512 fts
DY			26864.42980 fts	0.00121 fts	-0.00177 fts	0.00505 fts
DZ			-2740.84189 fts	-0.00833 fts	-0.00840 fts	0.00564 fts
DX	552	403	26765.21737 fts	0.00062 fts	-0.00351 fts	0.00866 fts
DY			13151.44771 fts	0.00453 fts	0.00840 fts	0.00860 fts
DZ			13483.85459 fts	0.01352 fts	0.01096 fts	0.00909 fts
DX	552	401	-26449.53024 fts	0.00154 fts	-0.00315 fts	0.00440 fts
DY			17086.85641 fts	0.00472 fts	-0.00430 fts	0.00433 fts
DZ			-6955.93589 fts	-0.01827 fts	-0.01818 fts	0.00482 fts
DX	703	705	5527.15715 fts	0.00125 fts	0.00072 fts	0.00495 fts
DY			-4896.70868 fts	-0.00007 fts	0.00062 fts	0.00486 fts
DZ			1044.37882 fts	-0.00069 fts	-0.00105 fts	0.00574 fts
DX	403	741	-2988.60344 fts	0.00046 fts	-0.00187 fts	0.00689 fts
DY			2417.67879 fts	0.00253 fts	-0.00069 fts	0.00669 fts
DZ			-594.65705 fts	-0.00535 fts	-0.00558 fts	0.00846 fts
DX	403	402	-36050.78892 fts	0.03609 fts	0.01975 fts	0.00981 fts
DY			-85383.26453 fts	-0.00052 fts	0.00843 fts	0.00971 fts
DZ			-36102.64627 fts	-0.04472 fts	-0.05328 fts	0.01037 fts
DX	702	729	39241.02372 fts	0.00377 fts	0.01716 fts	0.00364 fts
DY			-65192.67834 fts	-0.01791 fts	0.03379 fts	0.00358 fts
DZ			-875.99661 fts	0.09291 fts	0.08678 fts	0.00413 fts
DX	702	709	47625.07653 fts	-0.01532 fts	-0.00020 fts	0.00640 fts
DY			-16167.54959 fts	-0.00948 fts	0.00627 fts	0.00636 fts
DZ			14963.99159 fts	0.05299 fts	0.05561 fts	0.00673 fts
DX	702	705	3995.65175 fts	-0.00374 fts	-0.00656 fts	0.00505 fts
DY			30097.64299 fts	0.00541 fts	0.00062 fts	0.00499 fts
DZ			9208.24563 fts	0.00207 fts	0.00200 fts	0.00558 fts
DX	702	703	-1531.50537 fts	-0.00584 fts	-0.00633 fts	0.00509 fts
DY			34994.35166 fts	0.00381 fts	0.00069 fts	0.00502 fts
DZ			8163.86681 fts	0.00781 fts	0.00830 fts	0.00564 fts
DX	708	734	3425.50873 fts	-0.01749 fts	-0.01276 fts	0.00610 fts
DY			-25483.59060 fts	0.00394 fts	-0.00669 fts	0.00604 fts
DZ			-5154.24770 fts	0.01102 fts	0.01535 fts	0.00663 fts
DX	708	403	34528.30794 fts	0.00190 fts	-0.00715 fts	0.00869 fts
DY			86564.58264 fts	0.00974 fts	-0.00574 fts	0.00866 fts
DZ			35787.19145 fts	-0.02802 fts	-0.02825 fts	0.00912 fts
DX	551	741	54781.67479 fts	0.00722 fts	0.00512 fts	0.01037 fts
DY			-3404.52682 fts	-0.00151 fts	0.00128 fts	0.01024 fts
DZ			21189.16205 fts	-0.00833 fts	-0.00981 fts	0.01132 fts
DX	551	734	26667.47901 fts	0.02320 fts	0.00387 fts	0.00738 fts
DY			-117870.37886 fts	0.01001 fts	-0.00814 fts	0.00732 fts
DZ			-19157.62002 fts	-0.07221 fts	-0.07598 fts	0.00797 fts
DX	551	709	58096.50418 fts	-0.01542 fts	-0.01453 fts	0.00709 fts
DY			-43271.12478 fts	0.00745 fts	-0.01319 fts	0.00702 fts

			12359.24746 fts	-0.01165 fts	-0.00666 fts	0.00748 fts
DX	551	705	14467.07940 fts	0.00184 fts	0.00092 fts	0.00489 fts
DY			2994.06780 fts	0.00007 fts	0.00315 fts	0.00479 fts
DZ			6603.50146 fts	0.00400 fts	0.00295 fts	0.00561 fts
DX	551	703	8939.92228 fts	0.00069 fts	0.00125 fts	0.00482 fts
DY			7890.77651 fts	-0.00102 fts	0.00262 fts	0.00472 fts
DZ			5559.12264 fts	0.00607 fts	0.00545 fts	0.00558 fts
DX	551	403	57770.27823 fts	0.01798 fts	-0.00466 fts	0.00909 fts
DY			-5822.20558 fts	0.01677 fts	-0.00144 fts	0.00902 fts
DZ			21783.81913 fts	-0.05476 fts	-0.05981 fts	0.00955 fts
DX	709	704	-75355.48587 fts	0.01135 fts	-0.00052 fts	0.00810 fts
DY			54208.69099 fts	0.00791 fts	0.00896 fts	0.00807 fts
DZ			-16376.56088 fts	-0.00840 fts	-0.01348 fts	0.00856 fts
DX	1	704	-54428.42959 fts	0.00522 fts	0.01230 fts	0.00663 fts
DY			103951.41733 fts	-0.01125 fts	-0.01050 fts	0.00656 fts
DZ			4627.22063 fts	-0.02100 fts	-0.01824 fts	0.00719 fts
DX	1	701	-14957.48363 fts	-0.00289 fts	-0.01486 fts	0.01099 fts
DY			56204.02273 fts	0.01581 fts	-0.01480 fts	0.01093 fts
DZ			8256.82066 fts	-0.04767 fts	-0.04573 fts	0.01168 fts
DX	552	704	-48264.04255 fts	0.00745 fts	-0.00102 fts	0.00614 fts
DY			29911.21951 fts	0.00591 fts	0.00630 fts	0.00607 fts
DZ			-12317.27796 fts	-0.00531 fts	-0.00883 fts	0.00659 fts
DX	702	704	-27730.40930 fts	-0.01325 fts	-0.01033 fts	0.00614 fts
DY			38041.14137 fts	0.00381 fts	-0.00374 fts	0.00607 fts
DZ			-1412.56925 fts	0.01070 fts	0.01355 fts	0.00656 fts
DX	551	704	-17258.98166 fts	-0.00243 fts	-0.00033 fts	0.00587 fts
DY			10937.56618 fts	-0.00115 fts	-0.00036 fts	0.00581 fts
DZ			-4017.31342 fts	0.00482 fts	0.00548 fts	0.00646 fts
DX	551	1	37169.44794 fts	-0.00325 fts	-0.01706 fts	0.00518 fts
DY			-93013.85114 fts	0.01811 fts	0.00974 fts	0.00512 fts
DZ			-8644.53406 fts	0.00830 fts	0.00456 fts	0.00571 fts
DX	729	706	-25734.60588 fts	0.01421 fts	0.00220 fts	0.00377 fts
DY			15335.11579 fts	0.00656 fts	0.02484 fts	0.00361 fts
DZ			-6334.11135 fts	0.02536 fts	0.01637 fts	0.00502 fts
DX	729	706	-25734.60588 fts	0.00433 fts	0.00906 fts	0.00377 fts
DY			15335.11579 fts	-0.00797 fts	0.00266 fts	0.00361 fts
DZ			-6334.11135 fts	0.00768 fts	0.00722 fts	0.00502 fts
DX	1	552	-6164.38705 fts	-0.01591 fts	0.00423 fts	0.00328 fts
DY			74040.19782 fts	-0.01529 fts	-0.02110 fts	0.00322 fts
DZ			16944.49859 fts	-0.00354 fts	0.00604 fts	0.00381 fts
DX	1	552	-6164.38705 fts	0.00003 fts	0.00791 fts	0.00328 fts
DY			74040.19782 fts	-0.00935 fts	-0.02402 fts	0.00322 fts
DZ			16944.49859 fts	-0.04564 fts	-0.03914 fts	0.00381 fts
DX	706	708	-735.87517 fts	0.00951 fts	0.01703 fts	0.00262 fts
DY			-15425.65051 fts	-0.01414 fts	-0.00709 fts	0.00233 fts
DZ			-4188.52023 fts	-0.01745 fts	-0.01594 fts	0.00463 fts
DX	706	708	-735.87517 fts	-0.00430 fts	-0.00820 fts	0.00262 fts
DY			-15425.65051 fts	0.00699 fts	0.01099 fts	0.00233 fts
DZ			-4188.52023 fts	0.02543 fts	0.02293 fts	0.00463 fts
DX	552	708	-7763.09058 fts	0.01608 fts	0.01663 fts	0.00194 fts
DY			-73413.13493 fts	-0.00951 fts	0.01047 fts	0.00194 fts
DZ			-22303.33689 fts	0.00659 fts	0.00236 fts	0.00203 fts
DX	552	708	-7763.09058 fts	-0.00397 fts	0.00338 fts	0.00194 fts
DY			-73413.13493 fts	-0.00653 fts	0.00659 fts	0.00194 fts

			-22303.33689 fts	0.03012 fts	0.03018 fts	0.00203 fts
DX	551	708	23241.97028 fts	-0.00039 fts	0.00203 fts	0.00466 fts
DY			-92386.78826 fts	-0.00266 fts	-0.00482 fts	0.00459 fts
DZ			-14003.37235 fts	-0.00761 fts	-0.00614 fts	0.00505 fts
DX	551	708	23241.97028 fts	0.00190 fts	-0.00010 fts	0.00466 fts
DY			-92386.78826 fts	0.00135 fts	-0.00879 fts	0.00459 fts
DZ			-14003.37235 fts	-0.02559 fts	-0.02415 fts	0.00505 fts
DX	551	552	31005.06086 fts	-0.00240 fts	-0.00423 fts	0.00453 fts
DY			-18973.65329 fts	0.00351 fts	0.00322 fts	0.00446 fts
DZ			8299.96454 fts	0.00787 fts	0.00719 fts	0.00492 fts
DX	551	552	31005.06086 fts	0.00489 fts	0.00846 fts	0.00453 fts
DY			-18973.65329 fts	-0.00692 fts	-0.00814 fts	0.00446 fts
DZ			8299.96454 fts	-0.02011 fts	-0.01837 fts	0.00492 fts
DX	705	401	-9911.54878 fts	0.00197 fts	-0.00174 fts	0.00440 fts
DY			-4880.86471 fts	0.00328 fts	0.00207 fts	0.00430 fts
DZ			-5259.47281 fts	-0.00240 fts	-0.00361 fts	0.00505 fts
DX	705	401	-9911.54878 fts	0.00079 fts	0.00138 fts	0.00440 fts
DY			-4880.86471 fts	-0.00115 fts	-0.00338 fts	0.00430 fts
DZ			-5259.47281 fts	-0.00807 fts	-0.00732 fts	0.00505 fts
DX	729	402	-27992.96206 fts	0.01184 fts	0.01991 fts	0.00538 fts
DY			1090.78342 fts	-0.01608 fts	0.00305 fts	0.00531 fts
DZ			-10838.08639 fts	0.00446 fts	0.00351 fts	0.00591 fts
DX	729	402	-27992.96206 fts	-0.00308 fts	-0.02123 fts	0.00538 fts
DY			1090.78342 fts	0.02326 fts	0.00722 fts	0.00531 fts
DZ			-10838.08639 fts	-0.00427 fts	-0.00814 fts	0.00591 fts
DX	709	401	-53540.97356 fts	0.01440 fts	-0.00262 fts	0.00689 fts
DY			41384.32789 fts	0.01234 fts	-0.00906 fts	0.00686 fts
DZ			-11015.21882 fts	-0.06142 fts	-0.06358 fts	0.00728 fts
DX	709	401	-53540.97356 fts	0.02365 fts	0.00719 fts	0.00689 fts
DY			41384.32789 fts	0.00659 fts	0.01398 fts	0.00686 fts
DZ			-11015.21882 fts	-0.01716 fts	-0.02549 fts	0.00728 fts
DX	734	402	-4947.98971 fts	0.00643 fts	0.02011 fts	0.00643 fts
DY			26664.90874 fts	-0.01982 fts	-0.00581 fts	0.00636 fts
DZ			4838.79288 fts	-0.00230 fts	0.00020 fts	0.00699 fts
DX	734	402	-4947.98971 fts	-0.01818 fts	-0.02123 fts	0.00643 fts
DY			26664.90874 fts	0.01352 fts	-0.00223 fts	0.00636 fts
DZ			4838.79288 fts	0.01158 fts	0.01388 fts	0.00699 fts
DX	1	402	-15449.95860 fts	0.00705 fts	0.02244 fts	0.00515 fts
DY			1808.38103 fts	-0.02218 fts	-0.00128 fts	0.00509 fts
DZ			-5674.29311 fts	0.00984 fts	0.01148 fts	0.00568 fts
DX	1	402	-15449.95860 fts	-0.01860 fts	-0.02385 fts	0.00515 fts
DY			1808.38103 fts	0.01640 fts	0.00089 fts	0.00509 fts
DZ			-5674.29311 fts	0.01644 fts	0.01775 fts	0.00568 fts
DX	1	401	-32613.91732 fts	0.00915 fts	0.03750 fts	0.00509 fts
DY			91127.05426 fts	-0.03871 fts	-0.02674 fts	0.00502 fts
DZ			9988.56270 fts	-0.03458 fts	-0.02562 fts	0.00561 fts
DX	1	401	-32613.91732 fts	0.01581 fts	0.02556 fts	0.00509 fts
DY			91127.05426 fts	-0.02024 fts	-0.01250 fts	0.00502 fts
DZ			9988.56270 fts	-0.03422 fts	-0.03196 fts	0.00561 fts
DX	704	401	21814.51231 fts	-0.00889 fts	-0.00423 fts	0.00558 fts
DY			-12824.36310 fts	-0.00056 fts	-0.00249 fts	0.00551 fts
DZ			5361.34207 fts	0.01076 fts	0.01306 fts	0.00610 fts
DX	704	401	21814.51231 fts	-0.00200 fts	-0.00033 fts	0.00558 fts
DY			-12824.36310 fts	-0.00089 fts	-0.00344 fts	0.00551 fts

			5361.34207 fts	-0.00348 fts	-0.00223 fts	0.00610 fts
DX	552	729	18707.39051 fts	0.00860 fts	0.01437 fts	0.00361 fts
DY			-73322.60024 fts	-0.01158 fts	0.02303 fts	0.00354 fts
DZ			-11780.70531 fts	0.05200 fts	0.04662 fts	0.00407 fts
DX	552	729	18707.39051 fts	0.02657 fts	0.00430 fts	0.00361 fts
DY			-73322.60024 fts	0.01175 fts	0.01234 fts	0.00354 fts
DZ			-11780.70531 fts	-0.03225 fts	-0.04140 fts	0.00407 fts
DX	552	402	-9285.57155 fts	0.03980 fts	0.00817 fts	0.00561 fts
DY			-72231.81682 fts	0.01558 fts	-0.00505 fts	0.00554 fts
DZ			-22618.79170 fts	-0.10131 fts	-0.10955 fts	0.00623 fts
DX	552	402	-9285.57155 fts	0.02762 fts	-0.00436 fts	0.00561 fts
DY			-72231.81682 fts	0.02267 fts	0.01444 fts	0.00554 fts
DZ			-22618.79170 fts	-0.04160 fts	-0.05276 fts	0.00623 fts
DX	703	401	-4384.39162 fts	-0.00112 fts	-0.00059 fts	0.00433 fts
DY			-9777.57339 fts	0.00000 fts	0.00000 fts	0.00427 fts
DZ			-4215.09400 fts	0.00203 fts	0.00223 fts	0.00502 fts
DX	703	401	-4384.39162 fts	-0.00056 fts	0.00003 fts	0.00433 fts
DY			-9777.57339 fts	-0.00039 fts	-0.00062 fts	0.00427 fts
DZ			-4215.09400 fts	0.00000 fts	0.00030 fts	0.00502 fts
DX	702	401	-5915.89703 fts	-0.00154 fts	-0.00374 fts	0.00436 fts
DY			25216.77827 fts	0.00344 fts	0.00600 fts	0.00433 fts
DZ			3948.77281 fts	0.01296 fts	0.01148 fts	0.00479 fts
DX	702	401	-5915.89703 fts	0.00138 fts	-0.00384 fts	0.00436 fts
DY			25216.77827 fts	0.00541 fts	0.00758 fts	0.00433 fts
DZ			3948.77281 fts	0.00912 fts	0.00653 fts	0.00479 fts
DX	551	729	49712.45136 fts	-0.00761 fts	-0.00410 fts	0.00538 fts
DY			-92296.25353 fts	0.00003 fts	0.00594 fts	0.00531 fts
DZ			-3480.74077 fts	0.02762 fts	0.02772 fts	0.00587 fts
DX	551	729	49712.45136 fts	0.02090 fts	-0.00016 fts	0.00538 fts
DY			-92296.25353 fts	0.01332 fts	-0.00525 fts	0.00531 fts
DZ			-3480.74077 fts	-0.06503 fts	-0.06939 fts	0.00587 fts
DX	551	401	4555.53062 fts	-0.00285 fts	0.00036 fts	0.00390 fts
DY			-1886.79688 fts	-0.00220 fts	0.00213 fts	0.00381 fts
DZ			1344.02864 fts	0.01260 fts	0.01293 fts	0.00459 fts
DX	551	401	4555.53062 fts	0.00056 fts	0.00141 fts	0.00390 fts
DY			-1886.79688 fts	-0.00131 fts	0.00157 fts	0.00381 fts
DZ			1344.02864 fts	0.00417 fts	0.00387 fts	0.00459 fts
DX	1	706	-13191.60246 fts	0.00879 fts	-0.00417 fts	0.00341 fts
DY			16052.71340 fts	0.01060 fts	0.01814 fts	0.00325 fts
DZ			-1170.31807 fts	0.01467 fts	0.00768 fts	0.00469 fts
DX	1	706	-13191.60246 fts	0.01978 fts	0.00909 fts	0.00341 fts
DY			16052.71340 fts	0.00194 fts	0.01257 fts	0.00325 fts
DZ			-1170.31807 fts	-0.00807 fts	-0.01483 fts	0.00469 fts
DX	1	706	-13191.60246 fts	0.00171 fts	0.00476 fts	0.00341 fts
DY			16052.71340 fts	-0.00456 fts	0.00069 fts	0.00325 fts
DZ			-1170.31807 fts	0.00381 fts	0.00387 fts	0.00469 fts
DX	1	708	-13927.47766 fts	-0.00653 fts	-0.00833 fts	0.00302 fts
DY			627.06289 fts	0.00571 fts	0.00305 fts	0.00299 fts
DZ			-5358.83830 fts	0.01220 fts	0.01207 fts	0.00351 fts
DX	1	708	-13927.47766 fts	0.00627 fts	0.01070 fts	0.00302 fts
DY			627.06289 fts	-0.00869 fts	0.00354 fts	0.00299 fts
DZ			-5358.83830 fts	0.00712 fts	0.00620 fts	0.00351 fts
DX	1	708	-13927.47766 fts	-0.00594 fts	-0.00541 fts	0.00302 fts
DY			627.06289 fts	0.00259 fts	0.00801 fts	0.00299 fts

			-5358.83830 fts	0.02638 fts	0.02539 fts	0.00351 fts
**DX**	1	708	-13927.47766 fts	0.01470 fts	0.00535 fts	0.00302 fts
**DY**			627.06289 fts	0.00312 fts	0.01358 fts	0.00299 fts
**DZ**			-5358.83830 fts	0.00200 fts	-0.00417 fts	0.00351 fts
**DX**	729	708	-26470.48108 fts	-0.00433 fts	-0.01004 fts	0.00338 fts
**DY**			-90.53473 fts	0.00915 fts	-0.00220 fts	0.00331 fts
**DZ**			-10522.63158 fts	-0.00801 fts	-0.00781 fts	0.00381 fts
**DX**	729	708	-26470.48108 fts	0.01037 fts	0.00866 fts	0.00338 fts
**DY**			-90.53473 fts	-0.00364 fts	0.00151 fts	0.00331 fts
**DZ**			-10522.63158 fts	-0.01076 fts	-0.01260 fts	0.00381 fts
**DX**	729	708	-26470.48108 fts	-0.00272 fts	-0.00390 fts	0.00338 fts
**DY**			-90.53473 fts	0.00289 fts	0.01171 fts	0.00331 fts
**DZ**			-10522.63158 fts	0.02900 fts	0.02654 fts	0.00381 fts
**DX**	729	708	-26470.48108 fts	0.02621 fts	0.02251 fts	0.00338 fts
**DY**			-90.53473 fts	-0.00991 fts	0.00531 fts	0.00331 fts
**DZ**			-10522.63158 fts	-0.02287 fts	-0.02779 fts	0.00381 fts
**DX**	729	708	-26470.48108 fts	0.01115 fts	0.00630 fts	0.00338 fts
**DY**			-90.53473 fts	-0.00026 fts	0.01421 fts	0.00331 fts
**DZ**			-10522.63158 fts	0.01375 fts	0.00850 fts	0.00381 fts
**DX**	1	729	12543.00342 fts	-0.00433 fts	-0.00161 fts	0.00305 fts
**DY**			717.59761 fts	-0.00085 fts	0.00371 fts	0.00302 fts
**DZ**			5163.79328 fts	0.01745 fts	0.01752 fts	0.00348 fts
**DX**	1	729	12543.00342 fts	0.00548 fts	-0.00056 fts	0.00305 fts
**DY**			717.59761 fts	0.00417 fts	-0.00361 fts	0.00302 fts
**DZ**			5163.79328 fts	-0.02297 fts	-0.02372 fts	0.00348 fts
**DX**	1	729	12543.00342 fts	0.00020 fts	0.00554 fts	0.00305 fts
**DY**			717.59761 fts	-0.00646 fts	0.00581 fts	0.00302 fts
**DZ**			5163.79328 fts	0.02070 fts	0.02014 fts	0.00348 fts
**DX**	1	729	12543.00342 fts	-0.00256 fts	-0.00112 fts	0.00305 fts
**DY**			717.59761 fts	-0.00033 fts	-0.00279 fts	0.00302 fts
**DZ**			5163.79328 fts	-0.00161 fts	-0.00046 fts	0.00348 fts
**DX**	1	729	12543.00342 fts	0.00666 fts	-0.00030 fts	0.00305 fts
**DY**			717.59761 fts	0.00463 fts	-0.00095 fts	0.00302 fts
**DZ**			5163.79328 fts	-0.01946 fts	-0.02103 fts	0.00348 fts
**DX**	1	729	12543.00342 fts	0.00367 fts	-0.00164 fts	0.00305 fts
**DY**			717.59761 fts	0.00433 fts	0.00148 fts	0.00302 fts
**DZ**			5163.79328 fts	-0.00807 fts	-0.00961 fts	0.00348 fts

GPS Baseline Vector Residuals

	**Station**	**Target**	**Adj vector [fts]**	**Resid [fts]**	**Resid [ppm]**
DV	729	707	34760.55098	0.04318	1.2
DV	709	708	65743.24798	0.02818	0.4
DV	709	707	58863.45396	0.02415	0.4
DV	1	707	23273.74161	0.03701	1.6
DV	1	702	71368.25503	0.06673	0.9
DV	701	707	44331.58371	0.02822	0.6
DV	552	707	64989.95286	0.02516	0.4
DV	552	706	61156.16640	0.01385	0.2
DV	702	708	67490.09848	0.06604	1.0
DV	702	707	53781.72305	0.04751	0.9
DV	702	552	24630.03033	0.00804	0.3
DV	708	707	13902.94378	0.03451	2.5
DV	551	707	82454.34877	0.04685	0.6

DV	551	702	29172.57748	0.00492	0.2
DV	729	741	92390.69330	0.09285	1.0
DV	729	734	37826.85794	0.00525	0.1
DV	729	709	52198.28366	0.06352	1.2
DV	729	403	90448.81165	0.02493	0.3
DV	709	705	63852.35599	0.02589	0.4
DV	709	703	71275.18071	0.01033	0.1
DV	734	403	123281.70462	0.00699	0.1
DV	1	734	28959.68401	0.00722	0.2
DV	1	709	57908.88832	0.02720	0.5
DV	1	705	99826.95340	0.05049	0.5
DV	1	703	105737.38157	0.03114	0.3
DV	1	403	94618.50738	0.05797	0.6
DV	701	709	38625.55585	0.03159	0.8
DV	706	734	42048.63198	0.02444	0.6
DV	704	705	34386.68096	0.00623	0.2
DV	704	703	28060.17859	0.00394	0.1
DV	552	734	102729.23581	0.02805	0.3
DV	552	709	36616.81578	0.03507	1.0
DV	552	705	27549.29382	0.00866	0.3
DV	552	703	34872.34069	0.00856	0.2
DV	552	403	32728.45508	0.01427	0.4
DV	552	401	32247.84266	0.01893	0.6
DV	703	705	7457.74424	0.00141	0.2
DV	403	741	3889.79924	0.00591	1.5
DV	403	402	99465.38247	0.05745	0.6
DV	702	729	76096.71887	0.09468	1.2
DV	702	709	52473.40870	0.05597	1.1
DV	702	705	31727.35623	0.00689	0.2
DV	702	703	35966.63565	0.01050	0.3
DV	708	734	26224.29732	0.02106	0.8
DV	708	403	99831.62872	0.02972	0.3
DV	551	741	58835.39142	0.01115	0.2
DV	551	734	122358.46949	0.07651	0.6
DV	551	709	73487.03994	0.02073	0.3
DV	551	705	16182.30701	0.00443	0.3
DV	551	703	13156.38281	0.00620	0.5
DV	551	403	62014.82000	0.06001	1.0
DV	709	704	94261.46174	0.01621	0.2
DV	1	704	117429.81855	0.02438	0.2
DV	1	701	58743.45561	0.05033	0.9
DV	552	704	58101.75722	0.01089	0.2
DV	702	704	47096.70253	0.01745	0.4
DV	551	704	20824.06324	0.00551	0.3
DV	551	1	100537.91490	0.02018	0.2
DV	729	706	30619.54742	0.02982	1.0
DV	729	706	30619.54742	0.01188	0.4
DV	1	552	76204.11140	0.02234	0.3
DV	1	552	76204.11140	0.04659	0.6
DV	706	708	16001.12210	0.02438	1.5
DV	706	708	16001.12210	0.02674	1.7
DV	552	708	77118.04455	0.01982	0.3
DV	552	708	77118.04455	0.03107	0.4
DV	551	708	96289.15965	0.00807	0.1

	551	708	96289.15965	0.02569	0.3
DV	551	552	37285.42251	0.00896	0.2
DV	551	552	37285.42251	0.02182	0.6
DV	705	401	12236.16336	0.00453	0.4
DV	705	401	12236.16336	0.00820	0.7
DV	729	402	30037.64055	0.02047	0.7
DV	729	402	30037.64055	0.02385	0.8
DV	709	401	68561.16606	0.06427	0.9
DV	709	401	68561.16606	0.02995	0.4
DV	734	402	27548.39156	0.02096	0.8
DV	734	402	27548.39156	0.02546	0.9
DV	1	402	16558.05137	0.02526	1.5
DV	1	402	16558.05137	0.02972	1.8
DV	1	401	97301.48509	0.05269	0.5
DV	1	401	97301.48509	0.04278	0.4
DV	704	401	25866.60443	0.01394	0.5
DV	704	401	25866.60443	0.00410	0.2
DV	552	729	76582.99539	0.05397	0.7
DV	552	729	76582.99539	0.04341	0.6
DV	552	402	76257.89755	0.10997	1.4
DV	552	402	76257.89755	0.05486	0.7
DV	703	401	11514.80998	0.00230	0.2
DV	703	401	11514.80998	0.00069	0.1
DV	702	401	26200.69753	0.01348	0.5
DV	702	401	26200.69753	0.01070	0.4
DV	551	729	104890.61824	0.02867	0.3
DV	551	729	104890.61824	0.06959	0.7
DV	551	401	5110.70198	0.01312	2.6
DV	551	401	5110.70198	0.00443	0.9
DV	1	706	20810.51724	0.02011	1.0
DV	1	706	20810.51724	0.02146	1.0
DV	1	706	20810.51724	0.00620	0.3
DV	1	708	14936.02990	0.01499	1.0
DV	1	708	14936.02990	0.01286	0.9
DV	1	708	14936.02990	0.02717	1.8
DV	1	708	14936.02990	0.01519	1.0
DV	729	708	28485.44086	0.01289	0.5
DV	729	708	28485.44086	0.01539	0.5
DV	729	708	28485.44086	0.02927	1.0
DV	729	708	28485.44086	0.03619	1.3
DV	729	708	28485.44086	0.01772	0.6
DV	1	729	13583.32221	0.01798	1.3
DV	1	729	13583.32221	0.02398	1.8
DV	1	729	13583.32221	0.02169	1.6
DV	1	729	13583.32221	0.00302	0.2
DV	1	729	13583.32221	0.02106	1.6
DV	1	729	13583.32221	0.00984	0.7

Absolute Error Ellipses (2D - 39.4% 1D - 68.3%)

Station	A [fts]	B [fts]	A/B	Phi	Sd Hgt [fts]
1	0.00292	0.00285	1.0	11°	0.00344
401	0.00351	0.00338	1.0	11°	0.00387
402	0.00551	0.00538	1.0	11°	0.00597

403	0.00856	0.00846	1.0	11°	0.00886
551	0.00000	0.00000	1.0	13°	0.00000
552	0.00000	0.00000	1.0	12°	0.00000
701	0.01089	0.01073	1.0	11°	0.01155
702	0.00000	0.00000	1.0	12°	0.00000
703	0.00449	0.00433	1.0	11°	0.00489
704	0.00561	0.00548	1.0	11°	0.00594
705	0.00449	0.00436	1.0	11°	0.00489
706	0.00000	0.00000	1.0	11°	0.00492
707	0.00000	0.00000	1.0	11°	0.00620
708	0.00000	0.00000	1.0	11°	0.00000
709	0.00623	0.00617	1.0	11°	0.00646
729	0.00328	0.00322	1.0	11°	0.00371
734	0.00610	0.00597	1.0	11°	0.00653
741	0.01010	0.00984	1.0	11°	0.01073

Testing and Estimated Errors

Coordinate Tests

Station		MDB	BNR	W-Test	T-Test
551	Latitude	1.12062 fts	0.0	-0.04	0.00
	Longitude	0.91391 fts	0.1	-0.03	
	Height	2.79992 fts	0.0	0.04	
552	Latitude	0.01769 fts	12.6	1.54	1.80
	Longitude	0.01746 fts	12.7	-0.01	
	Height	0.01888 fts	12.8	-1.80	
702	Latitude	0.01999 fts	13.7	1.61	7.61 ⚠
	Longitude	0.01969 fts	13.8	-0.96	
	Height	0.02121 fts	13.7	4.32 ⚠	
706	Latitude	0.01740 fts	9.0	-1.67	1.43
	Longitude	0.01697 fts	9.0	0.24	
707	Latitude	0.02133 fts	10.7	3.80 ⚠	16.92 ⚠
	Longitude	0.02086 fts	10.6	4.37 ⚠	
708	Latitude	0.01425 fts	8.3	-3.56 ⚠	8.14 ⚠
	Longitude	0.01393 fts	8.2	-2.44	
	Height	0.02095 fts	12.2	-2.26	

Observation Tests

	Station	Target	MDB	Red	BNR	W-Test	T-Test
DX	729	707	0.05370 fts	92	1.0	-2.07	2.81
DY			0.05302 fts	93	0.9	0.93	
DZ			0.05985 fts	84	1.5	-1.89	
DX	709	708	0.08361 fts	92	1.0	0.81	0.50
DY			0.08333 fts	92	1.0	-0.44	
DZ			0.08609 fts	92	1.0	0.81	
DX	709	707	0.07781 fts	90	1.1	-0.38	0.46
DY			0.07735 fts	90	1.1	1.10	
DZ			0.08200 fts	86	1.3	-0.11	
DX	1	707	0.04358 fts	89	1.2	-3.03 ⚠	3.38 ⚠
DY			0.04272 fts	90	1.1	0.78	
DZ			0.05122 fts	78	1.8	-0.83	

DX	1	702	0.08653 fts	98	0.5	-0.14		2.30	
DY			0.08626 fts	98	0.4	-0.07			
DZ			0.08890 fts	97	0.5	-2.62	⚠		
DX	701	707	0.07681 fts	61	2.7	-1.97		1.36	
DY			0.07622 fts	62	2.7	0.19			
DZ			0.08201 fts	59	2.8	-0.44			
DX	552	707	0.08044 fts	98	0.5	-0.32		0.39	
DY			0.07998 fts	98	0.4	1.03			
DZ			0.08462 fts	93	0.9	0.12			
DX	552	706	0.07670 fts	98	0.4	-0.61		0.13	
DY			0.07629 fts	98	0.4	-0.12			
DZ			0.08033 fts	95	0.7	-0.02			
DX	702	708	0.08239 fts	99	0.3	0.28		2.42	
DY			0.08213 fts	99	0.3	-0.94			
DZ			0.08463 fts	99	0.3	2.51			
DX	702	707	0.07005 fts	97	0.5	-0.62		1.86	
DY			0.06951 fts	98	0.4	0.66			
DZ			0.07487 fts	92	1.0	2.17			
DX	702	552	0.04293 fts	97	0.5	-0.08		0.13	
DY			0.04243 fts	97	0.5	0.49			
DZ			0.04708 fts	97	0.5	0.40			
DX	708	707	0.03488 fts	90	1.1	-3.17	⚠	4.15	⚠
DY			0.03377 fts	92	1.0	0.06			
DZ			0.04467 fts	73	2.1	-1.93			
DX	551	707	0.09793 fts	96	0.6	-0.63		0.95	
DY			0.09753 fts	96	0.6	1.28			
DZ			0.10143 fts	93	0.9	-0.91			
DX	551	702	0.04934 fts	88	1.2	-0.11		0.05	
DY			0.04885 fts	88	1.2	-0.29			
DZ			0.05344 fts	88	1.2	0.22			
DX	729	741	0.11210 fts	88	1.2	-0.79		3.35	⚠
DY			0.11176 fts	89	1.2	-1.53			
DZ			0.11511 fts	87	1.3	2.66	⚠		
DX	729	734	0.05909 fts	84	1.4	-0.11		0.04	
DY			0.05867 fts	84	1.4	0.22			
DZ			0.06288 fts	84	1.5	0.24			
DX	729	709	0.07216 fts	88	1.3	-0.58		3.61	⚠
DY			0.07183 fts	88	1.3	0.19			
DZ			0.07516 fts	87	1.3	-3.24	⚠		
DX	729	403	0.10863 fts	91	1.1	-0.36		0.24	
DY			0.10838 fts	91	1.0	-0.27			
DZ			0.11084 fts	90	1.1	0.72			
DX	709	705	0.08300 fts	89	1.1	-0.28		0.45	
DY			0.08269 fts	89	1.1	-0.19			
DZ			0.08579 fts	89	1.2	-1.11			
DX	709	703	0.08963 fts	91	1.1	-0.06		0.06	
DY			0.08934 fts	91	1.0	-0.14			
DZ			0.09222 fts	90	1.1	-0.40			
DX	734	403	0.13980 fts	93	0.9	-0.16		0.01	
DY			0.13959 fts	93	0.9	0.08			
DZ			0.14165 fts	92	1.0	-0.01			
DX	1	734	0.05175 fts	80	1.7	-0.21		0.10	
DY			0.05126 fts	80	1.7	0.01			

DZ			0.05602 fts	80	1.7	0.51		
DX	1	709	0.07695 fts	90	1.1	-0.98	0.59	
DY			0.07663 fts	90	1.1	-0.52		
DZ			0.07974 fts	89	1.1	-0.72		
DX	1	705	0.11445 fts	97	0.6	-0.60	0.79	
DY			0.11422 fts	97	0.6	-0.83		
DZ			0.11646 fts	96	0.7	-1.16		
DX	1	703	0.12001 fts	97	0.6	-0.31	0.27	
DY			0.11979 fts	97	0.6	-0.65		
DZ			0.12193 fts	96	0.6	-0.55		
DX	1	403	0.11233 fts	92	1.0	0.57	1.20	
DY			0.11209 fts	92	1.0	-0.27		
DZ			0.11447 fts	91	1.0	-1.79		
DX	701	709	0.07589 fts	52	3.2	1.81	2.30	
DY			0.07538 fts	52	3.2	0.56		
DZ			0.08025 fts	52	3.2	-1.81		
DX	706	734	0.06273 fts	86	1.4	0.46	0.80	
DY			0.06223 fts	86	1.3	-1.47		
DZ			0.06719 fts	83	1.5	0.18		
DX	704	705	0.05640 fts	82	1.6	0.35	0.07	
DY			0.05589 fts	82	1.6	0.27		
DZ			0.06061 fts	81	1.6	-0.11		
DX	704	703	0.05138 fts	78	1.8	0.29	0.04	
DY			0.05081 fts	78	1.8	0.12		
DZ			0.05599 fts	77	1.8	-0.10		
DX	552	734	0.11750 fts	96	0.6	0.52	0.23	
DY			0.11728 fts	96	0.6	0.03		
DZ			0.11945 fts	96	0.7	-0.66		
DX	552	709	0.05839 fts	83	1.5	2.23	2.03	
DY			0.05798 fts	83	1.5	1.01		
DZ			0.06193 fts	83	1.5	-0.31		
DX	552	705	0.04880 fts	85	1.4	0.32	0.15	
DY			0.04828 fts	85	1.4	0.22		
DZ			0.05318 fts	84	1.4	-0.55		
DX	552	703	0.05493 fts	88	1.2	0.11	0.11	
DY			0.05446 fts	88	1.2	0.09		
DZ			0.05887 fts	87	1.3	-0.55		
DX	552	403	0.06183 fts	64	2.5	0.05	0.43	
DY			0.06140 fts	64	2.5	0.39		
DZ			0.06554 fts	66	2.4	1.06		
DX	552	401	0.05177 fts	90	1.1	0.11	0.56	
DY			0.05131 fts	90	1.1	0.35		
DZ			0.05568 fts	90	1.1	-1.24		
DX	703	705	0.03507 fts	62	2.6	0.19	0.01	
DY			0.03425 fts	62	2.7	-0.01		
DZ			0.04154 fts	66	2.4	-0.09		
DX	403	741	0.07350 fts	11	9.4	0.19	1.07	
DY			0.07299 fts	11	9.7	1.07		
DZ			0.07795 fts	16	7.7	-1.42		
DX	403	402	0.11773 fts	91	1.1	1.15	1.10	
DY			0.11748 fts	91	1.1	-0.02		
DZ			0.11996 fts	90	1.1	-1.41		
DX	702	729	0.09110 fts	98	0.5	0.14	4.21	⚠

DY			0.09085 fts	98	0.5	-0.69		
DZ			0.09335 fts	97	0.5	3.48	⚠	
DX	702	709	0.07181 fts	89	1.2	-0.81		2.75
DY			0.07147 fts	89	1.2	-0.50		
DZ			0.07472 fts	89	1.2	2.71	⚠	
DX	702	705	0.05222 fts	87	1.3	-0.28		0.09
DY			0.05174 fts	87	1.3	0.41		
DZ			0.05633 fts	86	1.3	0.15		
DX	702	703	0.05585 fts	89	1.2	-0.40		0.16
DY			0.05539 fts	89	1.2	0.26		
DZ			0.05973 fts	88	1.2	0.51		
DX	708	734	0.04984 fts	77	1.8	-1.55		1.11
DY			0.04932 fts	77	1.8	0.35		
DZ			0.05434 fts	77	1.8	0.89		
DX	708	403	0.11688 fts	93	0.9	0.06		0.28
DY			0.11666 fts	93	0.9	0.31		
DZ			0.11886 fts	92	1.0	-0.87		
DX	551	741	0.08425 fts	77	1.9	0.38		0.11
DY			0.08374 fts	77	1.8	-0.08		
DZ			0.08858 fts	74	2.0	-0.43		
DX	551	734	0.13664 fts	96	0.7	0.60		1.29
DY			0.13643 fts	96	0.6	0.26		
DZ			0.13841 fts	95	0.7	-1.86		
DX	551	709	0.09129 fts	92	1.0	-0.63		0.23
DY			0.09101 fts	92	1.0	0.30		
DZ			0.09371 fts	91	1.0	-0.46		
DX	551	705	0.03987 fts	77	1.9	0.20		0.06
DY			0.03917 fts	77	1.9	0.01		
DZ			0.04553 fts	77	1.9	0.39		
DX	551	703	0.03772 fts	74	2.0	0.09		0.14
DY			0.03697 fts	74	2.0	-0.13		
DZ			0.04368 fts	74	2.0	0.64		
DX	551	403	0.08409 fts	83	1.5	0.87		2.71
DY			0.08375 fts	83	1.5	0.82		
DZ			0.08702 fts	83	1.5	-2.59	⚠	
DX	709	704	0.11121 fts	93	0.9	0.37		0.09
DY			0.11098 fts	93	0.9	0.26		
DZ			0.11331 fts	92	1.0	-0.27		
DX	1	704	0.13154 fts	96	0.6	0.14		0.14
DY			0.13134 fts	96	0.6	-0.30		
DZ			0.13330 fts	96	0.7	-0.56		
DX	1	701	0.08579 fts	74	2.0	-0.16		2.34
DY			0.08537 fts	74	2.0	0.85		
DZ			0.08951 fts	72	2.1	-2.50		
DX	552	704	0.07647 fts	91	1.0	0.36		0.09
DY			0.07614 fts	91	1.0	0.29		
DZ			0.07933 fts	91	1.1	-0.25		
DX	702	704	0.06679 fts	88	1.2	-0.76		0.33
DY			0.06641 fts	89	1.2	0.22		
DZ			0.07006 fts	88	1.2	0.59		
DX	551	704	0.04556 fts	73	2.0	-0.25		0.09
DY			0.04495 fts	73	2.1	-0.12		
DZ			0.05055 fts	74	2.0	0.44		

DX	551	1	0.11482 fts	97	0.5	-0.10	0.13
DY			0.11460 fts	97	0.5	0.55	
DZ			0.11672 fts	97	0.6	0.25	
DX	729	706	0.04965 fts	92	1.0	1.05	1.48
DY			0.04909 fts	93	0.9	0.49	
DZ			0.05465 fts	88	1.2	1.78	
DX	729	706	0.04965 fts	92	1.0	0.32	0.25
DY			0.04909 fts	93	0.9	-0.60	
DZ			0.05465 fts	88	1.2	0.54	
DX	1	552	0.09104 fts	98	0.4	-0.61	0.24
DY			0.09078 fts	98	0.4	-0.58	
DZ			0.09328 fts	98	0.5	-0.13	
DX	1	552	0.09104 fts	98	0.4	0.00	1.01
DY			0.09078 fts	98	0.4	-0.36	
DZ			0.09328 fts	98	0.5	-1.70	
DX	706	708	0.03609 fts	93	0.9	0.96	1.89
DY			0.03525 fts	94	0.8	-1.45	
DZ			0.04337 fts	84	1.5	-1.63	
DX	706	708	0.03609 fts	93	0.9	-0.44	2.12
DY			0.03525 fts	94	0.8	0.72	
DZ			0.04337 fts	84	1.5	2.38	
DX	552	708	0.09144 fts	99	0.2	0.60	0.18
DY			0.09121 fts	99	0.2	-0.36	
DZ			0.09347 fts	99	0.3	0.24	
DX	552	708	0.09144 fts	99	0.2	-0.15	0.44
DY			0.09121 fts	99	0.2	-0.25	
DZ			0.09347 fts	99	0.3	1.11	
DX	551	708	0.11055 fts	97	0.5	-0.01	0.02
DY			0.11034 fts	97	0.5	-0.08	
DZ			0.11241 fts	97	0.5	-0.24	
DX	551	708	0.11055 fts	97	0.5	0.06	0.21
DY			0.11034 fts	97	0.5	0.04	
DZ			0.11241 fts	97	0.5	-0.80	
DX	551	552	0.05628 fts	91	1.0	-0.16	0.11
DY			0.05585 fts	91	1.0	0.23	
DZ			0.05989 fts	91	1.1	0.49	
DX	551	552	0.05628 fts	91	1.0	0.32	0.63
DY			0.05585 fts	91	1.0	-0.46	
DZ			0.05989 fts	91	1.1	-1.26	
DX	705	401	0.03596 fts	77	1.8	0.24	0.10
DY			0.03522 fts	77	1.8	0.41	
DZ			0.04188 fts	78	1.8	-0.25	
DX	705	401	0.03596 fts	77	1.8	0.10	0.25
DY			0.03522 fts	77	1.8	-0.14	
DZ			0.04188 fts	78	1.8	-0.84	
DX	729	402	0.05132 fts	84	1.4	0.93	0.86
DY			0.05083 fts	85	1.4	-1.27	
DZ			0.05558 fts	84	1.5	0.33	
DX	729	402	0.05132 fts	84	1.4	-0.24	1.18
DY			0.05083 fts	85	1.4	1.84	
DZ			0.05558 fts	84	1.5	-0.31	
DX	709	401	0.08666 fts	91	1.0	0.62	2.42
DY			0.08638 fts	92	1.0	0.53	
DZ			0.08918 fts	91	1.0	-2.57	

	709	401	0.08666 fts	91	1.0	1.01		0.54	
DY			0.08638 fts	92	1.0	0.28			
DZ			0.08918 fts	91	1.0	-0.72			
DX	734	402	0.05163 fts	76	1.9	0.56		1.12	
DY			0.05112 fts	76	1.9	-1.74			
DZ			0.05609 fts	76	1.9	-0.18			
DX	734	402	0.05163 fts	76	1.9	-1.58		1.59	
DY			0.05112 fts	76	1.9	1.19			
DZ			0.05609 fts	76	1.9	0.93			
DX	1	402	0.04073 fts	75	2.0	0.78		2.61	
DY			0.04012 fts	75	2.0	-2.51			
DZ			0.04595 fts	76	1.9	0.95			
DX	1	402	0.04073 fts	75	2.0	-2.07		3.42	⚠
DY			0.04012 fts	75	2.0	1.86			
DZ			0.04595 fts	76	1.9	1.59			
DX	1	401	0.11173 fts	97	0.5	0.29		0.90	
DY			0.11151 fts	97	0.5	-1.22			
DZ			0.11369 fts	97	0.6	-1.07			
DX	1	401	0.11173 fts	97	0.5	0.50		0.59	
DY			0.11151 fts	97	0.5	-0.64			
DZ			0.11369 fts	97	0.6	-1.06			
DX	704	401	0.04844 fts	80	1.7	-0.77		0.45	
DY			0.04789 fts	80	1.7	-0.05			
DZ			0.05294 fts	80	1.7	0.86			
DX	704	401	0.04844 fts	80	1.7	-0.18		0.04	
DY			0.04789 fts	80	1.7	-0.08			
DZ			0.05294 fts	80	1.7	-0.28			
DX	552	729	0.09153 fts	98	0.5	0.33		1.35	
DY			0.09128 fts	98	0.5	-0.44			
DZ			0.09377 fts	97	0.5	1.94			
DX	552	729	0.09153 fts	98	0.5	1.01		0.89	
DY			0.09128 fts	98	0.5	0.45			
DZ			0.09377 fts	97	0.5	-1.20			
DX	552	402	0.09249 fts	95	0.7	1.54		5.86	⚠
DY			0.09221 fts	95	0.7	0.60			
DZ			0.09495 fts	94	0.8	-3.85	⚠		
DX	552	402	0.09249 fts	95	0.7	1.07		1.47	
DY			0.09221 fts	95	0.7	0.88			
DZ			0.09495 fts	94	0.8	-1.58			
DX	703	401	0.03538 fts	77	1.9	-0.14		0.02	
DY			0.03462 fts	77	1.9	0.00			
DZ			0.04140 fts	78	1.8	0.21			
DX	703	401	0.03538 fts	77	1.9	-0.07		0.00	
DY			0.03462 fts	77	1.9	-0.05			
DZ			0.04140 fts	78	1.8	0.00			
DX	702	401	0.04664 fts	88	1.2	-0.13		0.36	
DY			0.04613 fts	88	1.2	0.29			
DZ			0.05094 fts	88	1.2	0.98			
DX	702	401	0.04664 fts	88	1.2	0.12		0.23	
DY			0.04613 fts	88	1.2	0.45			
DZ			0.05094 fts	88	1.2	0.69			
DX	551	729	0.11902 fts	97	0.5	-0.22		0.23	
DY			0.11881 fts	97	0.5	0.00			

DZ			0.12085 fts	97	0.6	0.80	
DX	551	729	0.11902 fts	97	0.5	0.61	1.37
DY			0.11881 fts	97	0.5	0.39	
DZ			0.12085 fts	97	0.6	-1.89	
DX	551	401	0.03027 fts	73	2.0	-0.44	0.88
DY			0.02940 fts	73	2.1	-0.35	
DZ			0.03694 fts	76	1.9	1.52	
DX	551	401	0.03027 fts	73	2.0	0.08	0.10
DY			0.02940 fts	73	2.1	-0.21	
DZ			0.03694 fts	76	1.9	0.50	
DX	1	706	0.04093 fts	91	1.1	0.81	1.02
DY			0.04026 fts	91	1.0	0.98	
DZ			0.04681 fts	86	1.4	1.24	
DX	1	706	0.04093 fts	91	1.1	1.81	1.29
DY			0.04026 fts	91	1.0	0.18	
DZ			0.04681 fts	86	1.4	-0.68	
DX	1	706	0.04093 fts	91	1.1	0.16	0.10
DY			0.04026 fts	91	1.0	-0.42	
DZ			0.04681 fts	86	1.4	0.32	
DX	1	708	0.03567 fts	90	1.1	-0.69	0.71
DY			0.03504 fts	90	1.1	0.61	
DZ			0.04102 fts	90	1.1	1.12	
DX	1	708	0.03567 fts	90	1.1	0.66	0.58
DY			0.03504 fts	90	1.1	-0.93	
DZ			0.04102 fts	90	1.1	0.65	
DX	1	708	0.03567 fts	90	1.1	-0.63	2.12
DY			0.03504 fts	90	1.1	0.28	
DZ			0.04102 fts	90	1.1	2.43	
DX	1	708	0.03567 fts	90	1.1	1.55	0.85
DY			0.03504 fts	90	1.1	0.34	
DZ			0.04102 fts	90	1.1	0.19	
DX	729	708	0.04741 fts	93	0.9	-0.33	0.31
DY			0.04694 fts	93	0.9	0.71	
DZ			0.05154 fts	93	0.9	-0.57	
DX	729	708	0.04741 fts	93	0.9	0.80	0.43
DY			0.04694 fts	93	0.9	-0.28	
DZ			0.05154 fts	93	0.9	-0.77	
DX	729	708	0.04741 fts	93	0.9	-0.21	1.45
DY			0.04694 fts	93	0.9	0.22	
DZ			0.05154 fts	93	0.9	2.06	
DX	729	708	0.04741 fts	93	0.9	2.02	2.44
DY			0.04694 fts	93	0.9	-0.77	
DZ			0.05154 fts	93	0.9	-1.63	
DX	729	708	0.04741 fts	93	0.9	0.86	0.56
DY			0.04694 fts	93	0.9	-0.02	
DZ			0.05154 fts	93	0.9	0.98	
DX	1	729	0.03463 fts	89	1.2	-0.48	0.98
DY			0.03399 fts	89	1.2	-0.10	
DZ			0.04002 fts	90	1.1	1.65	
DX	1	729	0.03463 fts	89	1.2	0.60	1.77
DY			0.03399 fts	89	1.2	0.47	
DZ			0.04002 fts	90	1.1	-2.17	
DX	1	729	0.03463 fts	89	1.2	0.02	1.45
DY			0.03399 fts	89	1.2	-0.72	

			0.04002 fts	90	1.1	1.96	
**DX**	1	729	0.03463 fts	89	1.2	-0.28	0.03
**DY**			0.03399 fts	89	1.2	-0.04	
**DZ**			0.04002 fts	90	1.1	-0.15	
**DX**	1	729	0.03463 fts	89	1.2	0.73	1.39
**DY**			0.03399 fts	89	1.2	0.52	
**DZ**			0.04002 fts	90	1.1	-1.84	
**DX**	1	729	0.03463 fts	89	1.2	0.40	0.33
**DY**			0.03399 fts	89	1.2	0.48	
**DZ**			0.04002 fts	90	1.1	-0.76	

Redundancy:

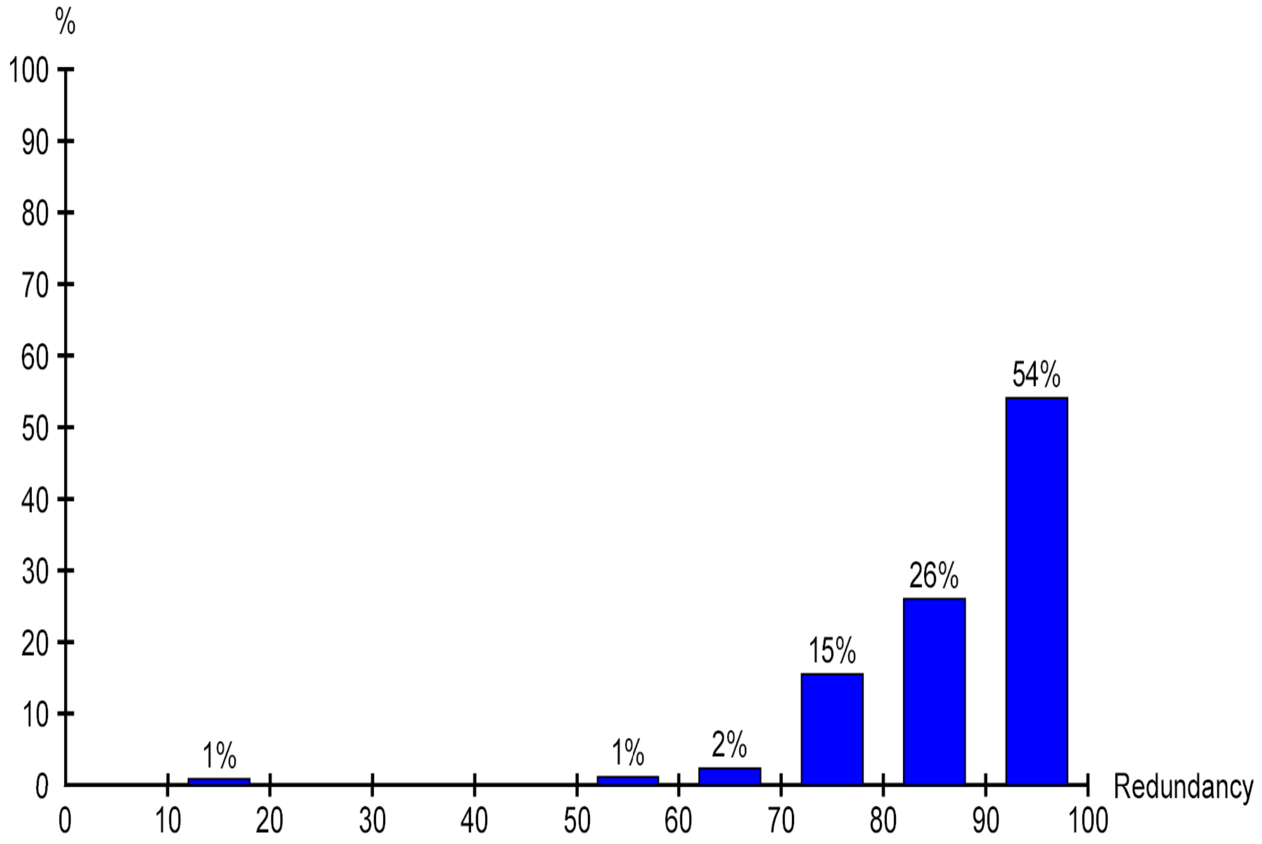

W-Test:

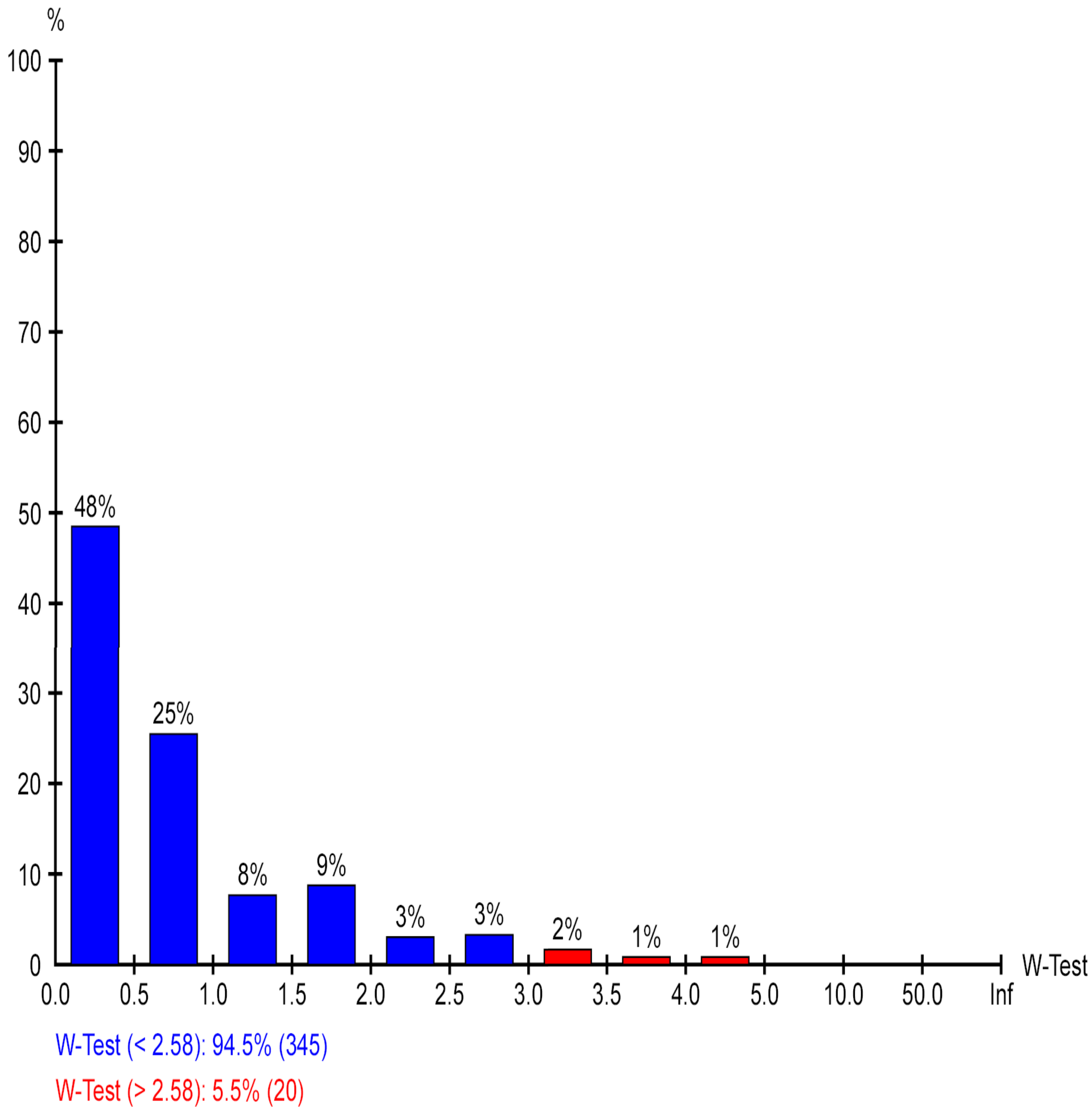

T-Test (2-dimensional):

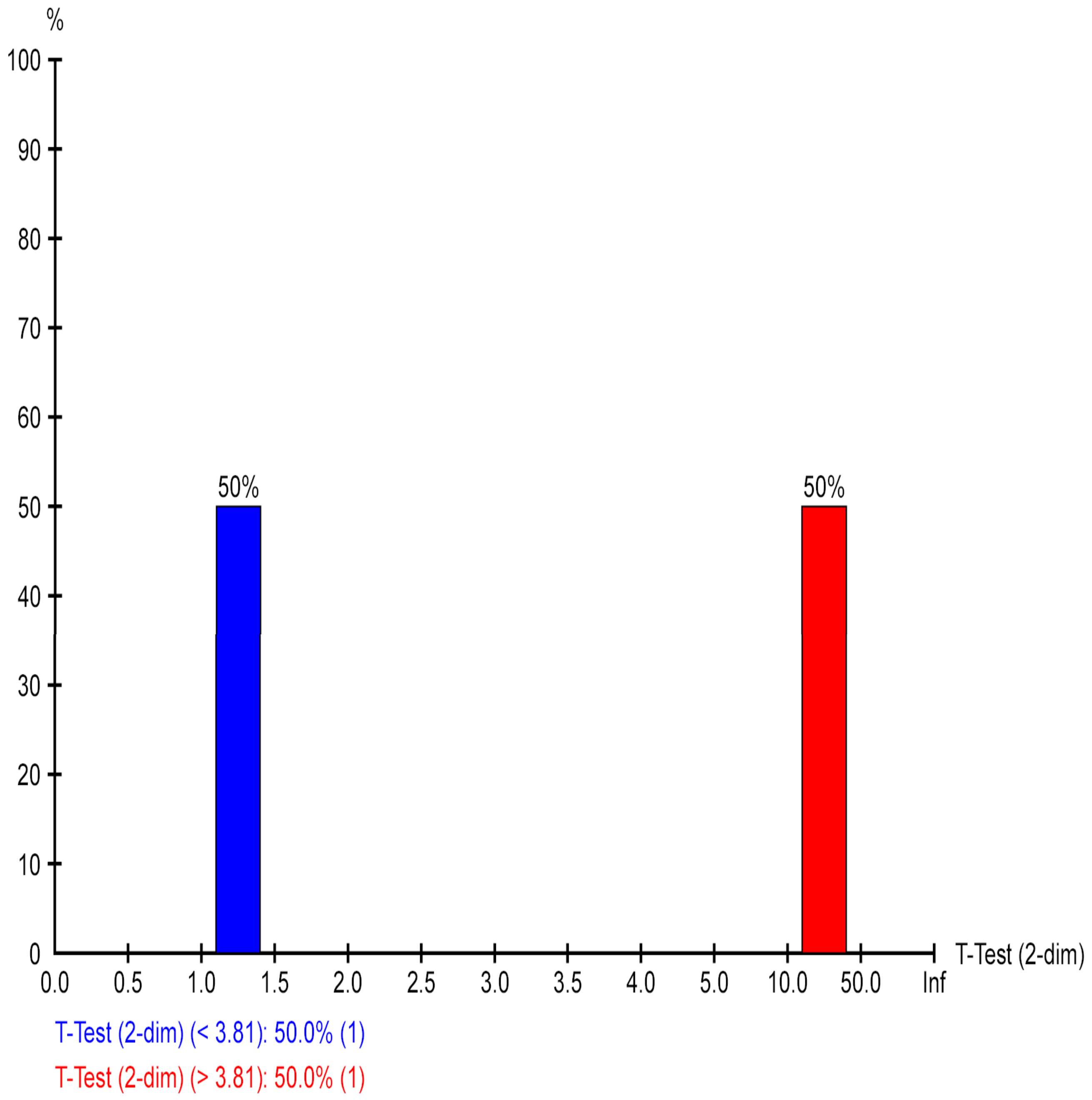

T-Test (3-dimensional):

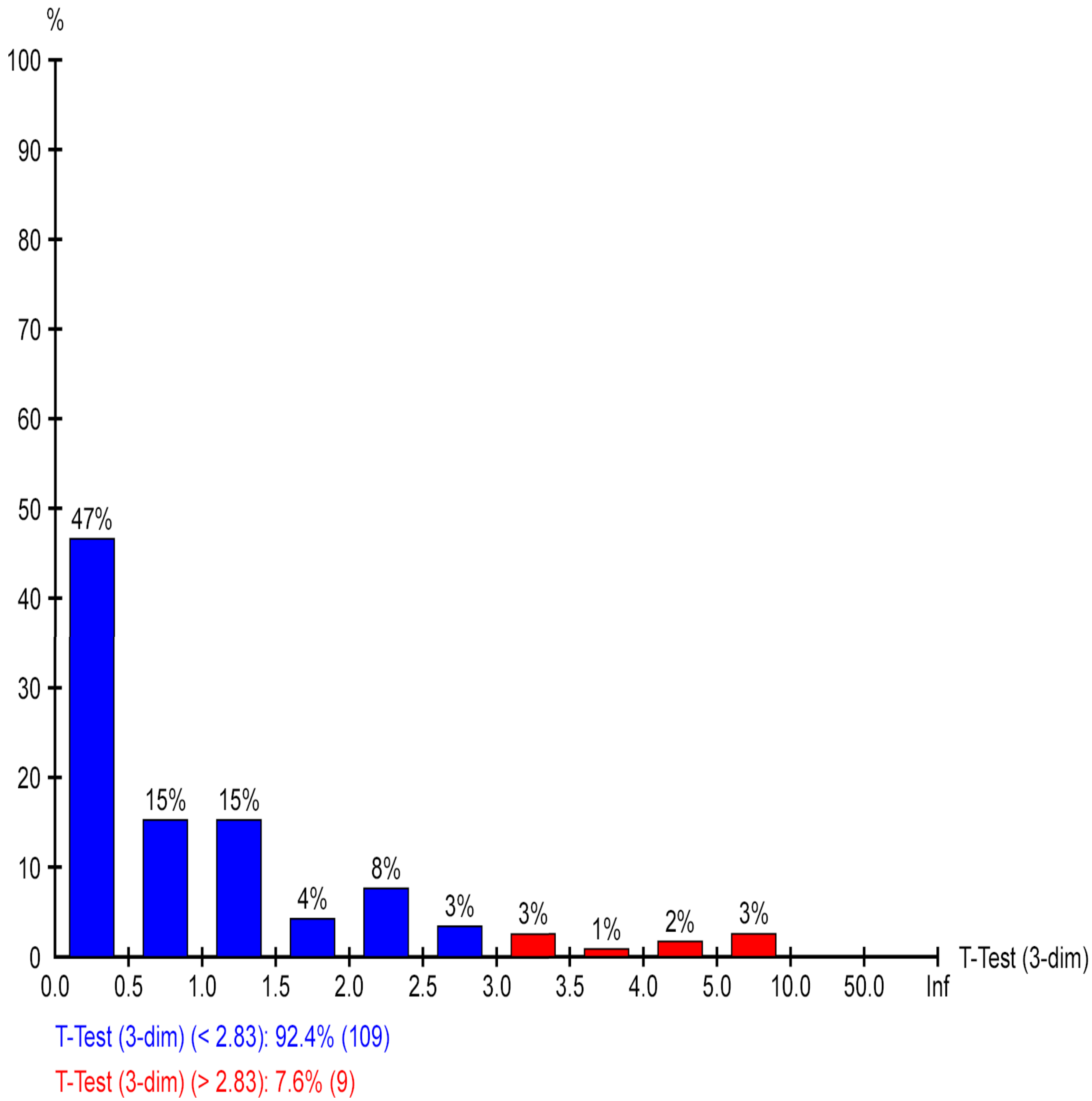

Estimated Errors (Coordinates)

Estimated Errors For Coordinates With Rejected W-Tests (max 10)

Station		W-Test	Fact	Est err
707	Longitude	4.37	1.7	0.02671 fts
702	Height	4.32	1.7	0.02679 fts
707	Latitude	3.80	1.5	0.02371 fts
708	Latitude	-3.56	1.4	-0.01484 fts

Estimated Errors For Stations With Rejected T-Tests (max 10)

Station		T-Test	Fact	Est err
702	Latitude	7.61	1.6	0.01058 fts
	Longitude			-0.00552 fts
	Height			0.02732 fts
707	Latitude	16.92	2.1	0.02394 fts
	Longitude			0.02690 fts
	Height			0.00000 fts
708	Latitude	8.14	1.7	-0.01526 fts
	Longitude			-0.01005 fts
	Height			-0.01460 fts

Estimated Errors (Observations)

Estimated Errors For Observations With Rejected W-Tests (max 10)

	Station	Target	W-Test	Fact	Est err
DZ	552	402	-3.85	1.5	-0.10699 fts
DZ	702	729	3.48	1.4	0.09513 fts
DZ	729	709	-3.24	1.3	-0.07118 fts
DX	708	707	-3.17	1.2	-0.03237 fts
DX	1	707	-3.03	1.2	-0.03866 fts
DZ	702	709	2.71	1.1	0.05923 fts
DZ	729	741	2.66	1.0	0.08972 fts
DZ	1	702	-2.62	1.0	-0.06816 fts
DZ	551	403	-2.59	1.0	-0.06590 fts

Estimated Errors For Observations With Rejected Antenna Hgt W-Tests (max 10)

Station	Target	W-Test	Fact	MDB [fts]	Est ant err [fts]
729	741	3.05	1.2	0.11455	0.10222
729	709	-2.74	1.1	0.07459	-0.05975
702	729	3.24	1.3	0.09297	0.08828
702	709	2.85	1.1	0.07419	0.06199
551	403	-2.84	1.1	0.08645	-0.07186
709	401	-2.67	1.0	0.08871	-0.06921
552	402	-4.17	1.6	0.09452	-0.11550

Estimated Errors For Observations With Rejected T-Tests (max 10)

	Station	Target	T-Test	Fact	Est err
DX	552	402	5.86	1.4	0.04180 fts
DY					0.01636 fts
DZ					-0.10704 fts
DX	702	729	4.21	1.2	0.00374 fts
DY					-0.01832 fts
DZ					0.09513 fts
DX	708	707	4.15	1.2	-0.03037 fts
DY					0.00081 fts
DZ					-0.02048 fts
DX	729	709	3.61	1.1	-0.01219 fts
DY					0.00400 fts
DZ					-0.07118 fts
DX	1	402	3.42	1.1	-0.02467 fts

DY					0.02181 fts
DZ					0.02142 fts
DX	1	707	3.38	1.1	-0.03791 fts
DY					0.00938 fts
DZ					-0.00982 fts
DX	729	741	3.35	1.1	-0.02604 fts
DY					-0.05003 fts
DZ					0.08973 fts
