# Supplementary material for: High-resolution prediction of American red squirrel in Interior Alaska: a role model for conservation using open access data, machine learning, GIS and LIDAR
Source: PeerJ. 2021 Sep 14;9:e11830. doi: 10.7717/peerj.11830 (PMC8447940; doi:10.7717/peerj.11830)
Supplement: Supplemental Information 26 — All metadata contents provided by the Alaska Division for Geographic and Geophysical Surveys for the used LIDAR dataset. [file peerj-09-11830-s026.zip › metadata/FNSB_LiDAR_Survey-_-Mapping-Narative_2010-11-02.pdf]

November 2, 2010
W.O. 60548

Mr. Jerry H. Zuspan
U.S. Department of the Army
Corps of Engineers, Pacific Ocean Division
Alaska District
P.O. Box 6898
Elmendorf Air Force Base, Alaska 99506-0898

Subject: Contract No. W911KB-07-D-0001, Delivery Order #0038
Fairbanks North Star Borough LiDAR Mapping Project

Dear Mr. Zuspan:

Attached, please find, DOWL HKM's Survey and Mapping Report for the Fairbanks North Star Borough LiDAR Mapping project in Fairbanks, Alaska. DOWL HKM performed check shot surveys and provided Light Detection and Ranging (LiDAR) mapping.

The work was done under DOWL HKM's Indefinite Delivery Contract with the Department of the Army, U.S. Army Engineers, Alaska District. This was Delivery Order No. 0038 under Contract No. W911KB-07-D-0001. The U.S. Army Corps of Engineers (USACE) Contracting Officer's Representative is Jerry H. Zuspan. I am DOWL HKM's Contract and Project Manager.

LiDAR mapping was performed by DOWL HKM's subconsultant, Aero-Metric, Inc., (Aero-Metric) of Anchorage, Alaska. Aero-Metric's Project Managers are John N. Ellis, CP, and Sean Bolender, and Senior Geodetic Surveyor, Jacques N. Cloutier. Mapping is based on LiDAR data acquired by Aero-Metric on May 4 and 6, 2010.

HORIZONTAL AND VERTICAL CONTROL

Coordinates are NAD83(2007.00) Alaska State Plane Coordinates, Zone 3, in US Feet. The Basis of Coordinates is the NAD83(2007.00) ASPC Zone 3 position of NGS Primary Airport Control Station "FAI A", PID DF3640 as retrieved from the NGS database on July 1, 2008, a 3-1/4" brass cap pipe monument, having coordinates of N=3,956,198.10967, E=1,351,803.19115 US Feet. Other horizontal positions shown here on were determined by differential static GPS observations using Leica dual frequency receivers during the period of July 2008 and May 2010.

The Project Combined Scale Factor is 0.99994797534. To convert ground distances to ASP grid distances, multiply by a combined scale factor of 0.99994797534. To convert ASP grid distances to ground distances, multiply by a combined scale factor of 100000000000/99994797534.

Elevations are NAVD88 in US Feet. The Basis of Elevations are USC&GS Bench Mark "G 121", PID TT2770, a 3-3/4" brass cap on stainless steel rod monument, having a value of 499.66 US Feet and USEO Bench Mark "DIKE 23 USE", PID TT2766, a brass cap having a value of 490.90 US Feet; as retrieved from the NGS database on July 1, 2008. Other elevations are derived from differential leveling using a Leica DNA10 digital level with fiberglass barcode rod,

or differential static GPS observations using Leica dual frequency GPS receivers. Elevations derived from differential static GPS observations or RTK GPS positioning were computed using GEOID06 orthometric heights, and adding a bias of 0.080 feet to the GEOID06 othrometric height to derive NAVD88 elevations.

A complete list of horizontal and vertical control points used to support the survey are provided in DOWL HKM's survey report.

LiDAR MAPPING

A bare-earth Digital Elevation Model (DEM) for this project was generated by Aero-Metric, Inc. of Anchorage, Alaska from Light Detection and Ranging (LiDAR) data acquired by them in early May, 2010. Said DEM is of a quality that meets or exceeds ASPRS Class II standards for the production of 2-foot contours in areas of moderate terrain and minimal vegetation. Areas with steep terrain or areas obscured by dense vegetation may be less accurate.

CHECK SHOT SURVEYS

DOWL HKM performed check shot surveys based on existing site control monuments, at various locations throughout the mapping limits in April and May of 2010. Check shot surveys were performed using Real Time Kinematic (RTK) GPS methods and appropriate quality assurance procedures. Check shot surveys result in the following accuracy statements applicable to the terrain and ground cover in the areas that were sampled.

The purpose of the surveys is to determine how well the LiDAR performed. Aero-Metric's accuracy statements are based on areas of moderate terrain and minimal vegetation. Diminished accuracies are to be expected in areas of extreme terrain and dense vegetation. The accuracy of each LiDAR point is expected to meet the vertical accuracy standard; however, derived products may be less accurate in extreme terrain and dense vegetation due to a lesser number of points defining the bare-earth in these areas. The project was planned to meet 0.33-foot accuracy within the mapping limits in flat, open areas.

DOWL HKM developed a point descriptor (cover code) system for denoting the type and density of ground cover in the project area. Ground cover is generally divided into the following categories:

1. Bare-earth and low grass (e.g., gravel roads, plowed fields, lawns).

2. High grass, weeds, and crops (e.g., hay fields, tundra).

3. Brush lands and low trees (e.g., willow brush, alder brush, berry brush).

4. Forested, fully covered by trees (e.g., deciduous, evergreens, mixed forests).

5. Urban areas (e.g., high, dense manmade structures).

A description of the cover codes defining the type and density of cover is provided in the check shot section of the report. Check shot surveys in a given area were designed so the shots would progress from bare-earth conditions into areas of dense cover. Test points were selected in

terrain that is flat or uniformly sloped within 5 meters in all directions. Test points were also selected in dense ground cover conditions where there is a reasonable expectation that LiDAR will reach the ground. The purpose of the check shot surveys is not to prove that LiDAR performs poorly on steep slopes and areas of extremely dense ground cover. The assumption being that LiDAR does not perform well in these areas.

DOWL HKM provided check shot point files to Aero-Metric after the LiDAR processing was complete. Aero-Metric processed the data and produced a check shot analysis, the results of which are detailed in their report. Generally, the results indicate that the LiDAR met the expected accuracy standard in the various types of ground cover.

LAS FILE EXAMINATION

DOWL HKM GIS personnel examined the LAS files received from Aero-Metric using LP360, which is a LiDAR data reader, within ArcGIS. Quality Assurance (QA) was performed to assure that not only did the data cover the full extent of the project area without any noticeable slivers, gaps, or anomalies, but that it also appeared accurate when overlaid on existing topographic data and orthoimagery. The data was scanned for completeness, continuity, lack of over-smoothing, and lack of artifacts and found to be acceptable.

Sincerely,
DOWL HKM

Stanley E. Ponsness, PLS, CFedS
Contract Manager

Attachments: LiDAR QA Summary as provided by Aero-Metric

D60548.Zuspan.SEP.110210.mas
