# Supplementary material for: High-resolution prediction of American red squirrel in Interior Alaska: a role model for conservation using open access data, machine learning, GIS and LIDAR
Source: PeerJ. 2021 Sep 14;9:e11830. doi: 10.7717/peerj.11830 (PMC8447940; doi:10.7717/peerj.11830)
Supplement: Supplemental Information 26 — All metadata contents provided by the Alaska Division for Geographic and Geophysical Surveys for the used LIDAR dataset. [file peerj-09-11830-s026.zip › metadata/Fairbanks_Lidar_QC_Analysis.pdf]

Analyzed on 9/13/2010 at 12:35:04 PM

Statistical Summary

Mean:	0.011	feet
Median:	0.005	feet
Mode:	0.006	feet
Kurtosis:	0.276	
Skew:	0.261	
Minimum:	-0.45	feet
Maximum:	0.5	feet
Range:	0.95	feet
Count:	4280	
RMSE:	0.128	
RMSE*1.96 (95% CI - NSSDA)	0.251	

Bin	*Frequency*
-0.45	0
-0.4	2
-0.35	3
-0.3	19
-0.25	37
-0.2	109
-0.15	226
-0.1	423
-0.05	571
0	686
0.05	685
0.1	545
0.15	391
0.2	245
0.25	173
0.3	84
0.35	45
0.4	22
0.45	11
0.5	3
**sum**	**4280**

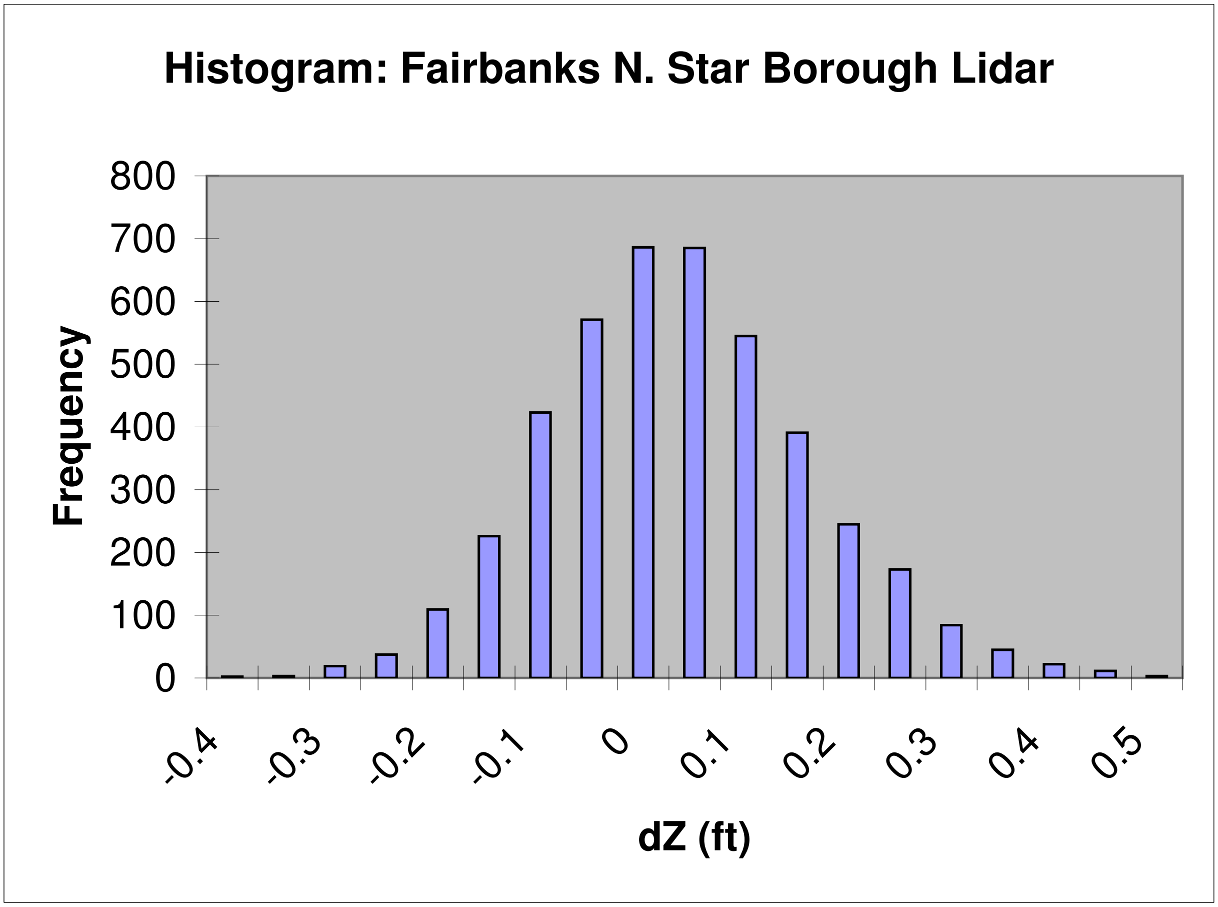

Fairbanks_North_Star_Borough\QAQC\Control\PENZ_PPK_Adjusted_ASPZ3_usft_G06.xyz

Number	Easting	Northing	Known Z	Laser Z	Dz	Dz^2
105936	1421440	3951995	475.693	475.28	-0.413	0.170569
103820	1373875	3957149	445.692	445.29	-0.402	0.161604
103808	1373798	3958258	445.12	444.73	-0.39	0.1521
103833	1373864	3956580	445.99	445.62	-0.37	0.1369
103795	1373728	3958823	445.317	444.95	-0.367	0.134689
100994	1334798	3948868	518.492	518.15	-0.342	0.116964
100947	1336107	3950317	433.048	432.71	-0.338	0.114244
101483	1348150	3965520	455.357	455.02	-0.337	0.113569
101164	1336571	3950775	432.784	432.45	-0.334	0.111556
104468	1373776	3952594	447.445	447.12	-0.325	0.105625
103781	1373640	3959384	443.601	443.28	-0.321	0.103041
100458	1348789	3966741	458.947	458.63	-0.317	0.100489
104474	1373781	3952782	447.656	447.34	-0.316	0.099856
101000	1334731	3948628	538.275	537.96	-0.315	0.099225
100819	1340533	3953671	432.94	432.63	-0.31	0.0961
106237	1421790	3970240	472.42	472.11	-0.31	0.0961
100931	1336594	3950821	432.838	432.53	-0.308	0.094864
103786	1373673	3959187	444.258	443.95	-0.308	0.094864
100154	1358223	3964121	435.197	434.89	-0.307	0.094249
103645	1374954	3964514	444.956	444.65	-0.306	0.093636
101515	1348959	3966969	459.593	459.29	-0.303	0.091809
100932	1336566	3950792	432.862	432.56	-0.302	0.091204
103357	1379815	3974783	494.162	493.86	-0.302	0.091204
104190	1372459	3947302	451.42	451.12	-0.3	0.09
104179	1372415	3947319	449.336	449.04	-0.296	0.087616
103359	1379776	3974694	491.621	491.33	-0.291	0.084681
104186	1372429	3947275	451.257	450.97	-0.287	0.082369
103787	1373679	3959148	444.284	444	-0.284	0.080656
103791	1373704	3958989	444.564	444.28	-0.284	0.080656
104210	1372623	3947573	450.463	450.18	-0.283	0.080089
103780	1373634	3959423	443.711	443.43	-0.281	0.078961
103355	1379857	3974870	496.881	496.6	-0.281	0.078961
100707	1343684	3957467	433.688	433.41	-0.278	0.077284
104185	1372419	3947275	451.158	450.88	-0.278	0.077284
100939	1336338	3950556	432.967	432.69	-0.277	0.076729
104178	1372426	3947323	449.566	449.29	-0.276	0.076176
103821	1373874	3957103	445.655	445.38	-0.275	0.075625
104472	1373780	3952727	447.764	447.49	-0.274	0.075076
101597	1352314	3969063	449.484	449.21	-0.274	0.075076
103452	1379599	3971436	449.484	449.21	-0.274	0.075076
107601	1396337	3957182	457.122	456.85	-0.272	0.073984
100987	1334945	3949112	494.142	493.87	-0.272	0.073984
106278	1421815	3971779	471.5	471.23	-0.27	0.0729
103796	1373732	3958780	445.408	445.14	-0.268	0.071824
103777	1373615	3959538	444.017	443.75	-0.267	0.071289
104174	1372470	3947352	450.347	450.08	-0.267	0.071289
101109	1334834	3948920	512.287	512.02	-0.267	0.071289
106267	1421805	3971224	470.046	469.78	-0.266	0.070756
104469	1373776	3952619	447.493	447.23	-0.263	0.069169
107002	1420630	3955001	475.453	475.19	-0.263	0.069169

106259	1421801	3970858	471.112	470.85	-0.262	0.068644
107661	1395442	3954138	458.421	458.16	-0.261	0.068121
101006	1334666	3948391	558.589	558.33	-0.259	0.067081
103776	1373609	3959575	444.038	443.78	-0.258	0.066564
105533	1429766	3934912	493.988	493.73	-0.258	0.066564
107636	1395848	3955519	457.806	457.55	-0.256	0.065536
106285	1421823	3972152	471.375	471.12	-0.255	0.065025
103667	1374854	3963666	447.104	446.85	-0.254	0.064516
103760	1373755	3960067	444.322	444.07	-0.252	0.063504
103822	1373873	3957058	445.402	445.15	-0.252	0.063504
107644	1395724	3955096	458.801	458.55	-0.251	0.063001
100609	1345670	3961015	436.989	436.74	-0.249	0.062001
104218	1372680	3947673	450.809	450.56	-0.249	0.062001
100455	1348854	3966858	459.199	458.95	-0.249	0.062001
101517	1349001	3967044	459.819	459.57	-0.249	0.062001
106946	1420725	3957601	474.959	474.71	-0.249	0.062001
100999	1334742	3948668	534.899	534.65	-0.249	0.062001
103843	1373852	3956180	445.788	445.54	-0.248	0.061504
103371	1379628	3974147	481.818	481.57	-0.248	0.061504
105657	1428183	3939838	488.698	488.45	-0.248	0.061504
104214	1372652	3947624	450.517	450.27	-0.247	0.061009
106299	1421838	3972903	475.016	474.77	-0.246	0.060516
100925	1336770	3951005	432.985	432.74	-0.245	0.060025
103915	1373773	3952859	447.545	447.3	-0.245	0.060025
103794	1373722	3958865	444.983	444.74	-0.243	0.059049
103804	1373772	3958435	445.222	444.98	-0.242	0.058564
101513	1348917	3966894	459.472	459.23	-0.242	0.058564
105633	1429044	3939091	490.272	490.03	-0.242	0.058564
103595	1375589	3967340	444.661	444.42	-0.241	0.058081
103793	1373717	3958907	444.901	444.66	-0.241	0.058081
101508	1348790	3966665	458.731	458.49	-0.241	0.058081
103828	1373869	3956792	445.49	445.25	-0.24	0.0576
100497	1347971	3965276	454.568	454.33	-0.238	0.056644
100530	1347254	3963994	440.666	440.43	-0.236	0.055696
100474	1348450	3966135	457.146	456.91	-0.236	0.055696
107014	1420374	3954262	474.475	474.24	-0.235	0.055225
103848	1373847	3955979	445.824	445.59	-0.234	0.054756
103805	1373778	3958391	445.023	444.79	-0.233	0.054289
103834	1373863	3956538	445.733	445.5	-0.233	0.054289
103746	1374016	3960415	445.352	445.12	-0.232	0.053824
107526	1398864	3959907	458.292	458.06	-0.232	0.053824
107682	1395023	3953094	463.502	463.27	-0.232	0.053824
100995	1334786	3948829	521.962	521.73	-0.232	0.053824
103381	1379604	3973691	470.661	470.43	-0.231	0.053361
103544	1377124	3968863	447.408	447.18	-0.228	0.051984
106751	1421793	3971481	469.718	469.49	-0.228	0.051984
100804	1340968	3954170	432.597	432.37	-0.227	0.051529
103774	1373597	3959648	444.107	443.88	-0.227	0.051529
101106	1334792	3948806	523.067	522.84	-0.227	0.051529
101096	1334674	3948371	560.377	560.15	-0.227	0.051529
103769	1373570	3959815	443.766	443.54	-0.226	0.051076
100722	1343324	3956904	433.145	432.92	-0.225	0.050625

103916	1373771	3952821	447.605	447.38	-0.225	0.050625
103383	1379604	3973600	468.875	468.65	-0.225	0.050625
106752	1421792	3971440	469.554	469.33	-0.224	0.050176
106728	1421814	3972633	473.974	473.75	-0.224	0.050176
100052	1357734	3960038	436.553	436.33	-0.223	0.049729
106773	1421774	3970447	472.163	471.94	-0.223	0.049729
101866	1356997	3974824	494.213	493.99	-0.223	0.049729
103815	1373876	3957380	446.562	446.34	-0.222	0.049284
106746	1421798	3971759	471.352	471.13	-0.222	0.049284
101790	1358371	3971862	441.681	441.46	-0.221	0.048841
101005	1334677	3948430	555.311	555.09	-0.221	0.048841
101518	1349022	3967081	459.95	459.73	-0.22	0.0484
106749	1421795	3971602	470.54	470.32	-0.22	0.0484
104202	1372563	3947469	450.709	450.49	-0.219	0.047961
105957	1421094	3953029	475.449	475.23	-0.219	0.047961
100983	1335055	3949227	479.809	479.59	-0.219	0.047961
107598	1396382	3957340	457.838	457.62	-0.218	0.047524
101300	1343797	3957641	434.207	433.99	-0.217	0.047089
101817	1358363	3973115	449.517	449.3	-0.217	0.047089
103593	1375641	3967410	443.436	443.22	-0.216	0.046656
101551	1349747	3968304	457.056	456.84	-0.216	0.046656
103382	1379604	3973646	469.645	469.43	-0.215	0.046225
103938	1373744	3951907	445.944	445.73	-0.214	0.045796
101590	1352000	3969072	450.674	450.46	-0.214	0.045796
100808	1340852	3954037	432.573	432.36	-0.213	0.045369
106793	1421251	3967867	468.663	468.45	-0.213	0.045369
106747	1421796	3971681	471.133	470.92	-0.213	0.045369
103660	1374915	3963945	446.112	445.9	-0.212	0.044944
103657	1374927	3964053	445.781	445.57	-0.211	0.044521
104409	1373737	3951090	446.261	446.05	-0.211	0.044521
104155	1372643	3947629	450.391	450.18	-0.211	0.044521
104195	1372496	3947359	450.921	450.71	-0.211	0.044521
100469	1348554	3966322	457.66	457.45	-0.21	0.0441
106741	1421803	3972000	471.54	471.33	-0.21	0.0441
105948	1421249	3952586	474.41	474.2	-0.21	0.0441
103782	1373647	3959345	443.699	443.49	-0.209	0.043681
104170	1372510	3947400	450.459	450.25	-0.209	0.043681
104216	1372666	3947649	450.649	450.44	-0.209	0.043681
106964	1420706	3956756	475.049	474.84	-0.209	0.043681
103810	1373812	3958172	445.518	445.31	-0.208	0.043264
103451	1379600	3971475	449.738	449.53	-0.208	0.043264
101548	1349670	3968205	457.618	457.41	-0.208	0.043264
101500	1348581	3966292	457.688	457.48	-0.208	0.043264
101480	1348064	3965367	454.767	454.56	-0.207	0.042849
103814	1373875	3957426	446.666	446.46	-0.206	0.042436
106764	1421781	3970855	471.046	470.84	-0.206	0.042436
103448	1379602	3971582	450.455	450.25	-0.205	0.042025
103920	1373766	3952676	447.724	447.52	-0.204	0.041616
103386	1379604	3973467	466.714	466.51	-0.204	0.041616
106739	1421805	3972081	471.524	471.32	-0.204	0.041616
100993	1334813	3948907	514.994	514.79	-0.204	0.041616
100484	1348244	3965765	456.233	456.03	-0.203	0.041209

101490	1348335	3965852	456.353	456.15	-0.203	0.041209
106858	1420880	3964685	470.433	470.23	-0.203	0.041209
106727	1421815	3972673	474.133	473.93	-0.203	0.041209
105950	1421223	3952668	474.453	474.25	-0.203	0.041209
107544	1398005	3959517	457.322	457.12	-0.202	0.040804
100981	1335109	3949284	472.992	472.79	-0.202	0.040804
100523	1347411	3964274	444.561	444.36	-0.201	0.040401
104213	1372645	3947611	450.481	450.28	-0.201	0.040401
100956	1335852	3950052	434.12	433.92	-0.2	0.04
101728	1358361	3969054	436.9	436.7	-0.2	0.04
101805	1358387	3972525	444.75	444.55	-0.2	0.04
103649	1374945	3964356	444.93	444.73	-0.2	0.04
103744	1374020	3960486	445.43	445.23	-0.2	0.04
103662	1374903	3963874	446.35	446.15	-0.2	0.04
104192	1372471	3947320	451.45	451.25	-0.2	0.04
103427	1379603	3971991	453.15	452.95	-0.2	0.04
103788	1373685	3959108	444.299	444.1	-0.199	0.039601
100365	1352615	3969067	447.149	446.95	-0.199	0.039601
104470	1373777	3952645	447.639	447.44	-0.199	0.039601
100389	1351566	3969111	451.699	451.5	-0.199	0.039601
100794	1341252	3954496	432.998	432.8	-0.198	0.039204
101729	1358362	3969085	436.608	436.41	-0.198	0.039204
103658	1374923	3964016	445.938	445.74	-0.198	0.039204
104212	1372637	3947598	450.438	450.24	-0.198	0.039204
100200	1358290	3966734	438.378	438.18	-0.197	0.038809
101815	1358382	3972979	448.677	448.48	-0.197	0.038809
101453	1347383	3964166	442.786	442.59	-0.196	0.038416
103918	1373769	3952747	447.786	447.59	-0.196	0.038416
104177	1372437	3947328	449.796	449.6	-0.196	0.038416
103790	1373698	3959029	444.445	444.25	-0.195	0.038025
103838	1373859	3956377	445.915	445.72	-0.195	0.038025
104450	1373758	3952027	446.125	445.93	-0.195	0.038025
104402	1373733	3950938	446.425	446.23	-0.195	0.038025
106934	1420736	3958157	474.215	474.02	-0.195	0.038025
105535	1429825	3935029	494.245	494.05	-0.195	0.038025
100241	1358344	3968528	436.974	436.78	-0.194	0.037636
105538	1429905	3935181	493.774	493.58	-0.194	0.037636
104475	1373782	3952812	447.613	447.42	-0.193	0.037249
104180	1372400	3947307	449.843	449.65	-0.193	0.037249
106049	1420735	3957226	474.663	474.47	-0.193	0.037249
107020	1420293	3954090	474.843	474.65	-0.193	0.037249
106723	1421820	3972871	474.953	474.76	-0.193	0.037249
101110	1334853	3948958	508.673	508.48	-0.193	0.037249
101726	1358360	3969000	437.142	436.95	-0.192	0.036864
103771	1373580	3959751	443.852	443.66	-0.192	0.036864
103688	1374490	3962925	448.522	448.33	-0.192	0.036864
100091	1358159	3961723	435.15	434.96	-0.19	0.0361
101135	1335638	3949811	436.031	435.84	-0.19	0.0361
101561	1350826	3969001	452.61	452.42	-0.19	0.0361
103379	1379604	3973783	472.87	472.68	-0.19	0.0361
105524	1429599	3934484	493.73	493.54	-0.19	0.0361
100936	1336452	3950674	432.919	432.73	-0.189	0.035721

103767	1373560	3959874	443.639	443.45	-0.189	0.035721
103758	1373810	3960088	444.249	444.06	-0.189	0.035721
105929	1421544	3951643	476.679	476.49	-0.189	0.035721
103588	1375739	3967538	442.779	442.59	-0.188	0.035344
103366	1379671	3974373	486.068	485.88	-0.188	0.035344
101296	1343617	3957326	433.387	433.2	-0.187	0.034969
103770	1373575	3959784	443.757	443.57	-0.187	0.034969
104427	1373744	3951466	446.627	446.44	-0.187	0.034969
104463	1373772	3952490	447.187	447	-0.187	0.034969
103473	1379347	3970560	451.077	450.89	-0.187	0.034969
101034	1333853	3947591	552.737	552.55	-0.187	0.034969
100536	1347126	3963757	440.536	440.35	-0.186	0.034596
104460	1373766	3952290	446.136	445.95	-0.186	0.034596
104176	1372448	3947334	450.036	449.85	-0.186	0.034596
107015	1420362	3954235	474.436	474.25	-0.186	0.034596
103376	1379606	3973920	476.516	476.33	-0.186	0.034596
103360	1379757	3974648	490.486	490.3	-0.186	0.034596
101100	1334719	3948535	546.176	545.99	-0.186	0.034596
101800	1358383	3972300	443.395	443.21	-0.185	0.034225
104462	1373772	3952473	447.035	446.85	-0.185	0.034225
100098	1358167	3962019	436.104	435.92	-0.184	0.033856
103653	1374937	3964202	445.404	445.22	-0.184	0.033856
101809	1358389	3972705	446.254	446.07	-0.184	0.033856
104152	1372669	3947676	450.624	450.44	-0.184	0.033856
103487	1378928	3970065	452.054	451.87	-0.184	0.033856
100466	1348616	3966433	458.004	457.82	-0.184	0.033856
106038	1420725	3956685	475.084	474.9	-0.184	0.033856
100218	1358311	3967533	435.453	435.27	-0.183	0.033489
100549	1346868	3963226	438.813	438.63	-0.183	0.033489
100358	1352917	3969063	444.753	444.57	-0.183	0.033489
103829	1373868	3956750	445.833	445.65	-0.183	0.033489
103889	1373803	3953915	447.263	447.08	-0.183	0.033489
101509	1348812	3966704	458.943	458.76	-0.183	0.033489
105707	1426016	3941353	486.053	485.87	-0.183	0.033489
105685	1427120	3940750	487.993	487.81	-0.183	0.033489
103358	1379795	3974739	492.983	492.8	-0.183	0.033489
100710	1343618	3957354	433.192	433.01	-0.182	0.033124
100231	1358325	3968106	438.252	438.07	-0.182	0.033124
106281	1421818	3971946	471.652	471.47	-0.182	0.033124
103373	1379617	3974056	479.592	479.41	-0.182	0.033124
105631	1429123	3939024	490.312	490.13	-0.182	0.033124
5527	1388588	4005429	737.802	737.62	-0.182	0.033124
101171	1336778	3950992	432.851	432.67	-0.181	0.032761
101172	1336808	3951023	432.931	432.75	-0.181	0.032761
101292	1343472	3957094	433.261	433.08	-0.181	0.032761
103465	1379498	3970893	450.291	450.11	-0.181	0.032761
103606	1375324	3966906	453.641	453.46	-0.181	0.032761
105529	1429679	3934720	493.121	492.94	-0.181	0.032761
100960	1335738	3949934	435.08	434.9	-0.18	0.0324
100537	1347106	3963717	440.37	440.19	-0.18	0.0324
101569	1351126	3969058	451.89	451.71	-0.18	0.0324
106249	1421811	3970564	471.75	471.57	-0.18	0.0324

106111	1420795	3960414	474.95	474.77	-0.18	0.0324
107003	1420615	3954931	475.4	475.22	-0.18	0.0324
100940	1336309	3950526	433.019	432.84	-0.179	0.032041
104405	1373735	3951006	446.249	446.07	-0.179	0.032041
107455	1402319	3961350	461.709	461.53	-0.179	0.032041
100618	1345490	3960699	436.878	436.7	-0.178	0.031684
103605	1375345	3966949	452.788	452.61	-0.178	0.031684
103608	1375285	3966820	455.438	455.26	-0.178	0.031684
100043	1357409	3959870	436.957	436.78	-0.177	0.031329
101446	1347183	3963807	440.137	439.96	-0.177	0.031329
103585	1375854	3967691	443.107	442.93	-0.177	0.031329
104175	1372459	3947342	450.247	450.07	-0.177	0.031329
101516	1348980	3967006	459.687	459.51	-0.177	0.031329
103393	1379602	3973166	462.697	462.52	-0.177	0.031329
106866	1420870	3964279	471.057	470.88	-0.177	0.031329
106730	1421813	3972554	473.817	473.64	-0.177	0.031329
106696	1421847	3974286	480.517	480.34	-0.177	0.031329
101044	1333375	3947313	540.817	540.64	-0.177	0.031329
100182	1358271	3965975	437.526	437.35	-0.176	0.030976
103789	1373692	3959068	444.426	444.25	-0.176	0.030976
100482	1348285	3965839	456.326	456.15	-0.176	0.030976
103812	1373825	3958086	445.755	445.58	-0.175	0.030625
104206	1372596	3947525	450.555	450.38	-0.175	0.030625
100438	1349219	3967512	459.975	459.8	-0.175	0.030625
107483	1400960	3960841	460.575	460.4	-0.175	0.030625
106742	1421802	3971960	471.675	471.5	-0.175	0.030625
100718	1343427	3957052	432.804	432.63	-0.174	0.030276
104217	1372673	3947661	450.704	450.53	-0.174	0.030276
105658	1428152	3939865	488.674	488.5	-0.174	0.030276
103852	1373843	3955825	445.933	445.76	-0.173	0.029929
103447	1379602	3971614	450.623	450.45	-0.173	0.029929
105548	1430027	3935413	493.503	493.33	-0.173	0.029929
103570	1376228	3968181	446.202	446.03	-0.172	0.029584
106066	1420750	3958099	474.262	474.09	-0.172	0.029584
100802	1341025	3954236	432.741	432.57	-0.171	0.029241
100239	1358343	3968445	437.011	436.84	-0.171	0.029241
106738	1421806	3972164	471.43	471.26	-0.171	0.029241
105981	1420375	3953932	475.411	475.24	-0.171	0.029241
103666	1374864	3963699	446.97	446.8	-0.17	0.0289
101816	1358376	3973024	449.03	448.86	-0.17	0.0289
100465	1348637	3966470	458.07	457.9	-0.17	0.0289
100413	1349820	3968455	458.5	458.33	-0.17	0.0289
106284	1421822	3972111	471.45	471.28	-0.17	0.0289
100989	1334894	3949050	500.88	500.71	-0.17	0.0289
101108	1334818	3948882	516.12	515.95	-0.17	0.0289
103803	1373766	3958480	445.489	445.32	-0.169	0.028561
103936	1373745	3951975	446.029	445.86	-0.169	0.028561
107624	1396026	3956125	457.129	456.96	-0.169	0.028561
106726	1421817	3972752	474.549	474.38	-0.169	0.028561
107019	1420303	3954110	474.689	474.52	-0.169	0.028561
101001	1334720	3948588	541.719	541.55	-0.169	0.028561
100128	1358206	3963252	435.338	435.17	-0.168	0.028224

103839	1373857	3956338	445.878	445.71	-0.168	0.028224
100374	1352208	3969080	450.018	449.85	-0.168	0.028224
105725	1424940	3941682	486.208	486.04	-0.168	0.028224
103665	1374873	3963733	446.807	446.64	-0.167	0.027889
100398	1351187	3969111	453.647	453.48	-0.167	0.027889
105973	1420651	3953706	475.547	475.38	-0.167	0.027889
103884	1373809	3954149	447.146	446.98	-0.166	0.027556
106152	1420907	3965299	470.456	470.29	-0.166	0.027556
105651	1428428	3939626	489.197	489.03	-0.166	0.027556
101398	1345925	3961436	436.945	436.78	-0.165	0.027225
101425	1346708	3962866	437.315	437.15	-0.165	0.027225
103594	1375615	3967376	443.995	443.83	-0.165	0.027225
100197	1358287	3966603	440.074	439.91	-0.164	0.026896
103654	1374935	3964164	445.484	445.32	-0.164	0.026896
103836	1373861	3956456	445.994	445.83	-0.164	0.026896
101573	1351286	3969072	451.144	450.98	-0.164	0.026896
101112	1334897	3949032	501.265	501.1	-0.164	0.026896
100139	1358212	3963566	434.783	434.62	-0.163	0.026569
100215	1358308	3967402	435.273	435.11	-0.163	0.026569
100357	1352960	3969062	444.553	444.39	-0.163	0.026569
103455	1379590	3971313	449.323	449.16	-0.163	0.026569
103377	1379605	3973874	475.293	475.13	-0.163	0.026569
100121	1358198	3963011	435.672	435.51	-0.162	0.026244
103754	1373921	3960167	444.252	444.09	-0.162	0.026244
103837	1373860	3956416	445.962	445.8	-0.162	0.026244
104411	1373738	3951132	446.382	446.22	-0.162	0.026244
104203	1372572	3947484	450.692	450.53	-0.162	0.026244
103482	1379102	3970230	452.052	451.89	-0.162	0.026244
106734	1421810	3972363	472.212	472.05	-0.162	0.026244
106731	1421812	3972477	473.122	472.96	-0.162	0.026244
100172	1358259	3965545	437.151	436.99	-0.161	0.025921
100199	1358289	3966690	438.871	438.71	-0.161	0.025921
100364	1352659	3969067	446.771	446.61	-0.161	0.025921
103387	1379604	3973424	466.071	465.91	-0.161	0.025921
106737	1421807	3972205	471.091	470.93	-0.161	0.025921
106743	1421801	3971879	471.581	471.42	-0.161	0.025921
107006	1420592	3954841	475.061	474.9	-0.161	0.025921
100998	1334753	3948708	531.551	531.39	-0.161	0.025921
106007	1420667	3955125	476	475.84	-0.16	0.0256
100997	1334764	3948748	528.28	528.12	-0.16	0.0256
100952	1335964	3950168	433.619	433.46	-0.159	0.025281
103623	1375003	3965446	446.429	446.27	-0.159	0.025281
103600	1375462	3967154	448.009	447.85	-0.159	0.025281
100986	1334972	3949141	490.579	490.42	-0.159	0.025281
100946	1336136	3950346	432.998	432.84	-0.158	0.024964
103650	1374943	3964317	445.168	445.01	-0.158	0.024964
104198	1372526	3947406	450.678	450.52	-0.158	0.024964
101157	1336367	3950562	432.917	432.76	-0.157	0.024649
103589	1375724	3967519	442.777	442.62	-0.157	0.024649
104156	1372634	3947613	450.297	450.14	-0.157	0.024649
104223	1372717	3947736	451.077	450.92	-0.157	0.024649
106005	1420654	3955051	475.887	475.73	-0.157	0.024649

103364	1379695	3974464	487.927	487.77	-0.157	0.024649
105521	1429558	3934343	493.707	493.55	-0.157	0.024649
104477	1373786	3952880	447.446	447.29	-0.156	0.024336
101093	1334598	3948162	571.716	571.56	-0.156	0.024336
100923	1336832	3951068	433.255	433.1	-0.155	0.024025
100206	1358297	3966999	436.035	435.88	-0.155	0.024025
100177	1358265	3965758	436.835	436.68	-0.155	0.024025
103965	1373728	3951110	446.245	446.09	-0.155	0.024025
101596	1352269	3969065	449.785	449.63	-0.155	0.024025
101847	1357624	3974186	480.125	479.97	-0.155	0.024025
101807	1358388	3972615	445.233	445.08	-0.154	0.023716
103557	1376650	3968556	445.934	445.78	-0.154	0.023716
104211	1372630	3947585	450.454	450.3	-0.154	0.023716
104194	1372487	3947345	451.034	450.88	-0.154	0.023716
106867	1420868	3964237	471.134	470.98	-0.154	0.023716
106250	1421811	3970581	471.824	471.67	-0.154	0.023716
105924	1421621	3951396	477.224	477.07	-0.154	0.023716
105628	1429201	3938956	490.524	490.37	-0.154	0.023716
103819	1373875	3957195	445.733	445.58	-0.153	0.023409
100404	1350946	3969076	454.523	454.37	-0.153	0.023409
106759	1421785	3971076	470.153	470	-0.153	0.023409
105738	1424321	3942076	486.423	486.27	-0.153	0.023409
104461	1373767	3952311	446.222	446.07	-0.152	0.023104
103378	1379604	3973829	473.992	473.84	-0.152	0.023104
100240	1358344	3968487	436.951	436.8	-0.151	0.022801
104447	1373755	3951941	446.011	445.86	-0.151	0.022801
101565	1350972	3969034	452.281	452.13	-0.151	0.022801
106052	1420739	3957385	474.791	474.64	-0.151	0.022801
101114	1334954	3949101	493.661	493.51	-0.151	0.022801
105532	1429747	3934874	493.691	493.54	-0.151	0.022801
103356	1379835	3974827	495.731	495.58	-0.151	0.022801
104204	1372580	3947498	450.65	450.5	-0.15	0.0225
103426	1379603	3972013	453.32	453.17	-0.15	0.0225
106735	1421808	3972285	471.29	471.14	-0.15	0.0225
100653	1344799	3959460	436.999	436.85	-0.149	0.022201
104453	1373760	3952111	446.029	445.88	-0.149	0.022201
104165	1372552	3947471	450.539	450.39	-0.149	0.022201
101494	1348439	3966039	456.859	456.71	-0.149	0.022201
107535	1398420	3959711	457.709	457.56	-0.149	0.022201
106244	1421806	3970464	471.949	471.8	-0.149	0.022201
107007	1420577	3954791	474.869	474.72	-0.149	0.022201
101115	1334985	3949134	489.719	489.57	-0.149	0.022201
101012	1334585	3948162	571.289	571.14	-0.149	0.022201
100602	1345817	3961277	436.848	436.7	-0.148	0.021904
103775	1373603	3959612	444.068	443.92	-0.148	0.021904
104473	1373780	3952754	447.708	447.56	-0.148	0.021904
104196	1372506	3947374	450.808	450.66	-0.148	0.021904
107653	1395569	3954568	458.458	458.31	-0.148	0.021904
105523	1429588	3934447	493.708	493.56	-0.148	0.021904
100670	1344438	3958814	436.858	436.71	-0.147	0.021609
101386	1345679	3960993	436.977	436.83	-0.147	0.021609
104428	1373745	3951487	446.568	446.42	-0.147	0.021609

103462	1379537	3971022	450.037	449.89	-0.147	0.021609
101506	1348748	3966588	458.547	458.4	-0.147	0.021609
105978	1420484	3953857	475.547	475.4	-0.147	0.021609
105647	1428578	3939495	489.557	489.41	-0.147	0.021609
100771	1341895	3955231	432.926	432.78	-0.146	0.021316
103717	1374048	3961578	447.126	446.98	-0.146	0.021316
104162	1372579	3947516	450.466	450.32	-0.146	0.021316
100426	1349478	3967981	459.606	459.46	-0.146	0.021316
101794	1358376	3972040	442.255	442.11	-0.145	0.021025
103755	1373895	3960144	444.245	444.1	-0.145	0.021025
103934	1373748	3952045	446.015	445.87	-0.145	0.021025
104200	1372545	3947439	450.705	450.56	-0.145	0.021025
105646	1428607	3939470	489.645	489.5	-0.145	0.021025
101865	1357020	3974784	493.835	493.69	-0.145	0.021025
101356	1345059	3959895	436.794	436.65	-0.144	0.020736
100185	1358274	3966102	438.894	438.75	-0.144	0.020736
103624	1375001	3965399	446.074	445.93	-0.144	0.020736
103692	1374396	3962760	448.754	448.61	-0.144	0.020736
104390	1373723	3950664	452.234	452.09	-0.144	0.020736
107665	1395381	3953933	459.174	459.03	-0.144	0.020736
100711	1343596	3957316	433.023	432.88	-0.143	0.020449
100556	1346734	3962977	437.323	437.18	-0.143	0.020449
101440	1347023	3963501	439.443	439.3	-0.143	0.020449
103747	1374013	3960381	445.123	444.98	-0.143	0.020449
103811	1373818	3958129	445.713	445.57	-0.143	0.020449
103435	1379601	3971886	452.433	452.29	-0.143	0.020449
106258	1421800	3970801	471.143	471	-0.143	0.020449
106959	1420712	3956974	474.993	474.85	-0.143	0.020449
105488	1429250	3933302	494.423	494.28	-0.143	0.020449
103801	1373755	3958568	445.952	445.81	-0.142	0.020164
103926	1373760	3952469	446.962	446.82	-0.142	0.020164
103524	1377790	3969294	447.752	447.61	-0.142	0.020164
104208	1372609	3947549	450.502	450.36	-0.142	0.020164
104189	1372453	3947294	451.082	450.94	-0.142	0.020164
101822	1358290	3973377	452.712	452.57	-0.142	0.020164
106191	1421180	3967712	470.132	469.99	-0.142	0.020164
106798	1421155	3967702	470.672	470.53	-0.142	0.020164
106736	1421807	3972245	471.312	471.17	-0.142	0.020164
106287	1421826	3972277	471.592	471.45	-0.142	0.020164
100992	1334831	3948944	511.412	511.27	-0.142	0.020164
100920	1336925	3951164	433.471	433.33	-0.141	0.019881
100917	1337023	3951257	433.491	433.35	-0.141	0.019881
101344	1344719	3959288	437.251	437.11	-0.141	0.019881
100532	1347209	3963914	440.731	440.59	-0.141	0.019881
103460	1379558	3971108	449.661	449.52	-0.141	0.019881
107546	1397887	3959465	457.521	457.38	-0.141	0.019881
101740	1358361	3969496	435.88	435.74	-0.14	0.0196
103799	1373745	3958654	445.91	445.77	-0.14	0.0196
101563	1350898	3969019	452.37	452.23	-0.14	0.0196
107668	1395344	3953807	459.48	459.34	-0.14	0.0196
100964	1335617	3949808	436.109	435.97	-0.139	0.019321
100965	1335586	3949776	436.689	436.55	-0.139	0.019321

103824	1373873	3956968	445.459	445.32	-0.139	0.019321
103542	1377196	3968910	447.369	447.23	-0.139	0.019321
101571	1351206	3969067	451.499	451.36	-0.139	0.019321
106335	1421876	3974849	484.289	484.15	-0.139	0.019321
105653	1428336	3939706	488.969	488.83	-0.139	0.019321
100955	1335880	3950081	433.978	433.84	-0.138	0.019044
103766	1373555	3959901	443.538	443.4	-0.138	0.019044
103846	1373849	3956059	445.768	445.63	-0.138	0.019044
103967	1373727	3951053	446.288	446.15	-0.138	0.019044
103681	1374642	3963192	448.888	448.75	-0.138	0.019044
104182	1372400	3947287	451.018	450.88	-0.138	0.019044
101491	1348355	3965889	456.478	456.34	-0.138	0.019044
106139	1420896	3964737	470.398	470.26	-0.138	0.019044
106805	1421039	3967452	470.818	470.68	-0.138	0.019044
106243	1421804	3970446	472.048	471.91	-0.138	0.019044
103365	1379683	3974419	487.088	486.95	-0.138	0.019044
105520	1429549	3934312	493.418	493.28	-0.138	0.019044
101004	1334688	3948469	551.988	551.85	-0.138	0.019044
101301	1343818	3957679	434.347	434.21	-0.137	0.018769
101787	1358365	3971719	441.107	440.97	-0.137	0.018769
101129	1335418	3949585	442.747	442.61	-0.137	0.018769
103797	1373736	3958738	445.647	445.51	-0.137	0.018769
104412	1373738	3951153	446.467	446.33	-0.137	0.018769
104466	1373774	3952549	447.407	447.27	-0.137	0.018769
104163	1372570	3947501	450.467	450.33	-0.137	0.018769
107614	1396164	3956590	456.197	456.06	-0.137	0.018769
107631	1395920	3955766	456.777	456.64	-0.137	0.018769
106273	1421810	3971524	469.937	469.8	-0.137	0.018769
106242	1421802	3970428	472.017	471.88	-0.137	0.018769
105742	1424171	3942242	486.038	485.9	-0.137	0.018769
103785	1373666	3959226	444.016	443.88	-0.136	0.018496
100977	1335223	3949400	459.196	459.06	-0.136	0.018496
106772	1421775	3970485	472.016	471.88	-0.136	0.018496
105482	1429186	3933183	494.556	494.42	-0.136	0.018496
101002	1334710	3948548	545.286	545.15	-0.136	0.018496
101792	1358373	3971952	442.045	441.91	-0.135	0.018225
103981	1373715	3950693	450.755	450.62	-0.135	0.018225
104188	1372447	3947286	451.055	450.92	-0.135	0.018225
101489	1348294	3965778	456.115	455.98	-0.135	0.018225
101511	1348876	3966818	459.205	459.07	-0.135	0.018225
101540	1349496	3967926	459.325	459.19	-0.135	0.018225
105942	1421353	3952264	475.185	475.05	-0.135	0.018225
105736	1424390	3942012	486.345	486.21	-0.135	0.018225
100810	1340794	3953970	432.824	432.69	-0.134	0.017956
100957	1335824	3950023	434.374	434.24	-0.134	0.017956
103849	1373846	3955940	445.824	445.69	-0.134	0.017956
105852	1423019	3947743	477.344	477.21	-0.134	0.017956
105638	1428887	3939227	489.924	489.79	-0.134	0.017956
105518	1429532	3934257	493.274	493.14	-0.134	0.017956
100938	1336396	3950615	432.953	432.82	-0.133	0.017689
100210	1358302	3967180	435.673	435.54	-0.133	0.017689
100157	1358226	3964241	435.833	435.7	-0.133	0.017689

101798	1358380	3972213	443.083	442.95	-0.133	0.017689
103733	1374031	3960906	445.803	445.67	-0.133	0.017689
104432	1373747	3951567	446.173	446.04	-0.133	0.017689
100366	1352571	3969068	447.474	447.34	-0.133	0.017689
105702	1426285	3941261	487.423	487.29	-0.133	0.017689
100644	1344998	3959817	436.812	436.68	-0.132	0.017424
101346	1344763	3959367	437.112	436.98	-0.132	0.017424
104416	1373740	3951237	446.712	446.58	-0.132	0.017424
104207	1372602	3947537	450.542	450.41	-0.132	0.017424
104183	1372405	3947281	451.242	451.11	-0.132	0.017424
100476	1348409	3966061	456.812	456.68	-0.132	0.017424
101512	1348897	3966856	459.342	459.21	-0.132	0.017424
106279	1421817	3971862	471.542	471.41	-0.132	0.017424
107011	1420542	3954668	475.022	474.89	-0.132	0.017424
101116	1335017	3949168	485.552	485.42	-0.132	0.017424
101127	1335357	3949522	447.161	447.03	-0.131	0.017161
103716	1374049	3961623	447.541	447.41	-0.131	0.017161
107600	1396360	3957261	457.361	457.23	-0.131	0.017161
106261	1421802	3970918	470.981	470.85	-0.131	0.017161
105992	1420362	3954197	474.291	474.16	-0.131	0.017161
107012	1420534	3954643	475.031	474.9	-0.131	0.017161
106722	1421822	3972951	475.211	475.08	-0.131	0.017161
105698	1426497	3941166	487.831	487.7	-0.131	0.017161
101166	1336629	3950837	432.65	432.52	-0.13	0.0169
101179	1337025	3951241	433.19	433.06	-0.13	0.0169
101290	1343344	3956909	433.45	433.32	-0.13	0.0169
100131	1358209	3963335	435.18	435.05	-0.13	0.0169
101730	1358362	3969117	436.48	436.35	-0.13	0.0169
103759	1373782	3960076	444.26	444.13	-0.13	0.0169
104504	1373826	3954242	447.2	447.07	-0.13	0.0169
103917	1373770	3952784	447.7	447.57	-0.13	0.0169
103510	1378102	3969502	450.02	449.89	-0.13	0.0169
100410	1350767	3969033	455.01	454.88	-0.13	0.0169
106265	1421803	3971096	470.221	470.09	-0.13	0.0169
107017	1420325	3954157	474.55	474.42	-0.13	0.0169
105967	1420840	3953475	475.34	475.21	-0.13	0.0169
105537	1429865	3935106	494.05	493.92	-0.13	0.0169
101617	1353196	3969030	445.049	444.92	-0.129	0.016641
107454	1402364	3961364	461.759	461.63	-0.129	0.016641
106750	1421794	3971562	470.109	469.98	-0.129	0.016641
106184	1421062	3967461	470.109	469.98	-0.129	0.016641
106231	1421784	3970046	471.799	471.67	-0.129	0.016641
106246	1421809	3970499	471.949	471.82	-0.129	0.016641
101422	1346631	3962719	436.898	436.77	-0.128	0.016384
101519	1349064	3967157	460.168	460.04	-0.128	0.016384
101836	1357958	3973899	467.098	466.97	-0.128	0.016384
106011	1420690	3955315	475.688	475.56	-0.128	0.016384
101013	1334566	3948128	572.108	571.98	-0.128	0.016384
101397	1345906	3961401	436.907	436.78	-0.127	0.016129
101788	1358367	3971767	441.257	441.13	-0.127	0.016129
103831	1373866	3956664	446.037	445.91	-0.127	0.016129
103540	1377265	3968954	447.417	447.29	-0.127	0.016129

106756	1421789	3971234	469.767	469.64	-0.127	0.016129
106277	1421815	3971737	471.427	471.3	-0.127	0.016129
101102	1334740	3948614	539.147	539.02	-0.127	0.016129
100107	1358177	3962417	436.756	436.63	-0.126	0.015876
103778	1373621	3959500	443.776	443.65	-0.126	0.015876
103932	1373750	3952116	446.056	445.93	-0.126	0.015876
100988	1334919	3949082	497.486	497.36	-0.126	0.015876
101165	1336600	3950806	432.695	432.57	-0.125	0.015625
100896	1337774	3951750	432.885	432.76	-0.125	0.015625
100751	1342491	3955912	433.215	433.09	-0.125	0.015625
100599	1345882	3961394	436.735	436.61	-0.125	0.015625
103582	1375910	3967765	443.365	443.24	-0.125	0.015625
103627	1374993	3965264	445.555	445.43	-0.125	0.015625
104181	1372398	3947297	450.495	450.37	-0.125	0.015625
101580	1351570	3969072	451.145	451.02	-0.125	0.015625
100412	1349849	3968485	458.435	458.31	-0.125	0.015625
106136	1420892	3964595	470.485	470.36	-0.125	0.015625
105630	1429149	3939001	490.315	490.19	-0.125	0.015625
101101	1334730	3948574	542.535	542.41	-0.125	0.015625
100137	1358212	3963499	434.934	434.81	-0.124	0.015376
100051	1357692	3960014	436.344	436.22	-0.124	0.015376
103573	1376141	3968073	445.184	445.06	-0.124	0.015376
103572	1376169	3968110	445.704	445.58	-0.124	0.015376
103637	1374971	3964842	445.914	445.79	-0.124	0.015376
104161	1372588	3947532	450.424	450.3	-0.124	0.015376
107570	1396881	3958715	459.954	459.83	-0.124	0.015376
106289	1421827	3972360	472.324	472.2	-0.124	0.015376
106729	1421814	3972593	474.004	473.88	-0.124	0.015376
105954	1421157	3952871	475.284	475.16	-0.124	0.015376
103370	1379635	3974192	482.704	482.58	-0.124	0.015376
100991	1334850	3948981	507.814	507.69	-0.124	0.015376
101142	1335860	3950042	434.093	433.97	-0.123	0.015129
101385	1345663	3960965	437.013	436.89	-0.123	0.015129
101784	1358361	3971580	440.573	440.45	-0.123	0.015129
101806	1358388	3972570	444.953	444.83	-0.123	0.015129
104408	1373737	3951069	446.263	446.14	-0.123	0.015129
101457	1347498	3964357	447.493	447.37	-0.123	0.015129
103459	1379566	3971150	449.523	449.4	-0.123	0.015129
103463	1379525	3970979	450.103	449.98	-0.123	0.015129
106770	1421778	3970600	471.843	471.72	-0.123	0.015129
106046	1420732	3957070	474.753	474.63	-0.123	0.015129
105941	1421366	3952225	475.273	475.15	-0.123	0.015129
105610	1429789	3938436	490.673	490.55	-0.123	0.015129
105451	1429154	3931906	495.383	495.26	-0.123	0.015129
101014	1334545	3948094	572.643	572.52	-0.123	0.015129
100721	1343350	3956941	433.082	432.96	-0.122	0.014884
100039	1357296	3959813	436.342	436.22	-0.122	0.014884
100041	1357365	3959848	436.742	436.62	-0.122	0.014884
103923	1373763	3952572	447.572	447.45	-0.122	0.014884
104471	1373778	3952672	447.672	447.55	-0.122	0.014884
101554	1349828	3968399	456.642	456.52	-0.122	0.014884
101111	1334874	3948996	504.902	504.78	-0.122	0.014884

101304	1343881	3957792	434.741	434.62	-0.121	0.014641
101759	1358381	3970599	439.601	439.48	-0.121	0.014641
103639	1374967	3964760	445.661	445.54	-0.121	0.014641
103464	1379512	3970936	450.171	450.05	-0.121	0.014641
105517	1429517	3934208	493.091	492.97	-0.121	0.014641
100719	1343402	3957015	432.88	432.76	-0.12	0.0144
101369	1345356	3960425	437.25	437.13	-0.12	0.0144
101452	1347338	3964088	441.72	441.6	-0.12	0.0144
103587	1375825	3967651	442.93	442.81	-0.12	0.0144
101608	1352806	3969045	445.66	445.54	-0.12	0.0144
101567	1351047	3969047	452.12	452	-0.12	0.0144
100459	1348767	3966702	458.84	458.72	-0.12	0.0144
100450	1348960	3967048	459.95	459.83	-0.12	0.0144
106753	1421792	3971399	469.591	469.47	-0.12	0.0144
101158	1336398	3950593	432.849	432.73	-0.119	0.014161
100933	1336537	3950762	432.919	432.8	-0.119	0.014161
100949	1336050	3950257	433.199	433.08	-0.119	0.014161
101735	1358364	3969295	435.829	435.71	-0.119	0.014161
101780	1358362	3971411	440.039	439.92	-0.119	0.014161
103726	1374038	3961199	446.109	445.99	-0.119	0.014161
104168	1372527	3947427	450.499	450.38	-0.119	0.014161
101510	1348854	3966780	459.079	458.96	-0.119	0.014161
106276	1421813	3971695	471.249	471.13	-0.119	0.014161
105543	1429963	3935293	493.489	493.37	-0.119	0.014161
101032	1333927	3947633	555.479	555.36	-0.119	0.014161
101336	1344548	3958982	436.718	436.6	-0.118	0.013924
100181	1358270	3965932	437.138	437.02	-0.118	0.013924
103571	1376198	3968146	446.058	445.94	-0.118	0.013924
101819	1358344	3973205	450.348	450.23	-0.118	0.013924
101484	1348171	3965557	455.538	455.42	-0.118	0.013924
101544	1349576	3968064	458.178	458.06	-0.118	0.013924
101537	1349443	3967831	459.368	459.25	-0.118	0.013924
106763	1421782	3970891	470.978	470.86	-0.118	0.013924
106953	1420718	3957275	474.878	474.76	-0.118	0.013924
101153	1336242	3950436	432.867	432.75	-0.117	0.013689
101802	1358385	3972390	443.837	443.72	-0.117	0.013689
103682	1374621	3963155	448.797	448.68	-0.117	0.013689
101526	1349236	3967465	460.097	459.98	-0.117	0.013689
105991	1420340	3954150	474.477	474.36	-0.117	0.013689
106954	1420717	3957238	474.917	474.8	-0.117	0.013689
106969	1420701	3956555	474.937	474.82	-0.117	0.013689
100953	1335936	3950139	433.746	433.63	-0.116	0.013456
101732	1358363	3969185	436.086	435.97	-0.116	0.013456
101347	1344785	3959406	437.006	436.89	-0.116	0.013456
101781	1358361	3971452	440.256	440.14	-0.116	0.013456
101449	1347270	3963967	440.626	440.51	-0.116	0.013456
104486	1373803	3953375	447.366	447.25	-0.116	0.013456
100368	1352482	3969070	448.176	448.06	-0.116	0.013456
100392	1351442	3969118	452.496	452.38	-0.116	0.013456
105993	1420373	3954220	474.296	474.18	-0.116	0.013456
105699	1426456	3941187	487.816	487.7	-0.116	0.013456
100926	1336740	3950973	432.865	432.75	-0.115	0.013225

100935	1336481	3950703	432.875	432.76	-0.115	0.013225
100950	1336021	3950227	433.305	433.19	-0.115	0.013225
100869	1338841	3952219	433.425	433.31	-0.115	0.013225
101359	1345125	3960012	436.845	436.73	-0.115	0.013225
101342	1344676	3959211	437.105	436.99	-0.115	0.013225
103768	1373565	3959845	443.715	443.6	-0.115	0.013225
103772	1373586	3959717	443.795	443.68	-0.115	0.013225
103718	1374047	3961534	446.735	446.62	-0.115	0.013225
104397	1373730	3950823	447.055	446.94	-0.115	0.013225
103708	1374094	3962044	449.335	449.22	-0.115	0.013225
103988	1373705	3950548	454.255	454.14	-0.115	0.013225
106760	1421785	3971038	470.215	470.1	-0.115	0.013225
106274	1421811	3971567	470.225	470.11	-0.115	0.013225
100217	1358310	3967489	435.464	435.35	-0.114	0.012996
101777	1358369	3971301	439.634	439.52	-0.114	0.012996
104445	1373754	3951882	445.984	445.87	-0.114	0.012996
104448	1373756	3951970	446.074	445.96	-0.114	0.012996
104485	1373802	3953330	447.104	446.99	-0.114	0.012996
100425	1349501	3968021	459.734	459.62	-0.114	0.012996
106766	1421780	3970784	471.124	471.01	-0.114	0.012996
105545	1429983	3935330	493.374	493.26	-0.114	0.012996
100990	1334871	3949016	504.334	504.22	-0.114	0.012996
100704	1343747	3957579	433.973	433.86	-0.113	0.012769
101454	1347406	3964205	443.623	443.51	-0.113	0.012769
103818	1373876	3957241	445.963	445.85	-0.113	0.012769
104465	1373773	3952528	447.143	447.03	-0.113	0.012769
101501	1348601	3966328	457.803	457.69	-0.113	0.012769
100461	1348723	3966624	458.603	458.49	-0.113	0.012769
100414	1349791	3968424	458.603	458.49	-0.113	0.012769
107016	1420350	3954209	474.423	474.31	-0.113	0.012769
106003	1420625	3954905	475.933	475.82	-0.113	0.012769
105549	1430041	3935439	493.503	493.39	-0.113	0.012769
100944	1336193	3950406	432.932	432.82	-0.112	0.012544
100079	1358146	3961207	435.932	435.82	-0.112	0.012544
101402	1346025	3961612	436.832	436.72	-0.112	0.012544
103802	1373761	3958524	445.682	445.57	-0.112	0.012544
103841	1373855	3956259	445.772	445.66	-0.112	0.012544
103887	1373806	3954009	447.112	447	-0.112	0.012544
103673	1374786	3963472	447.872	447.76	-0.112	0.012544
103493	1378703	3969902	452.092	451.98	-0.112	0.012544
101529	1349300	3967578	459.792	459.68	-0.112	0.012544
106061	1420746	3957850	474.582	474.47	-0.112	0.012544
105741	1424199	3942208	486.212	486.1	-0.112	0.012544
105460	1429122	3932291	495.052	494.94	-0.112	0.012544
101146	1335988	3950174	433.471	433.36	-0.111	0.012321
100156	1358225	3964200	435.521	435.41	-0.111	0.012321
101733	1358364	3969220	436.061	435.95	-0.111	0.012321
100234	1358332	3968235	437.951	437.84	-0.111	0.012321
100540	1347048	3963596	440.121	440.01	-0.111	0.012321
103590	1375706	3967497	442.611	442.5	-0.111	0.012321
103515	1377959	3969406	448.902	448.79	-0.111	0.012321
101507	1348769	3966627	458.601	458.49	-0.111	0.012321

106740	1421804	3972040	471.411	471.3	-0.111	0.012321
105925	1421608	3951437	477.121	477.01	-0.111	0.012321
105497	1429316	3933592	492.971	492.86	-0.111	0.012321
100086	1358154	3961512	435.02	434.91	-0.11	0.0121
103919	1373767	3952711	447.68	447.57	-0.11	0.0121
106325	1421865	3974286	480.5	480.39	-0.11	0.0121
100945	1336164	3950376	432.909	432.8	-0.109	0.011881
100963	1335648	3949840	435.739	435.63	-0.109	0.011881
100078	1358145	3961164	436.069	435.96	-0.109	0.011881
100633	1345212	3960208	436.749	436.64	-0.109	0.011881
100230	1358323	3968063	438.119	438.01	-0.109	0.011881
104484	1373800	3953238	446.719	446.61	-0.109	0.011881
103450	1379601	3971512	449.989	449.88	-0.109	0.011881
103367	1379661	3974327	485.069	484.96	-0.109	0.011881
101010	1334619	3948235	568.629	568.52	-0.109	0.011881
100553	1346785	3963073	438.058	437.95	-0.108	0.011664
101443	1347100	3963650	439.938	439.83	-0.108	0.011664
104197	1372516	3947389	450.738	450.63	-0.108	0.011664
107531	1398616	3959798	458.128	458.02	-0.108	0.011664
107555	1397470	3959258	458.768	458.66	-0.108	0.011664
106754	1421791	3971357	469.688	469.58	-0.108	0.011664
106840	1420897	3965621	470.868	470.76	-0.108	0.011664
105964	1420909	3953374	475.358	475.25	-0.108	0.011664
105751	1423924	3942655	485.098	484.99	-0.108	0.011664
101007	1334656	3948352	561.628	561.52	-0.108	0.011664
100954	1335908	3950110	433.917	433.81	-0.107	0.011449
103854	1373841	3955745	446.107	446	-0.107	0.011449
103715	1374051	3961667	448.027	447.92	-0.107	0.011449
100371	1352346	3969075	449.107	449	-0.107	0.011449
101830	1358124	3973701	459.527	459.42	-0.107	0.011449
101107	1334804	3948844	519.697	519.59	-0.107	0.011449
100893	1337888	3951800	432.676	432.57	-0.106	0.011236
101731	1358363	3969151	436.396	436.29	-0.106	0.011236
100598	1345904	3961432	436.897	436.79	-0.106	0.011236
101604	1352629	3969052	447.066	446.96	-0.106	0.011236
103619	1375014	3965639	448.496	448.39	-0.106	0.011236
104201	1372554	3947454	450.706	450.6	-0.106	0.011236
101575	1351366	3969073	451.036	450.93	-0.106	0.011236
103432	1379601	3971912	452.666	452.56	-0.106	0.011236
100462	1348701	3966585	458.536	458.43	-0.106	0.011236
107667	1395357	3953850	459.376	459.27	-0.106	0.011236
107567	1396980	3958841	459.786	459.68	-0.106	0.011236
103372	1379622	3974101	480.776	480.67	-0.106	0.011236
100924	1336801	3951036	433.065	432.96	-0.105	0.011025
100958	1335796	3949994	434.565	434.46	-0.105	0.011025
100068	1358134	3960726	436.865	436.76	-0.105	0.011025
100611	1345630	3960942	437.025	436.92	-0.105	0.011025
101744	1358365	3969977	437.925	437.82	-0.105	0.011025
103872	1373822	3954740	446.185	446.08	-0.105	0.011025
107618	1396106	3956396	456.615	456.51	-0.105	0.011025
101527	1349258	3967503	459.965	459.86	-0.105	0.011025
105963	1420955	3953299	475.485	475.38	-0.105	0.011025

104418	1373742	3951281	446.784	446.68	-0.104	0.010816
104169	1372519	3947414	450.464	450.36	-0.104	0.010816
103609	1375267	3966777	456.315	456.21	-0.104	0.010816
107543	1398043	3959535	457.534	457.43	-0.104	0.010816
107547	1397847	3959447	457.594	457.49	-0.104	0.010816
106142	1420899	3964880	470.484	470.38	-0.104	0.010816
106961	1420711	3956900	475.065	474.96	-0.104	0.010816
100081	1358148	3961294	435.533	435.43	-0.103	0.010609
101401	1346005	3961576	436.713	436.61	-0.103	0.010609
100201	1358291	3966778	437.823	437.72	-0.103	0.010609
104215	1372659	3947637	450.553	450.45	-0.103	0.010609
107622	1396049	3956203	457.103	457	-0.103	0.010609
106931	1420738	3958267	473.923	473.82	-0.103	0.010609
103369	1379642	3974237	483.503	483.4	-0.103	0.010609
104420	1373743	3951325	446.712	446.61	-0.102	0.010404
104154	1372651	3947644	450.482	450.38	-0.102	0.010404
105965	1420885	3953410	475.352	475.25	-0.102	0.010404
105750	1423942	3942615	485.232	485.13	-0.102	0.010404
100820	1340504	3953638	432.901	432.8	-0.101	0.010201
103971	1373725	3950936	446.531	446.43	-0.101	0.010201
104209	1372616	3947561	450.491	450.39	-0.101	0.010201
107625	1396004	3956045	456.851	456.75	-0.101	0.010201
100423	1349548	3968100	459.861	459.76	-0.101	0.010201
103397	1379602	3972997	461.051	460.95	-0.101	0.010201
106762	1421783	3970963	470.911	470.81	-0.101	0.010201
106313	1421852	3973634	477.971	477.87	-0.101	0.010201
105642	1428750	3939346	489.771	489.67	-0.101	0.010201
101868	1356938	3974945	494.991	494.89	-0.101	0.010201
100951	1335992	3950197	433.45	433.35	-0.1	0.01
100196	1358286	3966559	440.62	440.52	-0.1	0.01
103599	1375487	3967193	447.24	447.14	-0.1	0.01
103467	1379466	3970806	450.48	450.38	-0.1	0.01
107495	1400424	3960619	460.27	460.17	-0.1	0.01
107684	1394971	3953034	463.74	463.64	-0.1	0.01
106194	1421254	3967835	468.85	468.75	-0.1	0.01
106767	1421780	3970712	471.54	471.44	-0.1	0.01
106224	1421776	3969750	472.73	472.63	-0.1	0.01
105650	1428458	3939599	489.32	489.22	-0.1	0.01
105493	1429279	3933464	493.4	493.3	-0.1	0.01
105515	1429513	3934165	493.44	493.34	-0.1	0.01
105522	1429578	3934411	493.83	493.73	-0.1	0.01
100720	1343376	3956978	433.069	432.97	-0.099	0.009801
101766	1358388	3970889	438.469	438.37	-0.099	0.009801
103817	1373876	3957288	446.199	446.1	-0.099	0.009801
103960	1373732	3951250	446.819	446.72	-0.099	0.009801
103538	1377331	3968996	447.479	447.38	-0.099	0.009801
104158	1372616	3947581	450.279	450.18	-0.099	0.009801
103442	1379601	3971758	451.269	451.17	-0.099	0.009801
103611	1375050	3966018	456.979	456.88	-0.099	0.009801
107645	1395699	3955012	458.619	458.52	-0.099	0.009801
100429	1349412	3967862	459.299	459.2	-0.099	0.009801
101863	1357093	3974672	492.469	492.37	-0.099	0.009801

100942	1336251	3950466	432.928	432.83	-0.098	0.009604
101155	1336305	3950499	433.008	432.91	-0.098	0.009604
103630	1374987	3965135	445.928	445.83	-0.098	0.009604
103518	1377891	3969361	448.388	448.29	-0.098	0.009604
100460	1348745	3966663	458.678	458.58	-0.098	0.009604
106269	1421806	3971309	469.718	469.62	-0.098	0.009604
101191	1337503	3951589	432.577	432.48	-0.097	0.009409
100593	1346012	3961622	436.847	436.75	-0.097	0.009409
103784	1373660	3959265	443.867	443.77	-0.097	0.009409
103813	1373831	3958043	445.547	445.45	-0.097	0.009409
103840	1373856	3956298	445.847	445.75	-0.097	0.009409
104160	1372597	3947548	450.397	450.3	-0.097	0.009409
107651	1395609	3954705	458.207	458.11	-0.097	0.009409
100452	1348918	3966973	459.717	459.62	-0.097	0.009409
106143	1420899	3964915	470.467	470.37	-0.097	0.009409
106843	1420894	3965464	470.497	470.4	-0.097	0.009409
100982	1335082	3949255	476.397	476.3	-0.097	0.009409
105467	1429077	3932597	495.927	495.83	-0.097	0.009409
100220	1358313	3967623	435.886	435.79	-0.096	0.009216
101451	1347315	3964048	441.196	441.1	-0.096	0.009216
107619	1396095	3956357	456.716	456.62	-0.096	0.009216
100417	1349705	3968325	459.186	459.09	-0.096	0.009216
100431	1349369	3967783	459.286	459.19	-0.096	0.009216
106189	1421146	3967651	470.336	470.24	-0.096	0.009216
105540	1429937	3935242	493.576	493.48	-0.096	0.009216
103641	1374962	3964678	445.425	445.33	-0.095	0.009025
100361	1352790	3969065	445.645	445.55	-0.095	0.009025
103638	1374969	3964801	445.765	445.67	-0.095	0.009025
103553	1376796	3968650	446.325	446.23	-0.095	0.009025
100974	1335311	3949491	450.255	450.16	-0.095	0.009025
101553	1349801	3968367	456.765	456.67	-0.095	0.009025
100453	1348897	3966935	459.615	459.52	-0.095	0.009025
106744	1421800	3971839	471.515	471.42	-0.095	0.009025
106295	1421833	3972694	474.395	474.3	-0.095	0.009025
105994	1420384	3954243	474.445	474.35	-0.095	0.009025
100796	1341196	3954432	433.064	432.97	-0.094	0.008836
100918	1336990	3951227	433.494	433.4	-0.094	0.008836
100699	1343853	3957767	434.594	434.5	-0.094	0.008836
101765	1358388	3970851	438.574	438.48	-0.094	0.008836
101455	1347428	3964243	444.364	444.27	-0.094	0.008836
103629	1374989	3965178	445.794	445.7	-0.094	0.008836
103885	1373808	3954103	447.144	447.05	-0.094	0.008836
103607	1375304	3966863	454.554	454.46	-0.094	0.008836
103612	1375044	3965972	456.094	456	-0.094	0.008836
107525	1398906	3959926	458.424	458.33	-0.094	0.008836
105708	1425925	3941380	485.824	485.73	-0.094	0.008836
105743	1424145	3942277	485.914	485.82	-0.094	0.008836
105627	1429253	3938911	490.434	490.34	-0.094	0.008836
101860	1357175	3974569	491.114	491.02	-0.094	0.008836
105544	1429975	3935315	493.474	493.38	-0.094	0.008836
101008	1334644	3948313	564.364	564.27	-0.094	0.008836
100816	1340620	3953770	432.903	432.81	-0.093	0.008649

100622	1345414	3960567	436.833	436.74	-0.093	0.008649
103827	1373870	3956836	445.213	445.12	-0.093	0.008649
103955	1373734	3951390	446.683	446.59	-0.093	0.008649
104464	1373773	3952508	447.173	447.08	-0.093	0.008649
103680	1374662	3963227	448.683	448.59	-0.093	0.008649
100373	1352254	3969078	449.773	449.68	-0.093	0.008649
104172	1372491	3947375	450.423	450.33	-0.093	0.008649
106792	1421271	3967900	468.053	467.96	-0.093	0.008649
100996	1334775	3948789	525.153	525.06	-0.093	0.008649
100716	1343477	3957126	432.832	432.74	-0.092	0.008464
100161	1358230	3964406	436.872	436.78	-0.092	0.008464
103856	1373838	3955664	446.063	445.97	-0.092	0.008464
103664	1374881	3963768	446.712	446.62	-0.092	0.008464
103921	1373765	3952641	447.602	447.51	-0.092	0.008464
106106	1420789	3960163	474.942	474.85	-0.092	0.008464
105744	1424094	3942348	485.792	485.7	-0.092	0.008464
105527	1429648	3934641	493.202	493.11	-0.092	0.008464
105550	1430056	3935468	493.352	493.26	-0.092	0.008464
105554	1430144	3935637	493.862	493.77	-0.092	0.008464
101236	1340387	3953485	432.891	432.8	-0.091	0.008281
100037	1357273	3959802	436.231	436.14	-0.091	0.008281
100595	1345969	3961546	436.721	436.63	-0.091	0.008281
100534	1347167	3963835	440.641	440.55	-0.091	0.008281
101818	1358354	3973160	449.801	449.71	-0.091	0.008281
104166	1372544	3947456	450.541	450.45	-0.091	0.008281
103474	1379324	3970522	451.221	451.13	-0.091	0.008281
100449	1348981	3967086	460.061	459.97	-0.091	0.008281
105860	1422765	3948114	478.391	478.3	-0.091	0.008281
105749	1423961	3942575	485.231	485.14	-0.091	0.008281
105561	1430312	3935980	494.471	494.38	-0.091	0.008281
101103	1334751	3948652	535.821	535.73	-0.091	0.008281
101169	1336718	3950930	432.73	432.64	-0.09	0.0081
101456	1347451	3964281	445.3	445.21	-0.09	0.0081
103679	1374682	3963262	448.65	448.56	-0.09	0.0081
101833	1358044	3973805	462.81	462.72	-0.09	0.0081
106190	1421162	3967681	470.241	470.15	-0.09	0.0081
106133	1420889	3964489	470.45	470.36	-0.09	0.0081
106055	1420741	3957510	474.82	474.73	-0.09	0.0081
105975	1420598	3953760	475.55	475.46	-0.09	0.0081
101159	1336427	3950624	432.739	432.65	-0.089	0.007921
100697	1343894	3957841	434.869	434.78	-0.089	0.007921
103646	1374951	3964474	444.879	444.79	-0.089	0.007921
103743	1374021	3960522	445.489	445.4	-0.089	0.007921
103976	1373719	3950800	447.439	447.35	-0.089	0.007921
106932	1420737	3958230	474.109	474.02	-0.089	0.007921
105931	1421521	3951724	476.339	476.25	-0.089	0.007921
105446	1429148	3931683	496.059	495.97	-0.089	0.007921
105413	1428987	3930775	496.859	496.77	-0.089	0.007921
100040	1357347	3959839	436.678	436.59	-0.088	0.007744
100058	1357969	3960255	436.708	436.62	-0.088	0.007744
100664	1344562	3959036	436.788	436.7	-0.088	0.007744
100656	1344733	3959341	437.148	437.06	-0.088	0.007744

103576	1376057	3967961	444.228	444.14	-0.088	0.007744
103835	1373862	3956497	445.868	445.78	-0.088	0.007744
107506	1399873	3960371	459.048	458.96	-0.088	0.007744
100428	1349434	3967902	459.238	459.15	-0.088	0.007744
106905	1420762	3959578	475.168	475.08	-0.088	0.007744
5528	1388625	4005465	738.378	738.29	-0.088	0.007744
100233	1358329	3968192	438.037	437.95	-0.087	0.007569
100533	1347188	3963874	440.647	440.56	-0.087	0.007569
101450	1347293	3964008	440.757	440.67	-0.087	0.007569
107649	1395635	3954794	458.207	458.12	-0.087	0.007569
106260	1421802	3970888	471.047	470.96	-0.087	0.007569
106160	1420913	3965657	471.157	471.07	-0.087	0.007569
107013	1420525	3954618	475.007	474.92	-0.087	0.007569
105745	1424070	3942384	485.677	485.59	-0.087	0.007569
105541	1429947	3935262	493.617	493.53	-0.087	0.007569
101045	1333197	3947213	546.917	546.83	-0.087	0.007569
101392	1345793	3961198	436.876	436.79	-0.086	0.007396
101427	1346744	3962936	437.356	437.27	-0.086	0.007396
103597	1375537	3967268	445.926	445.84	-0.086	0.007396
103937	1373744	3951941	445.926	445.84	-0.086	0.007396
101574	1351326	3969073	451.076	450.99	-0.086	0.007396
101493	1348397	3965964	456.586	456.5	-0.086	0.007396
103392	1379602	3973208	463.156	463.07	-0.086	0.007396
106288	1421826	3972318	471.966	471.88	-0.086	0.007396
106733	1421811	3972401	472.446	472.36	-0.086	0.007396
100937	1336424	3950645	432.895	432.81	-0.085	0.007225
101293	1343496	3957132	433.275	433.19	-0.085	0.007225
103561	1376510	3968455	445.835	445.75	-0.085	0.007225
100399	1351144	3969107	453.785	453.7	-0.085	0.007225
103404	1379603	3972715	458.645	458.56	-0.085	0.007225
107664	1395405	3954016	458.995	458.91	-0.085	0.007225
105843	1423324	3947339	476.445	476.36	-0.085	0.007225
101419	1346445	3962371	437.194	437.11	-0.084	0.007056
101783	1358360	3971536	440.404	440.32	-0.084	0.007056
100363	1352703	3969066	446.334	446.25	-0.084	0.007056
103479	1379195	3970337	451.834	451.75	-0.084	0.007056
100408	1350819	3969047	454.934	454.85	-0.084	0.007056
106861	1420877	3964565	470.404	470.32	-0.084	0.007056
106903	1420763	3959656	475.254	475.17	-0.084	0.007056
105644	1428665	3939420	489.634	489.55	-0.084	0.007056
103361	1379740	3974602	489.814	489.73	-0.084	0.007056
100619	1345470	3960666	436.793	436.71	-0.083	0.006889
100669	1344459	3958851	436.833	436.75	-0.083	0.006889
104478	1373788	3952918	447.443	447.36	-0.083	0.006889
101587	1351869	3969070	450.833	450.75	-0.083	0.006889
107508	1399794	3960335	459.033	458.95	-0.083	0.006889
106293	1421831	3972569	474.013	473.93	-0.083	0.006889
5536	1388930	4005772	742.083	742	-0.083	0.006889
100133	1358211	3963385	435.162	435.08	-0.082	0.006724
100123	1358201	3963086	435.542	435.46	-0.082	0.006724
100205	1358296	3966954	436.252	436.17	-0.082	0.006724
101762	1358385	3970728	439.142	439.06	-0.082	0.006724

103845	1373850	3956099	445.832	445.75	-0.082	0.006724
103972	1373724	3950907	446.612	446.53	-0.082	0.006724
107603	1396315	3957105	456.722	456.64	-0.082	0.006724
100420	1349624	3968216	459.582	459.5	-0.082	0.006724
106266	1421804	3971138	470.112	470.03	-0.082	0.006724
106236	1421789	3970192	472.272	472.19	-0.082	0.006724
105956	1421111	3952990	475.522	475.44	-0.082	0.006724
105494	1429286	3933490	493.312	493.23	-0.082	0.006724
101021	1334341	3947880	570.922	570.84	-0.082	0.006724
100036	1357259	3959794	436.191	436.11	-0.081	0.006561
101424	1346671	3962794	436.941	436.86	-0.081	0.006561
103950	1373735	3951529	446.291	446.21	-0.081	0.006561
104479	1373790	3952960	447.361	447.28	-0.081	0.006561
103520	1377853	3969335	448.191	448.11	-0.081	0.006561
101504	1348683	3966474	458.071	457.99	-0.081	0.006561
107459	1402100	3961274	460.691	460.61	-0.081	0.006561
106268	1421805	3971267	469.831	469.75	-0.081	0.006561
105974	1420625	3953733	475.521	475.44	-0.081	0.006561
101003	1334699	3948509	548.651	548.57	-0.081	0.006561
100032	1357177	3959753	436.08	436	-0.08	0.0064
100581	1346275	3962093	436.91	436.83	-0.08	0.0064
100655	1344755	3959381	437.05	436.97	-0.08	0.0064
103761	1373729	3960061	444.15	444.07	-0.08	0.0064
103946	1373736	3951648	446.62	446.54	-0.08	0.0064
104499	1373819	3954003	447.17	447.09	-0.08	0.0064
103537	1377363	3969017	447.54	447.46	-0.08	0.0064
100394	1351357	3969119	453.13	453.05	-0.08	0.0064
100444	1349089	3967278	460.42	460.34	-0.08	0.0064
101011	1334603	3948198	570.13	570.05	-0.08	0.0064
101294	1343520	3957170	433.249	433.17	-0.079	0.006241
101343	1344698	3959249	437.189	437.11	-0.079	0.006241
103826	1373871	3956880	445.409	445.33	-0.079	0.006241
103857	1373837	3955623	446.079	446	-0.079	0.006241
101602	1352538	3969055	447.759	447.68	-0.079	0.006241
103497	1378553	3969804	451.869	451.79	-0.079	0.006241
103483	1379069	3970196	452.079	452	-0.079	0.006241
100400	1351102	3969103	453.99	453.91	-0.079	0.006241
101487	1348253	3965705	455.889	455.81	-0.079	0.006241
107660	1395466	3954220	458.269	458.19	-0.079	0.006241
107654	1395555	3954524	458.289	458.21	-0.079	0.006241
100454	1348875	3966897	459.449	459.37	-0.079	0.006241
106868	1420867	3964195	471.279	471.2	-0.079	0.006241
106235	1421788	3970167	472.169	472.09	-0.079	0.006241
106172	1420928	3966620	472.339	472.26	-0.079	0.006241
106298	1421836	3972819	474.719	474.64	-0.079	0.006241
5541	1389119	4005964	744.429	744.35	-0.079	0.006241
100227	1358320	3967932	437.718	437.64	-0.078	0.006084
103851	1373844	3955864	445.868	445.79	-0.078	0.006084
103546	1377052	3968816	447.418	447.34	-0.078	0.006084
103475	1379301	3970484	451.408	451.33	-0.078	0.006084
103476	1379276	3970447	451.568	451.49	-0.078	0.006084
107626	1395992	3956005	456.738	456.66	-0.078	0.006084

100442	1349133	3967356	460.508	460.43	-0.078	0.006084
106256	1421800	3970748	471.328	471.25	-0.078	0.006084
106950	1420722	3957423	474.948	474.87	-0.078	0.006084
105969	1420774	3953562	475.298	475.22	-0.078	0.006084
100104	1358173	3962284	436.647	436.57	-0.077	0.005929
100173	1358260	3965587	437.227	437.15	-0.077	0.005929
101778	1358366	3971335	439.577	439.5	-0.077	0.005929
103809	1373805	3958214	445.317	445.24	-0.077	0.005929
104415	1373740	3951216	446.668	446.59	-0.077	0.005929
104419	1373742	3951303	446.767	446.69	-0.077	0.005929
103697	1374282	3962553	450.477	450.4	-0.077	0.005929
103388	1379604	3973380	465.657	465.58	-0.077	0.005929
106816	1420930	3967005	471.947	471.87	-0.077	0.005929
106924	1420743	3958607	472.967	472.89	-0.077	0.005929
106051	1420737	3957305	474.707	474.63	-0.077	0.005929
107008	1420570	3954767	474.857	474.78	-0.077	0.005929
101095	1334663	3948329	563.637	563.56	-0.077	0.005929
100120	1358197	3962971	435.716	435.64	-0.076	0.005776
100629	1345289	3960343	436.816	436.74	-0.076	0.005776
100668	1344479	3958888	436.876	436.8	-0.076	0.005776
100539	1347067	3963637	440.156	440.08	-0.076	0.005776
104441	1373752	3951768	446.456	446.38	-0.076	0.005776
103963	1373730	3951166	446.506	446.43	-0.076	0.005776
103616	1375023	3965785	452.236	452.16	-0.076	0.005776
101525	1349215	3967426	460.186	460.11	-0.076	0.005776
105656	1428214	3939812	488.676	488.6	-0.076	0.005776
105534	1429805	3934990	494.246	494.17	-0.076	0.005776
5531	1388740	4005581	739.446	739.37	-0.076	0.005776
100871	1338754	3952181	433.215	433.14	-0.075	0.005625
104459	1373766	3952268	446.055	445.98	-0.075	0.005625
104157	1372625	3947598	450.305	450.23	-0.075	0.005625
103495	1378627	3969852	451.955	451.88	-0.075	0.005625
105614	1429642	3938573	490.045	489.97	-0.075	0.005625
105547	1430003	3935367	493.495	493.42	-0.075	0.005625
105440	1429143	3931388	495.915	495.84	-0.075	0.005625
101025	1334188	3947784	564.965	564.89	-0.075	0.005625
100053	1357776	3960065	436.644	436.57	-0.074	0.005476
100666	1344521	3958962	436.824	436.75	-0.074	0.005476
101789	1358369	3971815	441.494	441.42	-0.074	0.005476
103875	1373821	3954603	446.484	446.41	-0.074	0.005476
104417	1373741	3951259	446.734	446.66	-0.074	0.005476
103457	1379580	3971232	449.384	449.31	-0.074	0.005476
107457	1402186	3961305	461.084	461.01	-0.074	0.005476
105652	1428366	3939679	489.024	488.95	-0.074	0.005476
100930	1336623	3950851	432.713	432.64	-0.073	0.005329
100744	1342699	3956150	433.023	432.95	-0.073	0.005329
101375	1345475	3960634	436.953	436.88	-0.073	0.005329
100657	1344711	3959302	437.233	437.16	-0.073	0.005329
103699	1374240	3962467	450.563	450.49	-0.073	0.005329
104384	1373719	3950548	454.013	453.94	-0.073	0.005329
100448	1349002	3967123	460.113	460.04	-0.073	0.005329
103391	1379602	3973251	463.703	463.63	-0.073	0.005329

106280	1421817	3971904	471.483	471.41	-0.073	0.005329
107005	1420599	3954868	475.163	475.09	-0.073	0.005329
105747	1424024	3942460	485.383	485.31	-0.073	0.005329
105542	1429960	3935287	493.534	493.46	-0.073	0.005329
100793	1341280	3954527	433.012	432.94	-0.072	0.005184
100170	1358241	3964823	437.462	437.39	-0.072	0.005184
103748	1374009	3960347	444.972	444.9	-0.072	0.005184
103714	1374053	3961713	448.472	448.4	-0.072	0.005184
100470	1348534	3966285	457.592	457.52	-0.072	0.005184
107595	1396426	3957504	458.262	458.19	-0.072	0.005184
103385	1379604	3973512	467.422	467.35	-0.072	0.005184
106226	1421780	3969929	472.552	472.48	-0.072	0.005184
106885	1420848	3963333	473.852	473.78	-0.072	0.005184
105706	1426062	3941339	486.392	486.32	-0.072	0.005184
101104	1334761	3948691	532.482	532.41	-0.072	0.005184
101040	1333555	3947418	542.192	542.12	-0.072	0.005184
103734	1374030	3960865	445.741	445.67	-0.071	0.005041
101503	1348663	3966437	457.971	457.9	-0.071	0.005041
103384	1379604	3973556	468.141	468.07	-0.071	0.005041
106871	1420863	3964025	471.951	471.88	-0.071	0.005041
105727	1424855	3941717	486.231	486.16	-0.071	0.005041
101859	1357236	3974506	489.921	489.85	-0.071	0.005041
101077	1334040	3947685	559.441	559.37	-0.071	0.005041
101091	1334528	3948050	573.711	573.64	-0.071	0.005041
101178	1336993	3951210	433.08	433.01	-0.07	0.0049
100919	1336958	3951195	433.43	433.36	-0.07	0.0049
101396	1345886	3961367	436.96	436.89	-0.07	0.0049
103626	1374996	3965308	445.72	445.65	-0.07	0.0049
103700	1374220	3962422	450.36	450.29	-0.07	0.0049
103472	1379369	3970599	450.99	450.92	-0.07	0.0049
101568	1351086	3969053	452	451.93	-0.07	0.0049
100976	1335252	3949430	455.97	455.9	-0.07	0.0049
100485	1348223	3965728	456.25	456.18	-0.07	0.0049
100415	1349762	3968392	458.82	458.75	-0.07	0.0049
106757	1421788	3971194	469.95	469.88	-0.07	0.0049
106297	1421835	3972777	474.44	474.37	-0.07	0.0049
106044	1420731	3956992	474.81	474.74	-0.07	0.0049
101117	1335049	3949201	481.45	481.38	-0.07	0.0049
105719	1425315	3941564	485.54	485.47	-0.07	0.0049
105491	1429258	3933391	493.68	493.61	-0.07	0.0049
105448	1429151	3931758	495.84	495.77	-0.07	0.0049
100746	1342640	3956082	433.069	433	-0.069	0.004761
100150	1358219	3963971	435.059	434.99	-0.069	0.004761
101415	1346342	3962179	436.889	436.82	-0.069	0.004761
100560	1346685	3962883	437.079	437.01	-0.069	0.004761
100966	1335555	3949744	437.299	437.23	-0.069	0.004761
100543	1346989	3963472	439.679	439.61	-0.069	0.004761
104171	1372500	3947387	450.429	450.36	-0.069	0.004761
104164	1372561	3947485	450.449	450.38	-0.069	0.004761
104153	1372660	3947660	450.539	450.47	-0.069	0.004761
107616	1396141	3956513	456.229	456.16	-0.069	0.004761
107473	1401404	3961009	460.219	460.15	-0.069	0.004761

106153	1420908	3965370	470.449	470.38	-0.069	0.004761
106241	1421800	3970409	471.919	471.85	-0.069	0.004761
105946	1421287	3952464	474.729	474.66	-0.069	0.004761
106951	1420720	3957349	474.849	474.78	-0.069	0.004761
105970	1420751	3953591	475.339	475.27	-0.069	0.004761
101844	1357715	3974111	476.659	476.59	-0.069	0.004761
105526	1429634	3934601	493.429	493.36	-0.069	0.004761
105514	1429516	3934145	493.609	493.54	-0.069	0.004761
101009	1334632	3948274	566.729	566.66	-0.069	0.004761
100922	1336863	3951100	433.368	433.3	-0.068	0.004624
100088	1358156	3961598	434.988	434.92	-0.068	0.004624
100596	1345947	3961509	436.828	436.76	-0.068	0.004624
101753	1358374	3970348	440.108	440.04	-0.068	0.004624
103596	1375563	3967304	445.348	445.28	-0.068	0.004624
103975	1373720	3950826	447.058	446.99	-0.068	0.004624
100378	1352028	3969087	450.548	450.48	-0.068	0.004624
103494	1378665	3969877	452.058	451.99	-0.068	0.004624
107540	1398195	3959606	457.578	457.51	-0.068	0.004624
107548	1397808	3959429	457.608	457.54	-0.068	0.004624
107646	1395686	3954969	458.438	458.37	-0.068	0.004624
100421	1349598	3968178	459.558	459.49	-0.068	0.004624
106797	1421173	3967736	470.458	470.39	-0.068	0.004624
106774	1421773	3970408	472.128	472.06	-0.068	0.004624
101056	1333318	3947265	542.418	542.35	-0.068	0.004624
100589	1346098	3961777	436.547	436.48	-0.067	0.004489
101334	1344506	3958906	436.807	436.74	-0.067	0.004489
100575	1346394	3962309	437.058	436.99	-0.067	0.004489
100554	1346766	3963039	437.867	437.8	-0.067	0.004489
101786	1358364	3971672	440.927	440.86	-0.067	0.004489
103579	1375978	3967856	443.887	443.82	-0.067	0.004489
103850	1373845	3955902	445.877	445.81	-0.067	0.004489
103558	1376615	3968532	445.988	445.92	-0.067	0.004489
104421	1373744	3951346	446.657	446.59	-0.067	0.004489
101599	1352403	3969060	448.707	448.64	-0.067	0.004489
101560	1350792	3968992	452.807	452.74	-0.067	0.004489
104373	1373707	3950332	453.547	453.48	-0.067	0.004489
103390	1379603	3973294	464.348	464.28	-0.067	0.004489
106177	1420934	3966835	471.777	471.71	-0.067	0.004489
105552	1430107	3935565	493.657	493.59	-0.067	0.004489
5538	1389005	4005848	743.057	742.99	-0.067	0.004489
101239	1340469	3953577	432.806	432.74	-0.066	0.004356
100713	1343550	3957240	432.966	432.9	-0.066	0.004356
101352	1344922	3959651	436.936	436.87	-0.066	0.004356
101801	1358384	3972345	443.606	443.54	-0.066	0.004356
103735	1374029	3960825	445.726	445.66	-0.066	0.004356
103713	1374055	3961758	448.796	448.73	-0.066	0.004356
104199	1372536	3947422	450.656	450.59	-0.066	0.004356
100402	1351021	3969090	454.346	454.28	-0.066	0.004356
101556	1349886	3968460	456.356	456.29	-0.066	0.004356
101499	1348561	3966256	457.516	457.45	-0.066	0.004356
106844	1420894	3965423	470.356	470.29	-0.066	0.004356
106253	1421804	3970656	471.646	471.58	-0.066	0.004356

106330	1421871	3974549	482.056	481.99	-0.066	0.004356
105681	1427292	3940605	487.716	487.65	-0.066	0.004356
100214	1358307	3967358	435.315	435.25	-0.065	0.004225
101399	1345945	3961470	436.885	436.82	-0.065	0.004225
100649	1344890	3959621	436.965	436.9	-0.065	0.004225
103844	1373851	3956139	445.805	445.74	-0.065	0.004225
103798	1373741	3958696	445.815	445.75	-0.065	0.004225
103913	1373776	3952936	447.425	447.36	-0.065	0.004225
103675	1374755	3963403	447.955	447.89	-0.065	0.004225
101813	1358388	3972888	447.985	447.92	-0.065	0.004225
101814	1358385	3972933	448.385	448.32	-0.065	0.004225
101583	1351697	3969070	450.935	450.87	-0.065	0.004225
103485	1379000	3970128	452.255	452.19	-0.065	0.004225
107608	1396250	3956883	456.415	456.35	-0.065	0.004225
107643	1395736	3955138	458.795	458.73	-0.065	0.004225
101523	1349171	3967349	460.425	460.36	-0.065	0.004225
106058	1420743	3957682	474.675	474.61	-0.065	0.004225
106942	1420729	3957782	474.775	474.71	-0.065	0.004225
101042	1333482	3947374	540.735	540.67	-0.065	0.004225
101152	1336210	3950404	432.964	432.9	-0.064	0.004096
101405	1346107	3961758	436.524	436.46	-0.064	0.004096
103484	1379035	3970162	452.204	452.14	-0.064	0.004096
107524	1398988	3959964	458.394	458.33	-0.064	0.004096
107593	1396448	3957588	458.574	458.51	-0.064	0.004096
100962	1335679	3949871	435.513	435.45	-0.063	0.003969
101393	1345811	3961231	436.843	436.78	-0.063	0.003969
101796	1358378	3972127	442.703	442.64	-0.063	0.003969
103750	1373994	3960281	444.553	444.49	-0.063	0.003969
104454	1373761	3952138	446.113	446.05	-0.063	0.003969
103517	1377912	3969375	448.433	448.37	-0.063	0.003969
103696	1374304	3962595	450.363	450.3	-0.063	0.003969
104205	1372588	3947512	450.573	450.51	-0.063	0.003969
103471	1379390	3970639	450.913	450.85	-0.063	0.003969
101564	1350935	3969027	452.283	452.22	-0.063	0.003969
100403	1350983	3969083	454.443	454.38	-0.063	0.003969
100405	1350911	3969069	454.633	454.57	-0.063	0.003969
106138	1420895	3964701	470.383	470.32	-0.063	0.003969
103374	1379612	3974011	478.493	478.43	-0.063	0.003969
105704	1426153	3941309	487.043	486.98	-0.063	0.003969
105528	1429663	3934680	493.123	493.06	-0.063	0.003969
5532	1388779	4005620	739.993	739.93	-0.063	0.003969
100921	1336894	3951132	433.412	433.35	-0.062	0.003844
100122	1358200	3963049	435.622	435.56	-0.062	0.003844
100605	1345754	3961163	436.812	436.75	-0.062	0.003844
100786	1341474	3954748	432.801	432.74	-0.061	0.003721
101249	1341211	3954427	433.051	432.99	-0.061	0.003721
104440	1373751	3951742	446.501	446.44	-0.061	0.003721
103503	1378344	3969666	451.341	451.28	-0.061	0.003721
103481	1379134	3970265	451.971	451.91	-0.061	0.003721
101821	1358306	3973335	452.291	452.23	-0.061	0.003721
103436	1379602	3971874	452.351	452.29	-0.061	0.003721
101827	1358196	3973584	456.001	455.94	-0.061	0.003721

100479	1348346	3965951	456.561	456.5	-0.061	0.003721
100477	1348388	3966024	456.761	456.7	-0.061	0.003721
107640	1395785	3955307	458.181	458.12	-0.061	0.003721
105668	1427758	3940208	488.161	488.1	-0.061	0.003721
105639	1428860	3939250	489.961	489.9	-0.061	0.003721
5548	1389381	4006229	748.271	748.21	-0.061	0.003721
100916	1337056	3951287	433.54	433.48	-0.06	0.0036
100703	1343768	3957617	434.091	434.03	-0.06	0.0036
103969	1373727	3950995	446.37	446.31	-0.06	0.0036
100379	1351986	3969088	450.64	450.58	-0.06	0.0036
101591	1352044	3969072	450.66	450.6	-0.06	0.0036
103428	1379602	3971970	453	452.94	-0.06	0.0036
100401	1351061	3969097	454.17	454.11	-0.06	0.0036
101485	1348192	3965594	455.57	455.51	-0.06	0.0036
107602	1396326	3957144	456.92	456.86	-0.06	0.0036
100472	1348492	3966210	457.41	457.35	-0.06	0.0036
101541	1349515	3967959	459.2	459.14	-0.06	0.0036
106270	1421807	3971352	469.82	469.76	-0.06	0.0036
106803	1421070	3967527	470.93	470.87	-0.06	0.0036
106001	1420608	3954836	475.63	475.57	-0.06	0.0036
101105	1334782	3948768	526.21	526.15	-0.06	0.0036
100824	1340390	3953507	432.839	432.78	-0.059	0.003481
101149	1336084	3950274	433.079	433.02	-0.059	0.003481
100948	1336078	3950287	433.119	433.06	-0.059	0.003481
101442	1347080	3963612	439.979	439.92	-0.059	0.003481
103636	1374973	3964883	445.989	445.93	-0.059	0.003481
103466	1379483	3970850	450.339	450.28	-0.059	0.003481
101566	1351009	3969041	452.149	452.09	-0.059	0.003481
101478	1348021	3965289	454.559	454.5	-0.059	0.003481
100978	1335194	3949370	462.529	462.47	-0.059	0.003481
101851	1357499	3974288	483.449	483.39	-0.059	0.003481
105714	1425600	3941480	485.549	485.49	-0.059	0.003481
105513	1429518	3934126	493.739	493.68	-0.059	0.003481
101305	1343901	3957829	434.908	434.85	-0.058	0.003364
101739	1358362	3969455	435.888	435.83	-0.058	0.003364
103962	1373731	3951194	446.528	446.47	-0.058	0.003364
104414	1373739	3951195	446.608	446.55	-0.058	0.003364
101820	1358332	3973249	450.968	450.91	-0.058	0.003364
106132	1420888	3964418	470.589	470.53	-0.058	0.003364
106919	1420749	3958836	472.818	472.76	-0.058	0.003364
106904	1420762	3959617	475.368	475.31	-0.058	0.003364
105870	1422515	3948601	478.468	478.41	-0.058	0.003364
105678	1427419	3940500	487.658	487.6	-0.058	0.003364
100941	1336280	3950496	432.937	432.88	-0.057	0.003249
100152	1358221	3964044	435.087	435.03	-0.057	0.003249
101428	1346762	3962970	437.557	437.5	-0.057	0.003249
100548	1346889	3963266	438.877	438.82	-0.057	0.003249
101131	1335481	3949649	439.257	439.2	-0.057	0.003249
101782	1358360	3971494	440.317	440.26	-0.057	0.003249
101130	1335449	3949617	440.947	440.89	-0.057	0.003249
100430	1349390	3967823	459.227	459.17	-0.057	0.003249
106794	1421231	3967834	469.207	469.15	-0.057	0.003249

106245	1421808	3970481	471.967	471.91	-0.057	0.003249
106318	1421857	3973891	478.637	478.58	-0.057	0.003249
105748	1423981	3942537	485.217	485.16	-0.057	0.003249
105701	1426328	3941244	487.567	487.51	-0.057	0.003249
105649	1428489	3939573	489.357	489.3	-0.057	0.003249
103362	1379724	3974555	489.357	489.3	-0.057	0.003249
105637	1428914	3939204	489.967	489.91	-0.057	0.003249
101143	1335892	3950074	433.956	433.9	-0.056	0.003136
100129	1358207	3963281	435.276	435.22	-0.056	0.003136
100638	1345117	3960034	436.816	436.76	-0.056	0.003136
100583	1346231	3962015	436.866	436.81	-0.056	0.003136
100970	1335433	3949617	441.546	441.49	-0.056	0.003136
101803	1358386	3972435	444.066	444.01	-0.056	0.003136
103656	1374930	3964090	445.696	445.64	-0.056	0.003136
103855	1373839	3955704	446.086	446.03	-0.056	0.003136
104455	1373762	3952165	446.116	446.06	-0.056	0.003136
104407	1373736	3951049	446.206	446.15	-0.056	0.003136
101593	1352134	3969069	450.586	450.53	-0.056	0.003136
103433	1379601	3971903	452.596	452.54	-0.056	0.003136
100427	1349456	3967942	459.516	459.46	-0.056	0.003136
106745	1421799	3971799	471.406	471.35	-0.056	0.003136
105869	1422533	3948559	478.536	478.48	-0.056	0.003136
105726	1424897	3941699	486.276	486.22	-0.056	0.003136
105734	1424501	3941923	486.406	486.35	-0.056	0.003136
101394	1345829	3961264	436.845	436.79	-0.055	0.003025
101384	1345647	3960937	436.955	436.9	-0.055	0.003025
100165	1358234	3964590	437.105	437.05	-0.055	0.003025
103736	1374027	3960785	445.685	445.63	-0.055	0.003025
103461	1379548	3971065	449.865	449.81	-0.055	0.003025
100381	1351902	3969093	450.995	450.94	-0.055	0.003025
100409	1350792	3969040	454.975	454.92	-0.055	0.003025
103610	1375057	3966064	457.695	457.64	-0.055	0.003025
107662	1395429	3954097	458.635	458.58	-0.055	0.003025
106286	1421824	3972194	471.215	471.16	-0.055	0.003025
103380	1379604	3973737	471.765	471.71	-0.055	0.003025
106069	1420753	3958269	473.785	473.73	-0.055	0.003025
101842	1357775	3974061	474.575	474.52	-0.055	0.003025
101207	1338115	3951885	432.444	432.39	-0.054	0.002916
100044	1357436	3959884	437.024	436.97	-0.054	0.002916
103702	1374181	3962332	450.104	450.05	-0.054	0.002916
106123	1420878	3964026	471.894	471.84	-0.054	0.002916
106296	1421834	3972736	474.464	474.41	-0.054	0.002916
106724	1421819	3972831	474.764	474.71	-0.054	0.002916
105972	1420677	3953678	475.414	475.36	-0.054	0.002916
101843	1357745	3974086	475.574	475.52	-0.054	0.002916
105703	1426241	3941278	487.324	487.27	-0.054	0.002916
5540	1389081	4005926	743.984	743.93	-0.054	0.002916
100228	1358321	3967975	437.983	437.93	-0.053	0.002809
101447	1347205	3963848	440.163	440.11	-0.053	0.002809
101495	1348459	3966076	456.913	456.86	-0.053	0.002809
107464	1401852	3961182	460.313	460.26	-0.053	0.002809
101839	1357867	3973983	471.093	471.04	-0.053	0.002809

106771	1421776	3970523	471.963	471.91	-0.053	0.002809
105959	1421057	3953107	475.423	475.37	-0.053	0.002809
106690	1421853	3974628	482.253	482.2	-0.053	0.002809
105718	1425363	3941549	485.543	485.49	-0.053	0.002809
105546	1429993	3935348	493.403	493.35	-0.053	0.002809
101869	1356921	3974987	494.643	494.59	-0.053	0.002809
105470	1429075	3932734	495.753	495.7	-0.053	0.002809
105469	1429074	3932700	495.883	495.83	-0.053	0.002809
100151	1358220	3964007	435.062	435.01	-0.052	0.002704
103977	1373718	3950776	447.882	447.83	-0.052	0.002704
101589	1351956	3969071	450.682	450.63	-0.052	0.002704
104151	1372678	3947691	450.742	450.69	-0.052	0.002704
101577	1351446	3969073	451.322	451.27	-0.052	0.002704
101570	1351166	3969063	451.672	451.62	-0.052	0.002704
103496	1378590	3969828	451.912	451.86	-0.052	0.002704
106264	1421803	3971057	470.172	470.12	-0.052	0.002704
105551	1430072	3935498	493.362	493.31	-0.052	0.002704
101090	1334502	3948015	573.982	573.93	-0.052	0.002704
100818	1340562	3953704	432.871	432.82	-0.051	0.002601
100961	1335709	3949903	435.371	435.32	-0.051	0.002601
100094	1358162	3961849	435.441	435.39	-0.051	0.002601
100585	1346187	3961935	436.761	436.71	-0.051	0.002601
101132	1335544	3949714	437.541	437.49	-0.051	0.002601
103842	1373854	3956220	445.771	445.72	-0.051	0.002601
100372	1352301	3969076	449.441	449.39	-0.051	0.002601
100451	1348939	3967011	459.831	459.78	-0.051	0.002601
106869	1420866	3964153	471.431	471.38	-0.051	0.002601
106291	1421829	3972486	473.431	473.38	-0.051	0.002601
105935	1421464	3951920	475.861	475.81	-0.051	0.002601
101189	1337425	3951543	432.73	432.68	-0.05	0.0025
100209	1358301	3967135	435.68	435.63	-0.05	0.0025
100116	1358190	3962805	436.04	435.99	-0.05	0.0025
103816	1373876	3957334	446.41	446.36	-0.05	0.0025
100367	1352526	3969069	447.78	447.73	-0.05	0.0025
103454	1379595	3971354	449.35	449.3	-0.05	0.0025
104219	1372687	3947685	450.85	450.8	-0.05	0.0025
101831	1358098	3973737	460.53	460.48	-0.05	0.0025
105953	1421170	3952830	475.13	475.08	-0.05	0.0025
105723	1425076	3941635	485.85	485.8	-0.05	0.0025
105555	1430163	3935675	493.88	493.83	-0.05	0.0025
100928	1336681	3950912	432.709	432.66	-0.049	0.002401
101175	1336899	3951116	433.009	432.96	-0.049	0.002401
100865	1339013	3952297	433.939	433.89	-0.049	0.002401
100212	1358304	3967269	435.459	435.41	-0.049	0.002401
100033	1357201	3959765	436.039	435.99	-0.049	0.002401
103741	1374023	3960595	445.589	445.54	-0.049	0.002401
104423	1373744	3951386	446.649	446.6	-0.049	0.002401
100385	1351733	3969101	451.099	451.05	-0.049	0.002401
106223	1421776	3969732	472.809	472.76	-0.049	0.002401
105979	1420456	3953878	475.519	475.47	-0.049	0.002401
105474	1429103	3932901	495.369	495.32	-0.049	0.002401
105449	1429153	3931833	495.579	495.53	-0.049	0.002401

101297	1343664	3957406	433.788	433.74	-0.048	0.002304
101357	1345081	3959934	436.828	436.78	-0.048	0.002304
100243	1358341	3968605	437.058	437.01	-0.048	0.002304
103739	1374025	3960669	445.628	445.58	-0.048	0.002304
101808	1358388	3972660	445.768	445.72	-0.048	0.002304
104480	1373792	3953004	447.378	447.33	-0.048	0.002304
104225	1372733	3947763	451.178	451.13	-0.048	0.002304
103477	1379250	3970410	451.678	451.63	-0.048	0.002304
100499	1347929	3965200	454.338	454.29	-0.048	0.002304
106180	1420944	3966975	471.568	471.52	-0.048	0.002304
106239	1421796	3970371	471.968	471.92	-0.048	0.002304
106819	1420918	3966878	472.218	472.17	-0.048	0.002304
105729	1424735	3941776	486.208	486.16	-0.048	0.002304
105475	1429112	3932932	495.349	495.3	-0.048	0.002304
100825	1340361	3953474	432.867	432.82	-0.047	0.002209
100712	1343573	3957278	432.977	432.93	-0.047	0.002209
103943	1373739	3951742	446.487	446.44	-0.047	0.002209
101811	1358390	3972796	446.917	446.87	-0.047	0.002209
101584	1351740	3969069	450.827	450.78	-0.047	0.002209
100395	1351314	3969118	453.247	453.2	-0.047	0.002209
100496	1347992	3965313	454.677	454.63	-0.047	0.002209
107612	1396196	3956702	456.417	456.37	-0.047	0.002209
107560	1397255	3959107	459.027	458.98	-0.047	0.002209
107564	1397094	3958961	459.447	459.4	-0.047	0.002209
100441	1349154	3967394	460.397	460.35	-0.047	0.002209
105947	1421275	3952505	474.667	474.62	-0.047	0.002209
103368	1379651	3974282	484.287	484.24	-0.047	0.002209
100809	1340823	3954004	432.486	432.44	-0.046	0.002116
100650	1344867	3959581	437.027	436.98	-0.046	0.002116
103909	1373782	3953093	447.326	447.28	-0.046	0.002116
103458	1379573	3971191	449.466	449.42	-0.046	0.002116
104221	1372702	3947709	450.976	450.93	-0.046	0.002116
101826	1358218	3973543	455.146	455.1	-0.046	0.002116
107554	1397506	3959280	458.716	458.67	-0.046	0.002116
107557	1397361	3959186	458.926	458.88	-0.046	0.002116
100457	1348811	3966780	459.066	459.02	-0.046	0.002116
101535	1349408	3967770	459.326	459.28	-0.046	0.002116
106063	1420748	3957932	474.606	474.56	-0.046	0.002116
105737	1424355	3942044	486.366	486.32	-0.046	0.002116
100785	1341502	3954780	432.765	432.72	-0.045	0.002025
101400	1345984	3961540	436.685	436.64	-0.045	0.002025
100062	1358073	3960446	436.835	436.79	-0.045	0.002025
103756	1373867	3960122	444.125	444.08	-0.045	0.002025
100972	1335372	3949554	445.515	445.47	-0.045	0.002025
104437	1373749	3951670	446.875	446.83	-0.045	0.002025
103879	1373816	3954374	446.985	446.94	-0.045	0.002025
103880	1373815	3954330	447.125	447.08	-0.045	0.002025
103519	1377871	3969347	448.245	448.2	-0.045	0.002025
107538	1398270	3959641	457.635	457.59	-0.045	0.002025
106134	1420890	3964524	470.345	470.3	-0.045	0.002025
105985	1420298	3954001	476.045	476	-0.045	0.002025
105478	1429150	3933056	495.045	495	-0.045	0.002025

105463	1429099	3932429	495.635	495.59	-0.045	0.002025
100176	1358264	3965715	436.804	436.76	-0.044	0.001936
104444	1373753	3951852	446.154	446.11	-0.044	0.001936
103564	1376412	3968370	446.254	446.21	-0.044	0.001936
104503	1373825	3954202	447.224	447.18	-0.044	0.001936
107681	1395048	3953127	463.194	463.15	-0.044	0.001936
106939	1420731	3957895	474.734	474.69	-0.044	0.001936
105857	1422864	3947961	478.005	477.96	-0.044	0.001936
100904	1337476	3951594	433.143	433.1	-0.043	0.001849
100610	1345650	3960978	436.993	436.95	-0.043	0.001849
101750	1358370	3970225	440.263	440.22	-0.043	0.001849
101612	1352984	3969038	444.723	444.68	-0.043	0.001849
100495	1348013	3965351	454.853	454.81	-0.043	0.001849
107510	1399677	3960279	458.993	458.95	-0.043	0.001849
106196	1421291	3967898	467.824	467.78	-0.043	0.001849
105976	1420542	3953811	475.583	475.54	-0.043	0.001849
105648	1428519	3939547	489.433	489.39	-0.043	0.001849
100902	1337550	3951637	433.132	433.09	-0.042	0.001764
100102	1358171	3962195	436.652	436.61	-0.042	0.001764
101610	1352895	3969042	445.092	445.05	-0.042	0.001764
100362	1352747	3969066	445.962	445.92	-0.042	0.001764
103677	1374720	3963333	448.382	448.34	-0.042	0.001764
103689	1374467	3962884	448.522	448.48	-0.042	0.001764
103511	1378070	3969481	449.852	449.81	-0.042	0.001764
100397	1351230	3969114	453.552	453.51	-0.042	0.001764
101532	1349358	3967681	459.382	459.34	-0.042	0.001764
105717	1425411	3941536	485.622	485.58	-0.042	0.001764
105641	1428778	3939322	489.832	489.79	-0.042	0.001764
101736	1358364	3969334	435.751	435.71	-0.041	0.001681
101332	1344463	3958831	436.901	436.86	-0.041	0.001681
103698	1374260	3962510	450.621	450.58	-0.041	0.001681
101562	1350862	3969010	452.421	452.38	-0.041	0.001681
107450	1402590	3961419	461.391	461.35	-0.041	0.001681
106068	1420752	3958226	474.091	474.05	-0.041	0.001681
106043	1420730	3956914	474.831	474.79	-0.041	0.001681
106032	1420718	3956408	475.031	474.99	-0.041	0.001681
106300	1421839	3972945	475.161	475.12	-0.041	0.001681
100806	1340910	3954104	432.72	432.68	-0.04	0.0016
101291	1343397	3956983	433.49	433.45	-0.04	0.0016
101303	1343860	3957755	434.57	434.53	-0.04	0.0016
101607	1352762	3969047	445.99	445.95	-0.04	0.0016
103925	1373762	3952504	447.14	447.1	-0.04	0.0016
104379	1373713	3950453	453.52	453.48	-0.04	0.0016
100464	1348659	3966508	458.11	458.07	-0.04	0.0016
107484	1400925	3960827	460.62	460.58	-0.04	0.0016
106827	1420911	3966527	472.5	472.46	-0.04	0.0016
106881	1420852	3963529	473.65	473.61	-0.04	0.0016
105977	1420513	3953835	475.57	475.53	-0.04	0.0016
105716	1425458	3941521	485.64	485.6	-0.04	0.0016
105621	1429415	3938767	490.16	490.12	-0.04	0.0016
101776	1358371	3971269	439.709	439.67	-0.039	0.001521
100969	1335463	3949649	440.009	439.97	-0.039	0.001521

103873	1373822	3954694	446.289	446.25	-0.039	0.001521
104476	1373784	3952844	447.539	447.5	-0.039	0.001521
101734	1358364	3969257	435.958	435.92	-0.038	0.001444
100646	1344956	3959741	436.958	436.92	-0.038	0.001444
101752	1358372	3970307	440.048	440.01	-0.038	0.001444
101609	1352851	3969043	445.368	445.33	-0.038	0.001444
104452	1373760	3952083	446.088	446.05	-0.038	0.001444
104403	1373734	3950961	446.358	446.32	-0.038	0.001444
103528	1377594	3969168	447.788	447.75	-0.038	0.001444
103446	1379602	3971645	450.618	450.58	-0.038	0.001444
101588	1351913	3969071	450.958	450.92	-0.038	0.001444
103504	1378309	3969643	451.208	451.17	-0.038	0.001444
100422	1349573	3968140	459.829	459.79	-0.038	0.001444
100424	1349524	3968061	459.918	459.88	-0.038	0.001444
107670	1395306	3953677	460.108	460.07	-0.038	0.001444
107685	1394916	3952981	463.498	463.46	-0.038	0.001444
106087	1420770	3959203	474.168	474.13	-0.038	0.001444
106725	1421818	3972791	474.578	474.54	-0.038	0.001444
105739	1424289	3942109	486.308	486.27	-0.038	0.001444
105667	1427817	3940156	488.248	488.21	-0.038	0.001444
105645	1428636	3939445	489.658	489.62	-0.038	0.001444
100056	1357896	3960168	436.727	436.69	-0.037	0.001369
100236	1358337	3968319	437.417	437.38	-0.037	0.001369
103541	1377231	3968932	447.377	447.34	-0.037	0.001369
103505	1378273	3969619	450.977	450.94	-0.037	0.001369
101581	1351612	3969072	451.107	451.07	-0.037	0.001369
100489	1348139	3965577	455.697	455.66	-0.037	0.001369
100456	1348832	3966819	459.077	459.04	-0.037	0.001369
107565	1397036	3958901	459.527	459.49	-0.037	0.001369
106263	1421803	3971019	470.237	470.2	-0.037	0.001369
106865	1420872	3964362	470.757	470.72	-0.037	0.001369
106870	1420864	3964068	471.707	471.67	-0.037	0.001369
101840	1357836	3974010	472.227	472.19	-0.037	0.001369
106998	1420660	3955186	475.397	475.36	-0.037	0.001369
105405	1429140	3930434	496.937	496.9	-0.037	0.001369
101094	1334635	3948243	568.727	568.69	-0.037	0.001369
100790	1341362	3954622	432.696	432.66	-0.036	0.001296
100913	1337159	3951372	433.396	433.36	-0.036	0.001296
100077	1358143	3961121	436.126	436.09	-0.036	0.001296
101757	1358378	3970514	439.876	439.84	-0.036	0.001296
100529	1347276	3964034	440.856	440.82	-0.036	0.001296
103586	1375839	3967670	443.006	442.97	-0.036	0.001296
103832	1373865	3956622	446.026	445.99	-0.036	0.001296
103632	1374982	3965050	446.036	446	-0.036	0.001296
103881	1373814	3954285	447.176	447.14	-0.036	0.001296
101514	1348938	3966931	459.526	459.49	-0.036	0.001296
100979	1335165	3949341	465.956	465.92	-0.036	0.001296
106808	1420998	3967334	471.166	471.13	-0.036	0.001296
100985	1335000	3949170	486.906	486.87	-0.036	0.001296
103363	1379709	3974510	488.507	488.47	-0.036	0.001296
105655	1428274	3939759	488.866	488.83	-0.036	0.001296
105519	1429541	3934283	493.346	493.31	-0.036	0.001296

5534	1388855	4005696	741.136	741.1	-0.036	0.001296
100761	1342185	3955562	433.165	433.13	-0.035	0.001225
100590	1346076	3961738	436.485	436.45	-0.035	0.001225
101122	1335205	3949364	462.175	462.14	-0.035	0.001225
101023	1334264	3947830	567.685	567.65	-0.035	0.001225
100787	1341446	3954717	432.754	432.72	-0.034	0.001156
101147	1336020	3950207	433.314	433.28	-0.034	0.001156
100616	1345531	3960767	436.944	436.91	-0.034	0.001156
103577	1376029	3967925	444.104	444.07	-0.034	0.001156
101603	1352584	3969053	447.434	447.4	-0.034	0.001156
103480	1379165	3970301	451.863	451.83	-0.034	0.001156
104389	1373723	3950644	453.024	452.99	-0.034	0.001156
107617	1396118	3956435	456.294	456.26	-0.034	0.001156
107641	1395761	3955221	458.634	458.6	-0.034	0.001156
101530	1349321	3967615	459.574	459.54	-0.034	0.001156
106255	1421801	3970723	471.474	471.44	-0.034	0.001156
106009	1420682	3955238	475.754	475.72	-0.034	0.001156
105623	1429371	3938806	490.154	490.12	-0.034	0.001156
105516	1429513	3934186	493.184	493.15	-0.034	0.001156
105539	1429922	3935215	493.634	493.6	-0.034	0.001156
100762	1342155	3955527	433.203	433.17	-0.033	0.001089
101611	1352939	3969040	444.843	444.81	-0.033	0.001089
103966	1373728	3951082	446.313	446.28	-0.033	0.001089
103914	1373774	3952898	447.493	447.46	-0.033	0.001089
103683	1374600	3963119	448.693	448.66	-0.033	0.001089
104167	1372536	3947441	450.533	450.5	-0.033	0.001089
107606	1396272	3956955	456.103	456.07	-0.033	0.001089
106755	1421789	3971275	469.733	469.7	-0.033	0.001089
106145	1420901	3965018	470.433	470.4	-0.033	0.001089
105982	1420353	3953946	475.423	475.39	-0.033	0.001089
105712	1425694	3941451	485.603	485.57	-0.033	0.001089
101046	1333133	3947134	551.513	551.48	-0.033	0.001089
5544	1389229	4006075	746.043	746.01	-0.033	0.001089
100927	1336710	3950942	432.792	432.76	-0.032	0.001024
100108	1358178	3962461	436.612	436.58	-0.032	0.001024
100665	1344541	3958999	436.762	436.73	-0.032	0.001024
103535	1377423	3969056	447.612	447.58	-0.032	0.001024
100383	1351817	3969097	450.912	450.88	-0.032	0.001024
104371	1373705	3950291	453.632	453.6	-0.032	0.001024
106862	1420875	3964484	470.412	470.38	-0.032	0.001024
106777	1421770	3970256	472.102	472.07	-0.032	0.001024
105988	1420308	3954081	474.822	474.79	-0.032	0.001024
106331	1421873	3974637	482.302	482.27	-0.032	0.001024
105728	1424774	3941755	486.172	486.14	-0.032	0.001024
100702	1343790	3957654	434.181	434.15	-0.031	0.000961
100604	1345775	3961201	436.791	436.76	-0.031	0.000961
101390	1345758	3961136	436.901	436.87	-0.031	0.000961
100500	1347907	3965162	454.281	454.25	-0.031	0.000961
101555	1349857	3968430	456.471	456.44	-0.031	0.000961
101496	1348480	3966112	457.071	457.04	-0.031	0.000961
107460	1402057	3961258	460.481	460.45	-0.031	0.000961
106864	1420873	3964403	470.691	470.66	-0.031	0.000961

106131	1420887	3964383	470.691	470.66	-0.031	0.000961
106262	1421802	3970950	470.942	470.91	-0.031	0.000961
106811	1420966	3967211	471.811	471.78	-0.031	0.000961
106909	1420760	3959378	475.002	474.97	-0.031	0.000961
106002	1420617	3954870	475.761	475.73	-0.031	0.000961
106685	1421858	3974885	484.941	484.91	-0.031	0.000961
101853	1357434	3974340	485.191	485.16	-0.031	0.000961
101016	1334497	3948028	573.461	573.43	-0.031	0.000961
101173	1336838	3951054	432.93	432.9	-0.03	0.0009
101176	1336930	3951148	433.07	433.04	-0.03	0.0009
101137	1335701	3949876	435.27	435.24	-0.03	0.0009
101315	1344101	3958184	436.08	436.05	-0.03	0.0009
100648	1344912	3959662	436.93	436.9	-0.03	0.0009
100613	1345591	3960872	436.96	436.93	-0.03	0.0009
103723	1374042	3961323	446.29	446.26	-0.03	0.0009
103566	1376350	3968311	446.34	446.31	-0.03	0.0009
104422	1373744	3951366	446.65	446.62	-0.03	0.0009
103618	1375016	3965688	449.42	449.39	-0.03	0.0009
101492	1348376	3965927	456.5	456.47	-0.03	0.0009
106271	1421808	3971438	469.74	469.71	-0.03	0.0009
106113	1420867	3963572	473.59	473.56	-0.03	0.0009
106941	1420730	3957820	474.71	474.68	-0.03	0.0009
106039	1420726	3956723	475.09	475.06	-0.03	0.0009
105635	1428991	3939136	490.17	490.14	-0.03	0.0009
101862	1357119	3974637	491.94	491.91	-0.03	0.0009
101248	1340825	3953987	432.889	432.86	-0.029	0.000841
100087	1358155	3961555	435.039	435.01	-0.029	0.000841
101738	1358362	3969414	435.799	435.77	-0.029	0.000841
101615	1353118	3969032	444.779	444.75	-0.029	0.000841
103745	1374018	3960450	445.429	445.4	-0.029	0.000841
101810	1358389	3972750	446.619	446.59	-0.029	0.000841
104380	1373714	3950472	453.559	453.53	-0.029	0.000841
101520	1349085	3967195	460.289	460.26	-0.029	0.000841
101837	1357928	3973928	468.489	468.46	-0.029	0.000841
106222	1421775	3969709	472.69	472.66	-0.029	0.000841
105983	1420334	3953958	475.449	475.42	-0.029	0.000841
101022	1334303	3947854	569.259	569.23	-0.029	0.000841
101287	1343125	3956623	433.368	433.34	-0.028	0.000784
100140	1358212	3963601	434.768	434.74	-0.028	0.000784
100184	1358273	3966061	438.388	438.36	-0.028	0.000784
101795	1358377	3972084	442.458	442.43	-0.028	0.000784
103732	1374032	3960947	445.858	445.83	-0.028	0.000784
103635	1374976	3964925	446.048	446.02	-0.028	0.000784
104511	1373835	3954598	446.458	446.43	-0.028	0.000784
103552	1376833	3968674	446.628	446.6	-0.028	0.000784
104398	1373730	3950846	446.838	446.81	-0.028	0.000784
101468	1347751	3964806	453.228	453.2	-0.028	0.000784
104368	1373702	3950229	453.658	453.63	-0.028	0.000784
100491	1348097	3965501	455.528	455.5	-0.028	0.000784
107615	1396152	3956551	456.138	456.11	-0.028	0.000784
101533	1349375	3967710	459.328	459.3	-0.028	0.000784
107468	1401661	3961108	460.378	460.35	-0.028	0.000784

107467	1401698	3961123	460.418	460.39	-0.028	0.000784
106817	1420925	3966963	472.138	472.11	-0.028	0.000784
106309	1421847	3973418	476.828	476.8	-0.028	0.000784
105438	1429142	3931320	495.668	495.64	-0.028	0.000784
5550	1389458	4006308	749.138	749.11	-0.028	0.000784
100222	1358315	3967712	436.347	436.32	-0.027	0.000729
101366	1345273	3960278	436.957	436.93	-0.027	0.000729
101761	1358383	3970685	439.357	439.33	-0.027	0.000729
103792	1373710	3958948	444.517	444.49	-0.027	0.000729
103725	1374039	3961241	446.157	446.13	-0.027	0.000729
104413	1373738	3951174	446.537	446.51	-0.027	0.000729
104500	1373821	3954043	447.037	447.01	-0.027	0.000729
103902	1373790	3953366	447.137	447.11	-0.027	0.000729
104386	1373721	3950585	454.267	454.24	-0.027	0.000729
107511	1399638	3960261	458.917	458.89	-0.027	0.000729
106257	1421800	3970774	471.177	471.15	-0.027	0.000729
106057	1420743	3957639	474.757	474.73	-0.027	0.000729
106952	1420719	3957312	474.807	474.78	-0.027	0.000729
105643	1428722	3939371	489.677	489.65	-0.027	0.000729
100797	1341168	3954399	433.026	433	-0.026	0.000676
100813	1340707	3953870	433.166	433.14	-0.026	0.000676
101395	1345848	3961298	436.906	436.88	-0.026	0.000676
101444	1347120	3963688	439.916	439.89	-0.026	0.000676
103783	1373653	3959305	443.676	443.65	-0.026	0.000676
104439	1373751	3951717	446.616	446.59	-0.026	0.000676
103551	1376869	3968698	446.986	446.96	-0.026	0.000676
103924	1373763	3952539	447.206	447.18	-0.026	0.000676
103543	1377160	3968886	447.366	447.34	-0.026	0.000676
103684	1374579	3963081	448.656	448.63	-0.026	0.000676
100387	1351649	3969106	451.226	451.2	-0.026	0.000676
107552	1397617	3959339	458.616	458.59	-0.026	0.000676
107516	1399400	3960151	458.696	458.67	-0.026	0.000676
106853	1420887	3964972	470.296	470.27	-0.026	0.000676
106187	1421103	3967557	470.366	470.34	-0.026	0.000676
106687	1421856	3974756	482.986	482.96	-0.026	0.000676
105471	1429078	3932768	495.656	495.63	-0.026	0.000676
101170	1336748	3950961	432.775	432.75	-0.025	0.000625
101289	1343290	3956835	433.465	433.44	-0.025	0.000625
101742	1358361	3969581	436.285	436.26	-0.025	0.000625
100225	1358317	3967844	437.315	437.29	-0.025	0.000625
100226	1358319	3967888	437.555	437.53	-0.025	0.000625
103647	1374949	3964434	444.845	444.82	-0.025	0.000625
100360	1352833	3969064	445.275	445.25	-0.025	0.000625
104424	1373744	3951406	446.665	446.64	-0.025	0.000625
104436	1373749	3951649	446.815	446.79	-0.025	0.000625
103980	1373716	3950713	449.915	449.89	-0.025	0.000625
100380	1351943	3969091	450.795	450.77	-0.025	0.000625
100390	1351525	3969114	451.945	451.92	-0.025	0.000625
107686	1394889	3952957	463.405	463.38	-0.025	0.000625
106283	1421821	3972070	471.585	471.56	-0.025	0.000625
106248	1421811	3970549	471.785	471.76	-0.025	0.000625
105846	1423225	3947468	476.625	476.6	-0.025	0.000625

105878	1422360	3949039	476.805	476.78	-0.025	0.000625
105730	1424695	3941797	486.225	486.2	-0.025	0.000625
105694	1426699	3941054	488.195	488.17	-0.025	0.000625
100134	1358211	3963410	435.034	435.01	-0.024	0.000576
100211	1358303	3967225	435.514	435.49	-0.024	0.000576
100244	1358341	3968639	437.144	437.12	-0.024	0.000576
103580	1375954	3967824	443.674	443.65	-0.024	0.000576
103941	1373741	3951806	446.324	446.3	-0.024	0.000576
103883	1373811	3954194	447.214	447.19	-0.024	0.000576
103997	1373691	3950361	453.524	453.5	-0.024	0.000576
107542	1398081	3959553	457.614	457.59	-0.024	0.000576
107523	1399030	3959982	458.404	458.38	-0.024	0.000576
106240	1421798	3970390	471.974	471.95	-0.024	0.000576
106075	1420757	3958569	472.934	472.91	-0.024	0.000576
105884	1422257	3949370	475.704	475.68	-0.024	0.000576
101036	1333743	3947526	548.504	548.48	-0.024	0.000576
100815	1340649	3953803	432.943	432.92	-0.023	0.000529
101247	1340796	3953953	433.023	433	-0.023	0.000529
101737	1358363	3969373	435.813	435.79	-0.023	0.000529
101391	1345775	3961167	436.893	436.87	-0.023	0.000529
101431	1346830	3963107	438.343	438.32	-0.023	0.000529
103752	1373964	3960220	444.363	444.34	-0.023	0.000529
103930	1373751	3952188	446.053	446.03	-0.023	0.000529
101595	1352224	3969067	450.053	450.03	-0.023	0.000529
107553	1397543	3959300	458.703	458.68	-0.023	0.000529
106048	1420735	3957187	474.633	474.61	-0.023	0.000529
105472	1429082	3932802	495.623	495.6	-0.023	0.000529
101081	1334198	3947776	565.423	565.4	-0.023	0.000529
100724	1343271	3956832	433.112	433.09	-0.022	0.000484
100136	1358212	3963468	434.902	434.88	-0.022	0.000484
100168	1358239	3964731	437.342	437.32	-0.022	0.000484
101616	1353159	3969031	444.912	444.89	-0.022	0.000484
104506	1373828	3954362	447.112	447.09	-0.022	0.000484
100376	1352116	3969083	450.542	450.52	-0.022	0.000484
106791	1421291	3967933	467.502	467.48	-0.022	0.000484
106813	1420949	3967130	471.922	471.9	-0.022	0.000484
106221	1421775	3969684	472.472	472.45	-0.022	0.000484
101038	1333630	3947461	544.482	544.46	-0.022	0.000484
100097	1358166	3961976	435.951	435.93	-0.021	0.000441
100643	1345018	3959854	436.771	436.75	-0.021	0.000441
101345	1344741	3959327	437.221	437.2	-0.021	0.000441
103947	1373736	3951618	446.251	446.23	-0.021	0.000441
104513	1373836	3954677	446.331	446.31	-0.021	0.000441
103685	1374557	3963043	448.641	448.62	-0.021	0.000441
100411	1350746	3969027	455.111	455.09	-0.021	0.000441
107529	1398698	3959833	458.091	458.07	-0.021	0.000441
105625	1429302	3938867	490.381	490.36	-0.021	0.000441
101092	1334553	3948086	573.201	573.18	-0.021	0.000441
101160	1336456	3950654	432.72	432.7	-0.02	0.0004
101727	1358360	3969026	436.98	436.96	-0.02	0.0004
101613	1353030	3969036	444.65	444.63	-0.02	0.0004
103970	1373726	3950965	446.46	446.44	-0.02	0.0004

107561	1397187	3959050	459.16	459.14	-0.02	0.0004
101536	1349426	3967800	459.31	459.29	-0.02	0.0004
107470	1401552	3961065	460.3	460.28	-0.02	0.0004
106254	1421803	3970677	471.61	471.59	-0.02	0.0004
106067	1420751	3958141	474.26	474.24	-0.02	0.0004
101845	1357685	3974136	477.75	477.73	-0.02	0.0004
101078	1334079	3947708	560.8	560.78	-0.02	0.0004
100929	1336652	3950881	432.709	432.69	-0.019	0.000361
101217	1338524	3952066	432.739	432.72	-0.019	0.000361
100742	1342758	3956217	433.009	432.99	-0.019	0.000361
101144	1335924	3950107	433.879	433.86	-0.019	0.000361
100031	1357151	3959740	436.039	436.02	-0.019	0.000361
100681	1344212	3958412	436.659	436.64	-0.019	0.000361
101348	1344807	3959447	437.049	437.03	-0.019	0.000361
104457	1373764	3952218	446.069	446.05	-0.019	0.000361
104395	1373728	3950777	447.819	447.8	-0.019	0.000361
101828	1358173	3973625	457.019	457	-0.019	0.000361
107581	1396631	3958214	460.229	460.21	-0.019	0.000361
100980	1335137	3949312	469.449	469.43	-0.019	0.000361
106888	1420845	3963218	473.899	473.88	-0.019	0.000361
101017	1334470	3947997	573.539	573.52	-0.019	0.000361
100875	1338584	3952107	432.908	432.89	-0.018	0.000324
100113	1358185	3962678	436.338	436.32	-0.018	0.000324
101379	1345548	3960759	436.908	436.89	-0.018	0.000324
100542	1347009	3963514	439.868	439.85	-0.018	0.000324
103581	1375931	3967794	443.478	443.46	-0.018	0.000324
104431	1373746	3951547	446.208	446.19	-0.018	0.000324
104434	1373748	3951609	446.378	446.36	-0.018	0.000324
104145	1372730	3947782	451.078	451.06	-0.018	0.000324
103478	1379223	3970373	451.798	451.78	-0.018	0.000324
103491	1378780	3969953	451.918	451.9	-0.018	0.000324
100407	1350847	3969054	454.788	454.77	-0.018	0.000324
106790	1421312	3967966	466.848	466.83	-0.018	0.000324
106294	1421831	3972611	474.108	474.09	-0.018	0.000324
106329	1421870	3974505	481.908	481.89	-0.018	0.000324
101209	1338194	3951921	432.517	432.5	-0.017	0.000289
101162	1336514	3950714	432.717	432.7	-0.017	0.000289
100823	1340419	3953540	432.927	432.91	-0.017	0.000289
101177	1336961	3951179	433.077	433.06	-0.017	0.000289
100061	1358052	3960398	436.767	436.75	-0.017	0.000289
100612	1345610	3960907	436.998	436.98	-0.017	0.000289
101769	1358388	3970987	438.507	438.49	-0.017	0.000289
103800	1373750	3958611	446.017	446	-0.017	0.000289
103706	1374117	3962143	449.687	449.67	-0.017	0.000289
103984	1373712	3950632	453.137	453.12	-0.017	0.000289
107586	1396557	3957970	459.527	459.51	-0.017	0.000289
101838	1357897	3973956	469.847	469.83	-0.017	0.000289
106290	1421827	3972402	472.657	472.64	-0.017	0.000289
101161	1336485	3950684	432.666	432.65	-0.016	0.000256
100821	1340476	3953605	432.836	432.82	-0.016	0.000256
101263	1341835	3955141	432.906	432.89	-0.016	0.000256
100109	1358179	3962505	436.586	436.57	-0.016	0.000256

101408	1346193	3961911	436.746	436.73	-0.016	0.000256
103931	1373751	3952152	445.976	445.96	-0.016	0.000256
103945	1373737	3951679	446.526	446.51	-0.016	0.000256
104400	1373731	3950891	446.576	446.56	-0.016	0.000256
103922	1373764	3952606	447.516	447.5	-0.016	0.000256
103530	1377548	3969139	447.736	447.72	-0.016	0.000256
100382	1351859	3969095	450.896	450.88	-0.016	0.000256
101579	1351528	3969073	451.306	451.29	-0.016	0.000256
103429	1379602	3971951	452.856	452.84	-0.016	0.000256
106775	1421772	3970332	472.056	472.04	-0.016	0.000256
105731	1424656	3941820	486.236	486.22	-0.016	0.000256
105629	1429175	3938979	490.386	490.37	-0.016	0.000256
105411	1429030	3930681	496.946	496.93	-0.016	0.000256
101192	1337541	3951611	432.635	432.62	-0.015	0.000225
100805	1340939	3954137	432.655	432.64	-0.015	0.000225
100714	1343526	3957202	432.895	432.88	-0.015	0.000225
100099	1358168	3962062	436.265	436.25	-0.015	0.000225
100551	1346826	3963147	438.425	438.41	-0.015	0.000225
101751	1358371	3970266	440.195	440.18	-0.015	0.000225
103621	1375008	3965541	447.215	447.2	-0.015	0.000225
104173	1372481	3947363	450.435	450.42	-0.015	0.000225
103501	1378412	3969712	451.295	451.28	-0.015	0.000225
103425	1379603	3972036	453.425	453.41	-0.015	0.000225
107545	1397927	3959482	457.425	457.41	-0.015	0.000225
107562	1397155	3959020	459.265	459.25	-0.015	0.000225
105901	1421991	3950224	474.875	474.86	-0.015	0.000225
106701	1421842	3974026	479.225	479.21	-0.015	0.000225
105531	1429729	3934836	493.515	493.5	-0.015	0.000225
101098	1334696	3948453	553.295	553.28	-0.015	0.000225
101243	1340629	3953762	432.904	432.89	-0.014	0.000196
100083	1358150	3961381	435.344	435.33	-0.014	0.000196
100639	1345098	3959999	436.904	436.89	-0.014	0.000196
101387	1345710	3961050	436.914	436.9	-0.014	0.000196
103569	1376258	3968216	446.284	446.27	-0.014	0.000196
103904	1373788	3953289	446.894	446.88	-0.014	0.000196
104392	1373725	3950708	450.224	450.21	-0.014	0.000196
103502	1378378	3969689	451.344	451.33	-0.014	0.000196
100433	1349326	3967706	459.474	459.46	-0.014	0.000196
106073	1420756	3958482	473.174	473.16	-0.014	0.000196
106985	1420691	3955807	475.384	475.37	-0.014	0.000196
105853	1422993	3947778	477.444	477.43	-0.014	0.000196
106686	1421857	3974842	484.224	484.21	-0.014	0.000196
101163	1336542	3950744	432.723	432.71	-0.013	0.000169
101302	1343840	3957717	434.523	434.51	-0.013	0.000169
104482	1373797	3953145	447.143	447.13	-0.013	0.000169
101125	1335296	3949459	452.233	452.22	-0.013	0.000169
103993	1373695	3950446	453.613	453.6	-0.013	0.000169
100490	1348117	3965539	455.613	455.6	-0.013	0.000169
101522	1349128	3967272	460.433	460.42	-0.013	0.000169
106209	1421622	3968496	464.753	464.74	-0.013	0.000169
106146	1420902	3965052	470.453	470.44	-0.013	0.000169
106732	1421812	3972439	472.733	472.72	-0.013	0.000169

106949	1420722	3957459	474.933	474.92	-0.013	0.000169
105670	1427700	3940258	488.043	488.03	-0.013	0.000169
105487	1429249	3933283	494.463	494.45	-0.013	0.000169
101087	1334413	3947923	573.353	573.34	-0.013	0.000169
100715	1343502	3957164	432.832	432.82	-0.012	0.000144
100706	1343705	3957504	433.722	433.71	-0.012	0.000144
101407	1346149	3961834	436.842	436.83	-0.012	0.000144
101412	1346279	3962065	436.852	436.84	-0.012	0.000144
101748	1358368	3970144	439.272	439.26	-0.012	0.000144
101448	1347226	3963888	440.372	440.36	-0.012	0.000144
103690	1374443	3962842	448.492	448.48	-0.012	0.000144
107462	1401933	3961212	460.372	460.36	-0.012	0.000144
106860	1420878	3964605	470.473	470.46	-0.012	0.000144
105896	1422067	3949978	473.772	473.76	-0.012	0.000144
105951	1421210	3952709	474.552	474.54	-0.012	0.000144
105859	1422814	3948037	478.162	478.15	-0.012	0.000144
105486	1429244	3933265	494.582	494.57	-0.012	0.000144
101281	1342360	3955743	433.051	433.04	-0.011	0.000121
100126	1358204	3963191	435.381	435.37	-0.011	0.000121
100106	1358176	3962373	436.731	436.72	-0.011	0.000121
101411	1346257	3962027	436.811	436.8	-0.011	0.000121
100531	1347231	3963954	440.771	440.76	-0.011	0.000121
103933	1373749	3952080	445.921	445.91	-0.011	0.000121
104410	1373738	3951110	446.291	446.28	-0.011	0.000121
106135	1420891	3964560	470.361	470.35	-0.011	0.000121
106863	1420874	3964444	470.621	470.61	-0.011	0.000121
106130	1420886	3964347	470.731	470.72	-0.011	0.000121
106084	1420768	3959079	473.701	473.69	-0.011	0.000121
106292	1421830	3972528	473.721	473.71	-0.011	0.000121
105849	1423122	3947607	476.931	476.92	-0.011	0.000121
105468	1429075	3932632	495.911	495.9	-0.011	0.000121
105412	1429015	3930713	496.941	496.93	-0.011	0.000121
101031	1333963	3947655	556.831	556.82	-0.011	0.000121
101015	1334522	3948061	573.101	573.09	-0.011	0.000121
101198	1337768	3951728	432.54	432.53	-0.01	0.0001
101255	1341573	3954841	432.88	432.87	-0.01	0.0001
101335	1344527	3958944	436.71	436.7	-0.01	0.0001
103731	1374033	3960989	445.89	445.88	-0.01	0.0001
101461	1347584	3964508	450.72	450.71	-0.01	0.0001
101528	1349279	3967541	459.93	459.92	-0.01	0.0001
106765	1421781	3970819	471.02	471.01	-0.01	0.0001
106768	1421779	3970675	471.62	471.61	-0.01	0.0001
106040	1420728	3956800	474.95	474.94	-0.01	0.0001
106308	1421846	3973375	476.45	476.44	-0.01	0.0001
105617	1429536	3938664	490.11	490.1	-0.01	0.0001
105536	1429845	3935067	494.19	494.18	-0.01	0.0001
105408	1429091	3930543	496.89	496.88	-0.01	0.0001
101168	1336688	3950899	432.639	432.63	-0.009	0.000081
101251	1341271	3954494	433.039	433.03	-0.009	0.000081
100178	1358267	3965801	436.839	436.83	-0.009	0.000081
100224	1358316	3967800	436.949	436.94	-0.009	0.000081
101791	1358372	3971907	441.899	441.89	-0.009	0.000081

101793	1358375	3971997	442.069	442.06	-0.009	0.000081
103592	1375664	3967442	442.959	442.95	-0.009	0.000081
103773	1373591	3959682	443.949	443.94	-0.009	0.000081
103998	1373690	3950341	453.529	453.52	-0.009	0.000081
107656	1395529	3954435	458.029	458.02	-0.009	0.000081
107452	1402499	3961399	461.439	461.43	-0.009	0.000081
106842	1420895	3965504	470.459	470.45	-0.009	0.000081
106167	1420918	3965976	472.639	472.63	-0.009	0.000081
101154	1336274	3950468	432.958	432.95	-0.008	0.000064
101295	1343569	3957247	433.178	433.17	-0.008	0.000064
100042	1357386	3959858	436.898	436.89	-0.008	0.000064
100180	1358269	3965888	436.968	436.96	-0.008	0.000064
100186	1358275	3966143	439.398	439.39	-0.008	0.000064
100359	1352875	3969064	445.008	445	-0.008	0.000064
104404	1373735	3950984	446.298	446.29	-0.008	0.000064
103676	1374738	3963368	448.098	448.09	-0.008	0.000064
103469	1379430	3970721	450.708	450.7	-0.008	0.000064
105952	1421184	3952790	474.958	474.95	-0.008	0.000064
107001	1420637	3955037	475.348	475.34	-0.008	0.000064
105885	1422244	3949411	475.668	475.66	-0.008	0.000064
106698	1421845	3974199	480.178	480.17	-0.008	0.000064
100717	1343453	3957089	432.787	432.78	-0.007	0.000049
100799	1341111	3954334	432.907	432.9	-0.007	0.000049
101266	1341918	3955237	432.917	432.91	-0.007	0.000049
100138	1358212	3963532	434.897	434.89	-0.007	0.000049
100048	1357572	3959951	436.477	436.47	-0.007	0.000049
101350	1344852	3959527	437.147	437.14	-0.007	0.000049
100526	1347344	3964154	442.567	442.56	-0.007	0.000049
103806	1373785	3958346	444.817	444.81	-0.007	0.000049
103951	1373735	3951501	446.457	446.45	-0.007	0.000049
103694	1374350	3962678	449.697	449.69	-0.007	0.000049
104159	1372606	3947564	450.327	450.32	-0.007	0.000049
101539	1349478	3967893	459.207	459.2	-0.007	0.000049
100418	1349677	3968289	459.377	459.37	-0.007	0.000049
107463	1401892	3961197	460.367	460.36	-0.007	0.000049
106099	1420782	3959798	474.827	474.82	-0.007	0.000049
105939	1421405	3952108	475.357	475.35	-0.007	0.000049
105746	1424047	3942422	485.567	485.56	-0.007	0.000049
103353	1379903	3974955	499.417	499.41	-0.007	0.000049
5533	1388818	4005659	740.687	740.68	-0.007	0.000049
100943	1336222	3950436	432.856	432.85	-0.006	0.000036
100748	1342580	3956014	433.096	433.09	-0.006	0.000036
100213	1358306	3967314	435.416	435.41	-0.006	0.000036
100617	1345511	3960733	436.916	436.91	-0.006	0.000036
104451	1373759	3952055	446.066	446.06	-0.006	0.000036
104399	1373731	3950868	446.666	446.66	-0.006	0.000036
101812	1358390	3972842	447.456	447.45	-0.006	0.000036
103533	1377477	3969091	447.716	447.71	-0.006	0.000036
103523	1377804	3969303	447.846	447.84	-0.006	0.000036
103687	1374513	3962965	448.616	448.61	-0.006	0.000036
104220	1372695	3947697	450.956	450.95	-0.006	0.000036
103440	1379601	3971804	451.656	451.65	-0.006	0.000036

103486	1378964	3970096	452.216	452.21	-0.006	0.000036
104085	1373286	3948759	452.266	452.26	-0.006	0.000036
101549	1349695	3968239	457.256	457.25	-0.006	0.000036
107503	1400034	3960444	459.346	459.34	-0.006	0.000036
106017	1420703	3955632	475.166	475.16	-0.006	0.000036
105940	1421380	3952185	475.376	475.37	-0.006	0.000036
105960	1421018	3953184	475.416	475.41	-0.006	0.000036
101118	1335081	3949235	477.506	477.5	-0.006	0.000036
101064	1333563	3947408	542.266	542.26	-0.006	0.000036
100915	1337090	3951316	433.455	433.45	-0.005	0.000025
100229	1358322	3968019	438.096	438.09	-0.005	0.000025
104391	1373724	3950686	451.285	451.28	-0.005	0.000025
101479	1348043	3965328	454.595	454.59	-0.005	0.000025
101124	1335266	3949428	455.275	455.27	-0.005	0.000025
101557	1349916	3968489	456.165	456.16	-0.005	0.000025
106125	1420880	3964132	471.485	471.48	-0.005	0.000025
106997	1420665	3955224	475.255	475.25	-0.005	0.000025
105919	1421712	3951113	477.555	477.55	-0.005	0.000025
101846	1357655	3974161	478.925	478.92	-0.005	0.000025
106334	1421875	3974764	483.105	483.1	-0.005	0.000025
101049	1333151	3947146	550.665	550.66	-0.005	0.000025
101205	1338037	3951849	432.374	432.37	-0.004	0.000016
100747	1342610	3956048	433.054	433.05	-0.004	0.000016
100090	1358158	3961682	435.034	435.03	-0.004	0.000016
100232	1358327	3968149	438.134	438.13	-0.004	0.000016
101756	1358377	3970472	440.014	440.01	-0.004	0.000016
103935	1373747	3952010	446.074	446.07	-0.004	0.000016
104303	1373415	3948967	452.804	452.8	-0.004	0.000016
104004	1373686	3950223	453.504	453.5	-0.004	0.000016
104383	1373718	3950529	453.744	453.74	-0.004	0.000016
107528	1398781	3959870	458.064	458.06	-0.004	0.000016
105709	1425879	3941394	485.714	485.71	-0.004	0.000016
105654	1428305	3939732	488.944	488.94	-0.004	0.000016
100101	1358170	3962151	436.583	436.58	-0.003	0.000009
100248	1358341	3968736	437.303	437.3	-0.003	0.000009
103531	1377524	3969123	447.763	447.76	-0.003	0.000009
101572	1351246	3969070	451.273	451.27	-0.003	0.000009
104191	1372465	3947310	451.483	451.48	-0.003	0.000009
104078	1373353	3948878	452.373	452.37	-0.003	0.000009
103990	1373701	3950507	453.763	453.76	-0.003	0.000009
106800	1421119	3967634	470.943	470.94	-0.003	0.000009
106802	1421086	3967563	471.023	471.02	-0.003	0.000009
106828	1420911	3966496	472.503	472.5	-0.003	0.000009
105803	1424068	3945594	476.393	476.39	-0.003	0.000009
105661	1428029	3939971	488.503	488.5	-0.003	0.000009
105495	1429294	3933516	493.273	493.27	-0.003	0.000009
101097	1334685	3948413	556.873	556.87	-0.003	0.000009
101252	1341361	3954598	432.752	432.75	-0.002	0.000004
101253	1341392	3954634	432.782	432.78	-0.002	0.000004
100743	1342728	3956183	433.012	433.01	-0.002	0.000004
100737	1342904	3956387	433.032	433.03	-0.002	0.000004
101270	1342025	3955359	433.062	433.06	-0.002	0.000004

101311	1344021	3958043	435.582	435.58	-0.002	0.000004
100100	1358169	3962106	436.492	436.49	-0.002	0.000004
100591	1346054	3961699	436.572	436.57	-0.002	0.000004
100174	1358262	3965630	437.052	437.05	-0.002	0.000004
101445	1347161	3963767	440.232	440.23	-0.002	0.000004
103940	1373742	3951839	446.072	446.07	-0.002	0.000004
104449	1373757	3951999	446.122	446.12	-0.002	0.000004
103886	1373807	3954056	447.082	447.08	-0.002	0.000004
104467	1373775	3952571	447.632	447.63	-0.002	0.000004
103979	1373717	3950733	449.152	449.15	-0.002	0.000004
103604	1375366	3966991	451.832	451.83	-0.002	0.000004
100406	1350878	3969061	454.712	454.71	-0.002	0.000004
106247	1421811	3970533	471.842	471.84	-0.002	0.000004
101261	1341778	3955076	432.921	432.92	-0.001	0.000001
101271	1342052	3955389	433.091	433.09	-0.001	0.000001
101404	1346086	3961721	436.601	436.6	-0.001	0.000001
100592	1346033	3961660	436.691	436.69	-0.001	0.000001
100566	1346630	3962765	436.851	436.85	-0.001	0.000001
100641	1345059	3959926	436.861	436.86	-0.001	0.000001
101376	1345495	3960666	436.861	436.86	-0.001	0.000001
100065	1358116	3960591	436.921	436.92	-0.001	0.000001
103764	1373662	3960058	443.851	443.85	-0.001	0.000001
103939	1373743	3951873	445.931	445.93	-0.001	0.000001
104222	1372709	3947722	451.021	451.02	-0.001	0.000001
101824	1358256	3973460	453.941	453.94	-0.001	0.000001
101545	1349598	3968100	457.801	457.8	-0.001	0.000001
107504	1399993	3960425	459.261	459.26	-0.001	0.000001
107453	1402409	3961376	461.651	461.65	-0.001	0.000001
106175	1420931	3966728	472.311	472.31	-0.001	0.000001
106033	1420719	3956449	474.981	474.98	-0.001	0.000001
106688	1421854	3974713	482.571	482.57	-0.001	0.000001
5542	1389155	4006000	744.941	744.94	-0.001	0.000001
5543	1389191	4006037	745.551	745.55	-0.001	0.000001
100880	1338380	3952017	432.45	432.45	0	0
101238	1340441	3953546	432.91	432.91	0	0
100119	1358195	3962930	435.81	435.81	0	0
101333	1344484	3958869	436.85	436.85	0	0
100175	1358263	3965672	436.86	436.86	0	0
100354	1353090	3969060	444.43	444.43	0	0
103751	1373981	3960250	444.51	444.51	0	0
103978	1373717	3950754	448.42	448.42	0	0
104141	1372766	3947843	451.22	451.22	0	0
103982	1373714	3950673	451.68	451.68	0	0
104374	1373708	3950353	453.51	453.51	0	0
107613	1396175	3956627	456.25	456.25	0	0
107518	1399279	3960095	458.75	458.75	0	0
106144	1420900	3964950	470.461	470.46	0	0
106064	1420749	3958015	474.49	474.49	0	0
107010	1420556	3954717	474.94	474.94	0	0
101020	1334376	3947907	572.18	572.18	0	0
101258	1341691	3954976	433.029	433.03	0.001	0.000001
100758	1342277	3955668	433.079	433.08	0.001	0.000001

100764	1342096	3955460	433.279	433.28	0.001	0.000001
100700	1343832	3957730	434.449	434.45	0.001	0.000001
100959	1335767	3949964	434.789	434.79	0.001	0.000001
100057	1357934	3960210	436.699	436.7	0.001	0.000001
100538	1347087	3963677	440.219	440.22	0.001	0.000001
100521	1347455	3964353	446.729	446.73	0.001	0.000001
103711	1374068	3961899	449.109	449.11	0.001	0.000001
103443	1379601	3971731	450.979	450.98	0.001	0.000001
107559	1397290	3959134	458.919	458.92	0.001	0.000001
106129	1420885	3964311	470.949	470.95	0.001	0.000001
106892	1420771	3960133	474.729	474.73	0.001	0.000001
105984	1420306	3953984	475.909	475.91	0.001	0.000001
106706	1421838	3973771	478.319	478.32	0.001	0.000001
105679	1427388	3940525	487.659	487.66	0.001	0.000001
101055	1333293	3947250	543.419	543.42	0.001	0.000001
101028	1334074	3947718	560.669	560.67	0.001	0.000001
100735	1342963	3956454	433.028	433.03	0.002	0.000004
100046	1357499	3959915	436.748	436.75	0.002	0.000004
100628	1345307	3960375	436.918	436.92	0.002	0.000004
101353	1344968	3959733	437.007	437.01	0.002	0.000004
101373	1345436	3960566	437.008	437.01	0.002	0.000004
100183	1358272	3966018	437.878	437.88	0.002	0.000004
101585	1351783	3969068	450.788	450.79	0.002	0.000004
103394	1379602	3973124	462.318	462.32	0.002	0.000004
105986	1420294	3954018	475.748	475.75	0.002	0.000004
100784	1341529	3954812	432.807	432.81	0.003	0.000009
100776	1341753	3955068	433.107	433.11	0.003	0.000009
100103	1358172	3962240	436.667	436.67	0.003	0.000009
100600	1345860	3961355	436.777	436.78	0.003	0.000009
100063	1358091	3960495	436.867	436.87	0.003	0.000009
100654	1344777	3959420	436.947	436.95	0.003	0.000009
101760	1358382	3970642	439.477	439.48	0.003	0.000009
103695	1374327	3962636	450.067	450.07	0.003	0.000009
104255	1372980	3948197	451.717	451.72	0.003	0.000009
101825	1358238	3973502	454.427	454.43	0.003	0.000009
101505	1348705	3966512	458.217	458.22	0.003	0.000009
107642	1395748	3955179	458.827	458.83	0.003	0.000009
106036	1420723	3956608	474.867	474.87	0.003	0.000009
105987	1420295	3954038	475.357	475.36	0.003	0.000009
106978	1420697	3956158	475.537	475.54	0.003	0.000009
105409	1429061	3930613	496.897	496.9	0.003	0.000009
101018	1334442	3947966	573.417	573.42	0.003	0.000009
101274	1342132	3955482	433.026	433.03	0.004	0.000016
100147	1358216	3963861	434.906	434.91	0.004	0.000016
101349	1344829	3959487	437.106	437.11	0.004	0.000016
101758	1358380	3970556	439.806	439.81	0.004	0.000016
103911	1373779	3953015	447.366	447.37	0.004	0.000016
101486	1348233	3965668	455.806	455.81	0.004	0.000016
101558	1349945	3968517	456.096	456.1	0.004	0.000016
100463	1348680	3966546	458.346	458.35	0.004	0.000016
107566	1397008	3958871	459.646	459.65	0.004	0.000016
106159	1420912	3965621	470.926	470.93	0.004	0.000016

106822	1420914	3966755	472.276	472.28	0.004	0.000016
101119	1335113	3949268	473.656	473.66	0.004	0.000016
105695	1426660	3941078	488.066	488.07	0.004	0.000016
101215	1338441	3952030	432.475	432.48	0.005	0.000025
101254	1341483	3954738	432.815	432.82	0.005	0.000025
100914	1337124	3951344	433.385	433.39	0.005	0.000025
100967	1335525	3949712	437.825	437.83	0.005	0.000025
103655	1374932	3964127	445.575	445.58	0.005	0.000025
103729	1374035	3961073	446.055	446.06	0.005	0.000025
101466	1347710	3964733	453.125	453.13	0.005	0.000025
103991	1373699	3950486	453.715	453.72	0.005	0.000025
107632	1395908	3955725	456.955	456.96	0.005	0.000025
100447	1349024	3967162	460.125	460.13	0.005	0.000025
106149	1420905	3965192	470.485	470.49	0.005	0.000025
106714	1421830	3973360	476.315	476.32	0.005	0.000025
105640	1428805	3939298	489.885	489.89	0.005	0.000025
101241	1340522	3953638	432.844	432.85	0.006	0.000036
100792	1341308	3954559	432.924	432.93	0.006	0.000036
100144	1358213	3963751	434.794	434.8	0.006	0.000036
103578	1376003	3967890	443.964	443.97	0.006	0.000036
103643	1374958	3964595	445.184	445.19	0.006	0.000036
104401	1373732	3950914	446.454	446.46	0.006	0.000036
103954	1373735	3951418	446.654	446.66	0.006	0.000036
104396	1373729	3950800	447.374	447.38	0.006	0.000036
103602	1375412	3967074	449.904	449.91	0.006	0.000036
103617	1375019	3965737	450.824	450.83	0.006	0.000036
104193	1372478	3947332	451.134	451.14	0.006	0.000036
101474	1347914	3965099	454.084	454.09	0.006	0.000036
101482	1348129	3965482	455.244	455.25	0.006	0.000036
107533	1398497	3959746	457.814	457.82	0.006	0.000036
100416	1349733	3968359	459.024	459.03	0.006	0.000036
107474	1401366	3960995	460.244	460.25	0.006	0.000036
106854	1420885	3964890	470.374	470.38	0.006	0.000036
106238	1421791	3970264	472.164	472.17	0.006	0.000036
106933	1420736	3958193	474.204	474.21	0.006	0.000036
106059	1420744	3957725	474.684	474.69	0.006	0.000036
106940	1420731	3957857	474.734	474.74	0.006	0.000036
105927	1421582	3951519	476.934	476.94	0.006	0.000036
105455	1429151	3932083	494.884	494.89	0.006	0.000036
100741	1342787	3956251	433.033	433.04	0.007	0.000049
100071	1358138	3960859	436.453	436.46	0.007	0.000049
100603	1345796	3961239	436.823	436.83	0.007	0.000049
101552	1349774	3968336	456.803	456.81	0.007	0.000049
101120	1335144	3949300	469.743	469.75	0.007	0.000049
101085	1334348	3947869	571.453	571.46	0.007	0.000049
101174	1336869	3951085	433.002	433.01	0.008	0.000064
100092	1358160	3961765	435.302	435.31	0.008	0.000064
100038	1357286	3959808	436.272	436.28	0.008	0.000064
101382	1345599	3960849	436.862	436.87	0.008	0.000064
100968	1335494	3949680	438.712	438.72	0.008	0.000064
103575	1376085	3967998	444.402	444.41	0.008	0.000064
104438	1373750	3951693	446.672	446.68	0.008	0.000064

104487	1373805	3953419	447.462	447.47	0.008	0.000064
100396	1351272	3969116	453.402	453.41	0.008	0.000064
101546	1349621	3968136	457.782	457.79	0.008	0.000064
107596	1396415	3957462	458.182	458.19	0.008	0.000064
106197	1421329	3967963	466.732	466.74	0.008	0.000064
106795	1421211	3967802	469.672	469.68	0.008	0.000064
106929	1420740	3958378	473.552	473.56	0.008	0.000064
105996	1420544	3954620	475.702	475.71	0.008	0.000064
105710	1425833	3941408	485.642	485.65	0.008	0.000064
105720	1425219	3941592	485.692	485.7	0.008	0.000064
100822	1340447	3953572	432.821	432.83	0.009	0.000081
100749	1342551	3955980	433.101	433.11	0.009	0.000081
100247	1358341	3968717	437.351	437.36	0.009	0.000081
103574	1376113	3968036	444.721	444.73	0.009	0.000081
103742	1374023	3960558	445.571	445.58	0.009	0.000081
100973	1335341	3949523	447.881	447.89	0.009	0.000081
103712	1374063	3961851	449.151	449.16	0.009	0.000081
106748	1421795	3971641	470.931	470.94	0.009	0.000081
105943	1421340	3952304	474.991	475	0.009	0.000081
105839	1423437	3947186	476.541	476.55	0.009	0.000081
105802	1424073	3945551	476.541	476.55	0.009	0.000081
103375	1379609	3973965	477.391	477.4	0.009	0.000081
100895	1337812	3951767	432.92	432.93	0.01	0.0001
101269	1341999	3955328	433	433.01	0.01	0.0001
100148	1358217	3963898	434.94	434.95	0.01	0.0001
100584	1346209	3961975	436.75	436.76	0.01	0.0001
100573	1346429	3962374	436.94	436.95	0.01	0.0001
104512	1373836	3954637	446.34	446.35	0.01	0.0001
104184	1372411	3947277	450.98	450.99	0.01	0.0001
107537	1398307	3959659	457.62	457.63	0.01	0.0001
107532	1398576	3959781	458.05	458.06	0.01	0.0001
107509	1399716	3960298	459.03	459.04	0.01	0.0001
106859	1420879	3964645	470.481	470.49	0.01	0.0001
100788	1341418	3954685	432.709	432.72	0.011	0.000121
100705	1343726	3957542	433.829	433.84	0.011	0.000121
101141	1335829	3950009	434.319	434.33	0.011	0.000121
100667	1344500	3958925	436.83	436.84	0.011	0.000121
101328	1344380	3958683	436.909	436.92	0.011	0.000121
101417	1346405	3962295	437.159	437.17	0.011	0.000121
100187	1358276	3966184	439.929	439.94	0.011	0.000121
103470	1379410	3970679	450.799	450.81	0.011	0.000121
103439	1379602	3971824	451.839	451.85	0.011	0.000121
107633	1395884	3955644	457.579	457.59	0.011	0.000121
101538	1349461	3967862	459.199	459.21	0.011	0.000121
105806	1424047	3945765	476.169	476.18	0.011	0.000121
105605	1429968	3938254	491.059	491.07	0.011	0.000121
105553	1430125	3935601	493.759	493.77	0.011	0.000121
5529	1388664	4005504	738.759	738.77	0.011	0.000121
101203	1337960	3951814	432.348	432.36	0.012	0.000144
100886	1338148	3951915	432.558	432.57	0.012	0.000144
101267	1341945	3955267	432.928	432.94	0.012	0.000144
103625	1374998	3965353	445.778	445.79	0.012	0.000144

103719	1374046	3961491	446.528	446.54	0.012	0.000144
103703	1374163	3962286	450.138	450.15	0.012	0.000144
104150	1372687	3947707	450.798	450.81	0.012	0.000144
104146	1372721	3947767	451.048	451.06	0.012	0.000144
101481	1348086	3965406	454.858	454.87	0.012	0.000144
100487	1348181	3965654	455.908	455.92	0.012	0.000144
106272	1421809	3971481	469.788	469.8	0.012	0.000144
106077	1420761	3958700	472.708	472.72	0.012	0.000144
106958	1420714	3957050	474.998	475.01	0.012	0.000144
100817	1340591	3953737	432.877	432.89	0.013	0.000169
100795	1341224	3954464	433.047	433.06	0.013	0.000169
100911	1337228	3951426	433.297	433.31	0.013	0.000169
100127	1358205	3963222	435.347	435.36	0.013	0.000169
100663	1344583	3959074	436.867	436.88	0.013	0.000169
100169	1358240	3964777	437.417	437.43	0.013	0.000169
101804	1358387	3972480	444.447	444.46	0.013	0.000169
100486	1348202	3965691	456.127	456.14	0.013	0.000169
101521	1349107	3967233	460.387	460.4	0.013	0.000169
101832	1358072	3973772	461.277	461.29	0.013	0.000169
106218	1421739	3968910	470.357	470.37	0.013	0.000169
101848	1357593	3974211	481.187	481.2	0.013	0.000169
105616	1429562	3938642	490.107	490.12	0.013	0.000169
105626	1429278	3938889	490.457	490.47	0.013	0.000169
105606	1429939	3938286	491.107	491.12	0.013	0.000169
105441	1429144	3931459	496.187	496.2	0.013	0.000169
101053	1333249	3947224	545.377	545.39	0.013	0.000169
101029	1334037	3947697	559.517	559.53	0.013	0.000169
100089	1358157	3961640	435.066	435.08	0.014	0.000196
100642	1345039	3959890	436.826	436.84	0.014	0.000196
100567	1346621	3962743	436.926	436.94	0.014	0.000196
100245	1358341	3968669	437.217	437.23	0.014	0.000196
101743	1358364	3969934	437.666	437.68	0.014	0.000196
103651	1374941	3964279	445.147	445.16	0.014	0.000196
104498	1373818	3953962	447.226	447.24	0.014	0.000196
103547	1377017	3968792	447.376	447.39	0.014	0.000196
103453	1379597	3971395	449.396	449.41	0.014	0.000196
103701	1374200	3962378	450.196	450.21	0.014	0.000196
101592	1352089	3969071	450.756	450.77	0.014	0.000196
101559	1350759	3968982	453.016	453.03	0.014	0.000196
100501	1347886	3965124	454.276	454.29	0.014	0.000196
101524	1349193	3967388	460.336	460.35	0.014	0.000196
106156	1420908	3965477	470.686	470.7	0.014	0.000196
106170	1420921	3966125	472.466	472.48	0.014	0.000196
105932	1421499	3951804	476.136	476.15	0.014	0.000196
101019	1334410	3947936	572.946	572.96	0.014	0.000196
100114	1358187	3962721	436.265	436.28	0.015	0.000225
100069	1358136	3960771	436.725	436.74	0.015	0.000225
101380	1345565	3960789	436.895	436.91	0.015	0.000225
100353	1353134	3969059	444.515	444.53	0.015	0.000225
103705	1374131	3962191	449.835	449.85	0.015	0.000225
103420	1379603	3972163	454.105	454.12	0.015	0.000225
106147	1420902	3965087	470.515	470.53	0.015	0.000225

106937	1420733	3958008	474.585	474.6	0.015	0.000225
105968	1420796	3953534	475.275	475.29	0.015	0.000225
105938	1421417	3952071	475.485	475.5	0.015	0.000225
106715	1421827	3973276	475.825	475.84	0.015	0.000225
105804	1424058	3945680	476.145	476.16	0.015	0.000225
105445	1429147	3931645	496.185	496.2	0.015	0.000225
5546	1389306	4006153	747.245	747.26	0.015	0.000225
100050	1357651	3959992	436.344	436.36	0.016	0.000256
100597	1345926	3961471	436.814	436.83	0.016	0.000256
101594	1352179	3969068	450.344	450.36	0.016	0.000256
100481	1348306	3965876	456.444	456.46	0.016	0.000256
107623	1396038	3956164	457.134	457.15	0.016	0.000256
105894	1422104	3949858	474.044	474.06	0.016	0.000256
106045	1420732	3957031	474.764	474.78	0.016	0.000256
105563	1430345	3936057	494.394	494.41	0.016	0.000256
100863	1339095	3952338	434.163	434.18	0.017	0.000289
101413	1346300	3962103	436.723	436.74	0.017	0.000289
103722	1374043	3961364	446.273	446.29	0.017	0.000289
103549	1376943	3968746	447.233	447.25	0.017	0.000289
103488	1378892	3970035	451.903	451.92	0.017	0.000289
101542	1349534	3967993	459.023	459.04	0.017	0.000289
101841	1357805	3974036	473.403	473.42	0.017	0.000289
101140	1335797	3949976	434.622	434.64	0.018	0.000324
100146	1358215	3963824	434.902	434.92	0.018	0.000324
101741	1358361	3969538	436.002	436.02	0.018	0.000324
101410	1346236	3961989	436.692	436.71	0.018	0.000324
100163	1358232	3964496	436.812	436.83	0.018	0.000324
101619	1353261	3969027	445.232	445.25	0.018	0.000324
103652	1374939	3964240	445.232	445.25	0.018	0.000324
103847	1373848	3956019	445.772	445.79	0.018	0.000324
103560	1376544	3968481	445.852	445.87	0.018	0.000324
104433	1373747	3951588	446.272	446.29	0.018	0.000324
103548	1376980	3968769	447.322	447.34	0.018	0.000324
104388	1373722	3950624	453.572	453.59	0.018	0.000324
100468	1348575	3966359	457.782	457.8	0.018	0.000324
106833	1420903	3965961	472.512	472.53	0.018	0.000324
106830	1420910	3966442	472.642	472.66	0.018	0.000324
106962	1420710	3956863	474.922	474.94	0.018	0.000324
106306	1421844	3973245	475.792	475.81	0.018	0.000324
105921	1421673	3951233	477.422	477.44	0.018	0.000324
105620	1429438	3938748	490.142	490.16	0.018	0.000324
105624	1429326	3938846	490.342	490.36	0.018	0.000324
105476	1429121	3932964	495.372	495.39	0.018	0.000324
100207	1358298	3967044	435.951	435.97	0.019	0.000361
103927	1373754	3952293	446.051	446.07	0.019	0.000361
100369	1352437	3969072	448.521	448.54	0.019	0.000361
100375	1352162	3969081	450.261	450.28	0.019	0.000361
104382	1373717	3950510	453.651	453.67	0.019	0.000361
100488	1348160	3965616	455.751	455.77	0.019	0.000361
101488	1348273	3965741	456.051	456.07	0.019	0.000361
107597	1396393	3957381	458.171	458.19	0.019	0.000361
106179	1420938	3966905	471.661	471.68	0.019	0.000361

106060	1420746	3957809	474.651	474.67	0.019	0.000361
105612	1429698	3938522	490.221	490.24	0.019	0.000361
100870	1338797	3952200	433.26	433.28	0.02	0.0004
100067	1358130	3960682	436.87	436.89	0.02	0.0004
101745	1358366	3970020	438.15	438.17	0.02	0.0004
103516	1377935	3969390	448.64	448.66	0.02	0.0004
103498	1378517	3969780	451.76	451.78	0.02	0.0004
101467	1347731	3964770	453.2	453.22	0.02	0.0004
100494	1348034	3965389	455.02	455.04	0.02	0.0004
106282	1421819	3971987	471.66	471.68	0.02	0.0004
106926	1420742	3958533	473.18	473.2	0.02	0.0004
107004	1420607	3954898	475.26	475.28	0.02	0.0004
105962	1420977	3953261	475.48	475.5	0.02	0.0004
105911	1421840	3950722	477.03	477.05	0.02	0.0004
105611	1429758	3938466	490.48	490.5	0.02	0.0004
105492	1429265	3933415	493.56	493.58	0.02	0.0004
100701	1343811	3957692	434.339	434.36	0.021	0.000441
100572	1346446	3962404	436.899	436.92	0.021	0.000441
101768	1358388	3970958	438.479	438.5	0.021	0.000441
103468	1379448	3970763	450.539	450.56	0.021	0.000441
103987	1373706	3950569	454.099	454.12	0.021	0.000441
100432	1349347	3967745	459.479	459.5	0.021	0.000441
106216	1421718	3968800	469.119	469.14	0.021	0.000441
105799	1424087	3945378	476.879	476.9	0.021	0.000441
100780	1341641	3954940	433.058	433.08	0.022	0.000484
100054	1357817	3960096	436.598	436.62	0.022	0.000484
101324	1344298	3958537	436.709	436.73	0.022	0.000484
101441	1347061	3963575	439.848	439.87	0.022	0.000484
100188	1358277	3966225	440.478	440.5	0.022	0.000484
104230	1372772	3947830	451.338	451.36	0.022	0.000484
104365	1373699	3950167	453.698	453.72	0.022	0.000484
103986	1373708	3950589	453.948	453.97	0.022	0.000484
107611	1396208	3956739	456.558	456.58	0.022	0.000484
107647	1395674	3954926	458.228	458.25	0.022	0.000484
103403	1379603	3972754	459.118	459.14	0.022	0.000484
107572	1396837	3958648	460.068	460.09	0.022	0.000484
107487	1400784	3960771	460.538	460.56	0.022	0.000484
106799	1421137	3967669	470.828	470.85	0.022	0.000484
105680	1427325	3940577	487.758	487.78	0.022	0.000484
5524	1388467	4005315	735.988	736.01	0.022	0.000484
100807	1340881	3954071	432.777	432.8	0.023	0.000529
100676	1344314	3958595	436.777	436.8	0.023	0.000529
101327	1344360	3958646	436.897	436.92	0.023	0.000529
100352	1353179	3969057	444.627	444.65	0.023	0.000529
103995	1373693	3950403	453.537	453.56	0.023	0.000529
101121	1335174	3949333	465.877	465.9	0.023	0.000529
106807	1421010	3967374	471.147	471.17	0.023	0.000529
106967	1420702	3956620	474.997	475.02	0.023	0.000529
105690	1426898	3940921	488.337	488.36	0.023	0.000529
105483	1429194	3933203	494.357	494.38	0.023	0.000529
101089	1334473	3947983	574.057	574.08	0.023	0.000529
100757	1342308	3955703	433.126	433.15	0.024	0.000576

101136	1335669	3949843	435.697	435.72	0.024	0.000576
100115	1358189	3962763	436.176	436.2	0.024	0.000576
100594	1345990	3961584	436.836	436.86	0.024	0.000576
101388	1345726	3961078	436.936	436.96	0.024	0.000576
103949	1373736	3951558	446.066	446.09	0.024	0.000576
103912	1373777	3952976	447.386	447.41	0.024	0.000576
107605	1396282	3956992	456.256	456.28	0.024	0.000576
103398	1379602	3972956	460.776	460.8	0.024	0.000576
106968	1420702	3956587	474.896	474.92	0.024	0.000576
106965	1420705	3956721	475.087	475.11	0.024	0.000576
105862	1422719	3948193	478.486	478.51	0.024	0.000576
105688	1426974	3940865	488.436	488.46	0.024	0.000576
105632	1429070	3939069	490.226	490.25	0.024	0.000576
101060	1333437	3947334	540.437	540.46	0.024	0.000576
100885	1338186	3951932	432.605	432.63	0.025	0.000625
100135	1358212	3963438	434.955	434.98	0.025	0.000625
104425	1373744	3951425	446.645	446.67	0.025	0.000625
103983	1373713	3950653	452.485	452.51	0.025	0.000625
103434	1379601	3971895	452.485	452.51	0.025	0.000625
100975	1335281	3949460	452.935	452.96	0.025	0.000625
107630	1395932	3955806	456.655	456.68	0.025	0.000625
107563	1397124	3958990	459.345	459.37	0.025	0.000625
107587	1396545	3957928	459.495	459.52	0.025	0.000625
106856	1420883	3964807	470.305	470.33	0.025	0.000625
106163	1420915	3965799	471.725	471.75	0.025	0.000625
105944	1421313	3952384	474.785	474.81	0.025	0.000625
105879	1422347	3949082	476.675	476.7	0.025	0.000625
105665	1427877	3940103	488.295	488.32	0.025	0.000625
105466	1429081	3932564	495.915	495.94	0.025	0.000625
5551	1389498	4006349	749.535	749.56	0.025	0.000625
100659	1344667	3959225	437.164	437.19	0.026	0.000676
104446	1373755	3951911	445.924	445.95	0.026	0.000676
107635	1395861	3955561	457.394	457.42	0.026	0.000676
107594	1396437	3957546	458.354	458.38	0.026	0.000676
106878	1420855	3963648	473.394	473.42	0.026	0.000676
106107	1420790	3960205	474.854	474.88	0.026	0.000676
101850	1357531	3974262	482.614	482.64	0.026	0.000676
105683	1427225	3940661	487.744	487.77	0.026	0.000676
105660	1428060	3939944	488.514	488.54	0.026	0.000676
101244	1340684	3953824	432.963	432.99	0.027	0.000729
100731	1343077	3956587	433.163	433.19	0.027	0.000729
100698	1343873	3957804	434.693	434.72	0.027	0.000729
100571	1346461	3962433	436.853	436.88	0.027	0.000729
103749	1374003	3960314	444.723	444.75	0.027	0.000729
107556	1397433	3959235	458.883	458.91	0.027	0.000729
107569	1396905	3958747	459.903	459.93	0.027	0.000729
107496	1400387	3960603	460.163	460.19	0.027	0.000729
107478	1401177	3960924	460.173	460.2	0.027	0.000729
106855	1420884	3964848	470.343	470.37	0.027	0.000729
106127	1420882	3964204	471.273	471.3	0.027	0.000729
106034	1420720	3956489	474.973	475	0.027	0.000729
105888	1422193	3949574	475.003	475.03	0.027	0.000729

106093	1420776	3959524	475.163	475.19	0.027	0.000729
105619	1429486	3938708	490.083	490.11	0.027	0.000729
105558	1430239	3935830	493.873	493.9	0.027	0.000729
105447	1429149	3931721	495.983	496.01	0.027	0.000729
101057	1333346	3947281	541.573	541.6	0.027	0.000729
101250	1341241	3954460	433.002	433.03	0.028	0.000784
100096	1358164	3961933	435.802	435.83	0.028	0.000784
100223	1358316	3967756	436.652	436.68	0.028	0.000784
100635	1345174	3960139	436.682	436.71	0.028	0.000784
100060	1358028	3960350	436.752	436.78	0.028	0.000784
100541	1347028	3963555	440.032	440.06	0.028	0.000784
101473	1347873	3965025	453.852	453.88	0.028	0.000784
107672	1395280	3953590	460.682	460.71	0.028	0.000784
106161	1420914	3965728	471.332	471.36	0.028	0.000784
105847	1423200	3947502	476.712	476.74	0.028	0.000784
105693	1426739	3941029	488.292	488.32	0.028	0.000784
105636	1428940	3939182	490.042	490.07	0.028	0.000784
101183	1337164	3951359	432.851	432.88	0.029	0.000841
101232	1340277	3953359	432.932	432.96	0.029	0.000841
100763	1342125	3955493	433.181	433.21	0.029	0.000841
100142	1358211	3963676	434.751	434.78	0.029	0.000841
100153	1358222	3964082	435.161	435.19	0.029	0.000841
101420	1346466	3962409	437.071	437.1	0.029	0.000841
100559	1346695	3962903	437.132	437.16	0.029	0.000841
100658	1344689	3959263	437.141	437.17	0.029	0.000841
100198	1358288	3966646	439.541	439.57	0.029	0.000841
103903	1373789	3953328	446.962	446.99	0.029	0.000841
103414	1379603	3972356	455.351	455.38	0.029	0.000841
106140	1420896	3964773	470.361	470.39	0.029	0.000841
106999	1420650	3955110	475.511	475.54	0.029	0.000841
105934	1421476	3951882	475.931	475.96	0.029	0.000841
105735	1424463	3941951	486.381	486.41	0.029	0.000841
101035	1333816	3947569	551.351	551.38	0.029	0.000841
101083	1334275	3947822	568.371	568.4	0.029	0.000841
100752	1342460	3955878	433.11	433.14	0.03	0.0009
101360	1345146	3960050	436.82	436.85	0.03	0.0009
100045	1357466	3959899	436.83	436.86	0.03	0.0009
100249	1358342	3968753	437.32	437.35	0.03	0.0009
103506	1378237	3969594	450.78	450.81	0.03	0.0009
103499	1378482	3969757	451.58	451.61	0.03	0.0009
104013	1373682	3950054	453.62	453.65	0.03	0.0009
107512	1399599	3960243	458.92	458.95	0.03	0.0009
103389	1379603	3973337	465.03	465.06	0.03	0.0009
106124	1420879	3964061	471.79	471.82	0.03	0.0009
105886	1422232	3949452	475.44	475.47	0.03	0.0009
105842	1423348	3947308	476.25	476.28	0.03	0.0009
105615	1429615	3938597	490.08	490.11	0.03	0.0009
100879	1338420	3952035	432.509	432.54	0.031	0.000961
101272	1342079	3955420	433.099	433.13	0.031	0.000961
100631	1345250	3960276	436.729	436.76	0.031	0.000961
100625	1345360	3960471	437.099	437.13	0.031	0.000961
101438	1346988	3963427	439.029	439.06	0.031	0.000961

101754	1358375	3970389	440.199	440.23	0.031	0.000961
100351	1353224	3969056	444.809	444.84	0.031	0.000961
100522	1347433	3964314	445.539	445.57	0.031	0.000961
103671	1374812	3963539	447.619	447.65	0.031	0.000961
103456	1379585	3971272	449.339	449.37	0.031	0.000961
104003	1373687	3950243	453.519	453.55	0.031	0.000961
107485	1400855	3960799	460.599	460.63	0.031	0.000961
106030	1420716	3956285	475.339	475.37	0.031	0.000961
105439	1429142	3931354	495.799	495.83	0.031	0.000961
5545	1389268	4006115	746.769	746.8	0.031	0.000961
100894	1337850	3951784	432.818	432.85	0.032	0.001024
101280	1342302	3955676	432.998	433.03	0.032	0.001024
100756	1342339	3955738	433.198	433.23	0.032	0.001024
100141	1358212	3963638	434.778	434.81	0.032	0.001024
100149	1358218	3963935	434.948	434.98	0.032	0.001024
100588	1346120	3961816	436.828	436.86	0.032	0.001024
101133	1335575	3949746	436.968	437	0.032	0.001024
100246	1358341	3968695	437.288	437.32	0.032	0.001024
103910	1373780	3953054	447.298	447.33	0.032	0.001024
103507	1378202	3969571	450.618	450.65	0.032	0.001024
103492	1378742	3969927	452.038	452.07	0.032	0.001024
101464	1347648	3964622	452.138	452.17	0.032	0.001024
107652	1395596	3954659	458.348	458.38	0.032	0.001024
107469	1401588	3961079	460.238	460.27	0.032	0.001024
105659	1428122	3939891	488.538	488.57	0.032	0.001024
105590	1430386	3937550	491.828	491.86	0.032	0.001024
100162	1358231	3964450	436.807	436.84	0.033	0.001089
100607	1345712	3961088	436.887	436.92	0.033	0.001089
101779	1358364	3971372	439.637	439.67	0.033	0.001089
103567	1376319	3968281	446.257	446.29	0.033	0.001089
103721	1374044	3961406	446.307	446.34	0.033	0.001089
103490	1378818	3969979	451.887	451.92	0.033	0.001089
107522	1399071	3960001	458.547	458.58	0.033	0.001089
106126	1420881	3964168	471.378	471.41	0.033	0.001089
106917	1420751	3958957	473.227	473.26	0.033	0.001089
105479	1429158	3933085	495.027	495.06	0.033	0.001089
101076	1334002	3947663	558.037	558.07	0.033	0.001089
100934	1336509	3950733	432.826	432.86	0.034	0.001156
101235	1340359	3953453	432.876	432.91	0.034	0.001156
100093	1358161	3961807	435.346	435.38	0.034	0.001156
100661	1344625	3959149	437.106	437.14	0.034	0.001156
103663	1374897	3963838	446.486	446.52	0.034	0.001156
104232	1372788	3947858	451.456	451.49	0.034	0.001156
104375	1373709	3950373	453.526	453.56	0.034	0.001156
100492	1348076	3965464	455.286	455.32	0.034	0.001156
100445	1349067	3967239	460.336	460.37	0.034	0.001156
106203	1421470	3968202	463.566	463.6	0.034	0.001156
106758	1421787	3971154	469.936	469.97	0.034	0.001156
106847	1420892	3965256	470.486	470.52	0.034	0.001156
105490	1429252	3933367	493.816	493.85	0.034	0.001156
100070	1358137	3960815	436.535	436.57	0.035	0.001225
100672	1344397	3958741	436.905	436.94	0.035	0.001225

103500	1378447	3969734	451.385	451.42	0.035	0.001225
103408	1379603	3972569	457.435	457.47	0.035	0.001225
106192	1421198	3967742	469.925	469.96	0.035	0.001225
106252	1421806	3970636	471.705	471.74	0.035	0.001225
106996	1420669	3955263	475.325	475.36	0.035	0.001225
106312	1421851	3973591	477.875	477.91	0.035	0.001225
105700	1426370	3941225	487.695	487.73	0.035	0.001225
100727	1343189	3956726	433.074	433.11	0.036	0.001296
100755	1342369	3955773	433.104	433.14	0.036	0.001296
100095	1358163	3961891	435.675	435.71	0.036	0.001296
101429	1346796	3963039	438.084	438.12	0.036	0.001296
100550	1346847	3963186	438.434	438.47	0.036	0.001296
103952	1373735	3951474	446.604	446.64	0.036	0.001296
103957	1373733	3951333	446.754	446.79	0.036	0.001296
103441	1379601	3971782	451.464	451.5	0.036	0.001296
107591	1396481	3957715	458.704	458.74	0.036	0.001296
107513	1399520	3960206	458.765	458.8	0.036	0.001296
107568	1396954	3958811	459.814	459.85	0.036	0.001296
106050	1420736	3957265	474.664	474.7	0.036	0.001296
105691	1426859	3940949	488.344	488.38	0.036	0.001296
105613	1429669	3938548	490.154	490.19	0.036	0.001296
5539	1389043	4005887	743.524	743.56	0.036	0.001296
100773	1341837	3955165	433.023	433.06	0.037	0.001369
100145	1358214	3963788	434.863	434.9	0.037	0.001369
100130	1358208	3963308	435.223	435.26	0.037	0.001369
100073	1358139	3960948	436.503	436.54	0.037	0.001369
100059	1358001	3960302	436.743	436.78	0.037	0.001369
101389	1345742	3961107	436.933	436.97	0.037	0.001369
100675	1344335	3958631	436.933	436.97	0.037	0.001369
100535	1347146	3963796	440.543	440.58	0.037	0.001369
103591	1375686	3967471	442.733	442.77	0.037	0.001369
104505	1373827	3954282	447.233	447.27	0.037	0.001369
104393	1373726	3950730	449.163	449.2	0.037	0.001369
104378	1373712	3950434	453.523	453.56	0.037	0.001369
104000	1373689	3950302	453.553	453.59	0.037	0.001369
103994	1373694	3950424	453.563	453.6	0.037	0.001369
100440	1349176	3967434	460.223	460.26	0.037	0.001369
106185	1421075	3967493	470.183	470.22	0.037	0.001369
106851	1420888	3965053	470.453	470.49	0.037	0.001369
105900	1422004	3950182	474.663	474.7	0.037	0.001369
106047	1420733	3957109	474.693	474.73	0.037	0.001369
106955	1420716	3957163	474.853	474.89	0.037	0.001369
105863	1422696	3948233	478.553	478.59	0.037	0.001369
105868	1422552	3948517	478.583	478.62	0.037	0.001369
105715	1425553	3941494	485.633	485.67	0.037	0.001369
103562	1376476	3968427	446.042	446.08	0.038	0.001444
103906	1373785	3953210	446.882	446.92	0.038	0.001444
101463	1347627	3964585	451.802	451.84	0.038	0.001444
103438	1379602	3971842	452.082	452.12	0.038	0.001444
106056	1420742	3957596	474.722	474.76	0.038	0.001444
105732	1424577	3941869	486.282	486.32	0.038	0.001444
105530	1429712	3934798	493.292	493.33	0.038	0.001444

105525	1429611	3934523	493.721	493.76	0.038	0.001444
101150	1336115	3950307	433.021	433.06	0.039	0.001521
100725	1343244	3956796	433.041	433.08	0.039	0.001521
101139	1335765	3949943	434.791	434.83	0.039	0.001521
100055	1357857	3960130	436.671	436.71	0.039	0.001521
103583	1375890	3967739	443.271	443.31	0.039	0.001521
103878	1373817	3954420	446.881	446.92	0.039	0.001521
107637	1395823	3955435	457.921	457.96	0.039	0.001521
103406	1379603	3972641	458.011	458.05	0.039	0.001521
107507	1399834	3960353	459.041	459.08	0.039	0.001521
106818	1420921	3966920	472.141	472.18	0.039	0.001521
106080	1420764	3958871	472.921	472.96	0.039	0.001521
106902	1420764	3959734	475.041	475.08	0.039	0.001521
106029	1420715	3956245	475.341	475.38	0.039	0.001521
105473	1429095	3932868	495.491	495.53	0.039	0.001521
101065	1333596	3947427	543.231	543.27	0.039	0.001521
5549	1389421	4006270	748.671	748.71	0.039	0.001521
101184	1337200	3951387	432.91	432.95	0.04	0.0016
100766	1342038	3955394	433.31	433.35	0.04	0.0016
100110	1358181	3962548	436.52	436.56	0.04	0.0016
101785	1358362	3971625	440.76	440.8	0.04	0.0016
100971	1335402	3949586	443.43	443.47	0.04	0.0016
103559	1376579	3968507	445.88	445.92	0.04	0.0016
103730	1374034	3961031	445.92	445.96	0.04	0.0016
103888	1373805	3953962	447.18	447.22	0.04	0.0016
107520	1399195	3960057	458.66	458.7	0.04	0.0016
106112	1420865	3963498	473.75	473.79	0.04	0.0016
106062	1420747	3957891	474.59	474.63	0.04	0.0016
106020	1420707	3955796	475.27	475.31	0.04	0.0016
106992	1420682	3955457	475.42	475.46	0.04	0.0016
101260	1341749	3955043	432.899	432.94	0.041	0.001681
100745	1342669	3956116	433.059	433.1	0.041	0.001681
100074	1358140	3960991	436.409	436.45	0.041	0.001681
100627	1345325	3960407	437.039	437.08	0.041	0.001681
100202	1358292	3966821	437.259	437.3	0.041	0.001681
104435	1373748	3951628	446.569	446.61	0.041	0.001681
104490	1373810	3953592	447.289	447.33	0.041	0.001681
103522	1377820	3969313	447.889	447.93	0.041	0.001681
104394	1373727	3950754	448.399	448.44	0.041	0.001681
101600	1352448	3969059	448.499	448.54	0.041	0.001681
103445	1379602	3971674	450.589	450.63	0.041	0.001681
100512	1347650	3964702	452.939	452.98	0.041	0.001681
104381	1373715	3950491	453.609	453.65	0.041	0.001681
103395	1379602	3973081	461.839	461.88	0.041	0.001681
106835	1420901	3965849	472.139	472.18	0.041	0.001681
106901	1420764	3959771	474.939	474.98	0.041	0.001681
101277	1342216	3955577	432.948	432.99	0.042	0.001764
101362	1345189	3960127	436.908	436.95	0.042	0.001764
101340	1344633	3959134	436.938	436.98	0.042	0.001764
101354	1344991	3959774	437.018	437.06	0.042	0.001764
100525	1347366	3964194	443.158	443.2	0.042	0.001764
103974	1373721	3950852	446.858	446.9	0.042	0.001764

103526	1377633	3969193	447.688	447.73	0.042	0.001764
103678	1374701	3963297	448.468	448.51	0.042	0.001764
103603	1375389	3967033	450.838	450.88	0.042	0.001764
101582	1351654	3969071	451.008	451.05	0.042	0.001764
100393	1351400	3969119	452.858	452.9	0.042	0.001764
103396	1379602	3973039	461.378	461.42	0.042	0.001764
106700	1421842	3974069	479.418	479.46	0.042	0.001764
106691	1421851	3974544	481.968	482.01	0.042	0.001764
105733	1424539	3941895	486.368	486.41	0.042	0.001764
5526	1388548	4005390	737.248	737.29	0.042	0.001764
101213	1338358	3951993	432.507	432.55	0.043	0.001849
100801	1341054	3954269	432.717	432.76	0.043	0.001849
101755	1358376	3970430	440.177	440.22	0.043	0.001849
101578	1351487	3969073	451.437	451.48	0.043	0.001849
104082	1373314	3948810	452.317	452.36	0.043	0.001849
107610	1396219	3956776	456.667	456.71	0.043	0.001849
106215	1421709	3968763	468.597	468.64	0.043	0.001849
106182	1421037	3967395	470.527	470.57	0.043	0.001849
106117	1420873	3963766	472.807	472.85	0.043	0.001849
106914	1420754	3959123	473.957	474	0.043	0.001849
107018	1420314	3954133	474.597	474.64	0.043	0.001849
106103	1420786	3959998	475.047	475.09	0.043	0.001849
106990	1420686	3955574	475.227	475.27	0.043	0.001849
105808	1424028	3945887	476.097	476.14	0.043	0.001849
105864	1422653	3948313	478.747	478.79	0.043	0.001849
106326	1421866	3974330	480.757	480.8	0.043	0.001849
101048	1333141	3947134	550.927	550.97	0.043	0.001849
100887	1338110	3951899	432.466	432.51	0.044	0.001936
100614	1345571	3960836	436.936	436.98	0.044	0.001936
101368	1345314	3960352	437.086	437.13	0.044	0.001936
103737	1374026	3960746	445.676	445.72	0.044	0.001936
101823	1358274	3973419	453.416	453.46	0.044	0.001936
106848	1420891	3965215	470.476	470.52	0.044	0.001936
106150	1420906	3965227	470.486	470.53	0.044	0.001936
106925	1420743	3958570	473.036	473.08	0.044	0.001936
105557	1430220	3935792	493.846	493.89	0.044	0.001936
101058	1333375	3947298	540.996	541.04	0.044	0.001936
101214	1338400	3952012	432.486	432.53	0.045	0.002025
100601	1345839	3961316	436.805	436.85	0.045	0.002025
100370	1352392	3969073	448.705	448.75	0.045	0.002025
106935	1420734	3958082	474.225	474.27	0.045	0.002025
101167	1336658	3950868	432.614	432.66	0.046	0.002116
100783	1341557	3954844	432.904	432.95	0.046	0.002116
101242	1340549	3953669	432.904	432.95	0.046	0.002116
100125	1358203	3963157	435.434	435.48	0.046	0.002116
100651	1344844	3959541	437.064	437.11	0.046	0.002116
100652	1344822	3959500	437.074	437.12	0.046	0.002116
101432	1346848	3963141	438.314	438.36	0.046	0.002116
103928	1373753	3952259	445.955	446	0.046	0.002116
103720	1374045	3961448	446.354	446.4	0.046	0.002116
104489	1373808	3953549	447.334	447.38	0.046	0.002116
101126	1335327	3949491	449.584	449.63	0.046	0.002116

100384	1351775	3969099	450.994	451.04	0.046	0.002116
101576	1351405	3969074	451.124	451.17	0.046	0.002116
104372	1373706	3950312	453.574	453.62	0.046	0.002116
106186	1421089	3967525	470.344	470.39	0.046	0.002116
106825	1420912	3966636	472.374	472.42	0.046	0.002116
106105	1420788	3960122	474.834	474.88	0.046	0.002116
105696	1426579	3941123	487.945	487.99	0.046	0.002116
100587	1346142	3961856	436.843	436.89	0.047	0.002209
101774	1358375	3971215	439.523	439.57	0.047	0.002209
106193	1421235	3967804	469.143	469.19	0.047	0.002209
106122	1420877	3963990	472.063	472.11	0.047	0.002209
106927	1420741	3958455	473.243	473.29	0.047	0.002209
106694	1421848	3974417	481.163	481.21	0.047	0.002209
105724	1424984	3941666	486.113	486.16	0.047	0.002209
105456	1429148	3932117	495.063	495.11	0.047	0.002209
101030	1334000	3947676	558.133	558.18	0.047	0.002209
100878	1338461	3952052	432.572	432.62	0.048	0.002304
100899	1337662	3951696	432.892	432.94	0.048	0.002304
101151	1336178	3950372	432.932	432.98	0.048	0.002304
100839	1339947	3953001	433.172	433.22	0.048	0.002304
103807	1373792	3958302	444.782	444.83	0.048	0.002304
100386	1351691	3969103	451.162	451.21	0.048	0.002304
104376	1373710	3950393	453.522	453.57	0.048	0.002304
100503	1347844	3965048	454.022	454.07	0.048	0.002304
103613	1375038	3965926	455.122	455.17	0.048	0.002304
106137	1420894	3964665	470.392	470.44	0.048	0.002304
105851	1423070	3947675	477.183	477.23	0.048	0.002304
105600	1430127	3938054	490.902	490.95	0.048	0.002304
101262	1341807	3955108	432.911	432.96	0.049	0.002401
100768	1341981	3955329	433.181	433.23	0.049	0.002401
100862	1339135	3952359	434.411	434.46	0.049	0.002401
100132	1358210	3963360	435.211	435.26	0.049	0.002401
101618	1353230	3969028	445.141	445.19	0.049	0.002401
103948	1373736	3951588	446.151	446.2	0.049	0.002401
104097	1373164	3948542	451.881	451.93	0.049	0.002401
107634	1395872	3955602	457.541	457.59	0.049	0.002401
107534	1398458	3959729	457.721	457.77	0.049	0.002401
107657	1395504	3954347	458.061	458.11	0.049	0.002401
106852	1420887	3965013	470.321	470.37	0.049	0.002401
106883	1420850	3963411	473.701	473.75	0.049	0.002401
101187	1337349	3951495	432.81	432.86	0.05	0.0025
100864	1339054	3952317	434.09	434.14	0.05	0.0025
100143	1358212	3963714	434.81	434.86	0.05	0.0025
100030	1357123	3959726	435.84	435.89	0.05	0.0025
100626	1345343	3960439	437.13	437.18	0.05	0.0025
103830	1373867	3956707	445.87	445.92	0.05	0.0025
103659	1374920	3963980	446.02	446.07	0.05	0.0025
101606	1352718	3969049	446.36	446.41	0.05	0.0025
103670	1374823	3963570	447.43	447.48	0.05	0.0025
103707	1374105	3962094	449.51	449.56	0.05	0.0025
107521	1399112	3960020	458.58	458.63	0.05	0.0025
100436	1349262	3967590	459.89	459.94	0.05	0.0025

105990	1420329	3954127	474.51	474.56	0.05	0.0025
106945	1420725	3957637	474.91	474.96	0.05	0.0025
106995	1420672	3955302	475.279	475.33	0.05	0.0025
105966	1420862	3953444	475.35	475.4	0.05	0.0025
106000	1420590	3954769	475.65	475.7	0.05	0.0025
105928	1421556	3951601	476.8	476.85	0.05	0.0025
5547	1389342	4006191	747.67	747.72	0.05	0.0025
101180	1337059	3951271	433.099	433.15	0.051	0.002601
103568	1376288	3968249	446.339	446.39	0.051	0.002601
103449	1379602	3971548	450.249	450.3	0.051	0.002601
103437	1379602	3971860	452.229	452.28	0.051	0.002601
107539	1398233	3959624	457.649	457.7	0.051	0.002601
101543	1349555	3968028	458.519	458.57	0.051	0.002601
106836	1420901	3965811	471.779	471.83	0.051	0.002601
106090	1420773	3959365	474.729	474.78	0.051	0.002601
106035	1420721	3956529	474.979	475.03	0.051	0.002601
105848	1423148	3947572	476.919	476.97	0.051	0.002601
106695	1421847	3974330	480.719	480.77	0.051	0.002601
5537	1388968	4005811	742.589	742.64	0.051	0.002601
101240	1340496	3953608	432.798	432.85	0.052	0.002704
100779	1341669	3954972	433.078	433.13	0.052	0.002704
100662	1344604	3959111	436.808	436.86	0.052	0.002704
100167	1358237	3964685	437.238	437.29	0.052	0.002704
103669	1374834	3963602	447.338	447.39	0.052	0.002704
103686	1374535	3963004	448.618	448.67	0.052	0.002704
105705	1426107	3941324	486.758	486.81	0.052	0.002704
101864	1357043	3974746	493.468	493.52	0.052	0.002704
5530	1388703	4005543	739.068	739.12	0.052	0.002704
100709	1343641	3957392	433.437	433.49	0.053	0.002809
100035	1357242	3959786	436.157	436.21	0.053	0.002809
101355	1345036	3959855	436.847	436.9	0.053	0.002809
100645	1344977	3959779	436.867	436.92	0.053	0.002809
101383	1345631	3960908	436.917	436.97	0.053	0.002809
100576	1346375	3962275	436.917	436.97	0.053	0.002809
100242	1358343	3968568	436.957	437.01	0.053	0.002809
101749	1358369	3970184	439.787	439.84	0.053	0.002809
103584	1375872	3967714	443.197	443.25	0.053	0.002809
104187	1372439	3947279	451.117	451.17	0.053	0.002809
103615	1375027	3965833	453.238	453.29	0.053	0.002809
101547	1349645	3968171	457.657	457.71	0.053	0.002809
107549	1397730	3959393	457.967	458.02	0.053	0.002809
107585	1396570	3958012	459.467	459.52	0.053	0.002809
107458	1402143	3961290	460.857	460.91	0.053	0.002809
106154	1420908	3965406	470.387	470.44	0.053	0.002809
106839	1420897	3965659	471.157	471.21	0.053	0.002809
106082	1420765	3958955	473.158	473.21	0.053	0.002809
106970	1420701	3956522	475.057	475.11	0.053	0.002809
106689	1421853	3974671	482.337	482.39	0.053	0.002809
101854	1357401	3974367	485.867	485.92	0.053	0.002809
101182	1337129	3951330	432.896	432.95	0.054	0.002916
100034	1357222	3959776	436.076	436.13	0.054	0.002916
101317	1344139	3958253	436.156	436.21	0.054	0.002916

101339	1344612	3959096	436.746	436.8	0.054	0.002916
100419	1349650	3968253	459.426	459.48	0.054	0.002916
107671	1395293	3953633	460.296	460.35	0.054	0.002916
106042	1420729	3956876	474.816	474.87	0.054	0.002916
105971	1420727	3953620	475.286	475.34	0.054	0.002916
105721	1425171	3941606	485.706	485.76	0.054	0.002916
105454	1429153	3932048	494.926	494.98	0.054	0.002916
100734	1342992	3956487	433.055	433.11	0.055	0.003025
101145	1335956	3950140	433.665	433.72	0.055	0.003025
100582	1346253	3962054	436.875	436.93	0.055	0.003025
100673	1344376	3958704	436.905	436.96	0.055	0.003025
100527	1347322	3964114	441.965	442.02	0.055	0.003025
103648	1374947	3964395	444.915	444.97	0.055	0.003025
103908	1373783	3953132	447.125	447.18	0.055	0.003025
104488	1373806	3953462	447.485	447.54	0.055	0.003025
103534	1377451	3969074	447.695	447.75	0.055	0.003025
100509	1347715	3964817	453.345	453.4	0.055	0.003025
100480	1348326	3965914	456.535	456.59	0.055	0.003025
100437	1349241	3967551	459.885	459.94	0.055	0.003025
106898	1420766	3959917	474.905	474.96	0.055	0.003025
106966	1420704	3956687	474.995	475.05	0.055	0.003025
105489	1429249	3933345	493.975	494.03	0.055	0.003025
101066	1333630	3947447	544.375	544.43	0.055	0.003025
100906	1337404	3951549	433.324	433.38	0.056	0.003136
100085	1358152	3961468	435.104	435.16	0.056	0.003136
100208	1358299	3967090	435.784	435.84	0.056	0.003136
101377	1345513	3960697	436.834	436.89	0.056	0.003136
100640	1345078	3959963	436.884	436.94	0.056	0.003136
101341	1344655	3959173	437.024	437.08	0.056	0.003136
103727	1374038	3961157	446.114	446.17	0.056	0.003136
104142	1372757	3947828	451.144	451.2	0.056	0.003136
104007	1373685	3950163	453.524	453.58	0.056	0.003136
106210	1421637	3968534	465.174	465.23	0.056	0.003136
106004	1420633	3954941	475.864	475.92	0.056	0.003136
106307	1421845	3973331	476.194	476.25	0.056	0.003136
105477	1429140	3933025	495.194	495.25	0.056	0.003136
105444	1429146	3931570	496.264	496.32	0.056	0.003136
101051	1333171	3947175	549.774	549.83	0.056	0.003136
101364	1345231	3960203	436.963	437.02	0.057	0.003249
101365	1345252	3960240	436.973	437.03	0.057	0.003249
103757	1373839	3960104	444.183	444.24	0.057	0.003249
103532	1377501	3969107	447.753	447.81	0.057	0.003249
103444	1379602	3971703	450.753	450.81	0.057	0.003249
107650	1395622	3954750	458.183	458.24	0.057	0.003249
107592	1396470	3957673	458.613	458.67	0.057	0.003249
107558	1397326	3959161	458.893	458.95	0.057	0.003249
105893	1422117	3949818	474.153	474.21	0.057	0.003249
106956	1420716	3957125	474.923	474.98	0.057	0.003249
101059	1333405	3947316	540.623	540.68	0.057	0.003249
101080	1334158	3947753	563.713	563.77	0.057	0.003249
100883	1338262	3951965	432.592	432.65	0.058	0.003364
100765	1342067	3955427	433.302	433.36	0.058	0.003364

101138	1335733	3949909	435.032	435.09	0.058	0.003364
101320	1344218	3958395	436.602	436.66	0.058	0.003364
100250	1358342	3968768	437.332	437.39	0.058	0.003364
104458	1373765	3952243	446.052	446.11	0.058	0.003364
103944	1373738	3951710	446.432	446.49	0.058	0.003364
104502	1373824	3954162	447.212	447.27	0.058	0.003364
103527	1377615	3969181	447.793	447.85	0.058	0.003364
107499	1400235	3960535	459.802	459.86	0.058	0.003364
106195	1421272	3967866	468.362	468.42	0.058	0.003364
106053	1420739	3957427	474.762	474.82	0.058	0.003364
106054	1420740	3957468	474.832	474.89	0.058	0.003364
106980	1420694	3956040	475.462	475.52	0.058	0.003364
105801	1424078	3945508	476.612	476.67	0.058	0.003364
100876	1338542	3952089	432.801	432.86	0.059	0.003481
101725	1358159	3968891	436.961	437.02	0.059	0.003481
101374	1345456	3960601	436.991	437.05	0.059	0.003481
103942	1373740	3951773	446.391	446.45	0.059	0.003481
101534	1349391	3967740	459.411	459.47	0.059	0.003481
105604	1429996	3938222	491.101	491.16	0.059	0.003481
105442	1429145	3931496	496.291	496.35	0.059	0.003481
105443	1429145	3931533	496.361	496.42	0.059	0.003481
100736	1342934	3956421	433.01	433.07	0.06	0.0036
100080	1358147	3961251	435.7	435.76	0.06	0.0036
100221	1358314	3967667	436.08	436.14	0.06	0.0036
103640	1374964	3964719	445.59	445.65	0.06	0.0036
100520	1347477	3964392	448.15	448.21	0.06	0.0036
101586	1351826	3969069	450.78	450.84	0.06	0.0036
104370	1373704	3950271	453.6	453.66	0.06	0.0036
101123	1335235	3949396	458.61	458.67	0.06	0.0036
107000	1420644	3955073	475.35	475.41	0.06	0.0036
105850	1423096	3947641	476.99	477.05	0.06	0.0036
100782	1341585	3954876	432.889	432.95	0.061	0.003721
100694	1343955	3957950	435.149	435.21	0.061	0.003721
103563	1376444	3968398	446.189	446.25	0.061	0.003721
104094	1373193	3948594	451.919	451.98	0.061	0.003721
106158	1420912	3965586	470.609	470.67	0.061	0.003721
106181	1421025	3967361	470.629	470.69	0.061	0.003721
105997	1420563	3954676	475.659	475.72	0.061	0.003721
106692	1421850	3974502	481.909	481.97	0.061	0.003721
105674	1427562	3940379	487.789	487.85	0.061	0.003721
100828	1340274	3953375	432.918	432.98	0.062	0.003844
101323	1344278	3958501	436.728	436.79	0.062	0.003844
101378	1345531	3960728	436.878	436.94	0.062	0.003844
103724	1374040	3961282	446.218	446.28	0.062	0.003844
100475	1348429	3966098	457.029	457.09	0.062	0.003844
107489	1400678	3960730	460.468	460.53	0.062	0.003844
107679	1395117	3953232	462.268	462.33	0.062	0.003844
106936	1420733	3958045	474.398	474.46	0.062	0.003844
105812	1423981	3946076	475.648	475.71	0.062	0.003844
105687	1427049	3940808	488.298	488.36	0.062	0.003844
100888	1338073	3951882	432.507	432.57	0.063	0.003969
101245	1340711	3953856	433.097	433.16	0.063	0.003969

100528	1347299	3964074	441.307	441.37	0.063	0.003969
103556	1376686	3968580	445.868	445.93	0.063	0.003969
103554	1376759	3968627	446.017	446.08	0.063	0.003969
103514	1377985	3969423	449.117	449.18	0.063	0.003969
103508	1378168	3969547	450.487	450.55	0.063	0.003969
104011	1373683	3950088	453.577	453.64	0.063	0.003969
107449	1402635	3961428	461.277	461.34	0.063	0.003969
107687	1394861	3952933	463.238	463.3	0.063	0.003969
106120	1420875	3963880	472.397	472.46	0.063	0.003969
105899	1422030	3950099	474.197	474.26	0.063	0.003969
106322	1421861	3974111	479.667	479.73	0.063	0.003969
100884	1338223	3951948	432.526	432.59	0.064	0.004096
100689	1344054	3958128	435.866	435.93	0.064	0.004096
100112	1358184	3962635	436.376	436.44	0.064	0.004096
100636	1345155	3960104	436.736	436.8	0.064	0.004096
101338	1344590	3959058	436.746	436.81	0.064	0.004096
100647	1344934	3959702	436.926	436.99	0.064	0.004096
104426	1373744	3951446	446.626	446.69	0.064	0.004096
100377	1352072	3969085	450.636	450.7	0.064	0.004096
104385	1373720	3950566	454.156	454.22	0.064	0.004096
106128	1420883	3964239	471.166	471.23	0.064	0.004096
105873	1422447	3948774	477.936	478	0.064	0.004096
105634	1429018	3939114	490.166	490.23	0.064	0.004096
101284	1342865	3956319	433.005	433.07	0.065	0.004225
100684	1344152	3958304	436.305	436.37	0.065	0.004225
101403	1346045	3961648	436.775	436.84	0.065	0.004225
101416	1346363	3962218	436.925	436.99	0.065	0.004225
100558	1346707	3962925	437.125	437.19	0.065	0.004225
101764	1358387	3970811	438.815	438.88	0.065	0.004225
101128	1335387	3949554	444.845	444.91	0.065	0.004225
103728	1374037	3961115	446.065	446.13	0.065	0.004225
104443	1373753	3951824	446.265	446.33	0.065	0.004225
101605	1352673	3969050	446.705	446.77	0.065	0.004225
103620	1375011	3965590	447.705	447.77	0.065	0.004225
103691	1374420	3962801	448.515	448.58	0.065	0.004225
104353	1373692	3949912	453.945	454.01	0.065	0.004225
107620	1396083	3956318	456.855	456.92	0.065	0.004225
107571	1396859	3958682	460.035	460.1	0.065	0.004225
106225	1421777	3969766	472.735	472.8	0.065	0.004225
106015	1420700	3955511	475.585	475.65	0.065	0.004225
105686	1427085	3940779	488.075	488.14	0.065	0.004225
105484	1429206	3933220	494.355	494.42	0.065	0.004225
100800	1341083	3954302	432.804	432.87	0.066	0.004356
100832	1340158	3953241	432.864	432.93	0.066	0.004356
100623	1345396	3960535	436.844	436.91	0.066	0.004356
101763	1358386	3970770	438.984	439.05	0.066	0.004356
101797	1358379	3972170	442.934	443	0.066	0.004356
101614	1353075	3969034	444.704	444.77	0.066	0.004356
104442	1373752	3951796	446.374	446.44	0.066	0.004356
103710	1374075	3961947	449.274	449.34	0.066	0.004356
104224	1372725	3947749	451.144	451.21	0.066	0.004356
104356	1373694	3949978	453.814	453.88	0.066	0.004356

107628	1395968	3955925	456.614	456.68	0.066	0.004356
101497	1348500	3966148	457.264	457.33	0.066	0.004356
101835	1357987	3973869	465.624	465.69	0.066	0.004356
106857	1420882	3964767	470.354	470.42	0.066	0.004356
106886	1420847	3963294	473.904	473.97	0.066	0.004356
106018	1420705	3955673	475.274	475.34	0.066	0.004356
101186	1337274	3951442	432.743	432.81	0.067	0.004489
104514	1373838	3954755	446.243	446.31	0.067	0.004489
103882	1373813	3954239	447.173	447.24	0.067	0.004489
104377	1373711	3950414	453.513	453.58	0.067	0.004489
103999	1373689	3950321	453.523	453.59	0.067	0.004489
103985	1373710	3950610	453.553	453.62	0.067	0.004489
107580	1396658	3958292	460.583	460.65	0.067	0.004489
106938	1420732	3957970	474.633	474.7	0.067	0.004489
105845	1423251	3947435	476.583	476.65	0.067	0.004489
106702	1421841	3973983	479.003	479.07	0.067	0.004489
106693	1421849	3974460	481.693	481.76	0.067	0.004489
101852	1357466	3974314	484.443	484.51	0.067	0.004489
101414	1346321	3962141	436.862	436.93	0.068	0.004624
107551	1397654	3959357	458.422	458.49	0.068	0.004624
107472	1401479	3961037	460.282	460.35	0.068	0.004624
106188	1421117	3967589	470.372	470.44	0.068	0.004624
106877	1420857	3963729	473.062	473.13	0.068	0.004624
106705	1421839	3973813	478.492	478.56	0.068	0.004624
101855	1357367	3974393	486.512	486.58	0.068	0.004624
101113	1334924	3949067	497.502	497.57	0.068	0.004624
101276	1342187	3955545	432.961	433.03	0.069	0.004761
100179	1358268	3965845	436.841	436.91	0.069	0.004761
101771	1358387	3971037	438.741	438.81	0.069	0.004761
103709	1374084	3961995	449.371	449.44	0.069	0.004761
107536	1398382	3959694	457.721	457.79	0.069	0.004761
106012	1420695	3955393	475.741	475.81	0.069	0.004761
105805	1424052	3945723	476.151	476.22	0.069	0.004761
100803	1340997	3954203	432.7	432.77	0.07	0.0049
101219	1338972	3952264	434.3	434.37	0.07	0.0049
101351	1344875	3959568	437.09	437.16	0.07	0.0049
104111	1373035	3948312	451.88	451.95	0.07	0.0049
104025	1373677	3949824	453.68	453.75	0.07	0.0049
107607	1396261	3956919	456.29	456.36	0.07	0.0049
106233	1421786	3970118	471.95	472.02	0.07	0.0049
106165	1420917	3965904	472.23	472.3	0.07	0.0049
106021	1420708	3955838	475.18	475.25	0.07	0.0049
105980	1420400	3953916	475.41	475.48	0.07	0.0049
106979	1420695	3956080	475.42	475.49	0.07	0.0049
106302	1421840	3973030	475.6	475.67	0.07	0.0049
105713	1425647	3941465	485.58	485.65	0.07	0.0049
105673	1427589	3940355	487.88	487.95	0.07	0.0049
105464	1429094	3932463	495.69	495.76	0.07	0.0049
101067	1333665	3947468	545.57	545.64	0.07	0.0049
100829	1340245	3953342	432.839	432.91	0.071	0.005041
100874	1338626	3952125	432.929	433	0.071	0.005041
100606	1345733	3961125	436.859	436.93	0.071	0.005041

103968	1373727	3951024	446.279	446.35	0.071	0.005041
103399	1379603	3972914	460.469	460.54	0.071	0.005041
107579	1396673	3958330	460.549	460.62	0.071	0.005041
106761	1421784	3971000	470.309	470.38	0.071	0.005041
106889	1420844	3963180	473.779	473.85	0.071	0.005041
105840	1423392	3947247	476.359	476.43	0.071	0.005041
105697	1426539	3941145	487.859	487.93	0.071	0.005041
101043	1333446	3947353	540.409	540.48	0.071	0.005041
100798	1341140	3954367	432.988	433.06	0.072	0.005184
100632	1345231	3960242	436.678	436.75	0.072	0.005184
100570	1346475	3962460	436.738	436.81	0.072	0.005184
101439	1347005	3963464	439.378	439.45	0.072	0.005184
103876	1373820	3954557	446.618	446.69	0.072	0.005184
106207	1421571	3968382	464.008	464.08	0.072	0.005184
106148	1420903	3965121	470.688	470.76	0.072	0.005184
105961	1420998	3953223	475.428	475.5	0.072	0.005184
106010	1420686	3955277	475.688	475.76	0.072	0.005184
105910	1421852	3950684	476.918	476.99	0.072	0.005184
105452	1429155	3931942	495.348	495.42	0.072	0.005184
105404	1429174	3930359	496.968	497.04	0.072	0.005184
101230	1340222	3953296	432.757	432.83	0.073	0.005329
100778	1341697	3955004	433.117	433.19	0.073	0.005329
100691	1344015	3958057	435.577	435.65	0.073	0.005329
101747	1358367	3970103	438.797	438.87	0.073	0.005329
103642	1374960	3964636	445.297	445.37	0.073	0.005329
104429	1373745	3951507	446.437	446.51	0.073	0.005329
107674	1395235	3953465	461.387	461.46	0.073	0.005329
106947	1420723	3957530	474.857	474.93	0.073	0.005329
106948	1420723	3957494	474.907	474.98	0.073	0.005329
105682	1427259	3940633	487.687	487.76	0.073	0.005329
101199	1337806	3951745	432.416	432.49	0.074	0.005476
101310	1344001	3958008	435.286	435.36	0.074	0.005476
103509	1378134	3969524	450.166	450.24	0.074	0.005476
104387	1373722	3950604	453.916	453.99	0.074	0.005476
101849	1357562	3974236	482.026	482.1	0.074	0.005476
105666	1427847	3940130	488.296	488.37	0.074	0.005476
100789	1341390	3954653	432.745	432.82	0.075	0.005625
100660	1344646	3959187	437.205	437.28	0.075	0.005625
101498	1348541	3966220	457.365	457.44	0.075	0.005625
107675	1395218	3953425	461.615	461.69	0.075	0.005625
106806	1421024	3967413	471.046	471.12	0.075	0.005625
105898	1422042	3950058	474.055	474.13	0.075	0.005625
101033	1333890	3947612	554.155	554.23	0.075	0.005625
101218	1338566	3952084	432.814	432.89	0.076	0.005776
100726	1343217	3956761	433.134	433.21	0.076	0.005776
101309	1343981	3957972	435.184	435.26	0.076	0.005776
100204	1358295	3966909	436.494	436.57	0.076	0.005776
100637	1345136	3960069	436.854	436.93	0.076	0.005776
100066	1358124	3960637	436.894	436.97	0.076	0.005776
100574	1346412	3962342	437.024	437.1	0.076	0.005776
100443	1349111	3967317	460.414	460.49	0.076	0.005776
106006	1420661	3955088	475.904	475.98	0.076	0.005776

106316	1421856	3973805	478.374	478.45	0.076	0.005776
101194	1337618	3951653	432.394	432.47	0.077	0.005929
100912	1337193	3951399	433.403	433.48	0.077	0.005929
100630	1345270	3960310	436.783	436.86	0.077	0.005929
100671	1344418	3958777	436.863	436.94	0.077	0.005929
100579	1346316	3962167	436.893	436.97	0.077	0.005929
100674	1344355	3958668	436.903	436.98	0.077	0.005929
103513	1378012	3969442	449.353	449.43	0.077	0.005929
104264	1373055	3948328	451.963	452.04	0.077	0.005929
103614	1375032	3965880	454.243	454.32	0.077	0.005929
101829	1358149	3973664	458.083	458.16	0.077	0.005929
107659	1395479	3954262	458.133	458.21	0.077	0.005929
107588	1396532	3957886	459.213	459.29	0.077	0.005929
100439	1349197	3967473	460.123	460.2	0.077	0.005929
107491	1400606	3960699	460.393	460.47	0.077	0.005929
106101	1420784	3959917	475.024	475.1	0.077	0.005929
100586	1346165	3961895	436.782	436.86	0.078	0.006084
101434	1346900	3963246	438.732	438.81	0.078	0.006084
104507	1373829	3954401	446.972	447.05	0.078	0.006084
107530	1398656	3959816	458.102	458.18	0.078	0.006084
107515	1399440	3960169	458.712	458.79	0.078	0.006084
105892	1422129	3949777	474.272	474.35	0.078	0.006084
106108	1420792	3960289	474.922	475	0.078	0.006084
106303	1421841	3973115	475.882	475.96	0.078	0.006084
105462	1429105	3932394	495.542	495.62	0.078	0.006084
100732	1343049	3956554	433.171	433.25	0.079	0.006241
103765	1373644	3960058	443.861	443.94	0.079	0.006241
103704	1374146	3962239	450.001	450.08	0.079	0.006241
104240	1372853	3947970	451.621	451.7	0.079	0.006241
104002	1373688	3950262	453.491	453.57	0.079	0.006241
104359	1373697	3950041	453.641	453.72	0.079	0.006241
106162	1420915	3965763	471.501	471.58	0.079	0.006241
106169	1420920	3966088	472.371	472.45	0.079	0.006241
105664	1427907	3940077	488.331	488.41	0.079	0.006241
101062	1333500	3947371	540.861	540.94	0.079	0.006241
101027	1334112	3947740	562.041	562.12	0.079	0.006241
100781	1341613	3954908	432.93	433.01	0.08	0.0064
100866	1338970	3952277	433.92	434	0.08	0.0064
101436	1346936	3963318	438.65	438.73	0.08	0.0064
103929	1373752	3952224	446	446.08	0.08	0.0064
104236	1372821	3947914	451.63	451.71	0.08	0.0064
101502	1348642	3966400	457.85	457.93	0.08	0.0064
100435	1349283	3967629	459.8	459.88	0.08	0.0064
107465	1401774	3961152	460.33	460.41	0.08	0.0064
106957	1420715	3957087	474.93	475.01	0.08	0.0064
105930	1421533	3951684	476.55	476.63	0.08	0.0064
105722	1425123	3941621	485.74	485.82	0.08	0.0064
101246	1340767	3953920	433.029	433.11	0.081	0.006561
100905	1337440	3951571	433.239	433.32	0.081	0.006561
103825	1373872	3956924	445.199	445.28	0.081	0.006561
103601	1375437	3967114	448.899	448.98	0.081	0.006561
104241	1372860	3947984	451.639	451.72	0.081	0.006561

104348	1373688	3949807	453.699	453.78	0.081	0.006561
101531	1349340	3967649	459.489	459.57	0.081	0.006561
107448	1402725	3961443	461.079	461.16	0.081	0.006561
106065	1420750	3958057	474.339	474.42	0.081	0.006561
106971	1420700	3956455	475.089	475.17	0.081	0.006561
105923	1421647	3951314	477.369	477.45	0.081	0.006561
101308	1343961	3957936	435.118	435.2	0.082	0.006724
104283	1373212	3948609	451.828	451.91	0.082	0.006724
104012	1373683	3950071	453.588	453.67	0.082	0.006724
107683	1394997	3953063	463.608	463.69	0.082	0.006724
106251	1421808	3970617	471.668	471.75	0.082	0.006724
106880	1420853	3963568	473.518	473.6	0.082	0.006724
106088	1420771	3959285	474.479	474.56	0.082	0.006724
106026	1420713	3956084	475.338	475.42	0.082	0.006724
105833	1423585	3946972	476.928	477.01	0.082	0.006724
105596	1430240	3937874	490.888	490.97	0.082	0.006724
5525	1388508	4005352	736.478	736.56	0.082	0.006724
100754	1342400	3955808	433.057	433.14	0.083	0.006889
100049	1357611	3959971	436.287	436.37	0.083	0.006889
106930	1420739	3958341	473.737	473.82	0.083	0.006889
105926	1421595	3951478	476.987	477.07	0.083	0.006889
105854	1422967	3947814	477.437	477.52	0.083	0.006889
106332	1421874	3974679	482.487	482.57	0.083	0.006889
105562	1430329	3936018	494.447	494.53	0.083	0.006889
101331	1344442	3958794	436.836	436.92	0.084	0.007056
103550	1376906	3968722	447.136	447.22	0.084	0.007056
106275	1421812	3971653	471.086	471.17	0.084	0.007056
105465	1429085	3932530	495.886	495.97	0.084	0.007056
101050	1333163	3947167	550.386	550.47	0.084	0.007056
101193	1337580	3951632	432.525	432.61	0.085	0.007225
100569	1346489	3962486	436.935	437.02	0.085	0.007225
103779	1373627	3959461	443.715	443.8	0.085	0.007225
103598	1375512	3967231	446.525	446.61	0.085	0.007225
103539	1377298	3968976	447.435	447.52	0.085	0.007225
104140	1372774	3947858	451.305	451.39	0.085	0.007225
107609	1396229	3956812	456.435	456.52	0.085	0.007225
103405	1379603	3972677	458.325	458.41	0.085	0.007225
107475	1401327	3960981	460.205	460.29	0.085	0.007225
107494	1400461	3960635	460.265	460.35	0.085	0.007225
106118	1420874	3963805	472.675	472.76	0.085	0.007225
100082	1358149	3961338	435.434	435.52	0.086	0.007396
100111	1358182	3962592	436.454	436.54	0.086	0.007396
101358	1345103	3959973	436.804	436.89	0.086	0.007396
104246	1372906	3948066	451.784	451.87	0.086	0.007396
106720	1421823	3973032	475.554	475.64	0.086	0.007396
106719	1421824	3973072	475.614	475.7	0.086	0.007396
105669	1427729	3940233	488.104	488.19	0.086	0.007396
101337	1344569	3959020	436.704	436.79	0.087	0.007569
100621	1345433	3960599	436.863	436.95	0.087	0.007569
101367	1345294	3960315	436.983	437.07	0.087	0.007569
103897	1373795	3953564	447.303	447.39	0.087	0.007569
107599	1396371	3957300	457.533	457.62	0.087	0.007569

107461	1401974	3961227	460.423	460.51	0.087	0.007569
107676	1395200	3953385	461.753	461.84	0.087	0.007569
106151	1420906	3965263	470.403	470.49	0.087	0.007569
106155	1420908	3965441	470.443	470.53	0.087	0.007569
106837	1420900	3965773	471.503	471.59	0.087	0.007569
105920	1421686	3951193	477.463	477.55	0.087	0.007569
101134	1335606	3949779	436.452	436.54	0.088	0.007744
100562	1346666	3962846	437.022	437.11	0.088	0.007744
101477	1347978	3965212	454.412	454.5	0.088	0.007744
103400	1379603	3972874	460.182	460.27	0.088	0.007744
106815	1420936	3967047	471.992	472.08	0.088	0.007744
106227	1421781	3969943	472.352	472.44	0.088	0.007744
106037	1420724	3956647	474.982	475.07	0.088	0.007744
101088	1334443	3947952	573.862	573.95	0.088	0.007744
100117	1358192	3962847	435.991	436.08	0.089	0.007921
100634	1345193	3960173	436.731	436.82	0.089	0.007921
101421	1346507	3962488	437.171	437.26	0.089	0.007921
103489	1378855	3970007	451.892	451.98	0.089	0.007921
101470	1347812	3964915	453.581	453.67	0.089	0.007921
104364	1373699	3950146	453.661	453.75	0.089	0.007921
100504	1347823	3965009	453.921	454.01	0.089	0.007921
107663	1395417	3954057	458.701	458.79	0.089	0.007921
106846	1420892	3965298	470.471	470.56	0.089	0.007921
106711	1421832	3973482	477.291	477.38	0.089	0.007921
105915	1421777	3950917	477.442	477.53	0.089	0.007921
105663	1427968	3940024	488.381	488.47	0.089	0.007921
105415	1428961	3930832	496.891	496.98	0.089	0.007921
100891	1337962	3951833	432.57	432.66	0.09	0.0081
101256	1341603	3954875	432.83	432.92	0.09	0.0081
100578	1346336	3962204	436.81	436.9	0.09	0.0081
100545	1346949	3963390	439.23	439.32	0.09	0.0081
103622	1375006	3965493	446.81	446.9	0.09	0.0081
103877	1373818	3954465	446.83	446.92	0.09	0.0081
104341	1373685	3949666	453.36	453.45	0.09	0.0081
101475	1347935	3965136	454.12	454.21	0.09	0.0081
106174	1420930	3966692	472.18	472.27	0.09	0.0081
106333	1421874	3974722	482.82	482.91	0.09	0.0081
105594	1430301	3937757	491.21	491.3	0.09	0.0081
101072	1333852	3947576	552.46	552.55	0.09	0.0081
101075	1333964	3947641	556.67	556.76	0.09	0.0081
100910	1337264	3951452	433.279	433.37	0.091	0.008281
104483	1373798	3953192	447.019	447.11	0.091	0.008281
104481	1373795	3953097	447.319	447.41	0.091	0.008281
103992	1373697	3950466	453.649	453.74	0.091	0.008281
104366	1373700	3950188	453.649	453.74	0.091	0.008281
107655	1395542	3954479	458.259	458.35	0.091	0.008281
105955	1421142	3952910	475.51	475.6	0.091	0.008281
105740	1424227	3942175	486.199	486.29	0.091	0.008281
100881	1338340	3952000	432.478	432.57	0.092	0.008464
100084	1358151	3961425	435.188	435.28	0.092	0.008464
100580	1346296	3962130	436.838	436.93	0.092	0.008464
101363	1345210	3960165	437.008	437.1	0.092	0.008464

103898	1373794	3953523	447.338	447.43	0.092	0.008464
103521	1377836	3969324	448.058	448.15	0.092	0.008464
104014	1373682	3950035	453.628	453.72	0.092	0.008464
100493	1348055	3965426	455.128	455.22	0.092	0.008464
107471	1401516	3961051	460.348	460.44	0.092	0.008464
106943	1420727	3957709	474.868	474.96	0.092	0.008464
105458	1429133	3932222	494.908	495	0.092	0.008464
100750	1342521	3955946	433.157	433.25	0.093	0.008649
103959	1373733	3951277	446.827	446.92	0.093	0.008649
100518	1347520	3964470	449.977	450.07	0.093	0.008649
107673	1395250	3953506	461.167	461.26	0.093	0.008649
105949	1421236	3952627	474.387	474.48	0.093	0.008649
106100	1420784	3959877	474.937	475.03	0.093	0.008649
106972	1420700	3956420	475.127	475.22	0.093	0.008649
105819	1423887	3946368	475.977	476.07	0.093	0.008649
105874	1422432	3948819	477.667	477.76	0.093	0.008649
105677	1427449	3940475	487.647	487.74	0.093	0.008649
101079	1334118	3947730	562.157	562.25	0.093	0.008649
101273	1342106	3955451	433.126	433.22	0.094	0.008836
100160	1358229	3964363	437.386	437.48	0.094	0.008836
103964	1373729	3951138	446.236	446.33	0.094	0.008836
104034	1373671	3949663	453.276	453.37	0.094	0.008836
106096	1420780	3959679	475.106	475.2	0.094	0.008836
105999	1420581	3954737	475.556	475.65	0.094	0.008836
101071	1333815	3947554	550.906	551	0.094	0.008836
101200	1337844	3951762	432.345	432.44	0.095	0.009025
101181	1337094	3951301	432.985	433.08	0.095	0.009025
100577	1346356	3962239	436.775	436.87	0.095	0.009025
101381	1345582	3960819	436.875	436.97	0.095	0.009025
103738	1374025	3960707	445.675	445.77	0.095	0.009025
104456	1373763	3952191	446.125	446.22	0.095	0.009025
103996	1373692	3950381	453.575	453.67	0.095	0.009025
100478	1348367	3965987	456.665	456.76	0.095	0.009025
101550	1349721	3968272	457.145	457.24	0.095	0.009025
106183	1421049	3967429	470.375	470.47	0.095	0.009025
101185	1337237	3951415	432.844	432.94	0.096	0.009216
103565	1376381	3968341	446.284	446.38	0.096	0.009216
103668	1374844	3963633	447.244	447.34	0.096	0.009216
103693	1374373	3962719	449.254	449.35	0.096	0.009216
104275	1373144	3948485	451.854	451.95	0.096	0.009216
101834	1358016	3973838	464.234	464.33	0.096	0.009216
106214	1421699	3968725	467.983	468.08	0.096	0.009216
101073	1333889	3947597	553.885	553.98	0.096	0.009216
101265	1341891	3955205	432.913	433.01	0.097	0.009409
100760	1342215	3955598	433.133	433.23	0.097	0.009409
101371	1345396	3960496	437.173	437.27	0.097	0.009409
104501	1373822	3954083	447.073	447.17	0.097	0.009409
103900	1373792	3953444	447.353	447.45	0.097	0.009409
101601	1352493	3969057	448.103	448.2	0.097	0.009409
104092	1373213	3948630	451.923	452.02	0.097	0.009409
107639	1395798	3955350	457.973	458.07	0.097	0.009409
107519	1399237	3960076	458.723	458.82	0.097	0.009409

107590	1396494	3957757	458.783	458.88	0.097	0.009409
106200	1421388	3968063	465.023	465.12	0.097	0.009409
107009	1420563	3954742	474.793	474.89	0.097	0.009409
105672	1427617	3940331	487.923	488.02	0.097	0.009409
101857	1357300	3974448	488.313	488.41	0.097	0.009409
101063	1333531	3947389	541.523	541.62	0.097	0.009409
100565	1346639	3962787	436.822	436.92	0.098	0.009604
101361	1345167	3960089	436.902	437	0.098	0.009604
104147	1372713	3947751	451.022	451.12	0.098	0.009604
106789	1421332	3968000	466.302	466.4	0.098	0.009604
105906	1421913	3950482	476.182	476.28	0.098	0.009604
100984	1335027	3949199	483.322	483.42	0.098	0.009604
105603	1430051	3938157	491.032	491.13	0.098	0.009604
100770	1341924	3955264	432.931	433.03	0.099	0.009801
100908	1337334	3951502	433.311	433.41	0.099	0.009801
100547	1346909	3963307	438.741	438.84	0.099	0.009801
104510	1373834	3954558	446.581	446.68	0.099	0.009801
103973	1373723	3950879	446.701	446.8	0.099	0.009801
103672	1374800	3963506	447.781	447.88	0.099	0.009801
100388	1351608	3969109	451.461	451.56	0.099	0.009801
104016	1373681	3949997	453.701	453.8	0.099	0.009801
107604	1396304	3957067	456.611	456.71	0.099	0.009801
105958	1421076	3953068	475.452	475.55	0.099	0.009801
101212	1338317	3951975	432.49	432.59	0.1	0.01
100844	1339788	3952835	433.45	433.55	0.1	0.01
103740	1374024	3960632	445.58	445.68	0.1	0.01
103661	1374910	3963909	446.24	446.34	0.1	0.01
100506	1347780	3964933	453.88	453.98	0.1	0.01
107500	1400196	3960517	459.65	459.75	0.1	0.01
107486	1400820	3960785	460.54	460.64	0.1	0.01
106110	1420794	3960372	474.9	475	0.1	0.01
101086	1334381	3947895	572.61	572.71	0.1	0.01
100679	1344253	3958485	436.749	436.85	0.101	0.010201
104406	1373736	3951027	446.169	446.27	0.101	0.010201
104227	1372749	3947790	451.229	451.33	0.101	0.010201
104123	1372925	3948117	451.799	451.9	0.101	0.010201
100471	1348513	3966247	457.469	457.57	0.101	0.010201
107638	1395811	3955392	457.919	458.02	0.101	0.010201
106891	1420772	3960170	474.589	474.69	0.101	0.010201
106910	1420759	3959336	474.699	474.8	0.101	0.010201
101286	1343069	3956555	433.458	433.56	0.102	0.010404
101306	1343921	3957865	434.948	435.05	0.102	0.010404
100203	1358294	3966865	436.838	436.94	0.102	0.010404
104231	1372780	3947844	451.398	451.5	0.102	0.010404
104074	1373393	3948947	452.568	452.67	0.102	0.010404
107629	1395944	3955846	456.598	456.7	0.102	0.010404
107497	1400312	3960570	459.968	460.07	0.102	0.010404
107479	1401141	3960910	460.218	460.32	0.102	0.010404
106922	1420746	3958720	472.718	472.82	0.102	0.010404
106987	1420688	3955691	475.488	475.59	0.102	0.010404
100836	1340038	3953104	432.977	433.08	0.103	0.010609
100708	1343662	3957429	433.577	433.68	0.103	0.010609

101298	1343687	3957446	433.907	434.01	0.103	0.010609
100687	1344094	3958198	436.147	436.25	0.103	0.010609
100159	1358227	3964322	436.967	437.07	0.103	0.010609
101770	1358387	3971014	438.597	438.7	0.103	0.010609
104129	1372875	3948030	451.627	451.73	0.103	0.010609
104278	1373168	3948530	451.837	451.94	0.103	0.010609
104027	1373676	3949787	453.507	453.61	0.103	0.010609
104010	1373684	3950106	453.537	453.64	0.103	0.010609
103555	1376723	3968603	445.896	446	0.104	0.010816
104238	1372837	3947942	451.626	451.73	0.104	0.010816
104116	1372987	3948227	451.756	451.86	0.104	0.010816
100498	1347950	3965238	454.456	454.56	0.104	0.010816
106072	1420755	3958439	473.296	473.4	0.104	0.010816
105865	1422632	3948353	478.736	478.84	0.104	0.010816
101208	1338154	3951903	432.495	432.6	0.105	0.011025
100907	1337369	3951526	433.275	433.38	0.105	0.011025
104096	1373173	3948559	451.915	452.02	0.105	0.011025
106079	1420762	3958786	472.825	472.93	0.105	0.011025
106022	1420709	3955879	475.175	475.28	0.105	0.011025
105998	1420572	3954706	475.625	475.73	0.105	0.011025
105841	1423370	3947277	476.295	476.4	0.105	0.011025
105918	1421725	3951073	477.445	477.55	0.105	0.011025
101210	1338235	3951939	432.534	432.64	0.106	0.011236
101299	1343775	3957603	434.174	434.28	0.106	0.011236
100164	1358233	3964543	436.864	436.97	0.106	0.011236
103634	1374978	3964966	446.084	446.19	0.106	0.011236
104430	1373746	3951527	446.334	446.44	0.106	0.011236
103895	1373797	3953647	447.284	447.39	0.106	0.011236
104148	1372704	3947736	450.964	451.07	0.106	0.011236
106841	1420896	3965583	470.634	470.74	0.106	0.011236
106826	1420912	3966597	472.474	472.58	0.106	0.011236
106879	1420853	3963608	473.514	473.62	0.106	0.011236
106019	1420705	3955714	475.304	475.41	0.106	0.011236
105584	1430468	3937241	492.144	492.25	0.106	0.011236
105414	1428973	3930805	496.854	496.96	0.106	0.011236
101275	1342160	3955513	432.913	433.02	0.107	0.011449
100867	1338927	3952258	433.573	433.68	0.107	0.011449
100047	1357534	3959933	436.663	436.77	0.107	0.011449
101418	1346425	3962333	437.213	437.32	0.107	0.011449
101767	1358388	3970925	438.403	438.51	0.107	0.011449
104508	1373830	3954440	446.854	446.96	0.107	0.011449
101462	1347606	3964546	451.343	451.45	0.107	0.011449
104091	1373224	3948649	451.953	452.06	0.107	0.011449
104018	1373680	3949956	453.753	453.86	0.107	0.011449
103401	1379603	3972833	459.813	459.92	0.107	0.011449
105995	1420395	3954266	474.593	474.7	0.107	0.011449
105598	1430196	3937947	490.883	490.99	0.107	0.011449
101099	1334708	3948494	549.703	549.81	0.107	0.011449
100901	1337587	3951657	432.752	432.86	0.108	0.011664
100729	1343133	3956656	432.952	433.06	0.108	0.011664
101799	1358382	3972256	443.212	443.32	0.108	0.011664
106780	1421518	3968314	463.162	463.27	0.108	0.011664

101069	1333739	3947510	548.142	548.25	0.108	0.011664
101047	1333139	3947132	551.102	551.21	0.108	0.011664
101195	1337656	3951673	432.331	432.44	0.109	0.011881
104144	1372739	3947797	451.081	451.19	0.109	0.011881
107678	1395139	3953269	461.981	462.09	0.109	0.011881
106013	1420697	3955432	475.771	475.88	0.109	0.011881
105933	1421487	3951843	476.001	476.11	0.109	0.011881
105684	1427155	3940720	487.891	488	0.109	0.011881
105586	1430449	3937332	491.981	492.09	0.109	0.011881
100692	1343995	3958022	435.24	435.35	0.11	0.0121
100624	1345378	3960503	436.99	437.1	0.11	0.0121
103953	1373735	3951446	446.66	446.77	0.11	0.0121
103536	1377394	3969037	447.58	447.69	0.11	0.0121
104126	1372900	3948073	451.69	451.8	0.11	0.0121
107514	1399480	3960188	458.77	458.88	0.11	0.0121
101156	1336337	3950531	432.999	433.11	0.111	0.012321
100775	1341781	3955100	433.019	433.13	0.111	0.012321
101285	1343011	3956487	433.439	433.55	0.111	0.012321
100868	1338884	3952239	433.459	433.57	0.111	0.012321
104228	1372756	3947804	451.229	451.34	0.111	0.012321
104015	1373681	3950017	453.709	453.82	0.111	0.012321
107627	1395980	3955965	456.709	456.82	0.111	0.012321
107669	1395319	3953720	459.699	459.81	0.111	0.012321
107482	1400996	3960856	460.469	460.58	0.111	0.012321
106229	1421783	3970000	472.039	472.15	0.111	0.012321
106104	1420788	3960080	474.979	475.09	0.111	0.012321
106025	1420712	3956044	475.369	475.48	0.111	0.012321
101070	1333777	3947532	549.499	549.61	0.111	0.012321
100877	1338501	3952071	432.708	432.82	0.112	0.012544
100831	1340187	3953275	432.828	432.94	0.112	0.012544
100118	1358193	3962889	435.918	436.03	0.112	0.012544
103644	1374956	3964554	445.058	445.17	0.112	0.012544
104139	1372783	3947873	451.378	451.49	0.112	0.012544
107577	1396704	3958403	460.518	460.63	0.112	0.012544
106041	1420728	3956838	474.858	474.97	0.112	0.012544
106974	1420699	3956348	475.158	475.27	0.112	0.012544
105922	1421660	3951274	477.408	477.52	0.112	0.012544
101858	1357268	3974477	489.018	489.13	0.112	0.012544
101148	1336052	3950241	433.147	433.26	0.113	0.012769
100064	1358105	3960544	436.907	437.02	0.113	0.012769
100237	1358339	3968361	437.187	437.3	0.113	0.012769
103894	1373798	3953690	447.177	447.29	0.113	0.012769
106232	1421785	3970069	471.827	471.94	0.113	0.012769
106234	1421787	3970142	472.027	472.14	0.113	0.012769
106872	1420862	3963982	472.037	472.15	0.113	0.012769
106228	1421781	3969960	472.277	472.39	0.113	0.012769
105890	1422168	3949655	474.757	474.87	0.113	0.012769
106986	1420690	3955769	475.337	475.45	0.113	0.012769
106699	1421843	3974112	479.627	479.74	0.113	0.012769
101052	1333195	3947193	548.157	548.27	0.113	0.012769
100772	1341866	3955198	432.976	433.09	0.114	0.012996
104099	1373146	3948510	451.926	452.04	0.114	0.012996

107550	1397692	3959375	458.096	458.21	0.114	0.012996
106944	1420726	3957673	474.886	475	0.114	0.012996
105837	1423481	3947125	476.766	476.88	0.114	0.012996
106327	1421868	3974417	481.346	481.46	0.114	0.012996
105496	1429301	3933541	493.176	493.29	0.114	0.012996
101279	1342273	3955643	432.945	433.06	0.115	0.013225
100219	1358312	3967578	435.625	435.74	0.115	0.013225
106212	1421664	3968611	466.065	466.18	0.115	0.013225
106168	1420918	3966013	472.465	472.58	0.115	0.013225
106976	1420699	3956236	475.485	475.6	0.115	0.013225
105817	1423913	3946296	475.945	476.06	0.115	0.013225
106323	1421863	3974199	480.215	480.33	0.115	0.013225
105574	1430495	3936662	493.285	493.4	0.115	0.013225
101264	1341863	3955173	432.934	433.05	0.116	0.013456
100072	1358138	3960904	436.634	436.75	0.116	0.013456
107502	1400075	3960462	459.404	459.52	0.116	0.013456
103633	1374980	3965008	446.083	446.2	0.117	0.013689
105821	1423845	3946476	476.263	476.38	0.117	0.013689
100909	1337299	3951478	433.282	433.4	0.118	0.013924
101746	1358366	3970061	438.522	438.64	0.118	0.013924
104235	1372813	3947900	451.522	451.64	0.118	0.013924
104088	1373256	3948707	452.102	452.22	0.118	0.013924
100510	1347693	3964779	453.192	453.31	0.118	0.013924
104008	1373685	3950144	453.502	453.62	0.118	0.013924
107578	1396688	3958367	460.533	460.65	0.118	0.013924
106779	1421538	3968351	463.272	463.39	0.118	0.013924
105867	1422591	3948435	478.592	478.71	0.118	0.013924
101318	1344179	3958324	436.291	436.41	0.119	0.014161
103762	1373704	3960058	444.081	444.2	0.119	0.014161
103753	1373944	3960192	444.331	444.45	0.119	0.014161
100105	1358175	3962329	436.709	436.83	0.12	0.0144
101433	1346883	3963211	438.67	438.79	0.12	0.0144
104119	1372960	3948179	451.71	451.83	0.12	0.0144
104103	1373107	3948442	452.02	452.14	0.12	0.0144
103430	1379602	3971934	452.76	452.88	0.12	0.0144
103989	1373703	3950527	453.88	454	0.12	0.0144
106832	1420903	3965998	472.63	472.75	0.12	0.0144
106305	1421843	3973201	475.74	475.86	0.12	0.0144
106314	1421853	3973677	478.04	478.16	0.12	0.0144
101867	1356976	3974864	494.6	494.72	0.12	0.0144
101039	1333592	3947439	543.25	543.37	0.12	0.0144
101259	1341720	3955010	432.989	433.11	0.121	0.014641
100696	1343915	3957878	434.999	435.12	0.121	0.014641
101772	1358386	3971057	438.829	438.95	0.121	0.014641
101775	1358373	3971240	439.609	439.73	0.121	0.014641
103407	1379603	3972605	457.699	457.82	0.121	0.014641
106796	1421192	3967769	470.059	470.18	0.121	0.014641
106963	1420707	3956791	475.039	475.16	0.121	0.014641
106304	1421842	3973158	475.919	476.04	0.121	0.014641
105711	1425787	3941422	485.599	485.72	0.121	0.014641
105459	1429128	3932256	494.969	495.09	0.121	0.014641
101190	1337464	3951567	432.538	432.66	0.122	0.014884

104496	1373815	3953840	447.048	447.17	0.122	0.014884
106788	1421352	3968033	465.688	465.81	0.122	0.014884
106983	1420692	3955884	475.318	475.44	0.122	0.014884
106024	1420711	3956003	475.388	475.51	0.122	0.014884
105861	1422742	3948154	478.458	478.58	0.122	0.014884
105758	1423805	3943056	484.228	484.35	0.122	0.014884
101231	1340249	3953327	432.867	432.99	0.123	0.015129
100677	1344294	3958558	436.717	436.84	0.123	0.015129
103631	1374984	3965092	445.947	446.07	0.123	0.015129
104492	1373812	3953676	447.217	447.34	0.123	0.015129
104257	1372997	3948227	451.807	451.93	0.123	0.015129
107517	1399320	3960114	458.818	458.94	0.123	0.015129
106213	1421688	3968687	467.337	467.46	0.123	0.015129
105855	1422941	3947850	477.647	477.77	0.123	0.015129
100769	1341953	3955297	433.096	433.22	0.124	0.015376
100695	1343935	3957914	435.076	435.2	0.124	0.015376
103823	1373873	3957013	445.126	445.25	0.124	0.015376
103545	1377088	3968839	447.416	447.54	0.124	0.015376
104134	1372827	3947950	451.436	451.56	0.124	0.015376
101472	1347852	3964988	453.756	453.88	0.124	0.015376
106783	1421454	3968205	463.356	463.48	0.124	0.015376
105914	1421790	3950878	477.426	477.55	0.124	0.015376
103354	1379880	3974913	498.056	498.18	0.124	0.015376
101201	1337883	3951779	432.335	432.46	0.125	0.015625
100680	1344232	3958448	436.685	436.81	0.125	0.015625
101322	1344258	3958466	436.725	436.85	0.125	0.015625
100564	1346648	3962809	436.905	437.03	0.125	0.015625
100561	1346675	3962864	437.055	437.18	0.125	0.015625
103853	1373842	3955785	445.985	446.11	0.125	0.015625
104135	1372818	3947934	451.505	451.63	0.125	0.015625
100483	1348264	3965802	456.285	456.41	0.125	0.015625
106821	1420915	3966795	472.245	472.37	0.125	0.015625
105675	1427535	3940402	487.745	487.87	0.125	0.015625
105622	1429393	3938787	490.125	490.25	0.125	0.015625
106094	1420777	3959563	475.144	475.27	0.126	0.015876
106703	1421841	3973897	478.734	478.86	0.126	0.015876
105570	1430462	3936439	493.584	493.71	0.126	0.015876
100753	1342430	3955843	433.053	433.18	0.127	0.016129
100568	1346611	3962721	436.983	437.11	0.127	0.016129
103901	1373791	3953405	447.373	447.5	0.127	0.016129
104234	1372805	3947886	451.493	451.62	0.127	0.016129
100391	1351484	3969116	452.343	452.47	0.127	0.016129
104300	1373384	3948912	452.633	452.76	0.127	0.016129
106141	1420898	3964845	470.433	470.56	0.127	0.016129
106109	1420793	3960330	474.923	475.05	0.127	0.016129
106697	1421846	3974243	480.323	480.45	0.127	0.016129
106324	1421864	3974242	480.363	480.49	0.127	0.016129
101282	1342448	3955843	432.952	433.08	0.128	0.016384
101288	1343263	3956799	433.392	433.52	0.128	0.016384
100155	1358224	3964160	435.312	435.44	0.128	0.016384
101316	1344120	3958218	436.162	436.29	0.128	0.016384
104509	1373831	3954479	446.752	446.88	0.128	0.016384

104367	1373701	3950209	453.682	453.81	0.128	0.016384
107480	1401105	3960897	460.192	460.32	0.128	0.016384
107481	1401032	3960870	460.232	460.36	0.128	0.016384
105989	1420318	3954104	474.722	474.85	0.128	0.016384
105671	1427672	3940283	488.052	488.18	0.128	0.016384
105618	1429511	3938686	490.072	490.2	0.128	0.016384
101204	1337998	3951832	432.251	432.38	0.129	0.016641
103512	1378040	3969461	449.641	449.77	0.129	0.016641
101471	1347832	3964951	453.631	453.76	0.129	0.016641
106310	1421848	3973461	477.191	477.32	0.129	0.016641
105662	1427998	3939997	488.391	488.52	0.129	0.016641
105601	1430102	3938089	490.921	491.05	0.129	0.016641
100690	1344035	3958093	435.86	435.99	0.13	0.0169
100238	1358341	3968403	437.12	437.25	0.13	0.0169
103424	1379603	3972059	453.46	453.59	0.13	0.0169
100826	1340332	3953441	432.859	432.99	0.131	0.017161
100811	1340764	3953937	432.949	433.08	0.131	0.017161
100693	1343975	3957986	435.169	435.3	0.131	0.017161
101406	1346128	3961796	436.609	436.74	0.131	0.017161
101321	1344238	3958430	436.679	436.81	0.131	0.017161
100355	1353046	3969061	444.389	444.52	0.131	0.017161
103674	1374771	3963438	447.929	448.06	0.131	0.017161
104143	1372748	3947813	451.119	451.25	0.131	0.017161
104268	1373087	3948385	452.039	452.17	0.131	0.017161
106873	1420861	3963938	472.119	472.25	0.131	0.017161
106820	1420916	3966837	472.259	472.39	0.131	0.017161
105881	1422308	3949207	476.069	476.2	0.131	0.017161
105567	1430419	3936264	494.039	494.17	0.131	0.017161
101225	1340056	3953106	432.947	433.08	0.132	0.017424
100216	1358309	3967445	435.298	435.43	0.132	0.017424
104258	1373006	3948242	451.828	451.96	0.132	0.017424
106928	1420741	3958417	473.368	473.5	0.132	0.017424
106912	1420756	3959208	474.219	474.35	0.132	0.017424
100739	1342846	3956319	432.967	433.1	0.133	0.017689
100740	1342817	3956285	432.997	433.13	0.133	0.017689
100075	1358141	3961034	436.297	436.43	0.133	0.017689
104272	1373119	3948441	452.027	452.16	0.133	0.017689
106230	1421784	3970022	472.007	472.14	0.133	0.017689
106982	1420693	3955962	475.327	475.46	0.133	0.017689
105560	1430295	3935943	494.337	494.47	0.133	0.017689
105480	1429166	3933113	494.877	495.01	0.133	0.017689
100900	1337624	3951677	432.806	432.94	0.134	0.017956
101313	1344061	3958114	435.766	435.9	0.134	0.017956
103956	1373733	3951361	446.706	446.84	0.134	0.017956
104362	1373698	3950103	453.626	453.76	0.134	0.017956
107582	1396618	3958174	460.056	460.19	0.134	0.017956
106008	1420678	3955201	475.836	475.97	0.134	0.017956
101211	1338276	3951957	432.555	432.69	0.135	0.018225
103958	1373733	3951305	446.715	446.85	0.135	0.018225
107493	1400497	3960651	460.305	460.44	0.135	0.018225
106786	1421393	3968100	464.655	464.79	0.135	0.018225
106845	1420893	3965381	470.375	470.51	0.135	0.018225

106164	1420916	3965834	471.955	472.09	0.135	0.018225
106913	1420755	3959165	474.036	474.17	0.135	0.018225
106988	1420687	3955653	475.255	475.39	0.135	0.018225
100563	1346656	3962827	436.944	437.08	0.136	0.018496
100171	1358242	3964868	437.414	437.55	0.136	0.018496
104256	1372989	3948212	451.774	451.91	0.136	0.018496
104248	1372922	3948095	451.854	451.99	0.136	0.018496
104363	1373698	3950124	453.674	453.81	0.136	0.018496
104350	1373689	3949848	453.904	454.04	0.136	0.018496
107505	1399913	3960389	459.064	459.2	0.136	0.018496
101314	1344081	3958149	435.933	436.07	0.137	0.018769
105876	1422388	3948952	477.103	477.24	0.137	0.018769
106336	1421877	3974891	485.033	485.17	0.137	0.018769
105559	1430258	3935868	493.953	494.09	0.137	0.018769
105457	1429139	3932187	494.883	495.02	0.137	0.018769
100685	1344133	3958268	436.222	436.36	0.138	0.019044
100620	1345451	3960633	436.812	436.95	0.138	0.019044
104245	1372897	3948052	451.692	451.83	0.138	0.019044
104077	1373363	3948895	452.402	452.54	0.138	0.019044
100434	1349305	3967668	459.662	459.8	0.138	0.019044
106810	1420975	3967252	471.692	471.83	0.138	0.019044
106829	1420910	3966468	472.642	472.78	0.138	0.019044
106896	1420768	3959989	474.842	474.98	0.138	0.019044
106897	1420767	3959953	474.842	474.98	0.138	0.019044
105830	1423663	3946845	476.802	476.94	0.138	0.019044
105406	1429124	3930471	496.932	497.07	0.138	0.019044
104122	1372933	3948132	451.781	451.92	0.139	0.019321
104056	1373599	3949306	452.971	453.11	0.139	0.019321
107666	1395369	3953892	459.141	459.28	0.139	0.019321
106027	1420714	3956124	475.431	475.57	0.139	0.019321
105880	1422321	3949165	476.181	476.32	0.139	0.019321
101188	1337387	3951520	432.77	432.91	0.14	0.0196
103763	1373682	3960057	443.95	444.09	0.14	0.0196
104298	1373363	3948874	452.54	452.68	0.14	0.0196
100513	1347629	3964663	452.69	452.83	0.14	0.0196
104019	1373680	3949936	453.84	453.98	0.14	0.0196
103421	1379603	3972135	453.88	454.02	0.14	0.0196
106717	1421826	3973194	475.69	475.83	0.14	0.0196
100235	1358334	3968277	437.609	437.75	0.141	0.019881
103905	1373787	3953250	446.549	446.69	0.141	0.019881
103529	1377571	3969154	447.729	447.87	0.141	0.019881
107488	1400749	3960758	460.499	460.64	0.141	0.019881
106801	1421102	3967599	470.979	471.12	0.141	0.019881
106874	1420860	3963853	472.469	472.61	0.141	0.019881
106074	1420757	3958526	473.019	473.16	0.141	0.019881
105820	1423859	3946440	476.179	476.32	0.141	0.019881
105597	1430218	3937911	490.829	490.97	0.141	0.019881
101237	1340414	3953515	432.858	433	0.142	0.020164
100615	1345551	3960802	436.918	437.06	0.142	0.020164
104284	1373221	3948624	451.818	451.96	0.142	0.020164
107476	1401289	3960968	460.168	460.31	0.142	0.020164
106076	1420759	3958657	472.688	472.83	0.142	0.020164

105889	1422181	3949614	474.858	475	0.142	0.020164
106906	1420761	3959538	475.128	475.27	0.142	0.020164
106031	1420717	3956326	475.258	475.4	0.142	0.020164
105829	1423681	3946813	476.738	476.88	0.142	0.020164
106710	1421833	3973564	477.738	477.88	0.142	0.020164
105692	1426779	3941003	488.298	488.44	0.142	0.020164
105512	1429516	3934109	493.798	493.94	0.142	0.020164
100892	1337925	3951817	432.537	432.68	0.143	0.020449
104017	1373681	3949977	453.737	453.88	0.143	0.020449
106208	1421589	3968420	464.267	464.41	0.143	0.020449
106098	1420781	3959758	474.987	475.13	0.143	0.020449
101041	1333518	3947396	541.327	541.47	0.143	0.020449
101197	1337731	3951711	432.526	432.67	0.144	0.020736
100812	1340735	3953903	432.926	433.07	0.144	0.020736
104130	1372866	3948015	451.546	451.69	0.144	0.020736
106171	1420921	3966161	472.566	472.71	0.144	0.020736
106887	1420846	3963256	473.756	473.9	0.144	0.020736
105836	1423503	3947095	476.926	477.07	0.144	0.020736
101423	1346651	3962757	436.735	436.88	0.145	0.021025
103892	1373800	3953778	447.105	447.25	0.145	0.021025
104041	1373668	3949557	453.175	453.32	0.145	0.021025
103413	1379603	3972391	455.725	455.87	0.145	0.021025
106895	1420769	3960024	474.855	475	0.145	0.021025
105844	1423276	3947402	476.505	476.65	0.145	0.021025
105689	1426937	3940893	488.355	488.5	0.145	0.021025
101068	1333701	3947489	546.795	546.94	0.145	0.021025
100889	1338036	3951866	432.504	432.65	0.146	0.021316
101228	1340167	3953232	432.794	432.94	0.146	0.021316
100856	1339363	3952495	434.584	434.73	0.146	0.021316
104117	1372978	3948211	451.734	451.88	0.146	0.021316
104306	1373446	3949021	452.844	452.99	0.146	0.021316
105907	1421900	3950523	476.374	476.52	0.146	0.021316
101061	1333468	3947352	540.454	540.6	0.146	0.021316
100678	1344273	3958521	436.683	436.83	0.147	0.021609
104281	1373195	3948577	451.813	451.96	0.147	0.021609
104070	1373436	3949020	452.743	452.89	0.147	0.021609
106083	1420766	3958997	473.303	473.45	0.147	0.021609
106023	1420709	3955921	475.173	475.32	0.147	0.021609
106721	1421823	3972991	475.323	475.47	0.147	0.021609
106301	1421839	3972987	475.464	475.61	0.147	0.021609
5535	1388893	4005734	741.603	741.75	0.147	0.021609
104110	1373045	3948329	451.932	452.08	0.148	0.021904
107648	1395661	3954882	458.082	458.23	0.148	0.021904
107680	1395095	3953196	462.542	462.69	0.148	0.021904
106217	1421732	3968874	469.972	470.12	0.148	0.021904
105897	1422054	3950018	473.852	474	0.148	0.021904
101856	1357333	3974420	487.442	487.59	0.148	0.021904
100682	1344192	3958376	436.551	436.7	0.149	0.022201
104115	1372996	3948244	451.781	451.93	0.149	0.022201
104369	1373703	3950250	453.631	453.78	0.149	0.022201
106785	1421413	3968135	464.111	464.26	0.149	0.022201
106894	1420770	3960060	474.781	474.93	0.149	0.022201

106975	1420699	3956274	475.351	475.5	0.149	0.022201
106704	1421840	3973855	478.671	478.82	0.149	0.022201
105461	1429110	3932360	495.471	495.62	0.149	0.022201
104009	1373684	3950125	453.54	453.69	0.15	0.0225
107490	1400642	3960715	460.38	460.53	0.15	0.0225
106095	1420778	3959601	475.27	475.42	0.15	0.0225
105676	1427478	3940450	487.71	487.86	0.15	0.0225
100686	1344113	3958233	436.229	436.38	0.151	0.022801
101458	1347520	3964394	448.439	448.59	0.151	0.022801
104265	1373063	3948343	451.969	452.12	0.151	0.022801
104081	1373324	3948827	452.299	452.45	0.151	0.022801
107456	1402274	3961336	461.559	461.71	0.151	0.022801
106923	1420744	3958644	472.89	473.04	0.151	0.022801
103961	1373732	3951222	446.668	446.82	0.152	0.023104
104089	1373246	3948688	452.038	452.19	0.152	0.023104
103409	1379603	3972534	457.158	457.31	0.152	0.023104
107527	1398822	3959889	458.108	458.26	0.152	0.023104
107688	1394833	3952910	463.128	463.28	0.152	0.023104
106712	1421832	3973442	477.018	477.17	0.152	0.023104
101024	1334226	3947807	566.348	566.5	0.152	0.023104
100882	1338301	3951982	432.487	432.64	0.153	0.023409
101234	1340332	3953422	432.897	433.05	0.153	0.023409
104132	1372847	3947982	451.517	451.67	0.153	0.023409
104107	1373071	3948377	451.947	452.1	0.153	0.023409
104273	1373127	3948456	451.977	452.13	0.153	0.023409
104030	1373673	3949732	453.357	453.51	0.153	0.023409
100544	1346969	3963431	439.406	439.56	0.154	0.023716
104253	1372962	3948167	451.767	451.92	0.154	0.023716
104276	1373152	3948500	451.806	451.96	0.154	0.023716
101476	1347956	3965174	454.266	454.42	0.154	0.023716
106157	1420910	3965549	470.556	470.71	0.154	0.023716
106838	1420898	3965697	471.206	471.36	0.154	0.023716
106178	1420936	3966870	471.636	471.79	0.154	0.023716
105608	1429850	3938376	491.086	491.24	0.154	0.023716
105481	1429179	3933162	494.626	494.78	0.154	0.023716
100814	1340678	3953836	432.945	433.1	0.155	0.024025
100124	1358202	3963122	435.445	435.6	0.155	0.024025
100608	1345691	3961051	436.915	437.07	0.155	0.024025
104131	1372857	3947999	451.545	451.7	0.155	0.024025
104073	1373404	3948965	452.655	452.81	0.155	0.024025
103412	1379603	3972427	456.065	456.22	0.155	0.024025
106085	1420769	3959120	473.835	473.99	0.155	0.024025
105905	1421925	3950439	476.085	476.24	0.155	0.024025
106328	1421869	3974461	481.745	481.9	0.155	0.024025
105450	1429153	3931869	495.385	495.54	0.155	0.024025
103628	1374991	3965221	445.664	445.82	0.156	0.024336
104121	1372942	3948148	451.744	451.9	0.156	0.024336
104001	1373688	3950282	453.504	453.66	0.156	0.024336
106116	1420871	3963688	473.124	473.28	0.156	0.024336
100898	1337699	3951714	433.013	433.17	0.157	0.024649
100759	1342246	3955633	433.083	433.24	0.157	0.024649
103907	1373784	3953170	446.893	447.05	0.157	0.024649

100502	1347865	3965086	454.163	454.32	0.157	0.024649
105904	1421938	3950396	475.903	476.06	0.157	0.024649
105856	1422890	3947923	477.913	478.07	0.157	0.024649
105568	1430442	3936352	493.783	493.94	0.157	0.024649
103525	1377778	3969286	447.642	447.8	0.158	0.024964
104251	1372946	3948138	451.832	451.99	0.158	0.024964
104087	1373267	3948725	452.132	452.29	0.158	0.024964
101469	1347771	3964842	453.362	453.52	0.158	0.024964
101082	1334237	3947799	566.952	567.11	0.158	0.024964
100872	1338711	3952162	433.061	433.22	0.159	0.025281
101312	1344041	3958079	435.761	435.92	0.159	0.025281
101598	1352359	3969062	449.041	449.2	0.159	0.025281
104297	1373352	3948855	452.481	452.64	0.159	0.025281
105811	1424002	3946002	475.771	475.93	0.159	0.025281
105595	1430281	3937797	491.101	491.26	0.159	0.025281
105572	1430486	3936572	493.441	493.6	0.159	0.025281
101326	1344339	3958609	436.87	437.03	0.16	0.0256
106890	1420772	3960207	474.56	474.72	0.16	0.0256
101216	1338483	3952048	432.629	432.79	0.161	0.025921
101257	1341632	3954909	432.889	433.05	0.161	0.025921
101283	1342565	3955978	432.929	433.09	0.161	0.025921
105945	1421300	3952424	474.739	474.9	0.161	0.025921
101437	1346970	3963391	438.838	439	0.162	0.026244
101465	1347669	3964660	452.508	452.67	0.162	0.026244
100446	1349046	3967200	460.238	460.4	0.162	0.026244
107677	1395160	3953307	461.798	461.96	0.162	0.026244
105593	1430320	3937717	491.408	491.57	0.162	0.026244
104237	1372829	3947928	451.617	451.78	0.163	0.026569
106092	1420775	3959484	475.177	475.34	0.163	0.026569
104247	1372914	3948081	451.806	451.97	0.164	0.026896
104307	1373456	3949038	452.786	452.95	0.164	0.026896
106812	1420958	3967170	471.866	472.03	0.164	0.026896
106311	1421850	3973548	477.676	477.84	0.164	0.026896
101084	1334312	3947845	569.976	570.14	0.164	0.026896
100830	1340216	3953309	432.725	432.89	0.165	0.027225
105807	1424042	3945807	476.125	476.29	0.165	0.027225
101054	1333270	3947237	544.385	544.55	0.165	0.027225
100730	1343105	3956621	433.004	433.17	0.166	0.027556
107658	1395492	3954304	458.074	458.24	0.166	0.027556
107492	1400570	3960684	460.354	460.52	0.166	0.027556
106102	1420785	3959957	475.054	475.22	0.166	0.027556
106028	1420715	3956204	475.384	475.55	0.166	0.027556
105883	1422282	3949288	475.814	475.98	0.166	0.027556
105835	1423545	3947034	477.024	477.19	0.166	0.027556
106707	1421835	3973688	478.084	478.25	0.166	0.027556
105565	1430392	3936179	494.134	494.3	0.166	0.027556
101319	1344199	3958359	436.423	436.59	0.167	0.027889
100514	1347607	3964625	452.313	452.48	0.167	0.027889
106211	1421651	3968573	465.563	465.73	0.167	0.027889
104277	1373160	3948515	451.832	452	0.168	0.028224
104354	1373693	3949934	453.902	454.07	0.168	0.028224
106070	1420753	3958311	473.652	473.82	0.168	0.028224

101202	1337921	3951796	432.211	432.38	0.169	0.028561
100777	1341725	3955036	433.111	433.28	0.169	0.028561
104495	1373814	3953799	447.091	447.26	0.169	0.028561
104113	1373016	3948278	451.841	452.01	0.169	0.028561
105871	1422497	3948644	478.311	478.48	0.169	0.028561
104093	1373203	3948612	451.91	452.08	0.17	0.0289
107621	1396060	3956241	456.87	457.04	0.17	0.0289
106804	1421054	3967490	470.69	470.86	0.17	0.0289
106086	1420769	3959162	473.97	474.14	0.17	0.0289
101307	1343941	3957901	435.019	435.19	0.171	0.029241
105825	1423766	3946650	476.389	476.56	0.171	0.029241
105917	1421738	3951034	477.469	477.64	0.171	0.029241
100841	1339885	3952934	433.288	433.46	0.172	0.029584
104149	1372696	3947722	450.858	451.03	0.172	0.029584
106089	1420772	3959325	474.508	474.68	0.172	0.029584
106718	1421825	3973153	475.758	475.93	0.172	0.029584
105798	1424089	3945334	476.878	477.05	0.172	0.029584
100873	1338668	3952143	432.897	433.07	0.173	0.029929
104226	1372741	3947777	451.207	451.38	0.173	0.029929
104137	1372801	3947904	451.457	451.63	0.173	0.029929
104100	1373137	3948493	451.957	452.13	0.173	0.029929
104102	1373117	3948458	451.977	452.15	0.173	0.029929
106921	1420747	3958758	472.788	472.96	0.173	0.029929
105909	1421864	3950645	476.817	476.99	0.173	0.029929
105866	1422611	3948394	478.577	478.75	0.173	0.029929
106321	1421861	3974067	479.457	479.63	0.173	0.029929
101861	1357147	3974602	491.577	491.75	0.173	0.029929
100842	1339853	3952900	433.366	433.54	0.174	0.030276
100356	1353002	3969062	444.396	444.57	0.174	0.030276
104048	1373659	3949445	453.186	453.36	0.174	0.030276
104335	1373678	3949544	453.286	453.46	0.174	0.030276
104355	1373693	3949956	453.866	454.04	0.174	0.030276
106709	1421834	3973605	477.846	478.02	0.174	0.030276
105602	1430077	3938123	490.986	491.16	0.174	0.030276
101037	1333705	3947504	547.166	547.34	0.174	0.030276
104261	1373031	3948285	451.895	452.07	0.175	0.030625
104290	1373279	3948727	452.065	452.24	0.175	0.030625
104036	1373669	3949633	453.215	453.39	0.175	0.030625
104361	1373698	3950082	453.605	453.78	0.175	0.030625
106206	1421552	3968345	463.805	463.98	0.175	0.030625
105895	1422092	3949898	473.875	474.05	0.175	0.030625
106016	1420702	3955592	475.205	475.38	0.175	0.030625
106320	1421860	3974023	479.255	479.43	0.175	0.030625
107573	1396796	3958579	460.194	460.37	0.176	0.030976
107574	1396776	3958545	460.204	460.38	0.176	0.030976
106915	1420753	3959039	473.544	473.72	0.176	0.030976
106973	1420700	3956385	475.074	475.25	0.176	0.030976
105800	1424082	3945465	476.714	476.89	0.176	0.030976
105858	1422838	3947999	478.074	478.25	0.176	0.030976
100903	1337513	3951615	433.173	433.35	0.177	0.031329
100515	1347585	3964586	451.873	452.05	0.177	0.031329
104342	1373685	3949686	453.383	453.56	0.177	0.031329

107541	1398119	3959570	457.643	457.82	0.177	0.031329
106778	1421769	3970217	472.123	472.3	0.177	0.031329
105903	1421964	3950310	475.393	475.57	0.177	0.031329
101325	1344318	3958573	436.732	436.91	0.178	0.031684
107477	1401214	3960939	460.172	460.35	0.178	0.031684
106173	1420929	3966656	472.242	472.42	0.178	0.031684
100846	1339721	3952771	433.721	433.9	0.179	0.032041
103896	1373796	3953605	447.281	447.46	0.179	0.032041
104229	1372764	3947817	451.291	451.47	0.179	0.032041
106205	1421532	3968309	463.671	463.85	0.179	0.032041
105585	1430459	3937287	492.061	492.24	0.179	0.032041
101233	1340304	3953391	432.95	433.13	0.18	0.0324
100473	1348471	3966172	457.291	457.47	0.18	0.0324
107498	1400274	3960553	459.85	460.03	0.18	0.0324
101074	1333927	3947619	555.27	555.45	0.18	0.0324
100834	1340098	3953173	432.809	432.99	0.181	0.032761
101430	1346814	3963073	438.209	438.39	0.181	0.032761
104491	1373811	3953634	447.249	447.43	0.181	0.032761
104112	1373025	3948295	451.849	452.03	0.181	0.032761
104288	1373259	3948692	451.869	452.05	0.181	0.032761
104028	1373675	3949769	453.419	453.6	0.181	0.032761
107576	1396739	3958475	460.299	460.48	0.181	0.032761
106831	1420903	3966035	472.449	472.63	0.181	0.032761
105838	1423459	3947155	476.629	476.81	0.181	0.032761
104267	1373079	3948371	451.998	452.18	0.182	0.033124
104321	1373597	3949286	453.188	453.37	0.182	0.033124
106119	1420875	3963843	472.518	472.7	0.182	0.033124
106908	1420760	3959419	475.058	475.24	0.182	0.033124
105916	1421751	3950995	477.448	477.63	0.182	0.033124
106319	1421859	3973979	479.118	479.3	0.182	0.033124
105599	1430174	3937983	490.858	491.04	0.182	0.033124
104239	1372845	3947956	451.637	451.82	0.183	0.033489
104254	1372971	3948182	451.697	451.88	0.183	0.033489
104038	1373669	3949604	453.137	453.32	0.183	0.033489
104323	1373616	3949321	453.217	453.4	0.183	0.033489
103416	1379603	3972287	454.637	454.82	0.183	0.033489
100791	1341335	3954590	432.736	432.92	0.184	0.033856
104243	1372875	3948011	451.685	451.87	0.185	0.034225
104005	1373686	3950203	453.445	453.63	0.185	0.034225
104022	1373678	3949878	453.905	454.09	0.185	0.034225
106166	1420918	3965940	472.405	472.59	0.185	0.034225
101221	1339919	3952950	433.594	433.78	0.186	0.034596
104282	1373204	3948593	451.834	452.02	0.186	0.034596
104084	1373295	3948776	452.294	452.48	0.186	0.034596
106850	1420888	3965093	470.414	470.6	0.186	0.034596
106823	1420913	3966714	472.234	472.42	0.186	0.034596
100158	1358226	3964281	436.303	436.49	0.187	0.034969
104125	1372908	3948087	451.743	451.93	0.187	0.034969
103418	1379603	3972224	454.423	454.61	0.187	0.034969
101196	1337694	3951692	432.472	432.66	0.188	0.035344
100076	1358142	3961078	436.262	436.45	0.188	0.035344
100524	1347389	3964235	443.962	444.15	0.188	0.035344

100166	1358236	3964638	437.171	437.36	0.189	0.035721
100546	1346929	3963348	438.831	439.02	0.189	0.035721
103899	1373793	3953483	447.461	447.65	0.189	0.035721
107466	1401735	3961137	460.351	460.54	0.189	0.035721
106960	1420711	3956937	474.951	475.14	0.189	0.035721
106716	1421827	3973235	475.621	475.81	0.189	0.035721
100516	1347563	3964547	451.29	451.48	0.19	0.0361
104136	1372809	3947919	451.47	451.66	0.19	0.0361
104071	1373425	3949002	452.7	452.89	0.19	0.0361
104313	1373514	3949140	452.94	453.13	0.19	0.0361
106115	1420870	3963649	473.32	473.51	0.19	0.0361
106882	1420851	3963450	473.62	473.81	0.19	0.0361
105816	1423926	3946259	475.83	476.02	0.19	0.0361
105828	1423698	3946781	476.64	476.83	0.19	0.0361
104301	1373395	3948931	452.659	452.85	0.191	0.036481
104333	1373673	3949502	453.419	453.61	0.191	0.036481
103417	1379603	3972255	454.499	454.69	0.191	0.036481
106121	1420877	3963954	472.179	472.37	0.191	0.036481
105834	1423565	3947003	476.969	477.16	0.191	0.036481
104494	1373813	3953758	447.118	447.31	0.192	0.036864
104493	1373812	3953717	447.117	447.31	0.193	0.037249
104101	1373127	3948476	451.967	452.16	0.193	0.037249
103410	1379603	3972499	456.857	457.05	0.193	0.037249
103402	1379603	3972793	459.447	459.64	0.193	0.037249
106884	1420849	3963372	473.667	473.86	0.193	0.037249
105937	1421429	3952033	475.587	475.78	0.193	0.037249
105453	1429155	3932012	495.077	495.27	0.193	0.037249
104080	1373334	3948844	452.286	452.48	0.194	0.037636
106993	1420680	3955418	475.346	475.54	0.194	0.037636
103415	1379603	3972321	454.945	455.14	0.195	0.038025
107501	1400115	3960481	459.414	459.61	0.196	0.038416
106713	1421831	3973401	476.624	476.82	0.196	0.038416
103891	1373801	3953823	447.043	447.24	0.197	0.038809
104118	1372969	3948195	451.673	451.87	0.197	0.038809
106071	1420754	3958353	473.543	473.74	0.197	0.038809
105818	1423901	3946332	476.003	476.2	0.197	0.038809
107584	1396594	3958093	459.772	459.97	0.198	0.039204
104263	1373047	3948313	451.961	452.16	0.199	0.039601
104083	1373305	3948793	452.311	452.51	0.199	0.039601
103431	1379602	3971922	452.711	452.91	0.199	0.039601
100738	1342875	3956353	432.98	433.18	0.2	0.04
104133	1372837	3947966	451.48	451.68	0.2	0.04
105815	1423949	3946186	475.6	475.8	0.2	0.04
105809	1424019	3945926	476.07	476.27	0.2	0.04
104250	1372938	3948124	451.869	452.07	0.201	0.040401
104031	1373673	3949714	453.289	453.49	0.201	0.040401
105589	1430400	3937507	491.759	491.96	0.201	0.040401
101206	1338075	3951867	432.438	432.64	0.202	0.040804
100683	1344172	3958340	436.358	436.56	0.202	0.040804
104244	1372890	3948038	451.718	451.92	0.202	0.040804
104006	1373685	3950184	453.448	453.65	0.202	0.040804
105609	1429820	3938406	490.948	491.15	0.202	0.040804

100827	1340303	3953408	432.857	433.06	0.203	0.041209
100767	1342010	3955362	433.257	433.46	0.203	0.041209
103874	1373822	3954649	446.287	446.49	0.203	0.041209
101268	1341972	3955298	432.967	433.17	0.204	0.041616
101372	1345416	3960532	437.036	437.24	0.204	0.041616
104079	1373344	3948861	452.306	452.51	0.204	0.041616
100508	1347736	3964856	453.517	453.72	0.204	0.041616
104252	1372954	3948152	451.825	452.03	0.205	0.042025
104260	1373022	3948271	451.895	452.1	0.205	0.042025
106114	1420869	3963610	473.425	473.63	0.205	0.042025
106900	1420764	3959808	474.845	475.05	0.205	0.042025
101278	1342244	3955610	432.914	433.12	0.206	0.042436
100774	1341809	3955133	433.015	433.22	0.206	0.042436
100855	1339400	3952520	434.354	434.56	0.206	0.042436
101329	1344401	3958720	436.884	437.09	0.206	0.042436
104289	1373269	3948709	451.964	452.17	0.206	0.042436
104299	1373374	3948893	452.554	452.76	0.206	0.042436
101460	1347562	3964470	450.113	450.32	0.207	0.042849
106809	1420986	3967293	471.353	471.56	0.207	0.042849
105902	1421977	3950267	475.123	475.33	0.207	0.042849
104497	1373817	3953881	447.032	447.24	0.208	0.043264
104095	1373183	3948576	451.912	452.12	0.208	0.043264
100505	1347801	3964971	453.822	454.03	0.208	0.043264
105556	1430201	3935753	493.852	494.06	0.208	0.043264
104262	1373039	3948299	451.921	452.13	0.209	0.043681
104108	1373062	3948361	451.931	452.14	0.209	0.043681
104039	1373668	3949589	453.131	453.34	0.209	0.043681
106776	1421771	3970294	472.001	472.21	0.209	0.043681
105813	1423971	3946113	475.561	475.77	0.209	0.043681
105575	1430499	3936707	493.191	493.4	0.209	0.043681
101435	1346918	3963282	438.72	438.93	0.21	0.0441
103423	1379603	3972082	453.56	453.77	0.21	0.0441
106317	1421856	3973848	478.48	478.69	0.21	0.0441
107589	1396506	3957800	458.899	459.11	0.211	0.044521
100890	1337999	3951849	432.458	432.67	0.212	0.044944
106920	1420748	3958797	472.758	472.97	0.212	0.044944
106315	1421855	3973762	478.338	478.55	0.212	0.044944
104259	1373014	3948256	451.837	452.05	0.213	0.045369
104295	1373330	3948818	452.397	452.61	0.213	0.045369
104051	1373644	3949396	453.087	453.3	0.213	0.045369
107583	1396606	3958133	459.867	460.08	0.213	0.045369
106814	1420942	3967088	471.807	472.02	0.213	0.045369
105912	1421815	3950800	477.157	477.37	0.213	0.045369
106337	1421878	3974934	485.947	486.16	0.213	0.045369
104287	1373249	3948674	451.786	452	0.214	0.045796
104106	1373080	3948393	452.006	452.22	0.214	0.045796
106784	1421434	3968170	463.626	463.84	0.214	0.045796
106091	1420774	3959405	474.966	475.18	0.214	0.045796
105485	1429233	3933248	494.527	494.74	0.214	0.045796
105407	1429107	3930508	496.887	497.1	0.214	0.045796
101459	1347541	3964432	449.365	449.58	0.215	0.046225
105437	1429141	3931288	495.575	495.79	0.215	0.046225

100897	1337736	3951732	433.024	433.24	0.216	0.046656
106219	1421744	3968945	470.684	470.9	0.216	0.046656
100845	1339755	3952803	433.533	433.75	0.217	0.047089
104279	1373177	3948546	451.823	452.04	0.217	0.047089
104346	1373687	3949765	453.553	453.77	0.217	0.047089
103411	1379603	3972463	456.463	456.68	0.217	0.047089
105793	1424090	3945067	477.723	477.94	0.217	0.047089
105569	1430453	3936395	493.683	493.9	0.217	0.047089
100733	1343020	3956521	433.182	433.4	0.218	0.047524
104090	1373235	3948668	451.972	452.19	0.218	0.047524
104266	1373071	3948357	452.012	452.23	0.218	0.047524
104037	1373669	3949619	453.201	453.42	0.219	0.047961
104032	1373672	3949696	453.341	453.56	0.219	0.047961
104340	1373685	3949647	453.351	453.57	0.219	0.047961
106989	1420687	3955614	475.171	475.39	0.219	0.047961
105831	1423644	3946876	476.831	477.05	0.219	0.047961
101330	1344422	3958757	436.8	437.02	0.22	0.0484
104280	1373186	3948561	451.8	452.02	0.22	0.0484
101226	1340111	3953169	432.819	433.04	0.221	0.048841
104271	1373111	3948427	452.029	452.25	0.221	0.048841
104072	1373415	3948984	452.669	452.89	0.221	0.048841
104024	1373678	3949842	453.809	454.03	0.221	0.048841
104351	1373690	3949869	453.959	454.18	0.221	0.048841
106201	1421429	3968132	464.089	464.31	0.221	0.048841
106899	1420765	3959845	474.799	475.02	0.221	0.048841
105824	1423782	3946616	476.399	476.62	0.221	0.048841
105410	1429045	3930647	496.889	497.11	0.221	0.048841
104075	1373383	3948929	452.498	452.72	0.222	0.049284
104327	1373646	3949389	453.318	453.54	0.222	0.049284
101370	1345376	3960461	437.277	437.5	0.223	0.049729
104291	1373289	3948745	452.147	452.37	0.223	0.049729
106204	1421491	3968237	463.457	463.68	0.223	0.049729
106911	1420756	3959251	474.317	474.54	0.223	0.049729
100840	1339916	3952968	433.266	433.49	0.224	0.050176
101222	1339974	3953012	433.326	433.55	0.224	0.050176
104360	1373697	3950062	453.606	453.83	0.224	0.050176
106202	1421450	3968167	463.716	463.94	0.224	0.050176
101426	1346727	3962901	437.375	437.6	0.225	0.050625
104105	1373089	3948409	451.965	452.19	0.225	0.050625
106781	1421497	3968277	463.095	463.32	0.225	0.050625
101773	1358385	3971075	438.934	439.16	0.226	0.051076
100835	1340068	3953139	432.793	433.02	0.227	0.051529
100519	1347498	3964431	449.133	449.36	0.227	0.051529
100847	1339687	3952740	433.832	434.06	0.228	0.051984
104347	1373687	3949786	453.642	453.87	0.228	0.051984
105887	1422219	3949492	475.242	475.47	0.228	0.051984
105814	1423960	3946149	475.562	475.79	0.228	0.051984
104120	1372951	3948163	451.701	451.93	0.229	0.052441
105754	1423860	3942826	484.691	484.92	0.229	0.052441
104128	1372884	3948045	451.66	451.89	0.23	0.0529
104304	1373425	3948986	452.84	453.07	0.23	0.0529
104326	1373639	3949372	453.3	453.53	0.23	0.0529

106981	1420694	3956001	475.45	475.68	0.23	0.0529
104274	1373135	3948470	451.899	452.13	0.231	0.053361
106199	1421368	3968029	465.579	465.81	0.231	0.053361
106984	1420691	3955846	475.349	475.58	0.231	0.053361
106708	1421834	3973646	477.929	478.16	0.231	0.053361
100723	1343298	3956868	433.078	433.31	0.232	0.053824
104109	1373054	3948345	451.938	452.17	0.232	0.053824
100511	1347672	3964741	453.078	453.31	0.232	0.053824
101220	1339801	3952828	433.687	433.92	0.233	0.054289
104233	1372796	3947872	451.457	451.69	0.233	0.054289
104330	1373662	3949444	453.297	453.53	0.233	0.054289
100507	1347758	3964894	453.747	453.98	0.233	0.054289
101026	1334150	3947762	563.407	563.64	0.233	0.054289
105872	1422463	3948730	478.066	478.3	0.234	0.054756
105752	1423890	3942738	484.896	485.13	0.234	0.054756
105564	1430377	3936137	494.226	494.46	0.234	0.054756
100552	1346805	3963109	438.295	438.53	0.235	0.055225
104337	1373682	3949587	453.215	453.45	0.235	0.055225
106893	1420770	3960096	474.725	474.96	0.235	0.055225
104358	1373696	3950020	453.694	453.93	0.236	0.055696
107451	1402545	3961409	461.284	461.52	0.236	0.055696
106918	1420750	3958916	473.154	473.39	0.236	0.055696
105753	1423875	3942782	484.745	484.98	0.236	0.055696
105577	1430503	3936840	492.804	493.04	0.236	0.055696
104357	1373695	3949999	453.743	453.98	0.237	0.056169
106176	1420933	3966799	471.902	472.14	0.238	0.056644
105790	1424077	3944885	478.382	478.62	0.238	0.056644
104086	1373276	3948742	452.211	452.45	0.239	0.057121
100467	1348596	3966396	457.861	458.1	0.239	0.057121
104124	1372916	3948102	451.77	452.01	0.24	0.0576
106977	1420698	3956197	475.49	475.73	0.24	0.0576
105827	1423732	3946717	476.5	476.74	0.24	0.0576
105571	1430471	3936483	493.53	493.77	0.24	0.0576
100728	1343161	3956690	432.939	433.18	0.241	0.058081
104127	1372892	3948059	451.689	451.93	0.241	0.058081
104319	1373577	3949250	453.149	453.39	0.241	0.058081
104026	1373676	3949805	453.569	453.81	0.241	0.058081
100838	1339978	3953035	433.087	433.33	0.243	0.059049
100557	1346720	3962950	437.107	437.35	0.243	0.059049
104338	1373683	3949607	453.237	453.48	0.243	0.059049
104023	1373678	3949860	453.857	454.1	0.243	0.059049
105877	1422374	3948996	476.927	477.17	0.243	0.059049
105875	1422417	3948863	477.397	477.64	0.243	0.059049
105768	1423803	3943663	482.147	482.39	0.243	0.059049
105578	1430503	3936885	492.687	492.93	0.243	0.059049
100850	1339582	3952652	434.596	434.84	0.244	0.059536
106991	1420684	3955496	475.366	475.61	0.244	0.059536
100857	1339326	3952471	434.535	434.78	0.245	0.060025
103893	1373799	3953733	447.105	447.35	0.245	0.060025
105762	1423780	3943292	483.524	483.77	0.246	0.060516
106875	1420859	3963811	472.603	472.85	0.247	0.061009
106081	1420765	3958913	473.013	473.26	0.247	0.061009

106916	1420752	3958998	473.443	473.69	0.247	0.061009
105566	1430406	3936221	494.104	494.35	0.247	0.061009
106876	1420858	3963770	472.841	473.09	0.249	0.062001
100861	1339174	3952381	434.3	434.55	0.25	0.0625
104293	1373309	3948781	452.33	452.58	0.25	0.0625
104053	1373628	3949362	453.02	453.27	0.25	0.0625
105810	1424011	3945965	475.93	476.18	0.25	0.0625
105795	1424092	3945156	477.41	477.66	0.25	0.0625
105591	1430370	3937593	491.69	491.94	0.25	0.0625
100688	1344074	3958163	436.049	436.3	0.251	0.063001
104286	1373239	3948656	451.819	452.07	0.251	0.063001
103419	1379603	3972193	454.309	454.56	0.251	0.063001
104052	1373637	3949379	453.088	453.34	0.252	0.063504
106683	1421860	3974972	486.728	486.98	0.252	0.063504
100555	1346750	3963007	437.617	437.87	0.253	0.064009
106198	1421348	3967996	466.117	466.37	0.253	0.064009
100837	1340008	3953069	432.986	433.24	0.254	0.064516
104294	1373320	3948799	452.366	452.62	0.254	0.064516
106782	1421476	3968241	463.156	463.41	0.254	0.064516
106994	1420678	3955380	475.195	475.45	0.255	0.065025
105792	1424088	3945021	477.835	478.09	0.255	0.065025
100859	1339251	3952425	434.574	434.83	0.256	0.065536
106834	1420902	3965886	472.304	472.56	0.256	0.065536
104058	1373579	3949270	453.043	453.3	0.257	0.066049
104315	1373534	3949174	453.093	453.35	0.257	0.066049
105580	1430496	3937018	492.552	492.81	0.258	0.066564
104249	1372930	3948110	451.891	452.15	0.259	0.067081
104033	1373671	3949679	453.22	453.48	0.26	0.0676
104343	1373685	3949707	453.38	453.64	0.26	0.0676
104352	1373691	3949890	453.97	454.23	0.26	0.0676
105891	1422155	3949696	474.62	474.88	0.26	0.0676
105573	1430491	3936617	493.34	493.6	0.26	0.0676
103890	1373802	3953869	446.959	447.22	0.261	0.068121
104270	1373103	3948413	452.039	452.3	0.261	0.068121
104324	1373624	3949338	453.209	453.47	0.261	0.068121
106824	1420912	3966675	472.169	472.43	0.261	0.068121
103422	1379603	3972108	453.728	453.99	0.262	0.068644
100843	1339821	3952867	433.406	433.67	0.264	0.069696
100858	1339288	3952448	434.666	434.93	0.264	0.069696
104138	1372792	3947889	451.406	451.67	0.264	0.069696
104305	1373436	3949003	452.826	453.09	0.264	0.069696
105757	1423813	3943009	484.356	484.62	0.264	0.069696
104043	1373667	3949525	453.215	453.48	0.265	0.070225
104344	1373686	3949727	453.465	453.73	0.265	0.070225
101223	1340001	3953044	433.25	433.52	0.269	0.072361
101227	1340139	3953201	432.74	433.01	0.27	0.0729
104328	1373652	3949407	453.32	453.59	0.27	0.0729
106849	1420890	3965174	470.429	470.7	0.271	0.073441
105796	1424092	3945245	477.079	477.35	0.271	0.073441
100517	1347542	3964508	450.708	450.98	0.272	0.073984
105832	1423605	3946940	476.838	477.11	0.272	0.073984
104242	1372868	3947997	451.627	451.9	0.273	0.074529

104322	1373607	3949304	453.197	453.47	0.273	0.074529
104040	1373668	3949573	453.043	453.32	0.277	0.076729
107575	1396757	3958510	460.193	460.47	0.277	0.076729
104339	1373684	3949627	453.292	453.57	0.278	0.077284
106097	1420781	3959719	475.072	475.35	0.278	0.077284
100852	1339511	3952597	434.491	434.77	0.279	0.077841
104064	1373507	3949143	452.88	453.16	0.28	0.0784
106787	1421372	3968067	465.06	465.34	0.28	0.0784
106014	1420699	3955471	475.659	475.94	0.281	0.078961
105823	1423814	3946547	476.349	476.63	0.281	0.078961
106338	1421878	3974976	486.949	487.23	0.281	0.078961
105607	1429910	3938316	491.178	491.46	0.282	0.079524
104066	1373476	3949091	452.757	453.04	0.283	0.080089
104325	1373632	3949355	453.246	453.53	0.284	0.080656
104104	1373098	3948425	451.983	452.27	0.286	0.081796
100853	1339474	3952570	434.502	434.79	0.288	0.082944
104317	1373555	3949211	453.172	453.46	0.288	0.082944
105756	1423823	3942963	484.512	484.8	0.288	0.082944
104063	1373517	3949161	452.951	453.24	0.289	0.083521
105587	1430439	3937376	491.911	492.2	0.289	0.083521
104336	1373680	3949566	453.14	453.43	0.29	0.0841
105581	1430493	3937062	492.48	492.77	0.29	0.0841
104055	1373609	3949325	452.947	453.24	0.293	0.085849
104060	1373558	3949232	453.077	453.37	0.293	0.085849
104035	1373670	3949648	453.247	453.54	0.293	0.085849
105908	1421876	3950605	476.707	477	0.293	0.085849
104054	1373619	3949344	452.986	453.28	0.294	0.086436
104296	1373341	3948836	452.435	452.73	0.296	0.087616
104332	1373670	3949482	453.334	453.63	0.296	0.087616
105797	1424091	3945289	476.945	477.24	0.296	0.087616
105776	1423917	3944122	479.914	480.21	0.296	0.087616
104302	1373405	3948949	452.713	453.01	0.297	0.088209
104057	1373589	3949288	453.013	453.31	0.297	0.088209
104334	1373676	3949523	453.382	453.68	0.298	0.088804
105767	1423790	3943571	482.532	482.83	0.298	0.088804
104042	1373667	3949541	453.161	453.46	0.299	0.089401
104020	1373679	3949916	453.871	454.17	0.299	0.089401
105781	1423989	3944393	479.461	479.76	0.299	0.089401
105770	1423820	3943755	481.791	482.09	0.299	0.089401
100849	1339617	3952681	434.23	434.53	0.3	0.09
105882	1422295	3949248	475.88	476.18	0.3	0.09
105826	1423749	3946684	476.428	476.73	0.302	0.091204
101224	1340029	3953075	433.127	433.43	0.303	0.091809
104349	1373689	3949828	453.818	454.12	0.303	0.091809
105913	1421802	3950839	477.257	477.56	0.303	0.091809
104292	1373299	3948764	452.254	452.56	0.306	0.093636
105775	1423893	3944031	480.274	480.58	0.306	0.093636
105759	1423797	3943102	484.134	484.44	0.306	0.093636
104076	1373373	3948912	452.4	452.71	0.31	0.0961
105755	1423847	3942872	484.649	484.96	0.311	0.096721
105579	1430501	3936929	492.607	492.92	0.313	0.097969
104331	1373666	3949463	453.296	453.61	0.314	0.098596

105592	1430338	3937676	491.467	491.78	0.314	0.098596
105588	1430414	3937464	491.785	492.1	0.315	0.099225
104098	1373155	3948526	451.904	452.22	0.316	0.099856
104309	1373475	3949072	452.824	453.14	0.316	0.099856
104311	1373495	3949106	452.833	453.15	0.317	0.100489
105760	1423786	3943197	483.813	484.13	0.317	0.100489
101409	1346215	3961950	436.652	436.97	0.318	0.101124
104312	1373505	3949123	452.882	453.2	0.318	0.101124
105822	1423830	3946511	476.291	476.61	0.319	0.101761
104046	1373664	3949479	453.139	453.46	0.321	0.103041
104329	1373657	3949425	453.329	453.65	0.321	0.103041
104021	1373679	3949897	453.869	454.19	0.321	0.103041
104269	1373095	3948399	452.058	452.38	0.322	0.103684
104069	1373446	3949038	452.697	453.02	0.323	0.104329
104114	1373006	3948261	451.786	452.11	0.324	0.104976
106078	1420761	3958744	472.624	472.95	0.326	0.106276
104029	1373674	3949750	453.353	453.68	0.327	0.106929
105791	1424081	3944930	478.253	478.58	0.327	0.106929
105582	1430488	3937107	492.384	492.71	0.327	0.106929
104045	1373665	3949495	453.212	453.54	0.328	0.107584
104062	1373527	3949178	453.001	453.33	0.329	0.108241
106907	1420761	3959460	475.17	475.5	0.33	0.1089
105765	1423782	3943479	482.849	483.18	0.331	0.109561
105576	1430503	3936796	492.929	493.26	0.331	0.109561
104285	1373230	3948640	451.817	452.15	0.333	0.110889
105774	1423881	3943985	480.437	480.77	0.333	0.110889
104320	1373587	3949268	453.165	453.5	0.335	0.112225
105766	1423785	3943525	482.755	483.09	0.336	0.112896
100833	1340128	3953207	432.782	433.12	0.338	0.114244
104049	1373655	3949429	453.171	453.51	0.339	0.114921
104308	1373465	3949055	452.769	453.11	0.341	0.116281
101229	1340194	3953264	432.823	433.17	0.347	0.120409
106684	1421859	3974928	485.883	486.23	0.347	0.120409
105771	1423831	3943801	481.482	481.83	0.348	0.121104
104314	1373524	3949157	453.019	453.37	0.351	0.123201
104067	1373467	3949074	452.697	453.05	0.353	0.124609
104316	1373545	3949193	453.146	453.5	0.354	0.125316
105773	1423868	3943939	480.545	480.9	0.355	0.126025
100848	1339652	3952710	433.961	434.32	0.359	0.128881
105583	1430482	3937151	492.309	492.67	0.361	0.130321
104310	1373485	3949089	452.828	453.19	0.362	0.131044
104061	1373548	3949214	453.098	453.46	0.362	0.131044
104318	1373566	3949231	453.207	453.57	0.363	0.131769
105772	1423856	3943893	480.757	481.12	0.363	0.131769
104345	1373686	3949746	453.51	453.88	0.37	0.1369
105794	1424091	3945112	477.57	477.94	0.37	0.1369
104059	1373568	3949251	453.059	453.43	0.371	0.137641
104044	1373666	3949510	453.249	453.62	0.371	0.137641
105763	1423779	3943339	483.307	483.68	0.373	0.139129
100860	1339213	3952403	434.506	434.88	0.374	0.139876
105787	1424054	3944707	478.736	479.11	0.374	0.139876
105782	1424001	3944438	479.455	479.83	0.375	0.140625

104050	1373650	3949412	453.183	453.56	0.377	0.142129
104068	1373456	3949056	452.717	453.1	0.383	0.146689
105788	1424067	3944796	478.528	478.92	0.392	0.153664
100854	1339437	3952545	434.407	434.8	0.393	0.154449
105769	1423811	3943708	482.019	482.42	0.401	0.160801
104047	1373662	3949462	453.153	453.56	0.407	0.165649
105784	1424031	3944573	479.081	479.49	0.409	0.167281
105785	1424040	3944618	478.888	479.3	0.412	0.169744
105783	1424012	3944483	479.511	479.93	0.419	0.175561
105786	1424047	3944663	478.829	479.25	0.421	0.177241
104065	1373497	3949126	452.807	453.23	0.423	0.178929
105777	1423930	3944167	479.76	480.19	0.43	0.1849
105789	1424073	3944841	478.453	478.89	0.437	0.190969
105761	1423782	3943244	483.707	484.15	0.443	0.196249
105764	1423779	3943433	483.013	483.46	0.447	0.199809
105779	1423955	3944257	479.595	480.05	0.455	0.207025
105780	1423978	3944347	479.554	480.03	0.476	0.226576
100851	1339547	3952624	434.66	435.14	0.48	0.2304
