# Supplementary material for: High-resolution prediction of American red squirrel in Interior Alaska: a role model for conservation using open access data, machine learning, GIS and LIDAR
Source: PeerJ. 2021 Sep 14;9:e11830. doi: 10.7717/peerj.11830 (PMC8447940; doi:10.7717/peerj.11830)

Elevation QC Report

Project: AK__Fairbanks-North-Star-Borough_201
Contractor: AeroMetric
Data Delivery Date: 06/23/2011
Date Data Reviewed: 08/31/2011
Reviewer: Hannah Boggs
Total Square Miles Reviewed: 409
Elevation Type: LIDAR **Format:** LAS **Grid Spacing:** 4.0 feet **Tile size:** 3km x 3km

Projection: SPCS **Datum:** NAD83 **Units:** Feet

Licensing: Public Domain **Metadata:** Project Level

Materials Received:

LAS:
- All_Points (classified LAS)

Shapefiles:
- Project Footprint Shapefiles:
  - (bounds_main_block.shp)
  - (permafrost_tunnel_boundary.shp)
- Additional Shapefiles:
  - (bounds_permafrost_tunnel.shp)
  - (permafrost_tunnel_surveyed_boundary.shp)
  - (tile_index.shp)

Rasters:
- r_1.img (Fairbanks North Star Borough Data)
- r_1.img (CRREL Permafrost Tunnel Data)

Metadata:
- .IMG metadata (will function as project metadata) (xml format)
- Metadata for all shapefiles delivered (xml format)
- Data report for entire project from AeroMetric (.pdf format)
- QC Analysis Report from AeroMetric (.pdf format)
- LiDAR Calibration Report from AeroMetric (.pdf format)
- Contour metadata (.xml format)
- DEM metadata (.xml format)
- LAS metadata (.xml format)
- Read_me file (.txt format)

Miscellaneous:
- Flight line index (.dwg format)
- Permafrost Tunnel Contours (.mdb format)
- Permafrost Tunnel DEM (.mdb format)
- Fairbanks Contours (.mdb format)
- Fairbanks DEM (.mdb format)

Vertical Accuracy Test Performed: No **Test Point Source:** None **RMSE:** See below

Vertical Accuracy Notes:

Reviewer was unable to conduct a Vertical Accuracy test due to a lack of check points that were of a higher accuracy than the LiDAR data.

Vertical Accuracy of the data was calculated by the contractor. Contractor used ground truth points. Reviewer approves collection method, distribution, and amount of points collected for this dataset. There were 4,280 points well distributed throughout the delivery area.

Reported RMSE is 0.128 feet.
Reported standard deviation (2 sigma): 0.251 feet.

Please see QC Review Summary and NED processing Steps on the following two pages.

DELIVERABLES:

All deliverables are listed on the Read_Me.txt file included in the delivery. The reviewer received most deliverables, however, reviewer found that all databases with a .mdb file extension could not be opened in Microsoft Access. When reviewer opened ArcCatalog and selected the .mdb files with the "contents" tab selected, ArcCatalog showed an empty content window. The .mdb files cannot be previewed and no metadata has been created for these files. Reviewer was unable access the following databases; permafrost_tunnel_contours.mdb, permafrost_tunnel_DEM.mdb, fairbanks_contours.mdb, and fairbanks_DEM.mdb.

METADATA:

Metadata for the DEM data that will function as the project level metadata were run through the Metadata Parser in ArcCatalog. While general information is included and the metadata files reference the FGDC Content Standards for Digital Geospatial Metdata, the metadata parser came up with 11 errors. Metadata is in xml format. These errors will be detailed later in this document.

Reviewer fixed as many errors as possible and created a new project level metadata file for the NED titled AK_Fairbanks-North-Star-Borough_2010.xml. This is the best use metadata file for this project. It is located under the METADATA - Documents folder.

LAS:

Reviewer loaded the LAS "Main_Block" into Global Mapper. Classifications reviewer was able to see in Global Mapper included unclassified, ground, and overlap. Reviewer used several filters (vegetation, building, low noise) however there were no points within these classifications. It should be noted that reviewer did not receive any "grounds" or "bare-earth" LAS files for the NED.

DIGITAL ELEVATION MODELS:

Reviewer loaded the .img file into Global Mapper for review. Reviewer used the projection NAD 83, SPCS Alaska Zone 3 (FIPS 5003), units in feet, for the review. The data was examined at an average scale of 1:5100 throughout the project area.

There was no requirement for treatment of waterbodies or double line streams, therefore waterbodies are not flattened. Reviewer created an errors shapefile showing some of the waterbodies that have not been flattened for reference purposes. Examples are provided below.

Bare earth surface is good. Through the majority of the project, there is not excessive "over-editing" of the bare earth points. All vegetation, buildings and bridges appear to be removed properly. The surface is free from very high points that are most likely noise, artifacts, and quilting.

This data is recommended for NED 1/9th.

PROCESSING STEP FOR THE NED :

Reviewer loaded both .img files into ArcMap. Using the Mosaic to Raster Model, reviewer created a large ArcGrid file. Reviewer loaded both delivered .img datasets into the Mosaic To New Raster model. The reviewer used the 32 bit float parameter, and the blend method because there were over-lapping tiles. Reviewer kept original projection and resolution (NAD 83, SPCS Alaska Zone 3 (FIPS 5003), units in feet). The resulting raster is named mosaic and is located in the NED-FINAL_TO_NED folder.

DIGITAL ELEVATION MODELS:

Reviewer loaded the .img file into Global Mapper for review. Reviewer used the projection NAD 83, SPCS Alaska Zone 3 (FIPS 5003), units in feet, for the review. The data was examined at an average scale of 1:5100 throughout the project area.

There was no requirement for treatment of waterbodies or double line streams, therefore waterbodies are not flattened. Reviewer created an errors shapefile showing some of the waterbodies that have not been flattened for reference purposes. Examples are provided below.

Bare earth surface is good. Through the majority of the project, there is not excessive "over-editing" of the bare earth points. All vegetation, buildings and bridges appear to be removed properly. The surface is free from very high points that are most likely noise, artifacts, and quilting.

This data is recommended for NED 1/9th.

PROCESSING STEP FOR THE NED :

Reviewer loaded both .img files into ArcMap. Using the Mosaic to Raster Model, reviewer created a large ArcGrid file. Reviewer loaded both delivered .img datasets into the Mosaic To New Raster model. The reviewer used the 32 bit float parameter, and the blend method because there were over-lapping tiles. Reviewer kept original projection and resolution (NAD 83, SPCS Alaska Zone 3 (FIPS 5003), units in feet). The resulting raster is named mosaic and is located in the NED-FINAL_TO_NED folder.

AK_Fairbanks-North-Star-Borough_2010: Project Area

Scale = 1:197,421

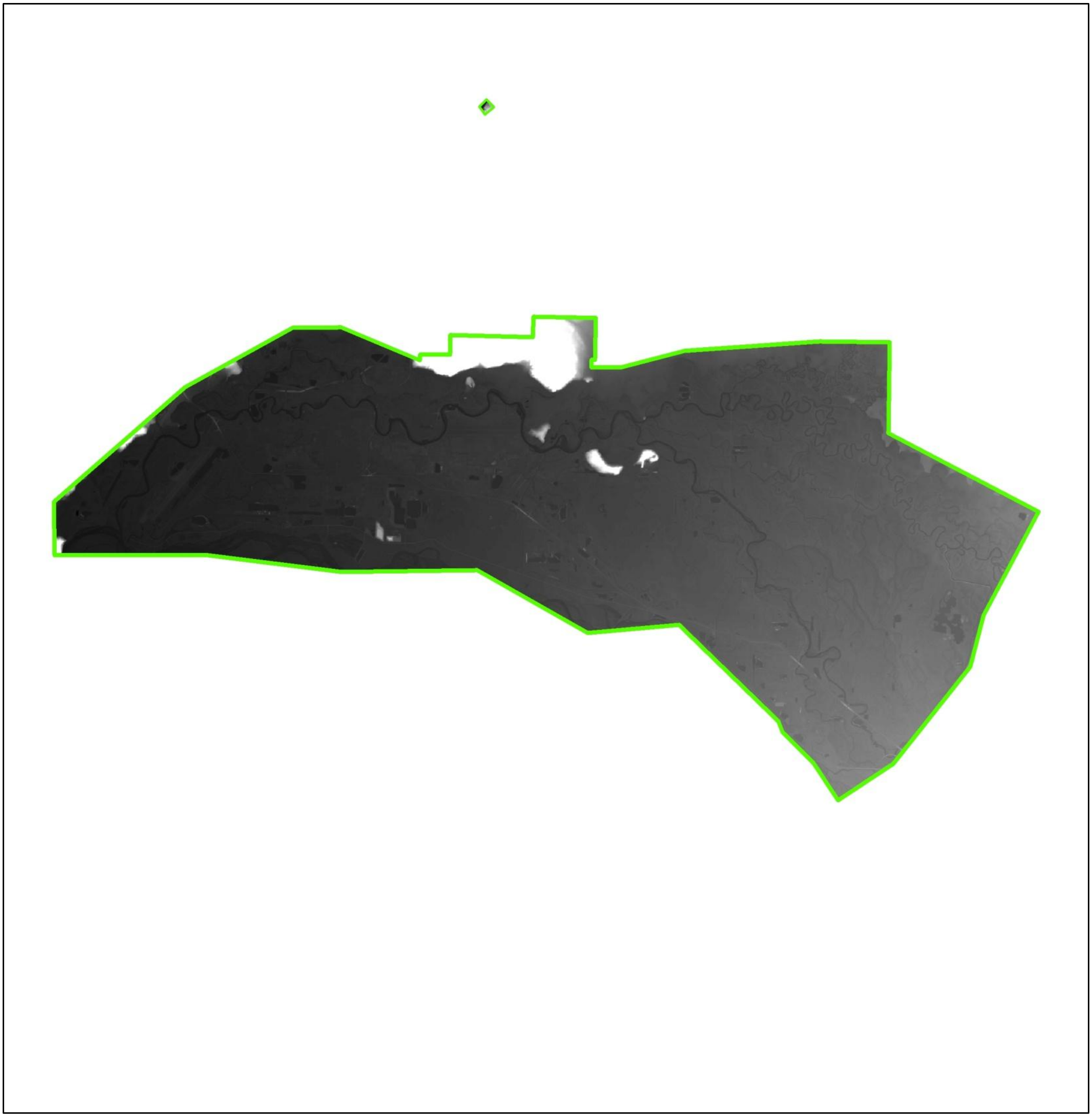

AK_Fairbanks-North-Star-Borough_2010: Project Area Hillshade

Scale = 1:211,548

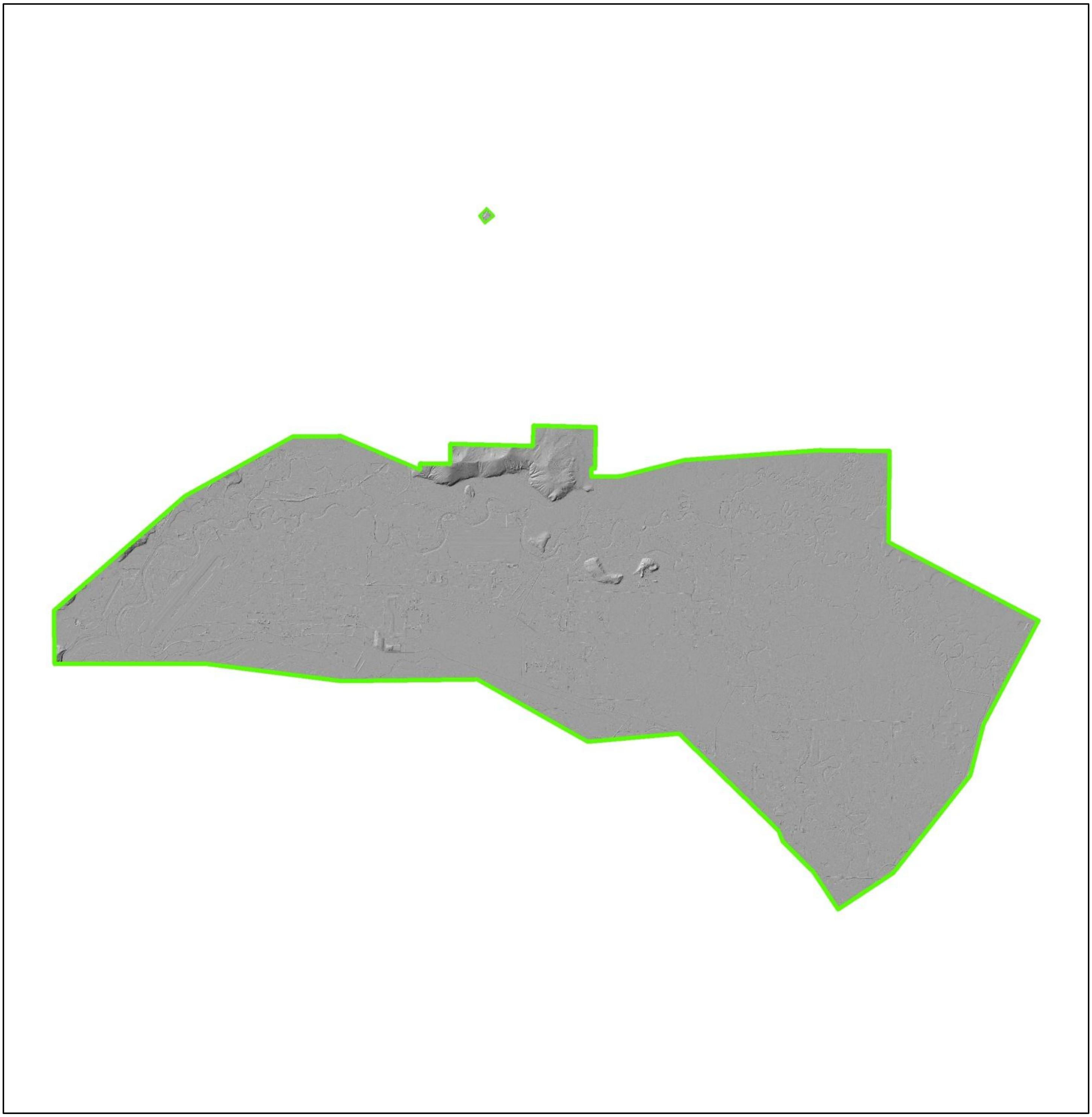

AK_Fairbanks-North-Star-Borough _2010: Project Footprint and LAS tiling scheme

Scale = 1:211,548

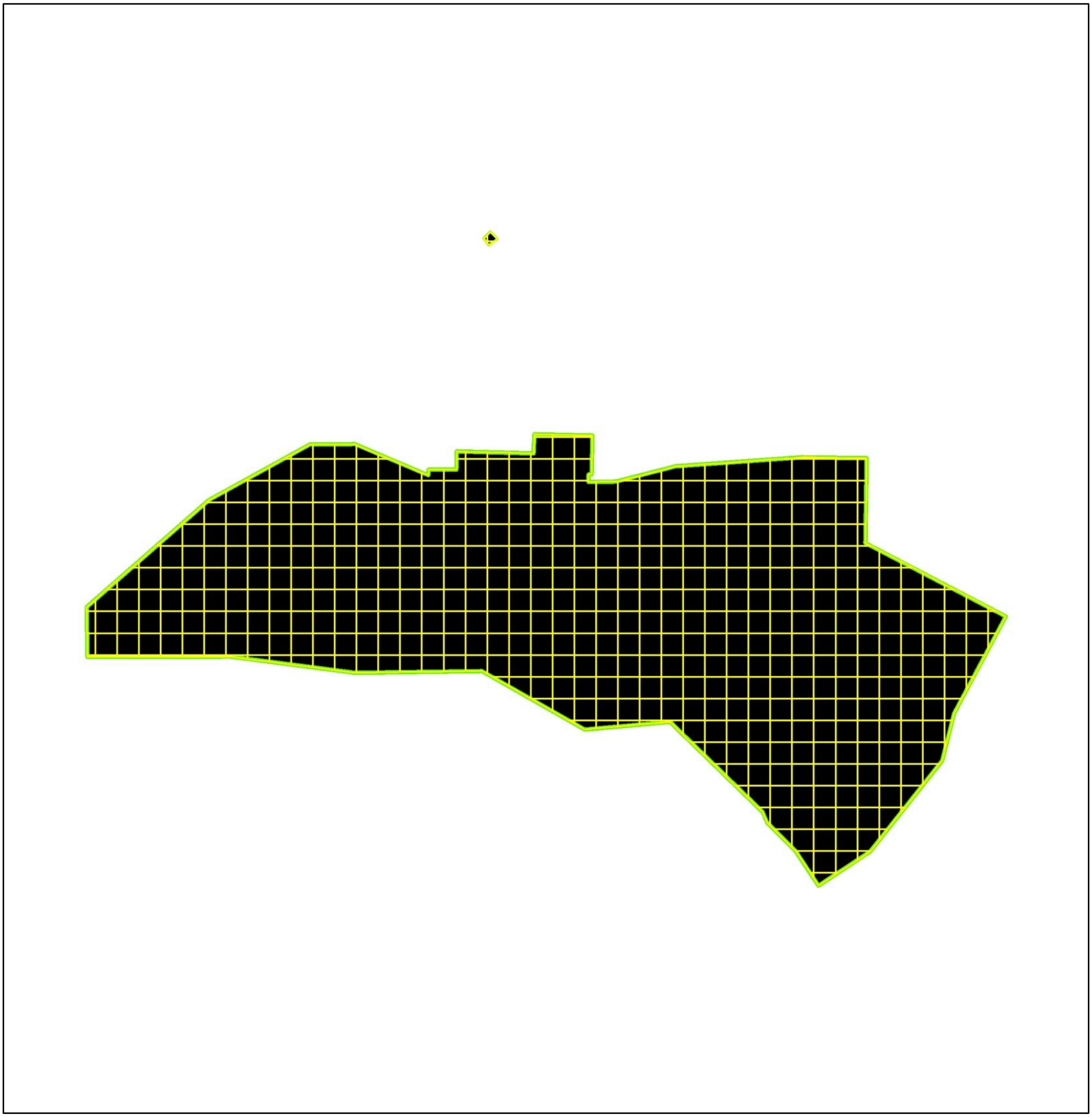

AK_Fairbanks-North-Star-Borough_2010: Example of Waterbodies not Flattened

Scale = 1:16040

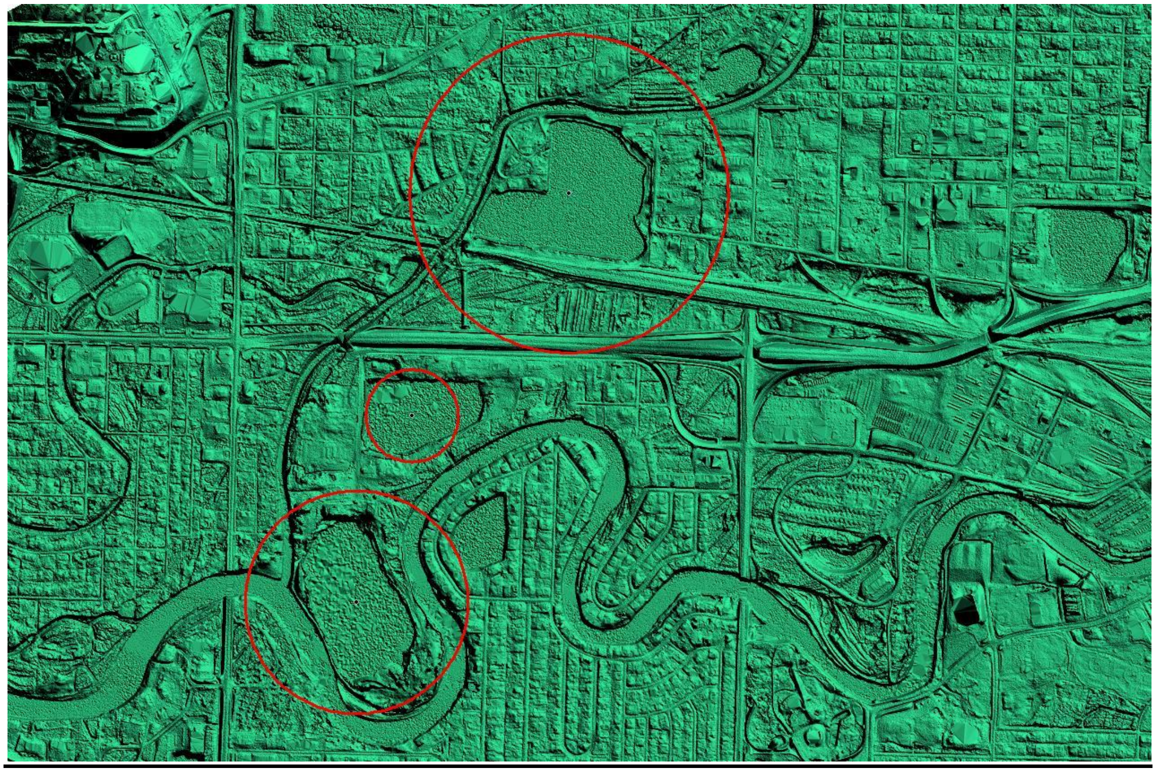

AK_Fairbanks-North-Star-Borough_2010: Example of Waterbody not Flattened (2)

Scale = 1:11160

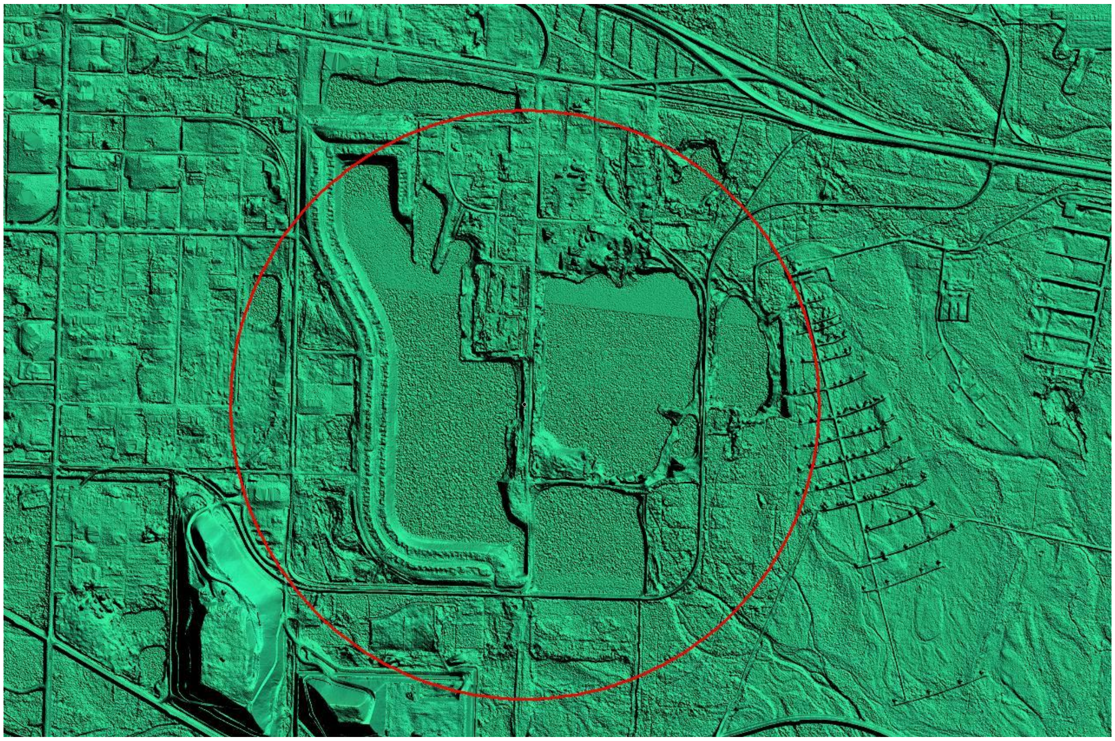

AK_Fairbanks-North-Star-Borough_2010: Errors Found in Metadata Parser

```
Executing: mp D:\LiDAR\Projects\Alaska\metadata_Bare-Earth_DEM.xml # # #
Start Time: Wed Aug 31 14:34:43 2011
Running script mp...
mp D:\LiDAR\Projects\Alaska\metadata_Bare-Earth_DEM.xml
: mp 2.9.6 - Peter N. Schweitzer (U.S. Geological Survey)
: Info: input file = D:\LiDAR\Projects\Alaska\metadata_Bare-Earth_DEM.xml
: Error (line 111): Logical_Consistency_Report is required in Data_Quality_Information
: Error (line 112): Attribute_Accuracy_Report is required in Attribute_Accuracy
: Error (line 127): Source_Citation is required in Source_Information
: Error (line 127): Type_of_Source_Media is required in Source_Information
: Error (line 127): Source_Citation_Abbreviation is required in Source_Information
: Error (line 127): Source_Contribution is required in Source_Information
: Error (line 208): Depth_Datum_Name is required in Depth_System_Definition
: Error (line 208): Depth_Resolution is required in Depth_System_Definition
: Error (line 208): Depth_Distance_Units is required in Depth_System_Definition
: Error (line 208): Depth_Encoding_Method is required in Depth_System_Definition
: Error (line 213): Entity_Type_Definition_Source is required in Entity_Type
: 11 errors: 11 missing
Completed script mp...
Executed (mp) successfully.
End Time: Wed Aug 31 14:34:44 2011 (Elapsed Time: 1.00 seconds)
```

AK_Fairbanks-North-Star-Borough_2010: NGTOC corrected Metadata, Remaining Errors Found in Metadata Parser

```
Executing: mp D:\LiDAR\Projects\Alaska\metadata_Bare-Earth_DEM.xml # # #
Start Time: Wed Aug 31 14:41:50 2011
Running script mp...
mp D:\LiDAR\Projects\Alaska\metadata_Bare-Earth_DEM.xml
: mp 2.9.6 - Peter N. Schweitzer (U.S. Geological Survey)
: Info: input file = D:\LiDAR\Projects\Alaska\metadata_Bare-Earth_DEM.xml
: Error (line 111): Attribute_Accuracy_Explanation is required in
Quantitative_Attribute_Accuracy_Assessment
: Error (line 140): Originator is required in Citation_Information
: Error (line 140): Publication_Date is required in Citation_Information
: Error (line 172): improper value for Grid_Coordinate_System_Name
: Error (line 207): improper value for Depth_Resolution
: Error (line 207): improper value for Depth_Encoding_Method
: 6 errors: 3 missing, 3 bad_value
Completed script mp...
Executed (mp) successfully.
End Time: Wed Aug 31 14:41:50 2011 (Elapsed Time: 0.00 seconds)
```

AK_Fairbanks-North-Star-Borough_2010: FINAL_TO_NED

Scale = 1:122,900

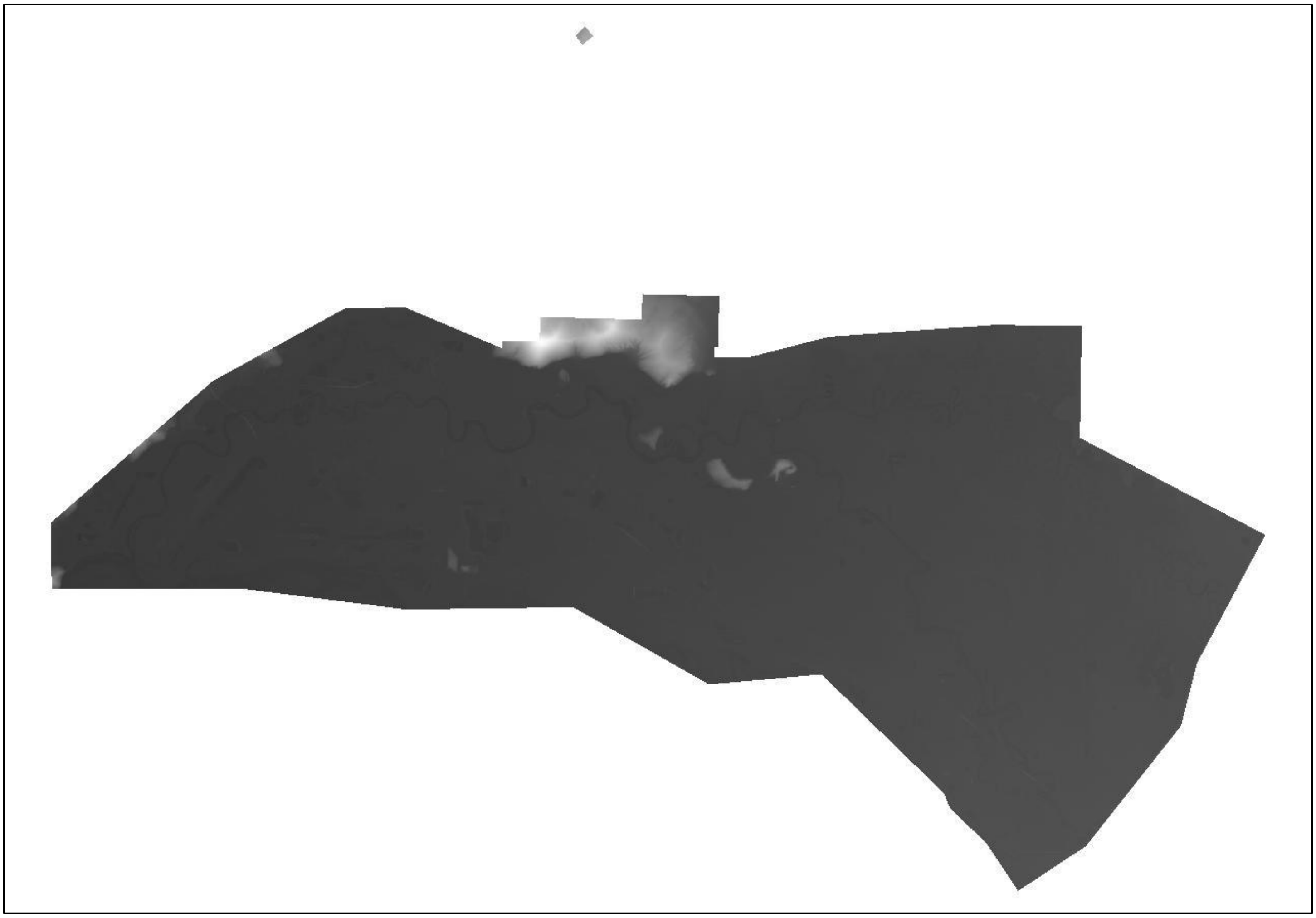

Supplement: Supplemental Information 26 — All metadata contents provided by the Alaska Division for Geographic and Geophysical Surveys for the used LIDAR dataset. [file peerj-09-11830-s026.zip › metadata/AK_Fairbanks-North-Star-Borough_2010.pdf]
