# Supplementary material for: High-resolution prediction of American red squirrel in Interior Alaska: a role model for conservation using open access data, machine learning, GIS and LIDAR
Source: PeerJ. 2021 Sep 14;9:e11830. doi: 10.7717/peerj.11830 (PMC8447940; doi:10.7717/peerj.11830)
Supplement: Supplemental Information 26 — All metadata contents provided by the Alaska Division for Geographic and Geophysical Surveys for the used LIDAR dataset. [file peerj-09-11830-s026.zip › metadata/Fairbanks_Lidar_Report.pdf]

PROCEDURAL REPORT

FOR

FAIRBANKS NORTH STAR BOROUGH LIDAR

AERO-METRIC, INC.

NOVEMBER 2010

Table of Contents

Overview 1
Lidar Acquisition Planning 1
Lidar Acquisition 1
Lidar Processing 1
Lidar Accuracy 2
Deliverables 3
Conclusions 3

Overview

DOWL/HKM awarded Aero-Metric, Inc. a contract to acquire and produce lidar derived mapping products, for the Fairbanks North Star Borough Area, including the CRREL Permafrost Tunnel. This report will outline the planning, processing and quality control procedures used for this project, in the Fairbanks portion of the project. For more information about the CRREL Permafrost Tunnel, see report titled: Permafrost Tunnel Lidar Report, dated Novembe 2010.

Lidar Acquisition Planning

The lidar data for this project was collected with Aero-Metric's Optech Gemini-167 Airborne lidar system (Serial Number 03SEN145). All flight planning and acquisition was completed using Optech's ALTM-Nav, version 1.0.67b (flight planning and lidar control software). The nominal point spacing for this project was 2.7 feet. The following are the planned lidar settings for acquiring the data for this project.

- Flying Height (Above Ground): 4600 feet
- Laser Pulse Rate: 70 kHz
- Mirror Scan Frequency: 48 Hz
- Scan Angle (+/-): 12°
- Side Lap: 50 %
- Ground Speed: 150 kts

Lidar Acquisition

The following are the dates and mission names of the data acquisition:

1. May 4, 2010 (43Q12410B)
2. May 4, 2010 (43Q12410C)
3. May 6, 2010 (43Q12610A)
4. May 6, 2010 (43Q12610B)

DOWL/HKM provided two static GPS base stations located in the project area for airborne GPS control. These points, in conjunction with the two stations operated by Aero-Metric at the Fairbanks airport constituted the basis for the airborne GPS/IMU control.

Lidar Processing

The raw lidar data was merged with the processed ABGPS/IMU data using Optech's ASDA software, which computes an adjusted lidar point cloud for each flight line. Each flight line was then verified for data coverage and consistency with the adjacent flight lines.

Automated classification algorithms in TerraSolid's TerraScan, version 9.011 were used to produce the majority of the bare-earth dataset. The remainder of the data was classified using manual classification techniques.

The digital elevation models and were created from the ground classified lidar points using LP360 (QCoherent Software, LLC.). These were converted to Arc GeoDatabase format.

Lidar Accuracy

The final lidar dataset was verified using 4280 GPS surveyed ground truth points. These points were collected by DOWL/HKM for use in this project. The ground truth points were compared against the lidar using TerraScan, which computes the difference between each ground truth point and the lidar generated surface. These differences are recorded in an output file in ASCII format. This file is imported into Microsoft Excel, where a statistical analysis is performed. The full lidar control check analysis is included in the deliverables for this project, named: Fairbanks_Lidar_QC_Analysis.pdf

For this dataset the vertical accuracy, assessed at the 95% confidence interval (1.96 x RMSE) was 0.251 feet.

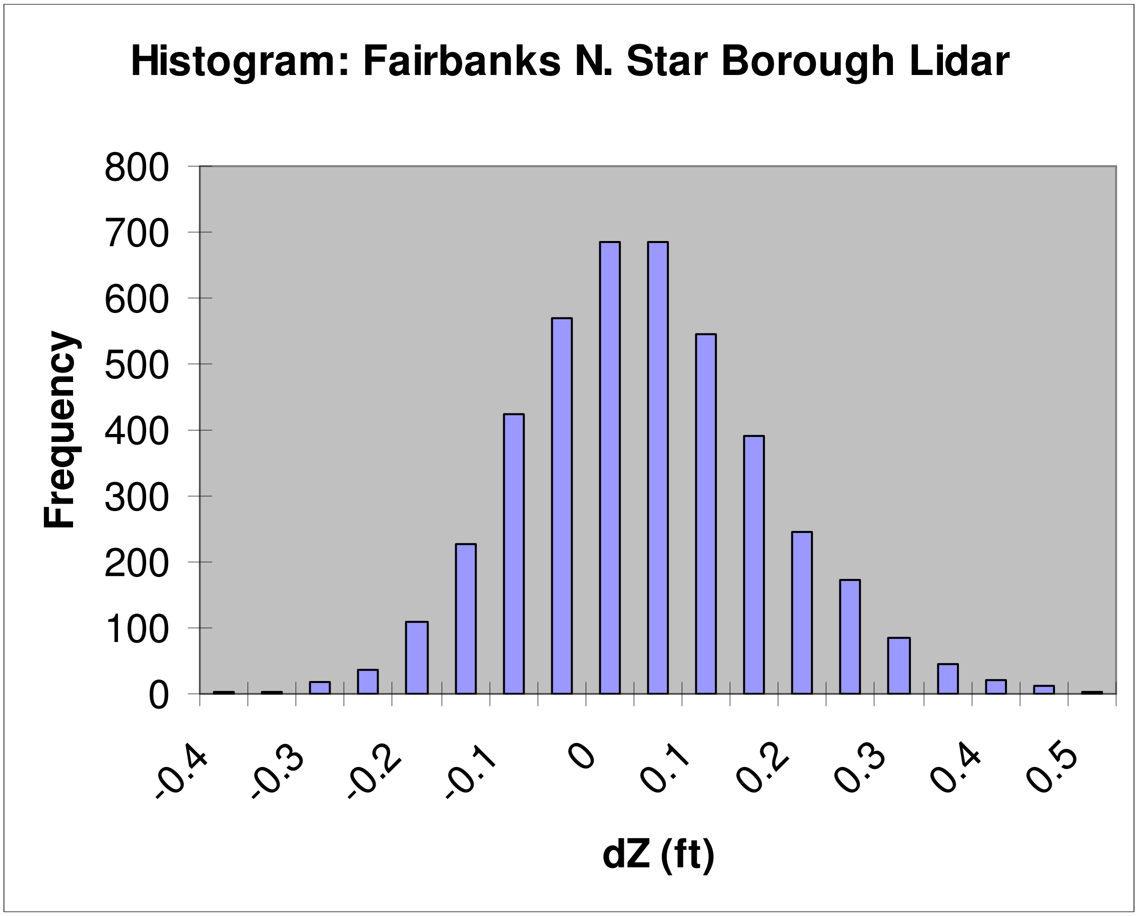

Figure 1: Histogram of QC data check

Deliverables

Project Scope: This report covers the Fairbanks area

Bare-Earth Digital Elevation Model: Derived from the bare-earth processed data, it is in Arc GeoDatabase format. The following are the deliverable parameters:

- Grid Resolution: 4 feet
- 32 bit floating point rasters

LAS Point Clouds: Classified lidar point cloud data, in LAS v1.2 format.

- Included Classes:
  - Class 1: Unclassified
  - Class 2: Ground
  - Class 12: Overlap (Unclassified)
- The tile size is 3,000 x 3,000 meters
- Tiles with the prefix “FNSB” correspond to the main block.

Flight Line Index: AutoCAD2007 drawing file displaying the lidar flight lines.

Contours: 2 foot contours in Arc GeoDatabase format .

FDGC Compliant Metadata: Deliverable level metadata for all delivered lidar datasets, in .xml format.

Reports: Three .PDF files:

1. ***100109_LIDAR_Calibration_Report.pdf*** is the sensor calibration and testing report.
2. ***Fairbanks_Lidar_QC_Analysis.pdf*** is the lidar control check analysis.
3. ***Fairbanks_Lidar_Report.pdf*** is the procedural report (this document).

Tile Index: An index shapefile depicting the tiling scheme of all the lidar data delivered for this project.

Boundary: Shapefile depicting the extents of the deliverables.

Conclusions

The lidar data included in this delivery was acquired for a planned point spacing of approximately 2.7 feet. The vertical accuracy, assessed at the 95% confidence interval was 0.251 feet, on hard, open surfaces. The accuracy of the lidar data was not verified on all surface types, and accuracies may be degraded in areas of steep terrain or heavy vegetation.
