# Supplementary material for: High-resolution prediction of American red squirrel in Interior Alaska: a role model for conservation using open access data, machine learning, GIS and LIDAR
Source: PeerJ. 2021 Sep 14;9:e11830. doi: 10.7717/peerj.11830 (PMC8447940; doi:10.7717/peerj.11830)
Supplement: Supplemental Information 26 — All metadata contents provided by the Alaska Division for Geographic and Geophysical Surveys for the used LIDAR dataset. [file peerj-09-11830-s026.zip › metadata/100109_LIDAR_Calibration_Report.pdf]

Calibration and Testing Report

ALTM Gemini-167
(Serial Number: 03SEN145)

October 2, 2009
Aero-Metric, Anchorage

Table of Contents

Overview 3
Intensity Range Testing .... **Error! Bookmark not defined.**
System Calibration Procedures 4
Calibration Results 5
Conclusions 5

Overview

The purpose of this report is to document the results from the calibration flights collected October 1-2, 2009. The data acquired on October 1 was located at Merrill Field, and collected at 33 and 50kHz. The data acquired on October 2 was located at Palmer, and collected at 70, 100 and 125 kHz.

Aircraft: *Piper Navajo - N6GR*
System: *Optech Gemini-167 LIDAR System (Serial Number: 03SEN145)*
GPS/IMU Processing Software: *Applanix POSPac version 4.4*
Laser Processing Software: *DASHMap version 1.2*
Calibration Software: *TerraScan and TerraMatch (TerraSolid, Ltd.) version 9*

System Calibration Procedures

The system was calibrated for 33, 50, 70, 100, 125 kHz. The flight plans used were 6090303_Merrill_Calibration_100109_V1.pln and 6080103_Lidar_Calibration_Palmer_Airport_V3.pln, and the methodology for calibration is as follows.

1. Lines 1-10 were flown in perpendicular directions. Below are the system settings and date flown for each line.

   Merrill Calibration

	Pulse Rate (kHz)	Scan Freq. (Hz)	Scan Angle (+/-)	Altitude -MSL (m)	Date Flown
Pass 1	50	30	14	2000	10/1/09
Pass 2	50	30	14	2000	10/1/09
Pass 3	50	24	22	2000	10/1/09
Pass 4	50	24	22	2000	10/1/09
Pass 5	33	14	22	4000	10/1/09
Pass 6	33	14	22	4000	10/1/09

   Palmer Calibration*

	Pulse Rate (kHz)	Scan Freq. (Hz)	Scan Angle (+/-)	Altitude -MSL (m)	Date Flown
Pass 5	70	34	22	1500	10/2/09
Pass 6	70	34	22	1500	10/2/09
Pass 7	100	45	22	1000	10/2/09
Pass 8	100	45	22	1000	10/2/09
Pass 9	125	45	22	900	10/2/09
Pass 10	125	45	22	900	10/2/09

   * Passes 1-4 were not acquired, those calibration flights were completed over Merrill Field

2. The GPS/IMU trajectory was computed using Applanix's POSPac version 4.4. Two dual-frequency GPS units were set up at the calibration site, minimizing the baseline length and GPS error during acquisition

3. Each LIDAR pulse was positioned and assigned an intensity values using Optech's DASHMap version 1.2. The following files were used for the initial processing. Each flight line was computed as a separate file in LAS 1.0 format.
   a. ALTM_145_March_2008.res
   b. 2008_03SSEN145_33k.txt
   c. 2008_03SSEN145_50k.txt
   d. 2008_03SSEN145_70k.txt
   e. 2008_03SSEN145_100k.txt
   f. 2008_03SSEN145_125k.txt

4. TerraSolid's TerraMatch module was used to computed any misalignments in roll, pitch and heading, and also compute any systematic mirror scale. These misalignments are computed in an iterative process by comparing the bare-earth surface models from each overlapping flight line of the same frequency and flying height.

5. The corrections computed by TerraMatch were applied to ALTM_145_March_2008.res, and new *.res files were created for each PRF. The data was reprocessed in DASHMap using the appropriately modified *.res file and step 5 was repeated. If the new TerraMatch results were minimal, the corrections were applied correctly and the system was properly calibrated for those settings.

Calibration Results

TerraMatch Results

Frequency-Altitude	Delta Scale	Delta Roll	Delta Pitch	Delta Heading
33k-4000m	0.00190898	-0.01658911	0.00018149	-0.00011360
50k-2000m (14°)	+0.00215	-0.0272	+0.0008	+0.0023
50k-2000m	0.00195903	-0.03192958	0.00057499	-0.00012867
70k-1500m	0.0014966	-0.0096	0.00428246	0.001206
100k-1000m	0.00134889	-0.01801227	0.00686669	-0.00552374
125k-900m	0.00123234	-0.01633449	0.00789717	-0.00957337

Conclusions

The system is operating properly for the pulse rates of 33-125 kHz. No problems were detected during data acquisition and processing. The *.res files created during this calibration can be used for the duration of this installation.
